# Supplementary figures and images for: PiVR: An affordable and versatile closed-loop platform to study unrestrained sensorimotor behavior (part 1 of 2)
Source: PLoS Biol. 2020 Jul 14;18(7):e3000712. doi: 10.1371/journal.pbio.3000712 (PMC7360024; doi:10.1371/journal.pbio.3000712)

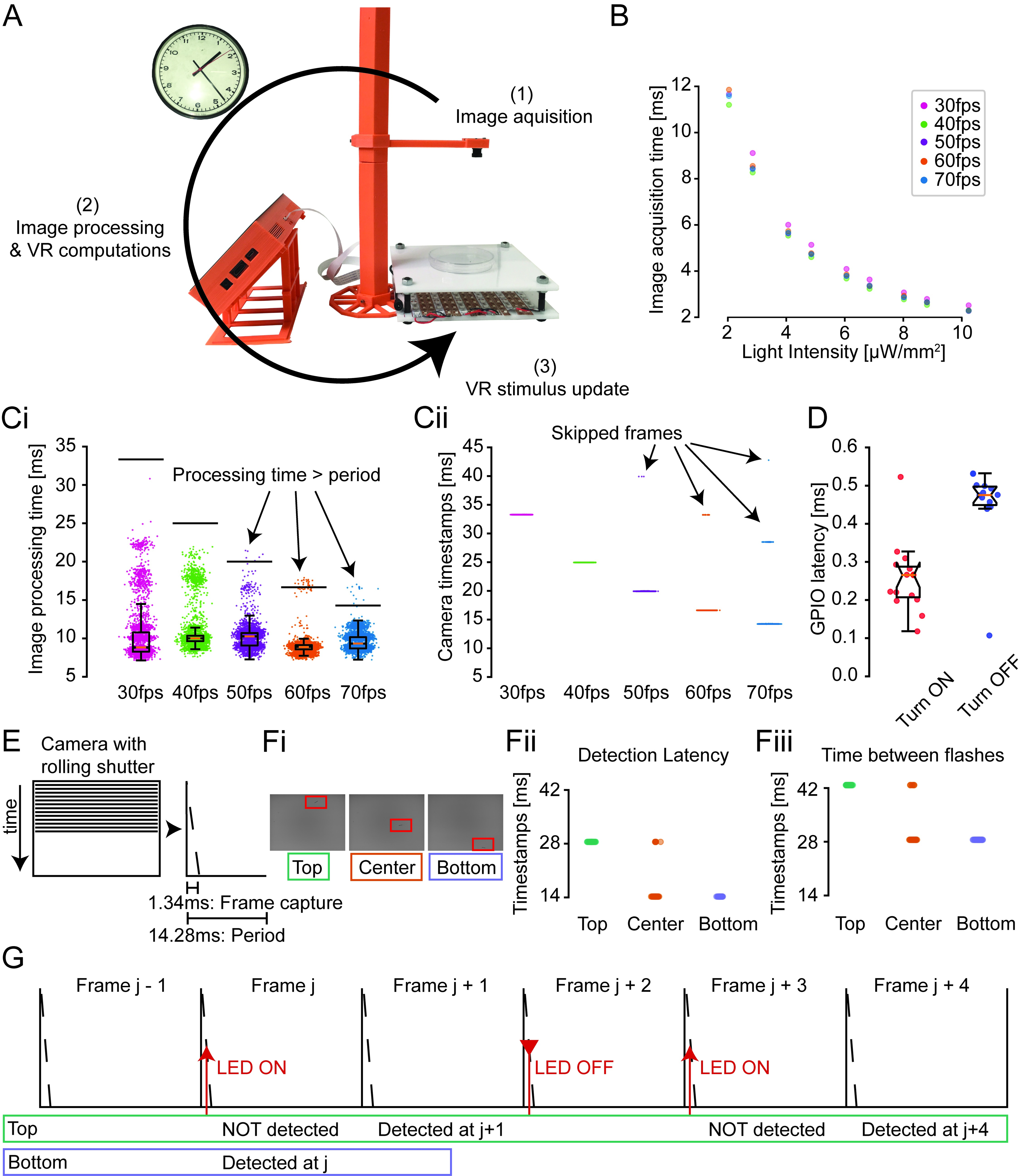

Supplement: S1 Fig — (A) Illustration of the three parameters measured to estimate overall loop time and latency. (B) Dependence of image acquisition time on the infrared background illumination strength. (Ci) To measure image processing and VR computation time, the non-real-time python 3.5 time.time() function is used. At 50, 60, and 70 fps some frames (6/5,598, 25/7,198 and 16/8,398, respectively) take longer than the period of the frame rate, which leads to dropped frames. (Cii) To confirm these measurements, we also recorded timestamps of the images assigned by the real-time clock of the GPU. (D) To estimate the software-to-hardware latency during a full update cycle of the tracker, we measured the time between the GPIO pin being instructed to turn ON and the GPIO pin reporting being turned ON. (E) Cameras with a rolling shutter take images by reading out lines of pixels from top to bottom (E, left). This can be illustrated in a simple x/y plot (E, right). To estimate maximum latency between the animal position and the update of the LED intensity, we used an LED flash paradigm while PiVR was tracking a dummy object at 70 Hz. Our latency results depend on the ROI associated with the location of the dummy object. When PiVR tracks an ROI located at the top of the frame (Fi, left), it detects the LED only two frames later (Fii, left). By contrast, when PiVR tracks an ROI located at the bottom of the frame (Fi, right), it detects the LED in the next possible frame (Fii, right). If PiVR tracks an ROI in the center of the image (Fi, center), it either detects the LED during the next frame or two frames later. We conclude that the LED is being turned ON while the next image is being formed (1.34 milliseconds). For a full LED ON–LED OFF–LED ON sequence, we find that there are three frames between the LED being turned ON when PiVR tracks the top of the image (Fiii, left), whereas it takes two frames when PiVR tracks the bottom of the image (Fiii, right). Because the time between two light fla [file pbio.3000712.s001.tif]

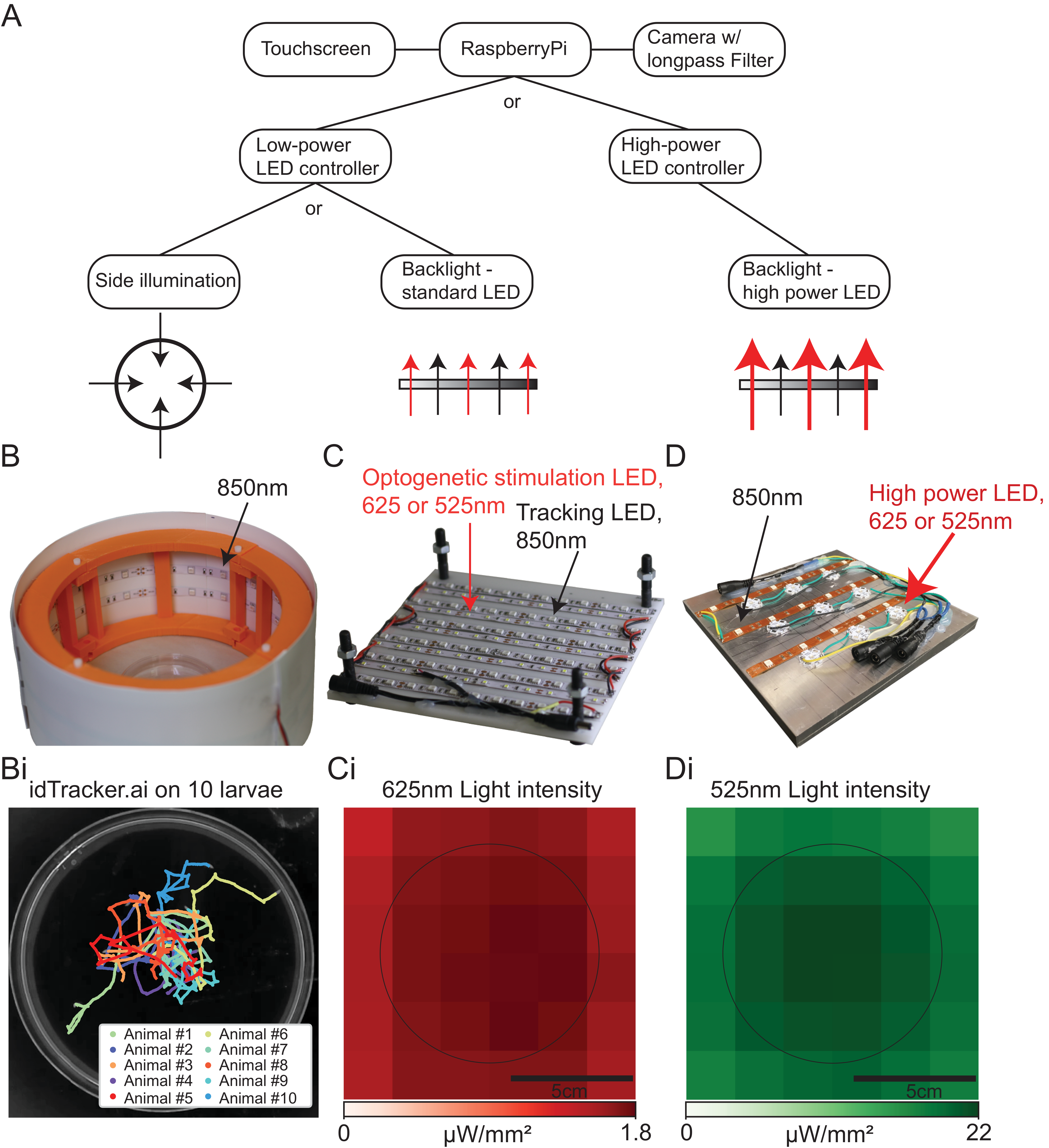

Supplement: S2 Fig — (A) PiVR is highly modular. The standard version (shown in Fig 1A) can easily be adapted to allow for side (or top) illumination using the same LED controller system. If high-light intensities are needed, a high-power LED controller can be used in combination with high-power LEDs. Panel B shows an example of a side illuminator. Side illumination increases contrast on the surface of the animal. (Bi) This side illuminator was used to collect videos with sufficient detail for idtracker.ai [25] to track 10 fruit fly larvae while retaining their identity. (C) The standard PiVR illuminator consists of at least two different 12-V LED strips: one for background illumination (850 nm) and another color (here, 625 nm) to stimulate the optogenetic tool of choice. (D) The high-power stimulation arena uses an LED controller that keeps current to high-power LEDs constant and can drive a maximum of 12 high-power LEDs. (Ci and Di) The intensity of the stimulation light was measured using a Photodiode (Thorlabs Inc. S130VC). Each pixel is 2 cm2. The black circles indicate petri dishes used as behavioral arenas. LED, light-emitting diode; PiVR, Raspberry Pi Virtual Reality. (TIF) [file pbio.3000712.s002.tif]

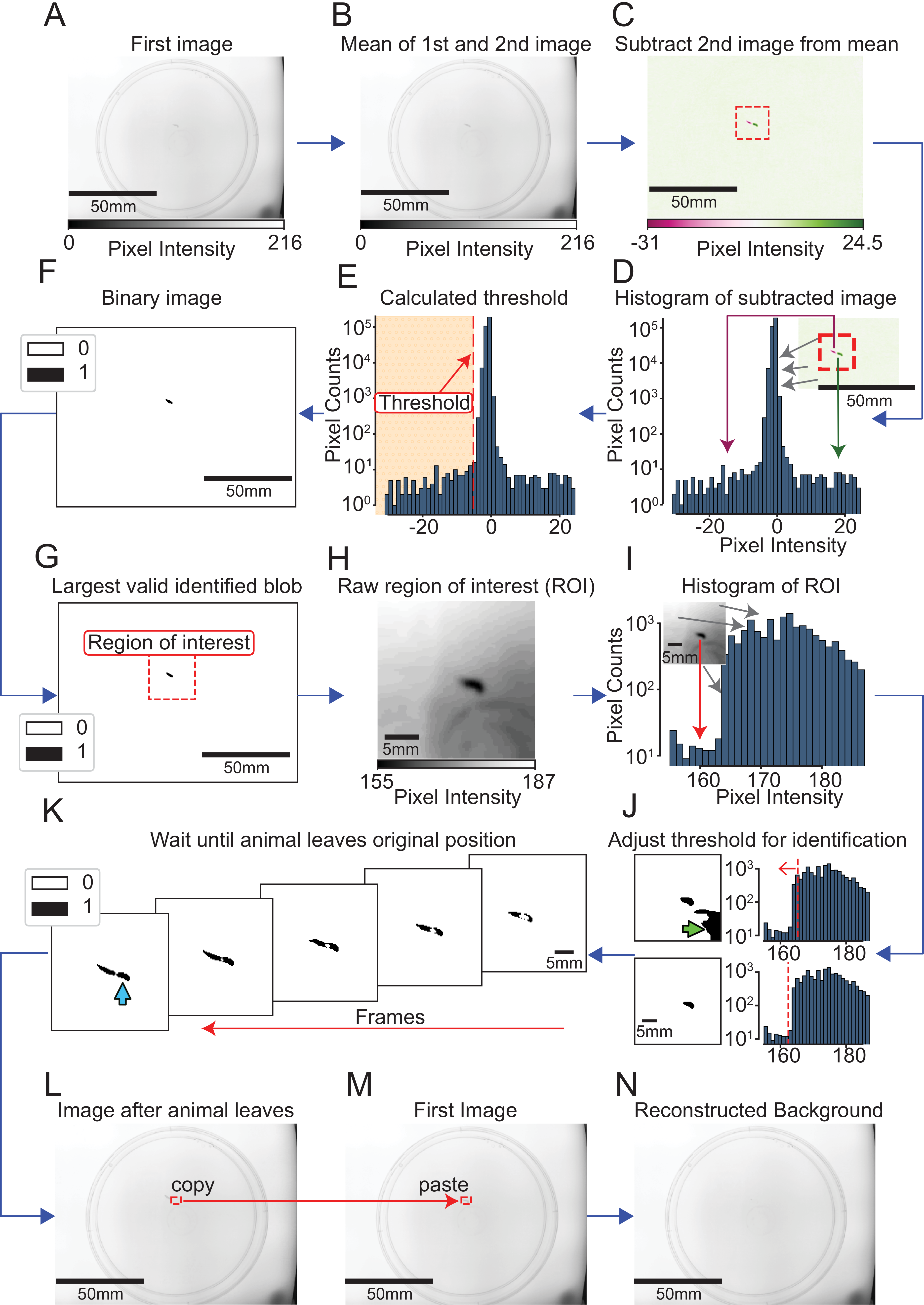

Supplement: S3 Fig — (A) After placing the animal and pressing “start tracking” on the graphical user interface, the software will grab the first image. All images are immediately filtered using a Gaussian kernel with a sigma depending on the size of the animal (user defined) to reduce camera noise. (B) Then a second image is taken. The mean of the images taken so far is being calculated. (C) The current image (in this example, the second image of the experiment) is then subtracted from the mean image shown in panel B. (D) The histogram of the subtracted image shows most gray scale values to be 0. For the region where the animal has moved since the first frame, the pixel values are negative (magenta). For the region that the animal has left since the first frame, the pixel values are positive (green). (E) The threshold value is calculated based on the histogram: it is the mean of the image subtracted by 4 (optimal value defined by trial and error). (F) The threshold value is used to binarize the subtracted image shown in panel C. If there is no or more than one blob with a minimal area (defined by user in animal parameters file), the loop restarts at step (B). (G) If there is exactly one blob, an area around the blob (defined by user in animal parameters file) is defined as the current region of interest. (H) The region of interest is the area where movement has been detected. The algorithm will now restrict the search for the animal to this region. (I) The histogram of this small area of the first image (A) shows that the few pixel defining the animal are distinct from the background. (J) To find the optimal local threshold for binarizing the image, the threshold is adjusted if more than one blob is detected (top, green arrow). As soon as only one blob with the characteristics of the animal (defined by user in animal parameters file) has been detected, the local threshold value is set, and the shape of the identified animal in the first frame is saved (bottom). (K) Using the local thre [file pbio.3000712.s003.tif]

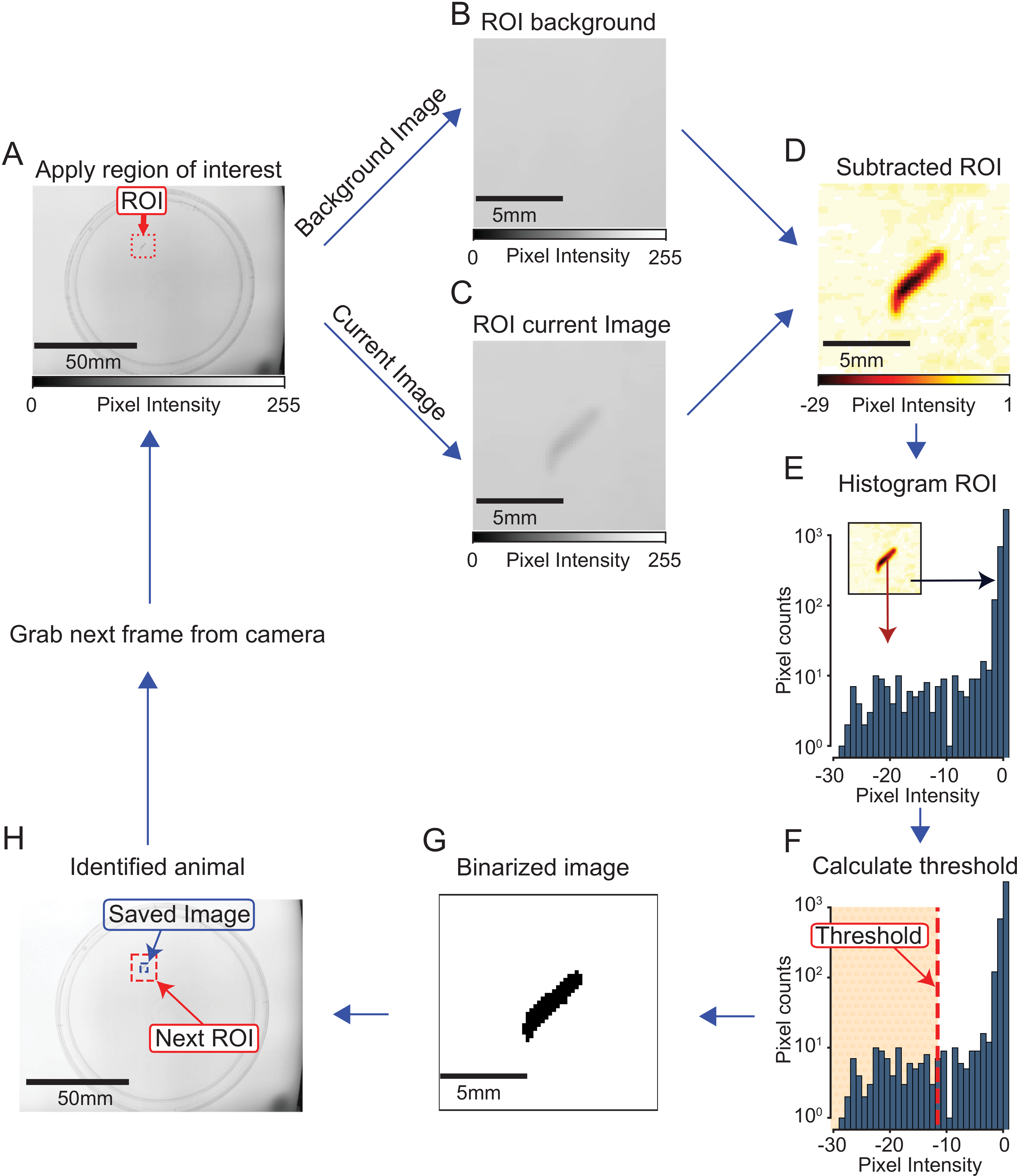

Supplement: S4 Fig — (A) At the start of the experiment, the ROI is defined during animal detection (S3G Fig). During the experiment, the current ROI is defined using the previous frame. The ROI of the current image (C) is then subtracted from the ROI of the background (B). The fact that the tracking algorithm only considers a subsample of the image is central to the temporal performances (short processing time) of PiVR. (D) In the resulting image, the animal clearly stands out relative to the background. (E) The histogram of the image indicates that whereas the background consists mostly of values around 0, the animal has pixel intensity values that are negative. (F) The threshold is defined as being three standard deviations away from the mean (G). This threshold is used to binarize the subtracted ROI. The largest blob with animal characteristics (defined by animal parameters) is defined to be the animal. (H) The image of the detected animal is saved, and the next ROI is designated (defined by animal parameters). All data used to create these plots are available from https://doi.org/10.25349/D9ZK50. PiVR, Raspberry Pi Virtual Reality; ROI, region of interest. (TIF) [file pbio.3000712.s004.tif]

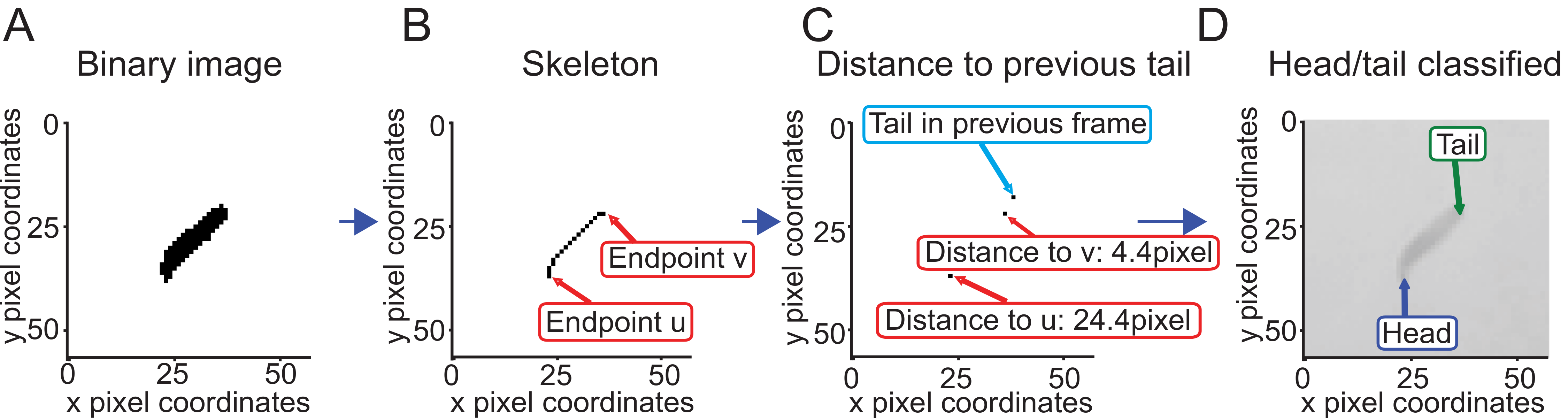

Supplement: S5 Fig — (A) During tracking, head/tail classification starts with the binarized image (S4G Fig). (B) The binary image is used to calculate the morphological skeleton, which in turn is used to identify the two endpoints, one of which must be the head and the other the tail. (C) The Euclidian distance between the tail position in the previous frame and each of the endpoints is calculated. If the tail was not defined in the previous frame, the centroid position is used instead. (D) Whichever endpoint has less distance is defined as the tail (here v). The other endpoint is defined as the head. (TIF) [file pbio.3000712.s005.tif]

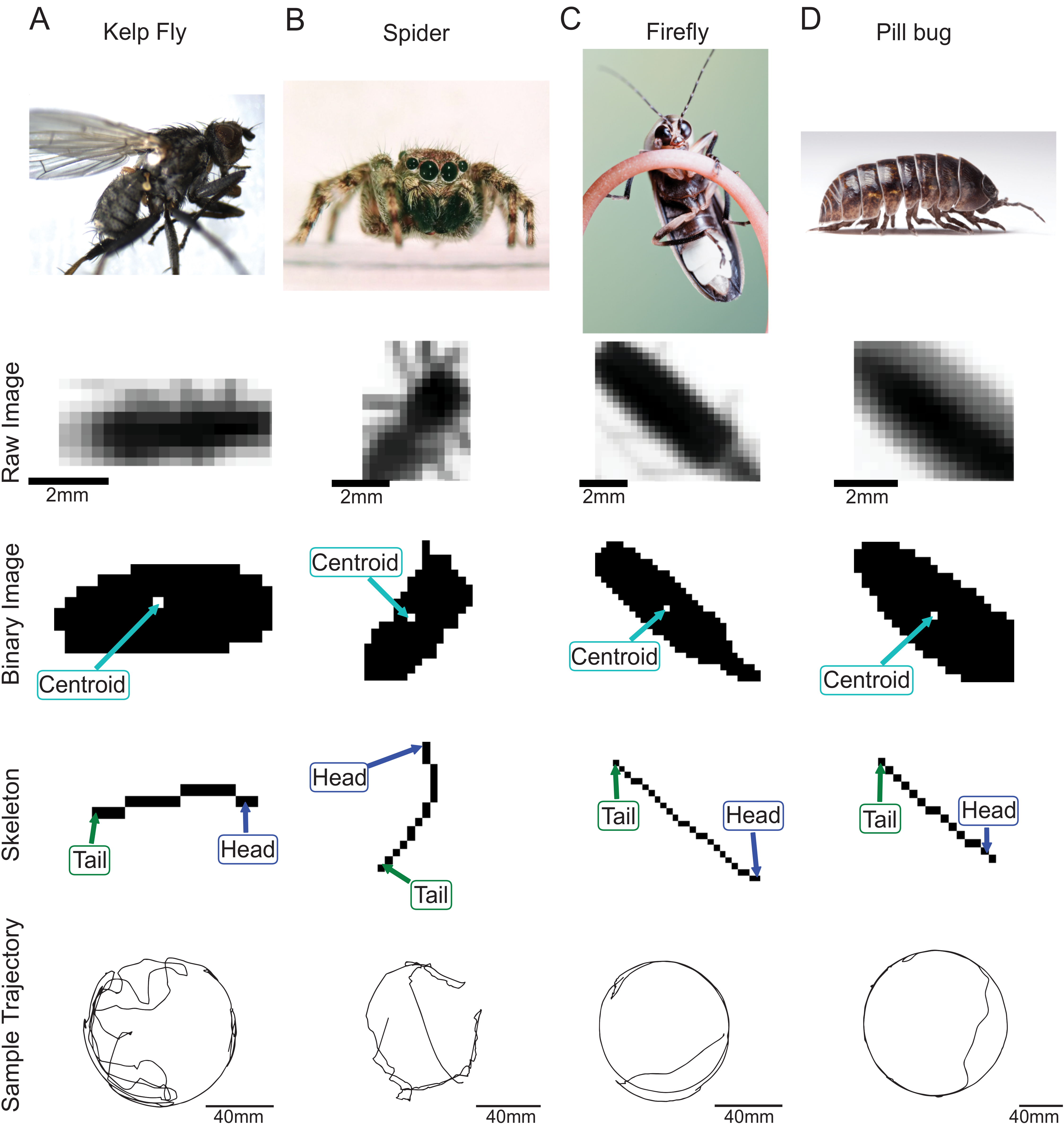

Supplement: S6 Fig — PiVR is able to detect, track, and assign head and tail positions to a variety of invertebrate species with different body plans: (A) kelp fly, (B) jumping spider, (C) firefly, and (D) pill bug. PiVR, Raspberry Pi Virtual Reality. (TIF) [file pbio.3000712.s006.tif]

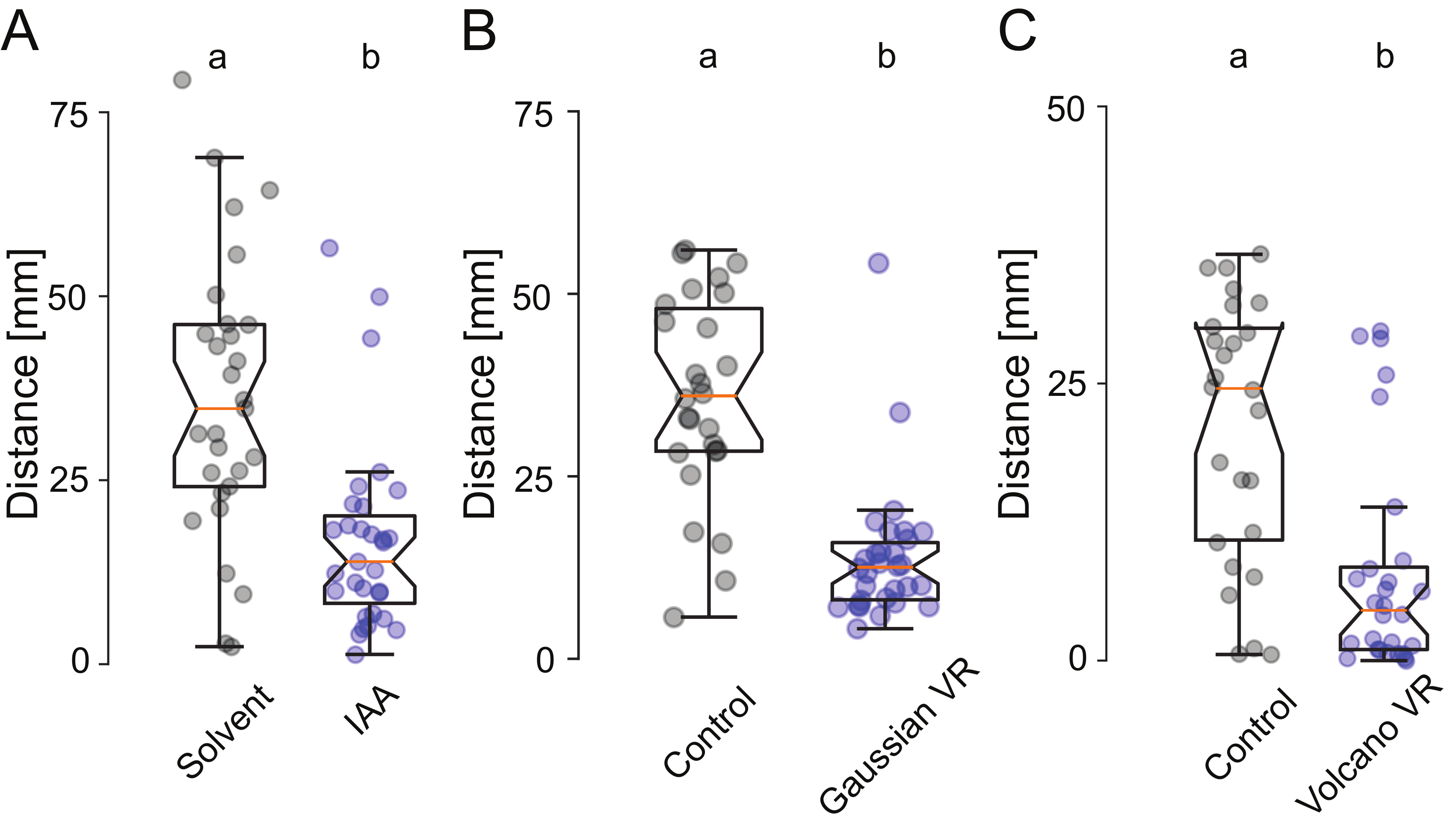

Supplement: S7 Fig — (A) Distance to real-odor source (isoamyl acetate, n = 30) and the solvent (paraffin oil, n = 30), (B) between the Gaussian-shaped virtual-odor reality (n = 31) and the control (n = 26), and (C) the distance to the local maximum (rim of the volcano, n = 29) and the control (n = 26). Time point is 4 minutes into the experiment (Mann–Whitney U test, p < 0.001). All data used to create these plots are available from https://doi.org/10.25349/D9ZK50. (TIF) [file pbio.3000712.s007.tif]

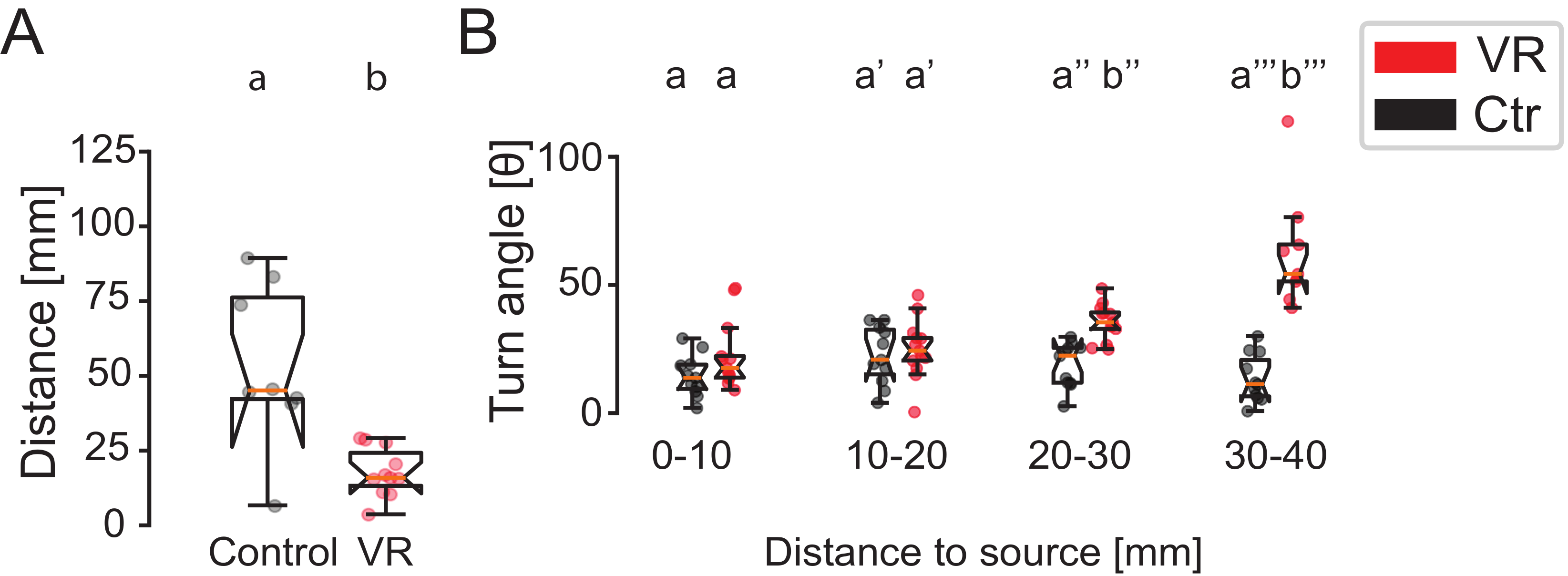

Supplement: S8 Fig — (A) Distance to virtual-light source of control (black) and experimental condition (red) at 4 minutes into the experiment. (B) Relationship between turn angle θ and distance to the virtual-light source (Mann–Whitney U test, different letters indicate p < 0.01, n = 11 and 13). All reported p-values are Bonferroni corrected. All data used to create these plots are available from https://doi.org/10.25349/D9ZK50. (TIF) [file pbio.3000712.s008.tif]

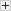

Supplement: S1 HTML — PiVR, Raspberry Pi Virtual Reality. (ZIP) [file pbio.3000712.s019.zip › S1HTLM/_static/plus.png]

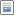

Supplement: S1 HTML — PiVR, Raspberry Pi Virtual Reality. (ZIP) [file pbio.3000712.s019.zip › S1HTLM/_static/file.png]

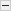

Supplement: S1 HTML — PiVR, Raspberry Pi Virtual Reality. (ZIP) [file pbio.3000712.s019.zip › S1HTLM/_static/minus.png]

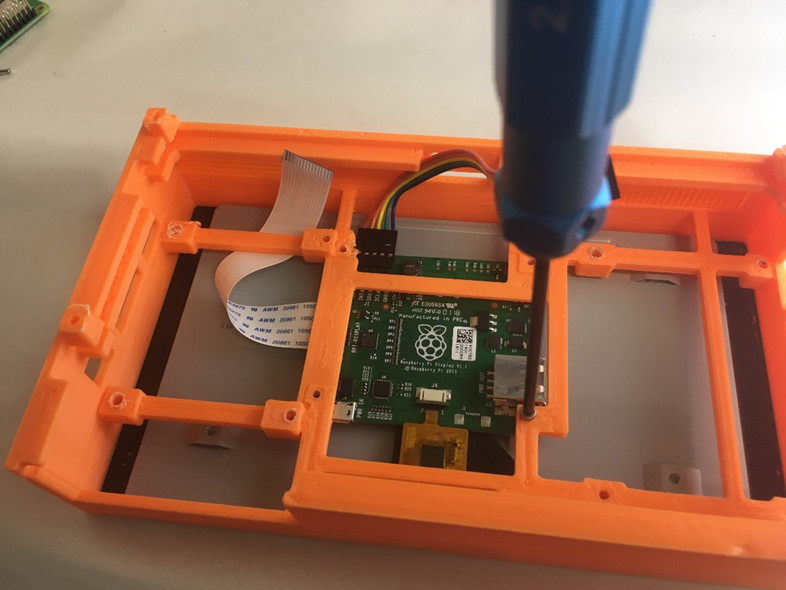

Supplement: S1 HTML — PiVR, Raspberry Pi Virtual Reality. (ZIP) [file pbio.3000712.s019.zip › S1HTLM/_images/7_2_Casing_on_Touchscreen.jpg]

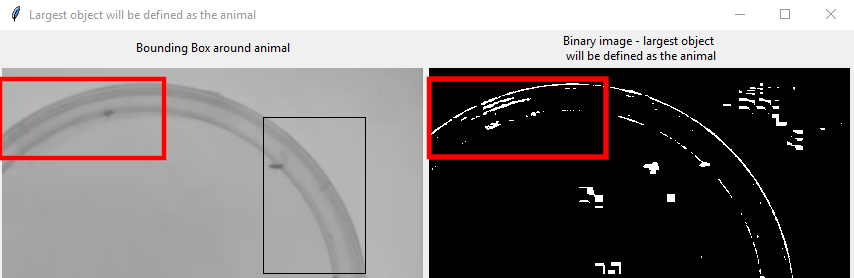

Supplement: S1 HTML — PiVR, Raspberry Pi Virtual Reality. (ZIP) [file pbio.3000712.s019.zip › S1HTLM/_images/16_detection_problems1.png]

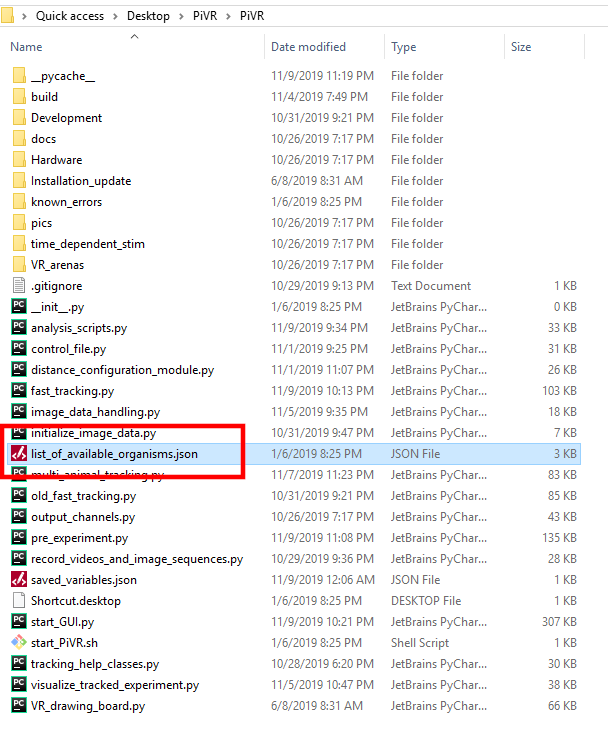

Supplement: S1 HTML — PiVR, Raspberry Pi Virtual Reality. (ZIP) [file pbio.3000712.s019.zip › S1HTLM/_images/7_open_organism_json.png]

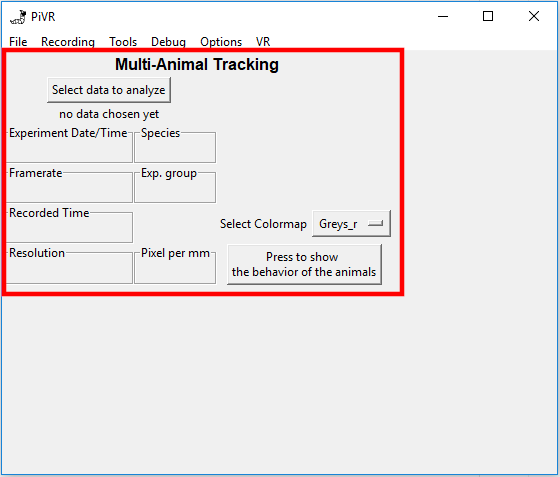

Supplement: S1 HTML — PiVR, Raspberry Pi Virtual Reality. (ZIP) [file pbio.3000712.s019.zip › S1HTLM/_images/6_MultiAnimalTracker.png]

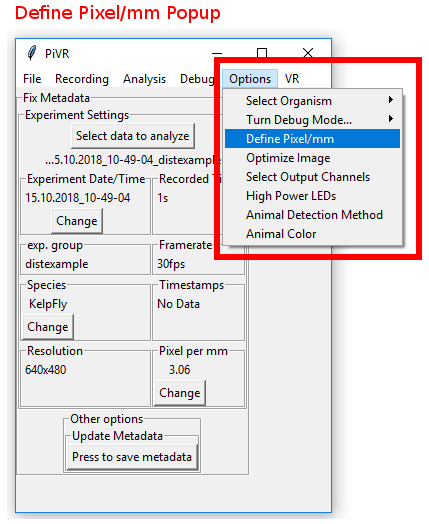

Supplement: S1 HTML — PiVR, Raspberry Pi Virtual Reality. (ZIP) [file pbio.3000712.s019.zip › S1HTLM/_images/OptionsDefinePxMm.png]

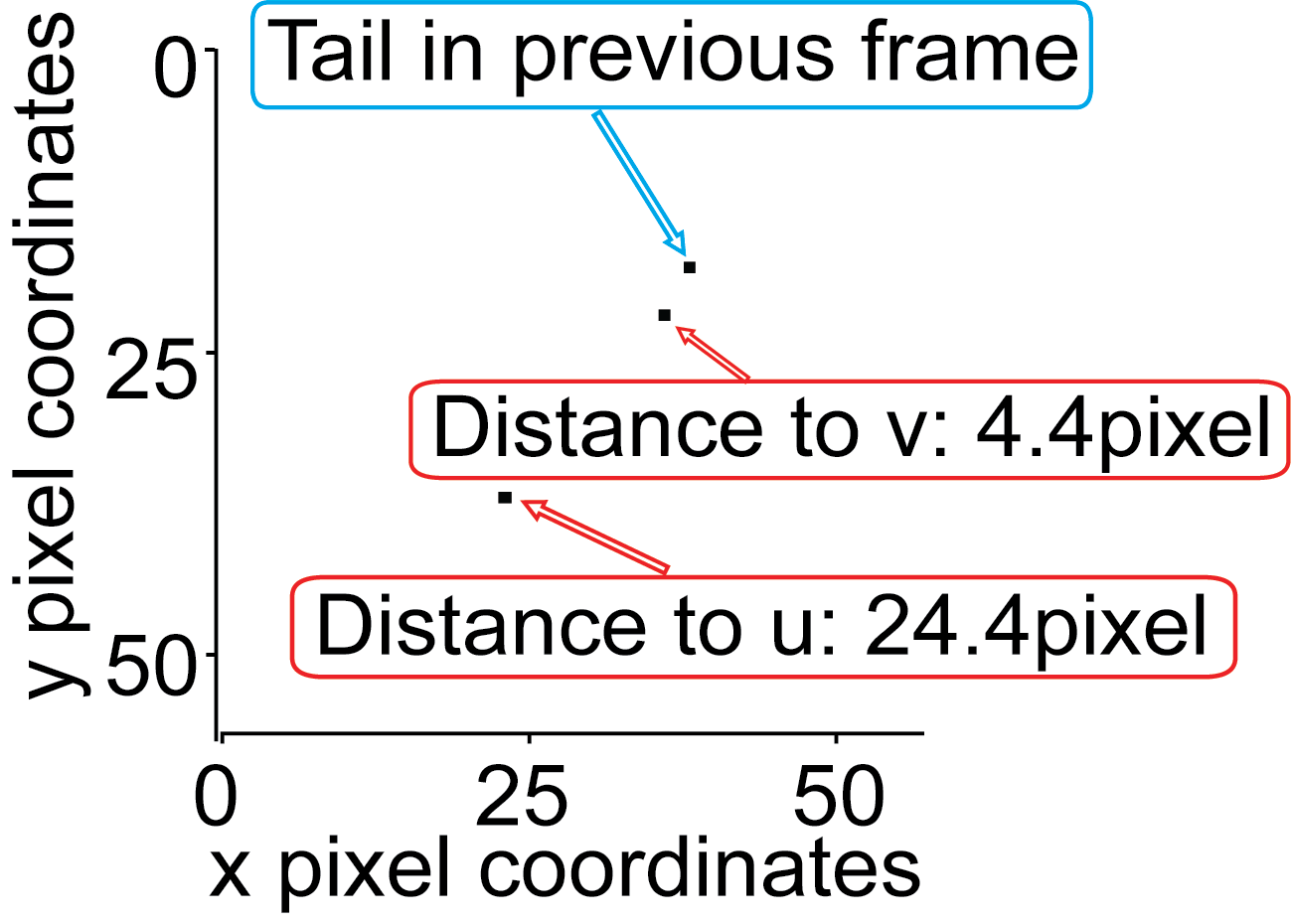

Supplement: S1 HTML — PiVR, Raspberry Pi Virtual Reality. (ZIP) [file pbio.3000712.s019.zip › S1HTLM/_images/FigS5_DistanceEndpoints.png]

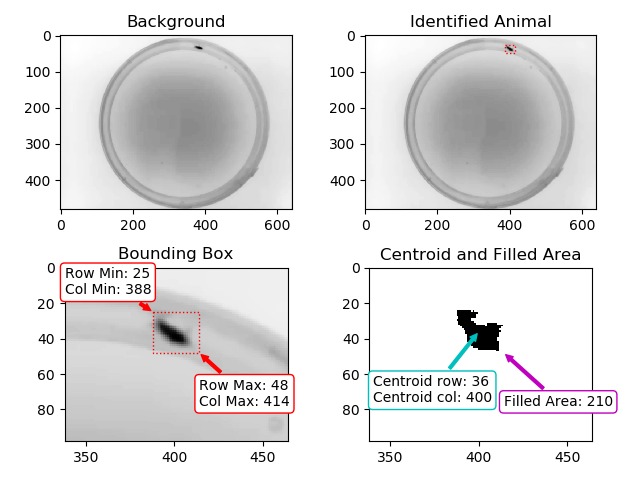

Supplement: S1 HTML — PiVR, Raspberry Pi Virtual Reality. (ZIP) [file pbio.3000712.s019.zip › S1HTLM/_images/Mode1Output.png]

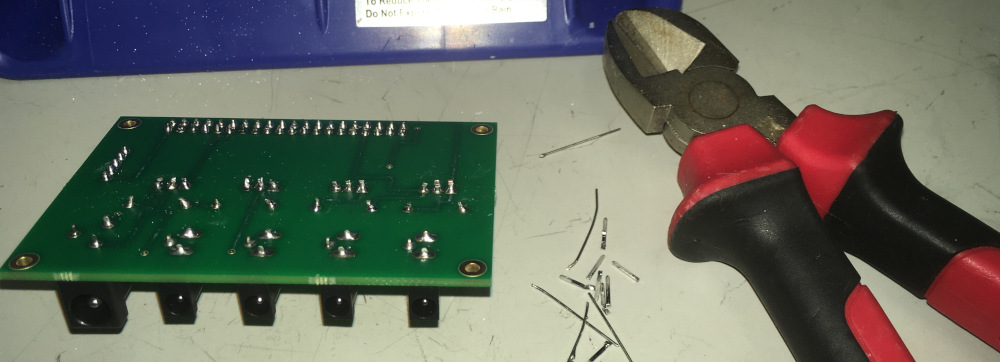

Supplement: S1 HTML — PiVR, Raspberry Pi Virtual Reality. (ZIP) [file pbio.3000712.s019.zip › S1HTLM/_images/S_16.jpg]

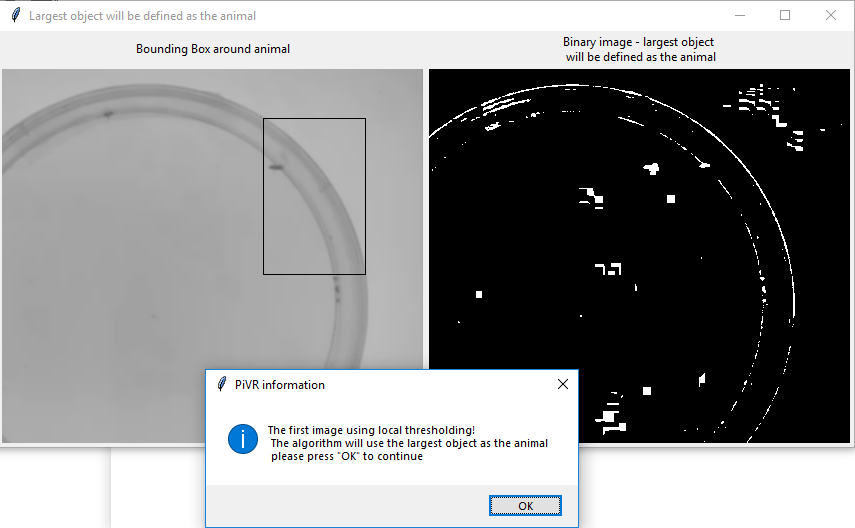

Supplement: S1 HTML — PiVR, Raspberry Pi Virtual Reality. (ZIP) [file pbio.3000712.s019.zip › S1HTLM/_images/14_simulate_tracking_detection_broken.png]

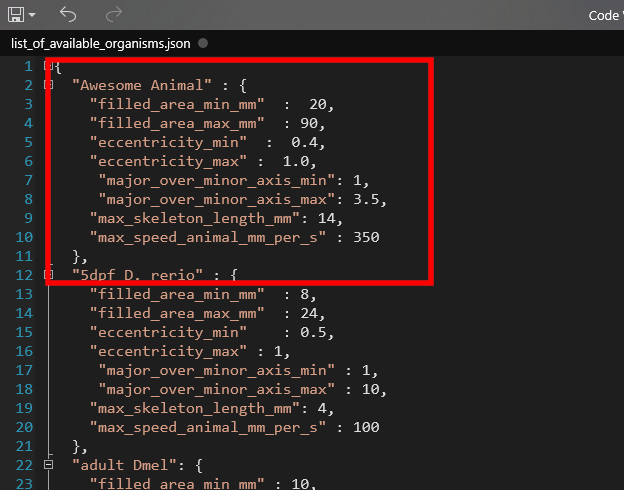

Supplement: S1 HTML — PiVR, Raspberry Pi Virtual Reality. (ZIP) [file pbio.3000712.s019.zip › S1HTLM/_images/9_modified_organism_json.png]

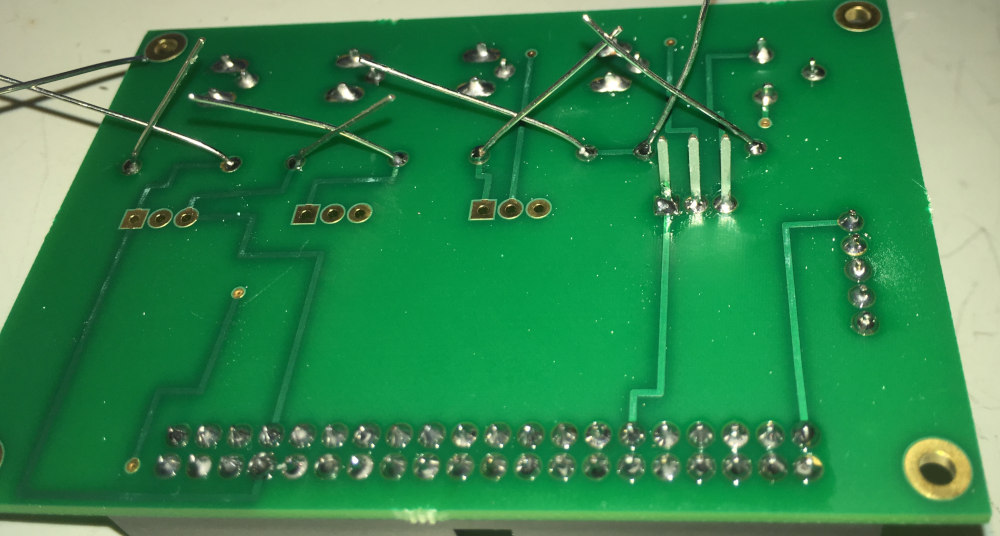

Supplement: S1 HTML — PiVR, Raspberry Pi Virtual Reality. (ZIP) [file pbio.3000712.s019.zip › S1HTLM/_images/S_14.jpg]

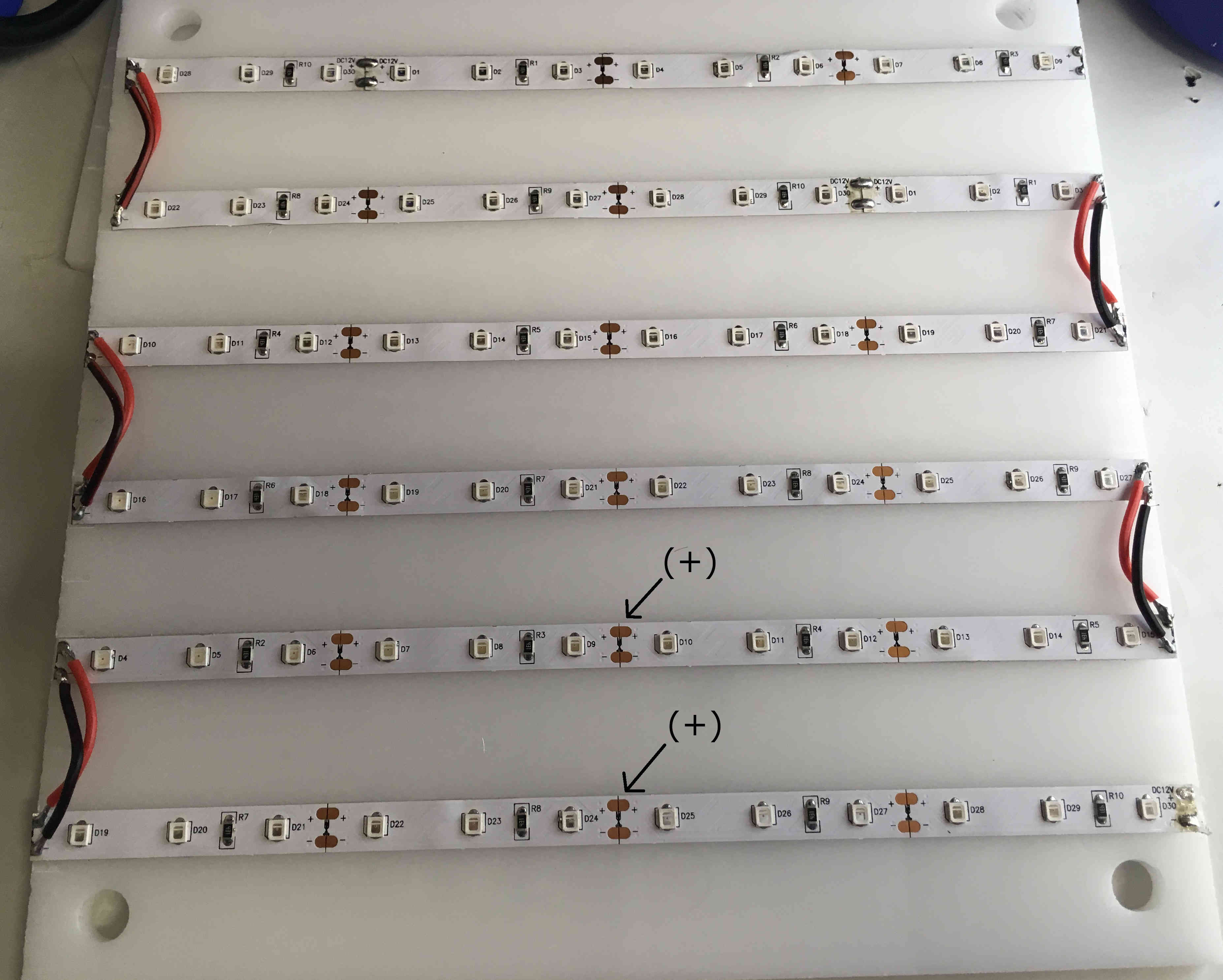

Supplement: S1 HTML — PiVR, Raspberry Pi Virtual Reality. (ZIP) [file pbio.3000712.s019.zip › S1HTLM/_images/23_soldered_arena.jpg]

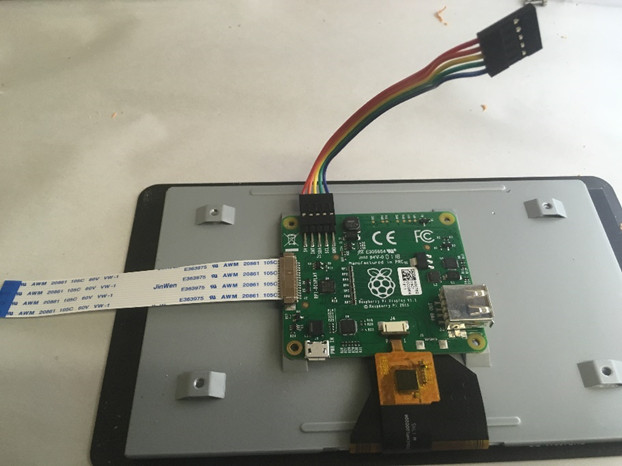

Supplement: S1 HTML — PiVR, Raspberry Pi Virtual Reality. (ZIP) [file pbio.3000712.s019.zip › S1HTLM/_images/6_Touchscreen.jpg]

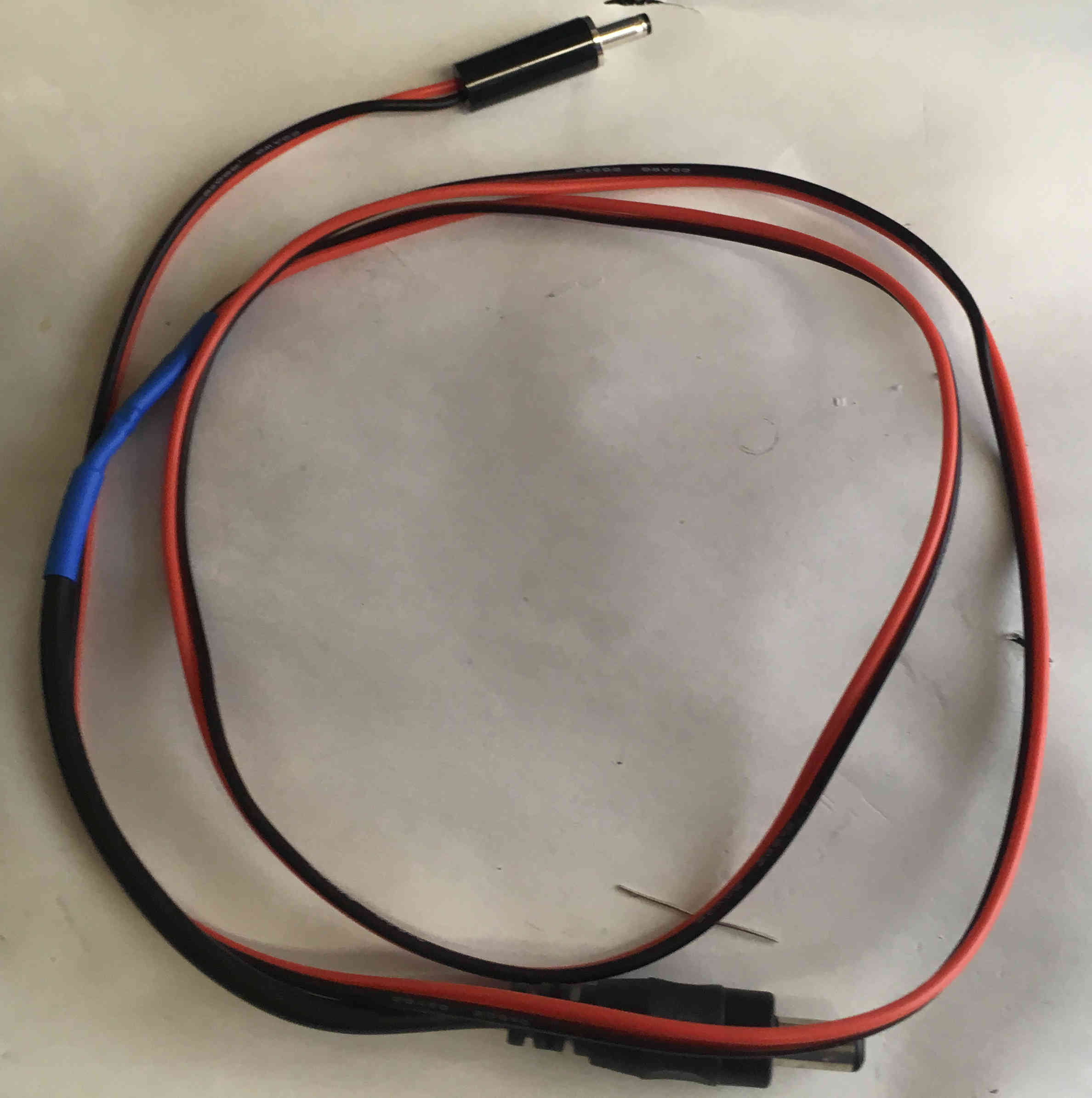

Supplement: S1 HTML — PiVR, Raspberry Pi Virtual Reality. (ZIP) [file pbio.3000712.s019.zip › S1HTLM/_images/28_cable_done.jpg]

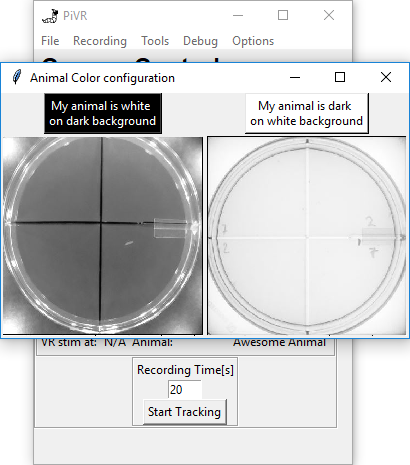

Supplement: S1 HTML — PiVR, Raspberry Pi Virtual Reality. (ZIP) [file pbio.3000712.s019.zip › S1HTLM/_images/AnimalColorOptions.png]

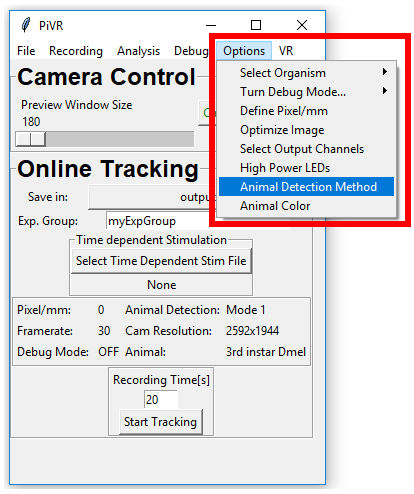

Supplement: S1 HTML — PiVR, Raspberry Pi Virtual Reality. (ZIP) [file pbio.3000712.s019.zip › S1HTLM/_images/OptionsAnimalDetectionMethods.png]

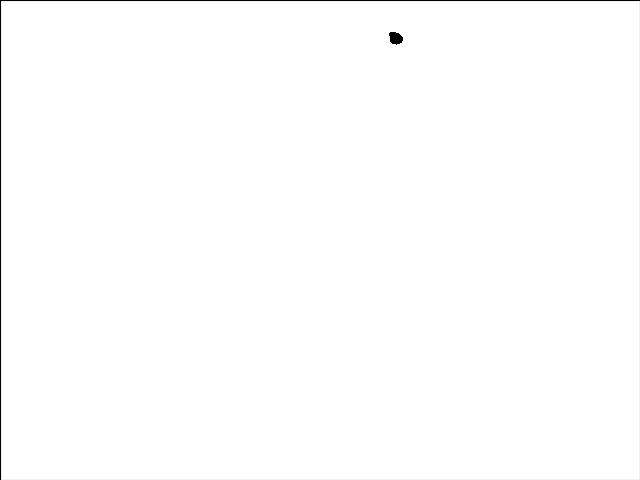

Supplement: S1 HTML — PiVR, Raspberry Pi Virtual Reality. (ZIP) [file pbio.3000712.s019.zip › S1HTLM/_images/Mode1Binary.png]

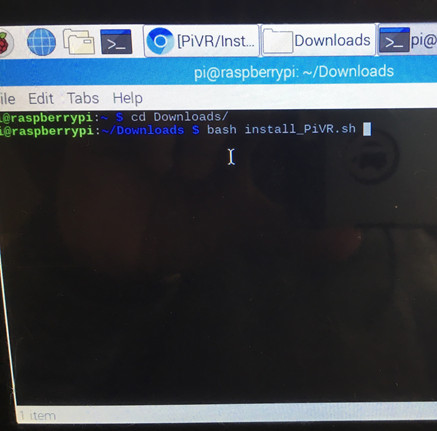

Supplement: S1 HTML — PiVR, Raspberry Pi Virtual Reality. (ZIP) [file pbio.3000712.s019.zip › S1HTLM/_images/21_install_PiVR_software.jpg]

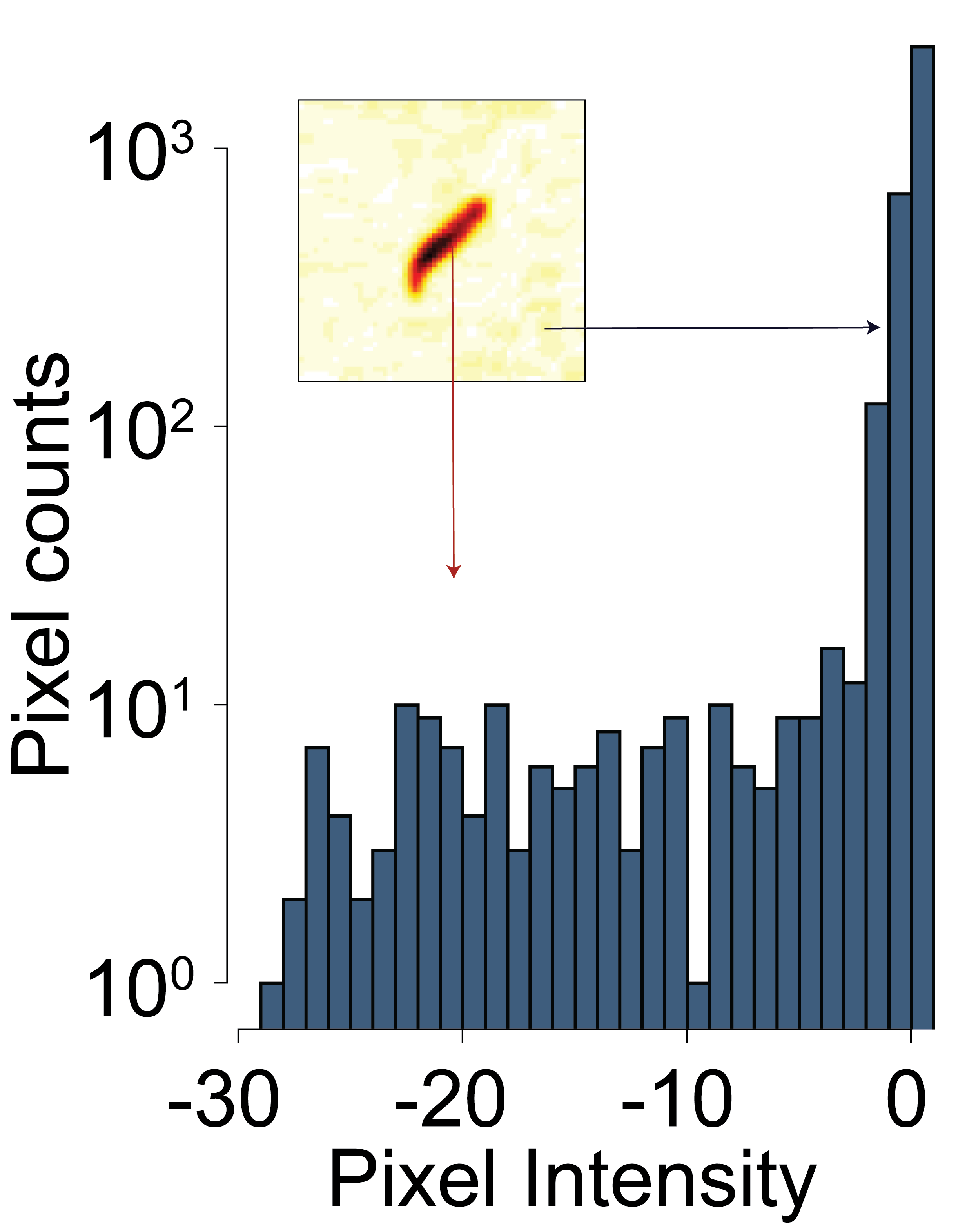

Supplement: S1 HTML — PiVR, Raspberry Pi Virtual Reality. (ZIP) [file pbio.3000712.s019.zip › S1HTLM/_images/FigS4_Histogram.png]

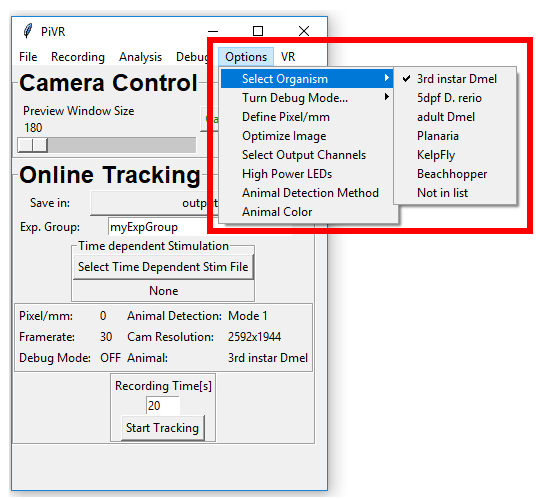

Supplement: S1 HTML — PiVR, Raspberry Pi Virtual Reality. (ZIP) [file pbio.3000712.s019.zip › S1HTLM/_images/OptionsSelectAnimal.png]

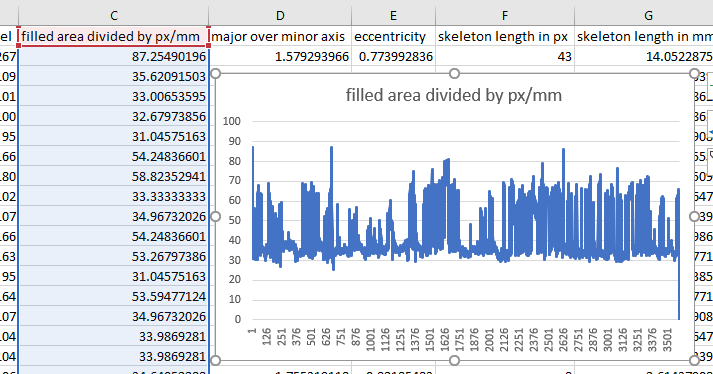

Supplement: S1 HTML — PiVR, Raspberry Pi Virtual Reality. (ZIP) [file pbio.3000712.s019.zip › S1HTLM/_images/6_heuristic_plot_example.png]

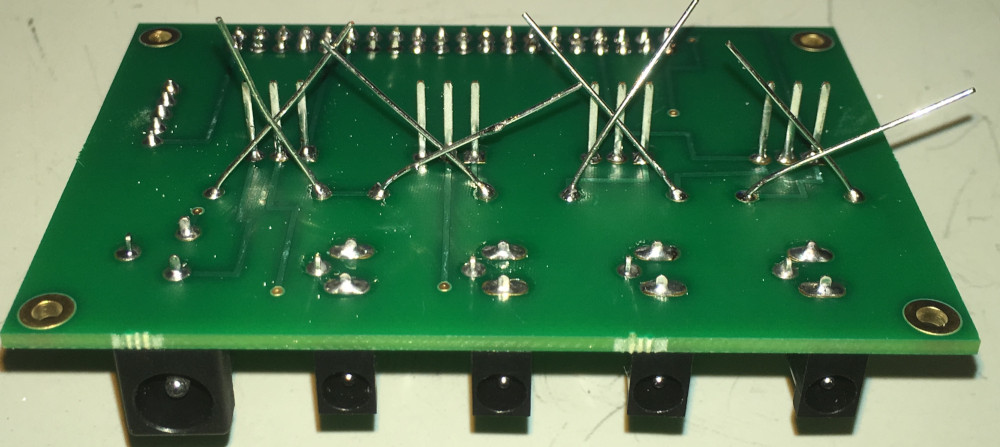

Supplement: S1 HTML — PiVR, Raspberry Pi Virtual Reality. (ZIP) [file pbio.3000712.s019.zip › S1HTLM/_images/S_15.jpg]

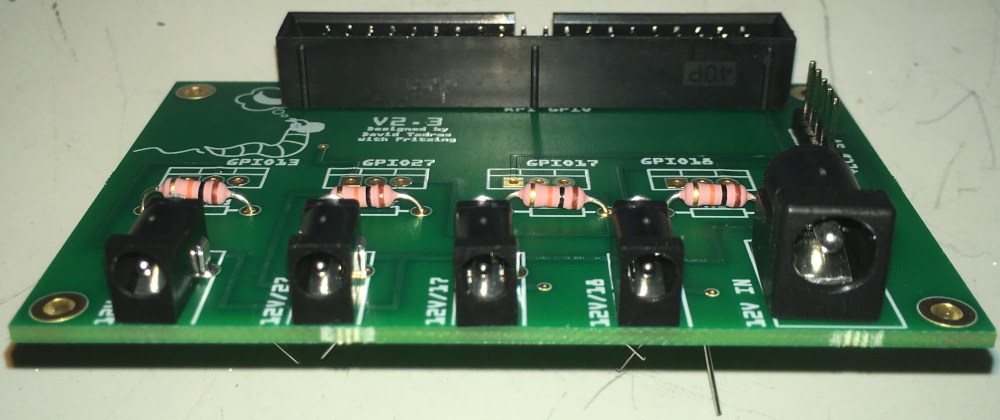

Supplement: S1 HTML — PiVR, Raspberry Pi Virtual Reality. (ZIP) [file pbio.3000712.s019.zip › S1HTLM/_images/S_11.jpg]

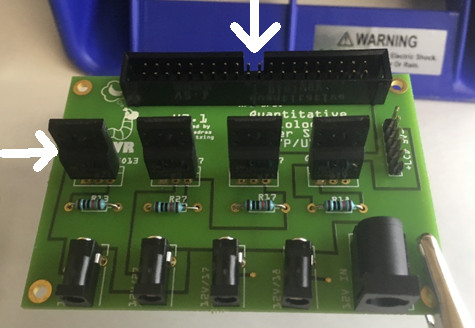

Supplement: S1 HTML — PiVR, Raspberry Pi Virtual Reality. (ZIP) [file pbio.3000712.s019.zip › S1HTLM/_images/3_solderedPCB.jpg]

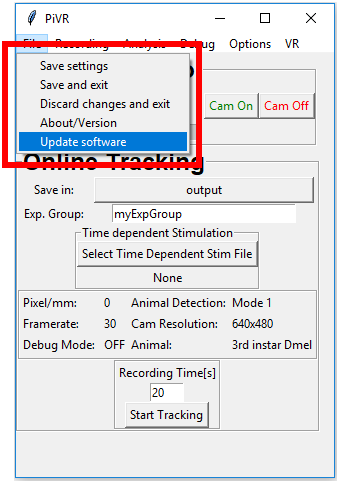

Supplement: S1 HTML — PiVR, Raspberry Pi Virtual Reality. (ZIP) [file pbio.3000712.s019.zip › S1HTLM/_images/FileUpdate.png]

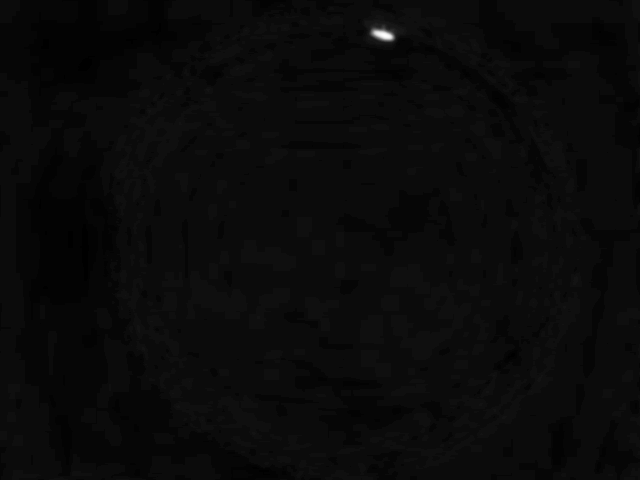

Supplement: S1 HTML — PiVR, Raspberry Pi Virtual Reality. (ZIP) [file pbio.3000712.s019.zip › S1HTLM/_images/Mode2SubtractedImage.png]

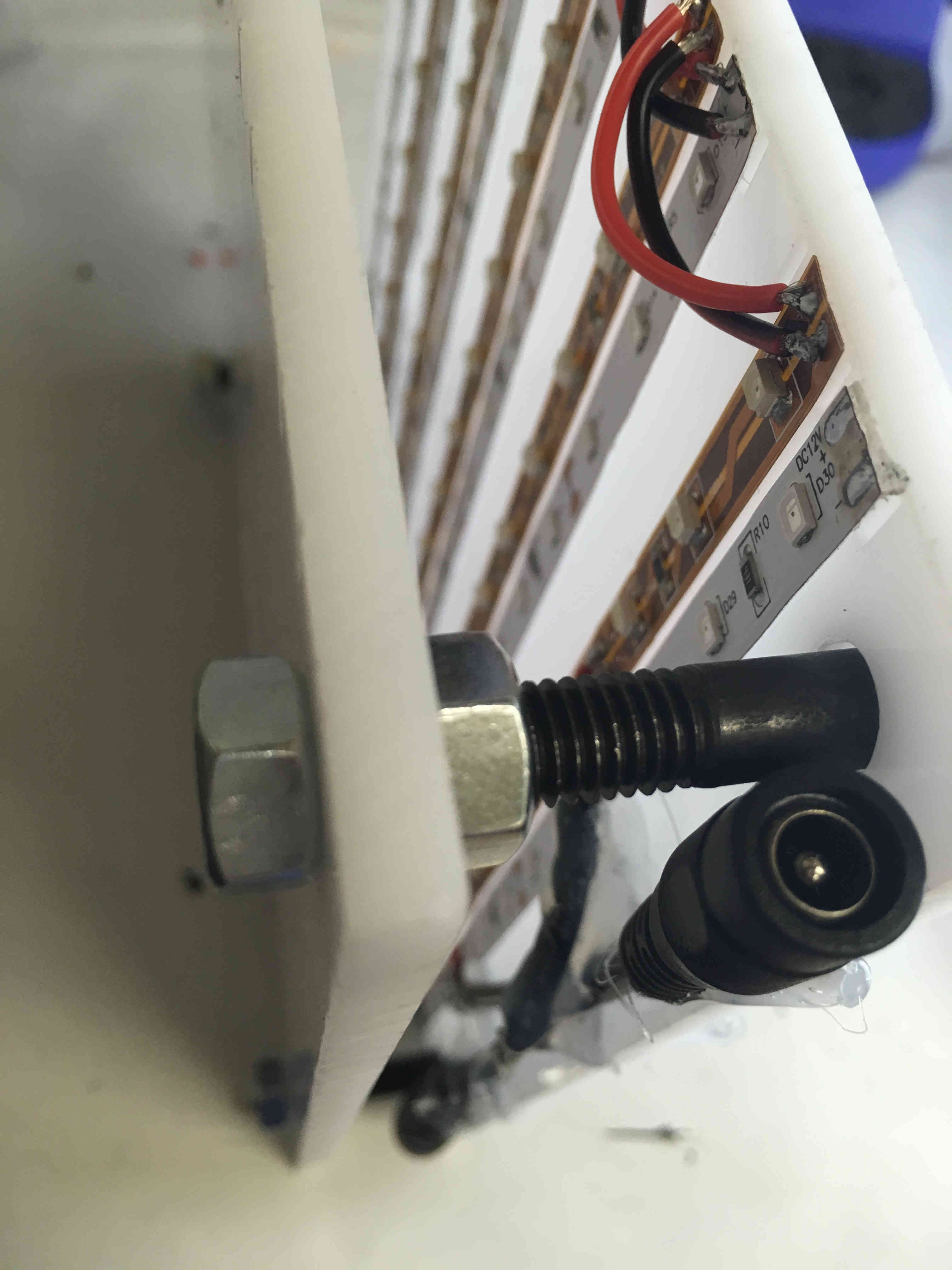

Supplement: S1 HTML — PiVR, Raspberry Pi Virtual Reality. (ZIP) [file pbio.3000712.s019.zip › S1HTLM/_images/26_second_plate_fastened.jpg]

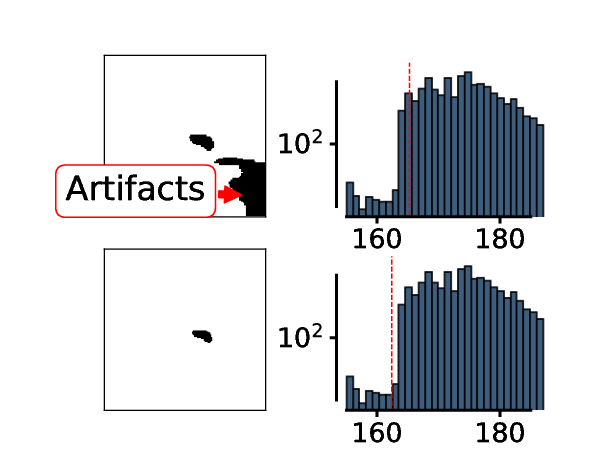

Supplement: S1 HTML — PiVR, Raspberry Pi Virtual Reality. (ZIP) [file pbio.3000712.s019.zip › S1HTLM/_images/Mode3MovingTheSTD.png]

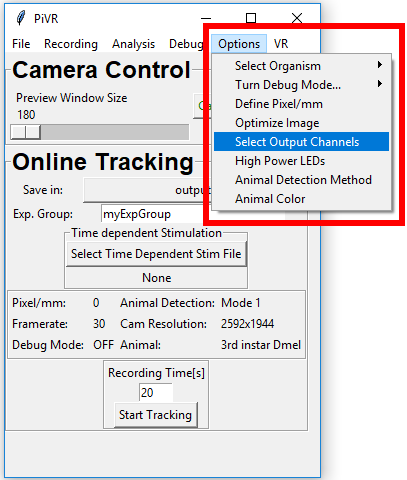

Supplement: S1 HTML — PiVR, Raspberry Pi Virtual Reality. (ZIP) [file pbio.3000712.s019.zip › S1HTLM/_images/OptionsDefineOutputChannels.png]

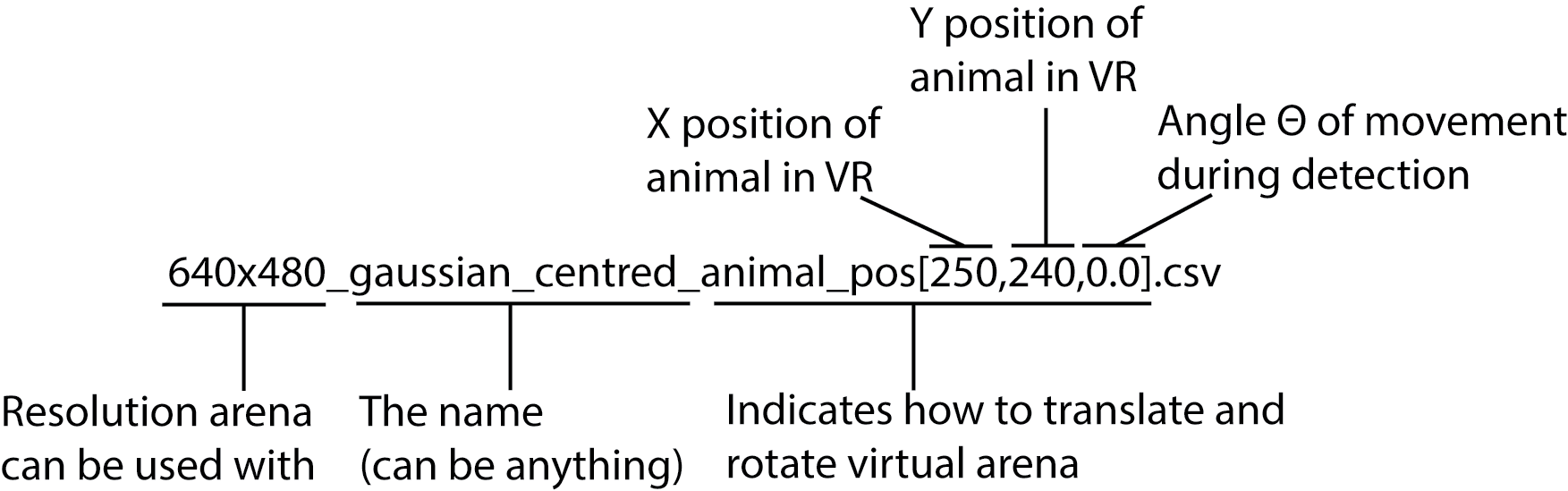

Supplement: S1 HTML — PiVR, Raspberry Pi Virtual Reality. (ZIP) [file pbio.3000712.s019.zip › S1HTLM/_images/7_explain_names.png]

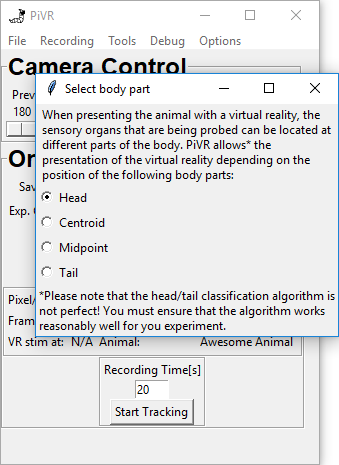

Supplement: S1 HTML — PiVR, Raspberry Pi Virtual Reality. (ZIP) [file pbio.3000712.s019.zip › S1HTLM/_images/VRStimulationPoint_Menu.png]

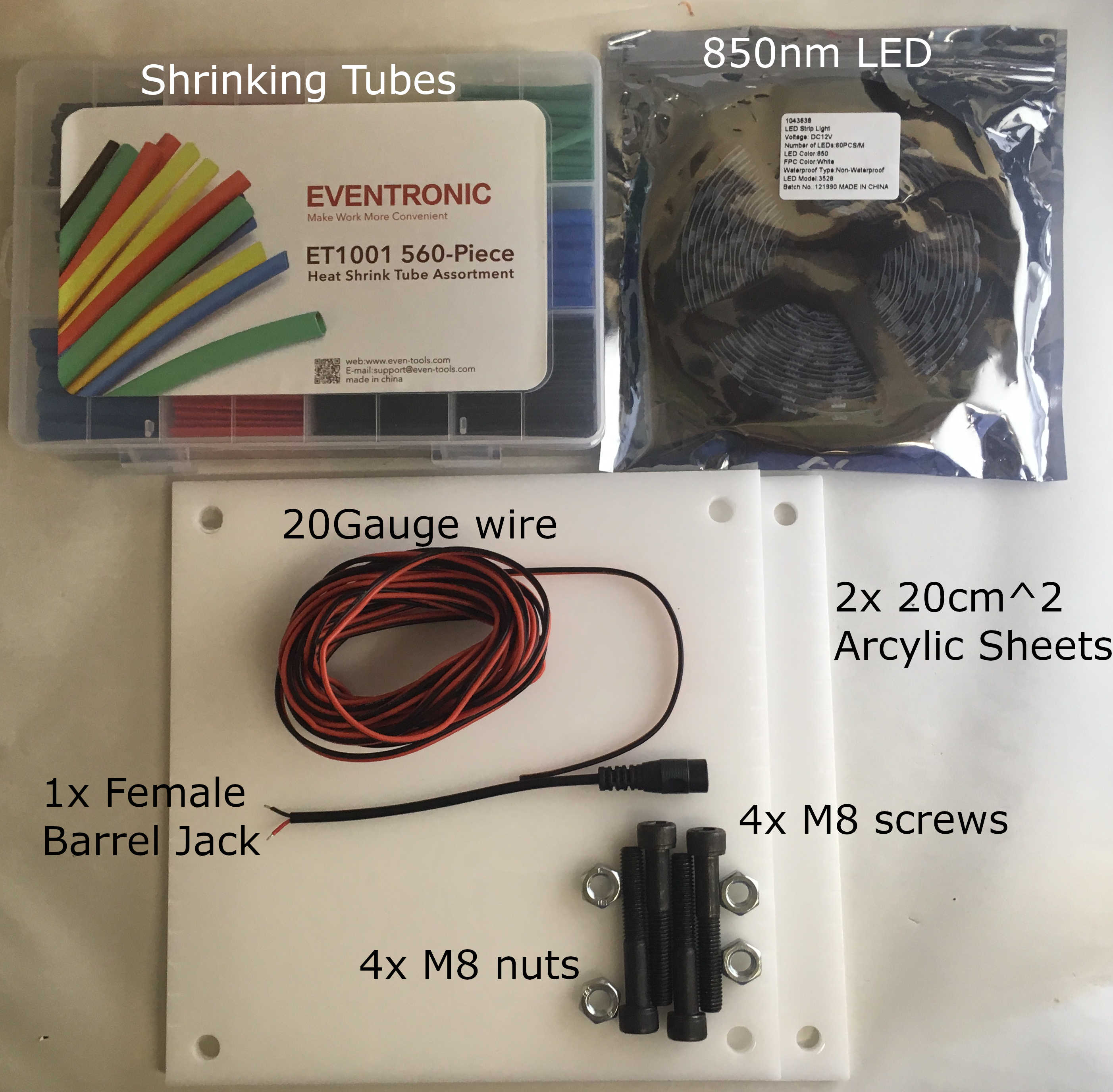

Supplement: S1 HTML — PiVR, Raspberry Pi Virtual Reality. (ZIP) [file pbio.3000712.s019.zip › S1HTLM/_images/22_Arena_overview.jpg]

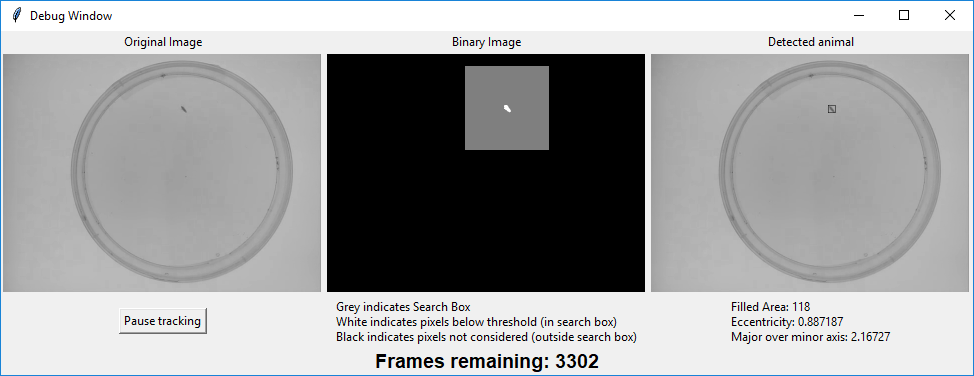

Supplement: S1 HTML — PiVR, Raspberry Pi Virtual Reality. (ZIP) [file pbio.3000712.s019.zip › S1HTLM/_images/3_debug_tracking.png]

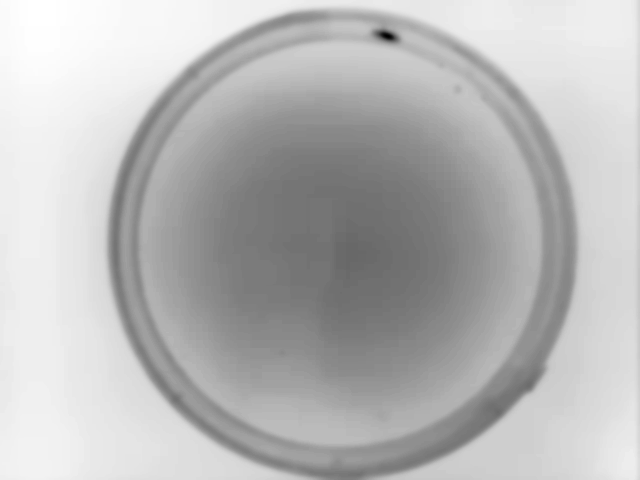

Supplement: S1 HTML — PiVR, Raspberry Pi Virtual Reality. (ZIP) [file pbio.3000712.s019.zip › S1HTLM/_images/SecondMeanImage.png]

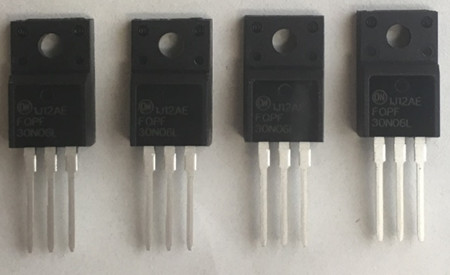

Supplement: S1 HTML — PiVR, Raspberry Pi Virtual Reality. (ZIP) [file pbio.3000712.s019.zip › S1HTLM/_images/2_1_transistors.jpg]

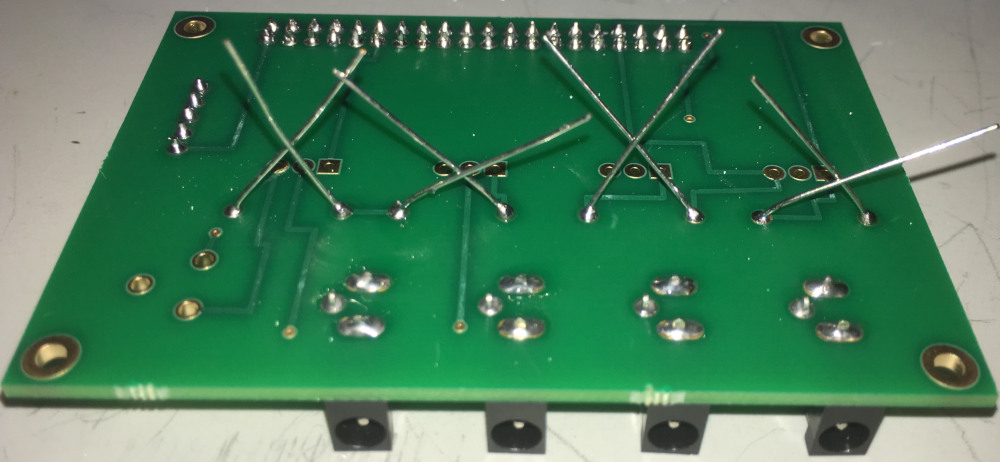

Supplement: S1 HTML — PiVR, Raspberry Pi Virtual Reality. (ZIP) [file pbio.3000712.s019.zip › S1HTLM/_images/S_10.jpg]

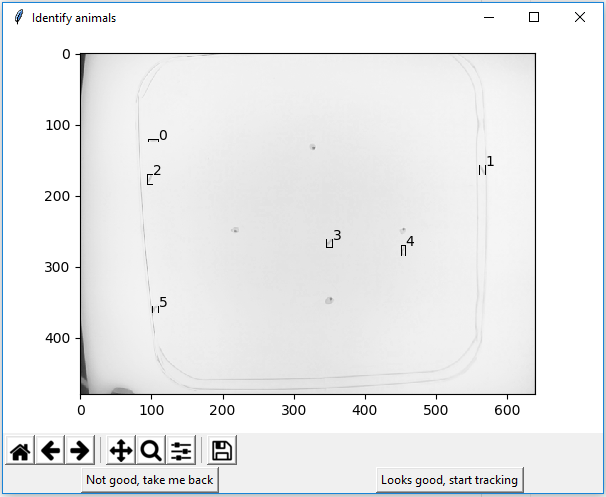

Supplement: S1 HTML — PiVR, Raspberry Pi Virtual Reality. (ZIP) [file pbio.3000712.s019.zip › S1HTLM/_images/6_7_MultiAnimalTrackerStartTracking.png]

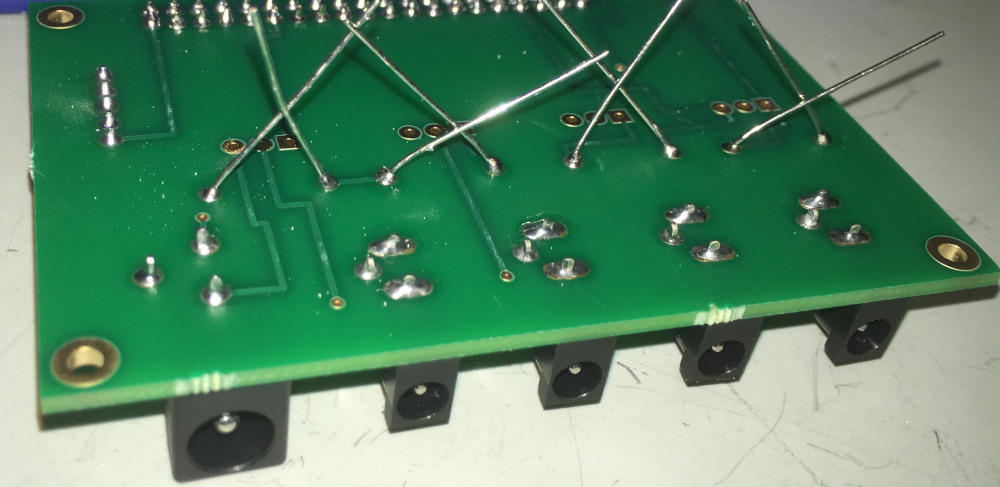

Supplement: S1 HTML — PiVR, Raspberry Pi Virtual Reality. (ZIP) [file pbio.3000712.s019.zip › S1HTLM/_images/S_12.jpg]

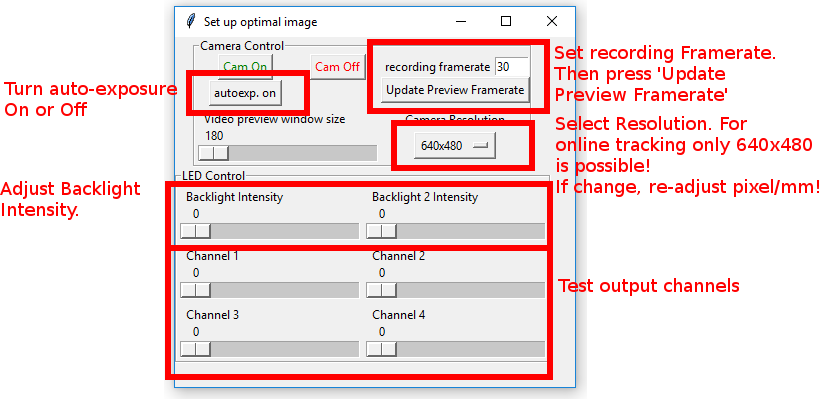

Supplement: S1 HTML — PiVR, Raspberry Pi Virtual Reality. (ZIP) [file pbio.3000712.s019.zip › S1HTLM/_images/OptimizeImageOverview.png]

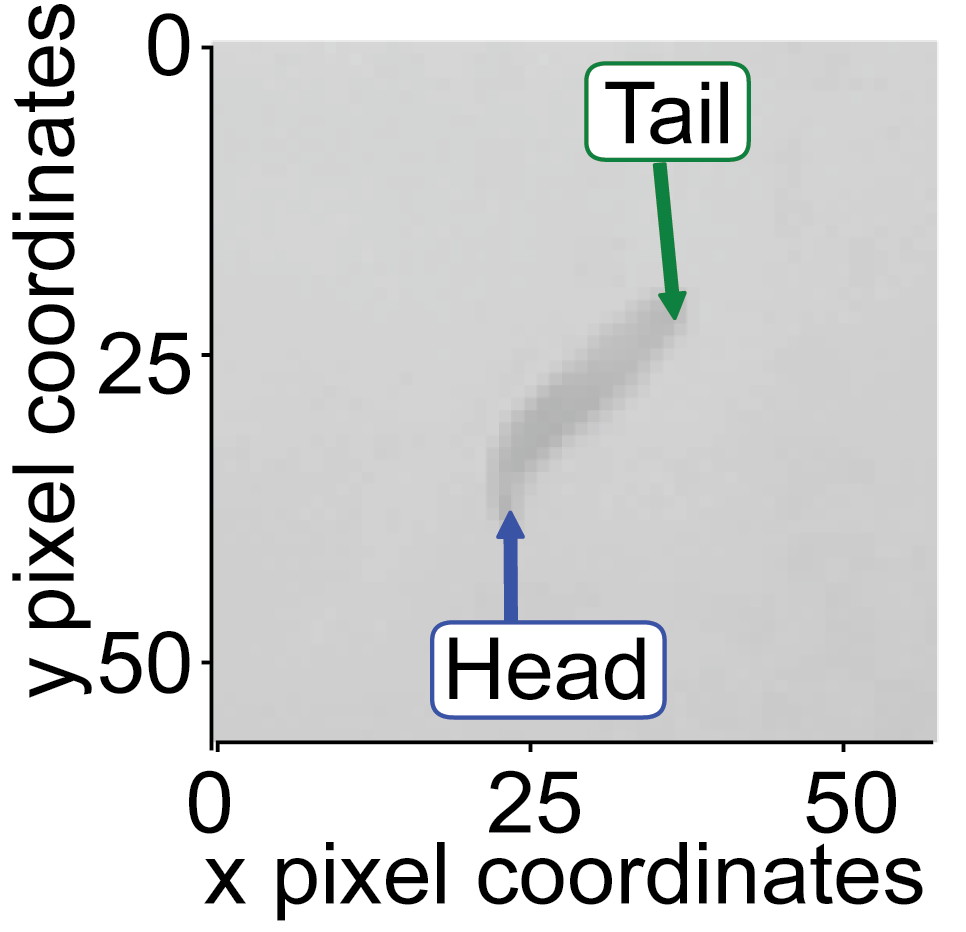

Supplement: S1 HTML — PiVR, Raspberry Pi Virtual Reality. (ZIP) [file pbio.3000712.s019.zip › S1HTLM/_images/FigS5_TailClassified.png]

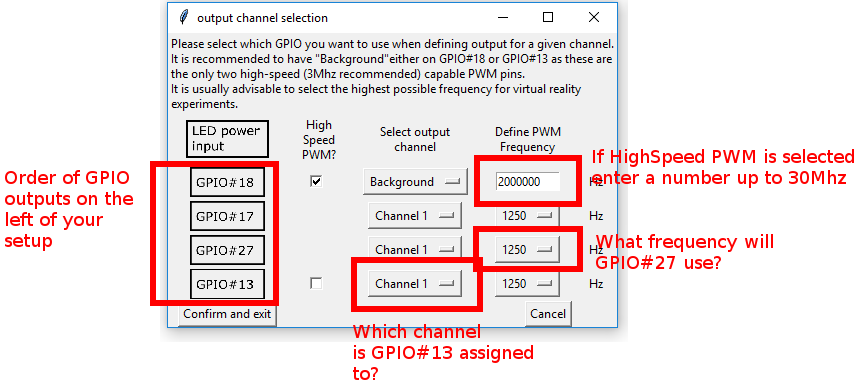

Supplement: S1 HTML — PiVR, Raspberry Pi Virtual Reality. (ZIP) [file pbio.3000712.s019.zip › S1HTLM/_images/outputChannelSelection.png]

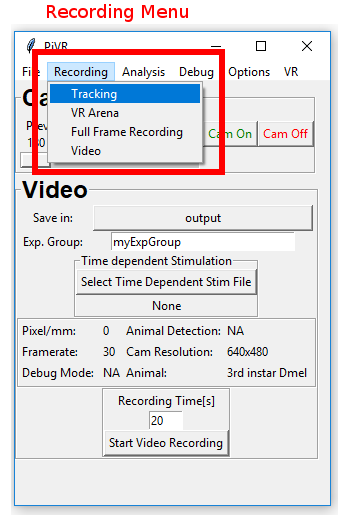

Supplement: S1 HTML — PiVR, Raspberry Pi Virtual Reality. (ZIP) [file pbio.3000712.s019.zip › S1HTLM/_images/RecordingMenu.png]

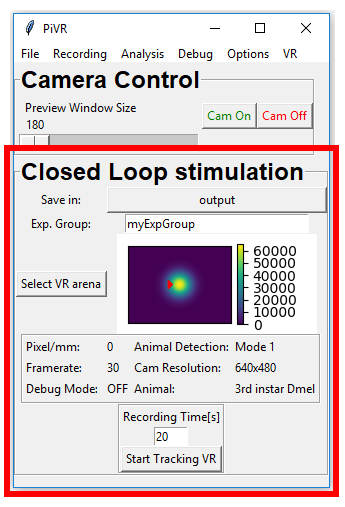

Supplement: S1 HTML — PiVR, Raspberry Pi Virtual Reality. (ZIP) [file pbio.3000712.s019.zip › S1HTLM/_images/ExperimentControlFrameVRArena.png]

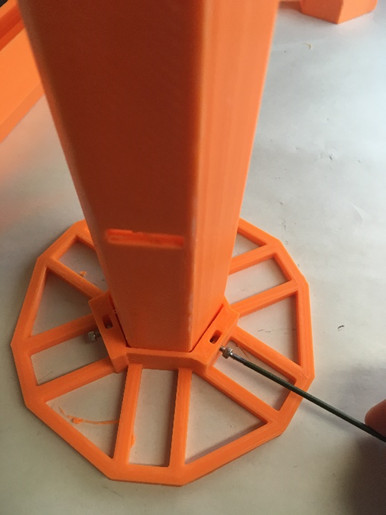

Supplement: S1 HTML — PiVR, Raspberry Pi Virtual Reality. (ZIP) [file pbio.3000712.s019.zip › S1HTLM/_images/14_TowerPedestal_attached.jpg]

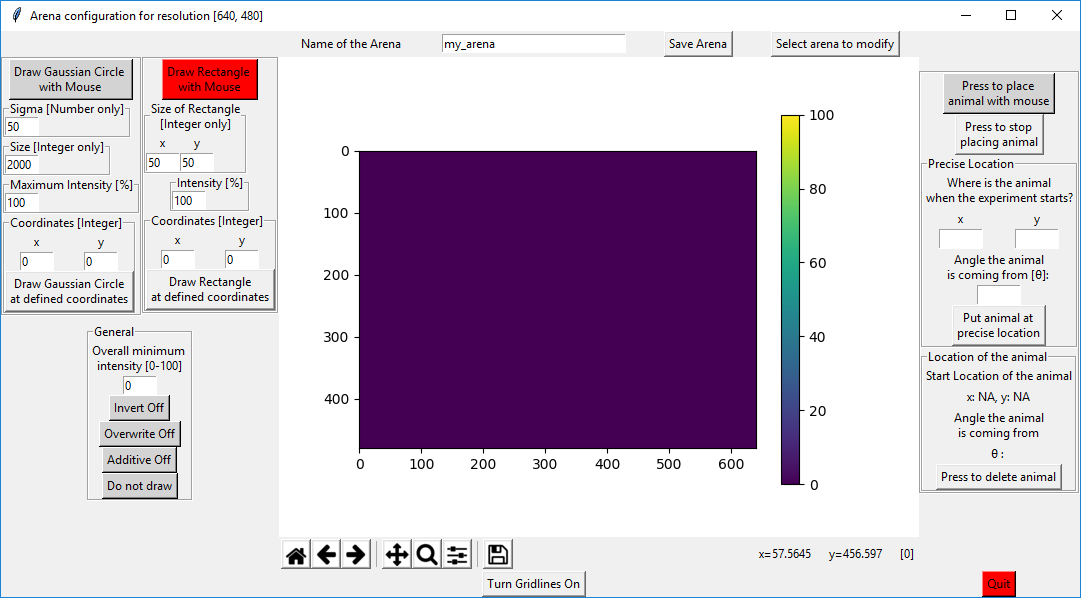

Supplement: S1 HTML — PiVR, Raspberry Pi Virtual Reality. (ZIP) [file pbio.3000712.s019.zip › S1HTLM/_images/9_DrawVRArena_overview.png]

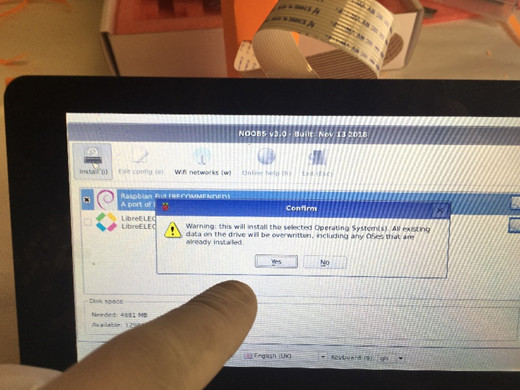

Supplement: S1 HTML — PiVR, Raspberry Pi Virtual Reality. (ZIP) [file pbio.3000712.s019.zip › S1HTLM/_images/20_Install_Raspian.jpg]

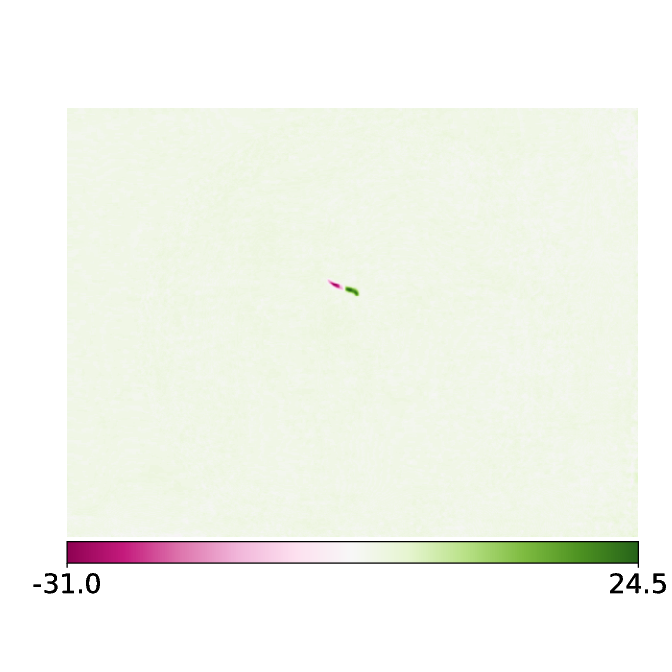

Supplement: S1 HTML — PiVR, Raspberry Pi Virtual Reality. (ZIP) [file pbio.3000712.s019.zip › S1HTLM/_images/Mode3SecondSubtractedImage.png]

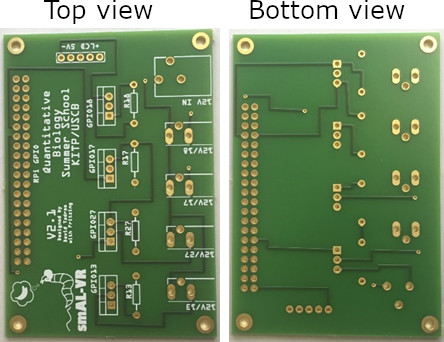

Supplement: S1 HTML — PiVR, Raspberry Pi Virtual Reality. (ZIP) [file pbio.3000712.s019.zip › S1HTLM/_images/2_0_PCB.jpg]

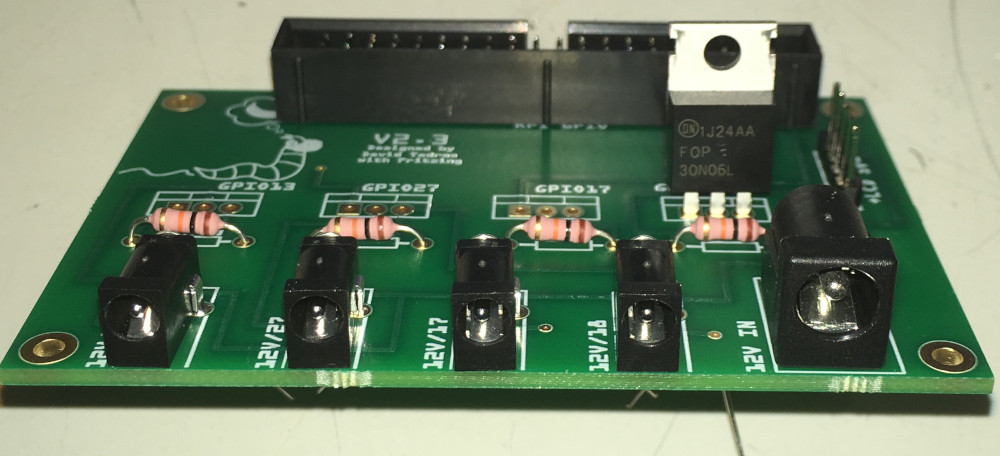

Supplement: S1 HTML — PiVR, Raspberry Pi Virtual Reality. (ZIP) [file pbio.3000712.s019.zip › S1HTLM/_images/S_13.jpg]

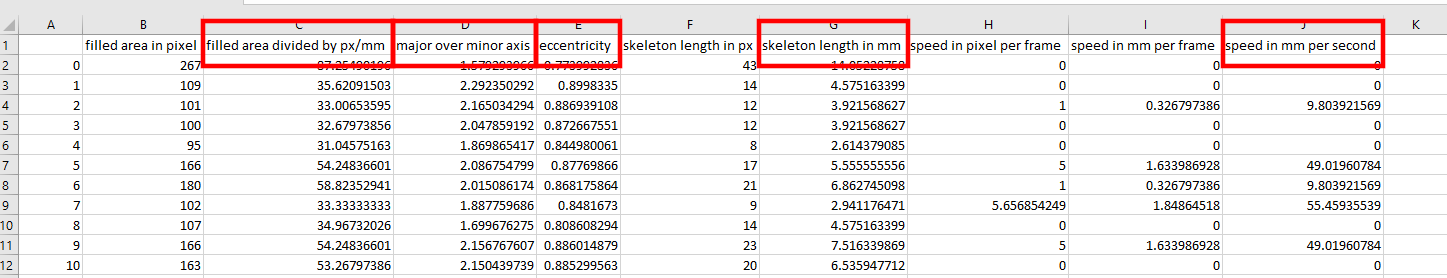

Supplement: S1 HTML — PiVR, Raspberry Pi Virtual Reality. (ZIP) [file pbio.3000712.s019.zip › S1HTLM/_images/5_heuristics_columns.png]

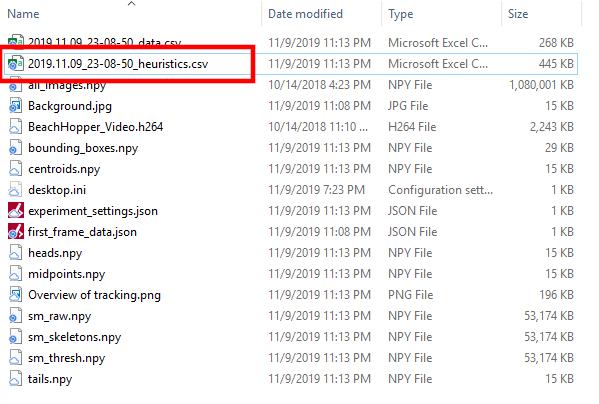

Supplement: S1 HTML — PiVR, Raspberry Pi Virtual Reality. (ZIP) [file pbio.3000712.s019.zip › S1HTLM/_images/4_heuristics.png]

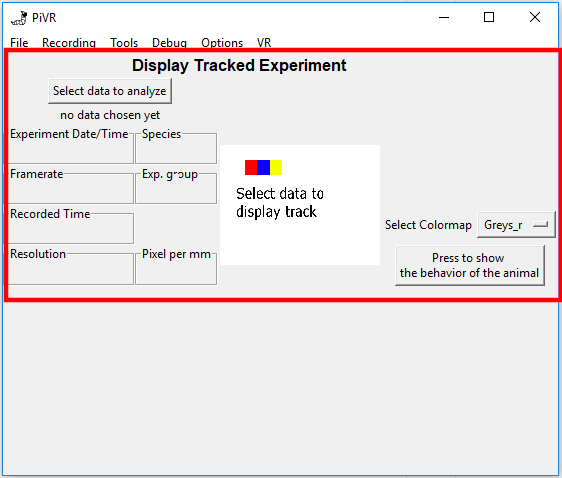

Supplement: S1 HTML — PiVR, Raspberry Pi Virtual Reality. (ZIP) [file pbio.3000712.s019.zip › S1HTLM/_images/5_DisplayTrackedExperiment.png]

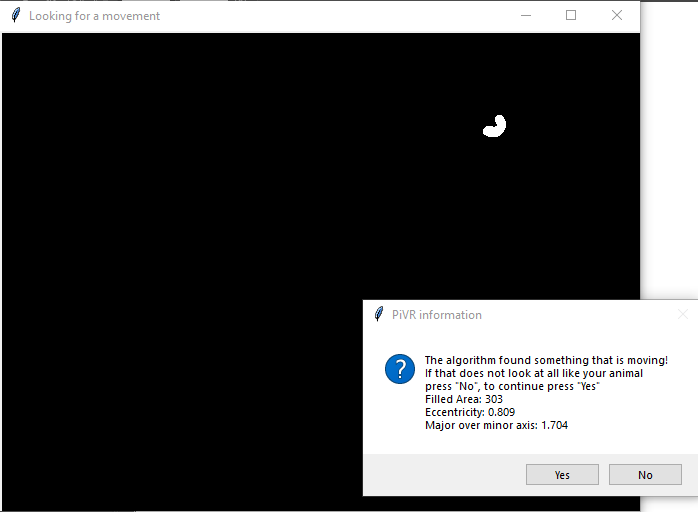

Supplement: S1 HTML — PiVR, Raspberry Pi Virtual Reality. (ZIP) [file pbio.3000712.s019.zip › S1HTLM/_images/1_debug_detection1.png]

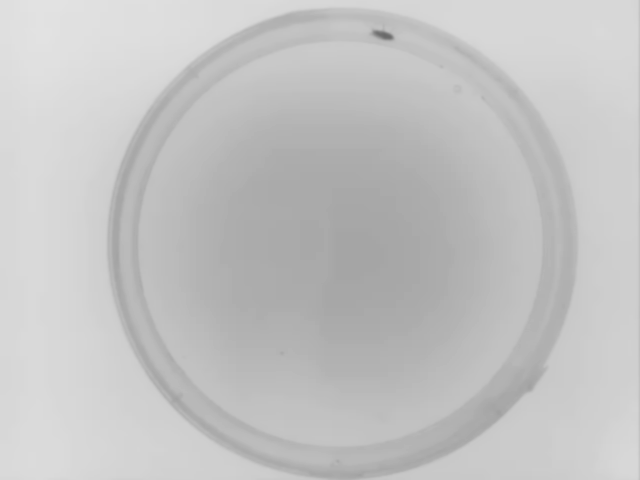

Supplement: S1 HTML — PiVR, Raspberry Pi Virtual Reality. (ZIP) [file pbio.3000712.s019.zip › S1HTLM/_images/Mode2FirstImage.png]

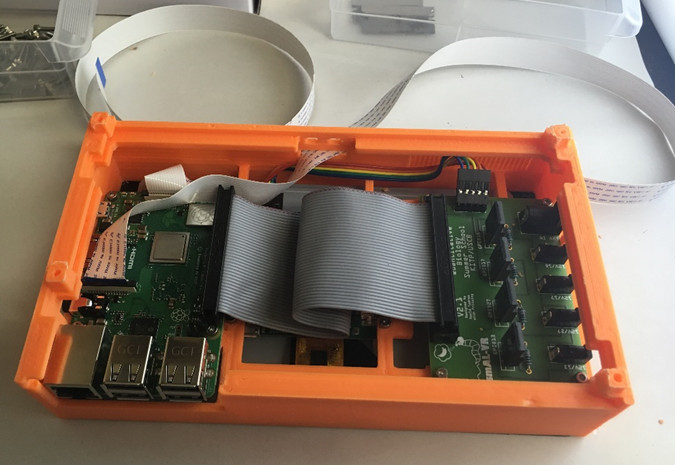

Supplement: S1 HTML — PiVR, Raspberry Pi Virtual Reality. (ZIP) [file pbio.3000712.s019.zip › S1HTLM/_images/12_Casing_stuffed.jpg]

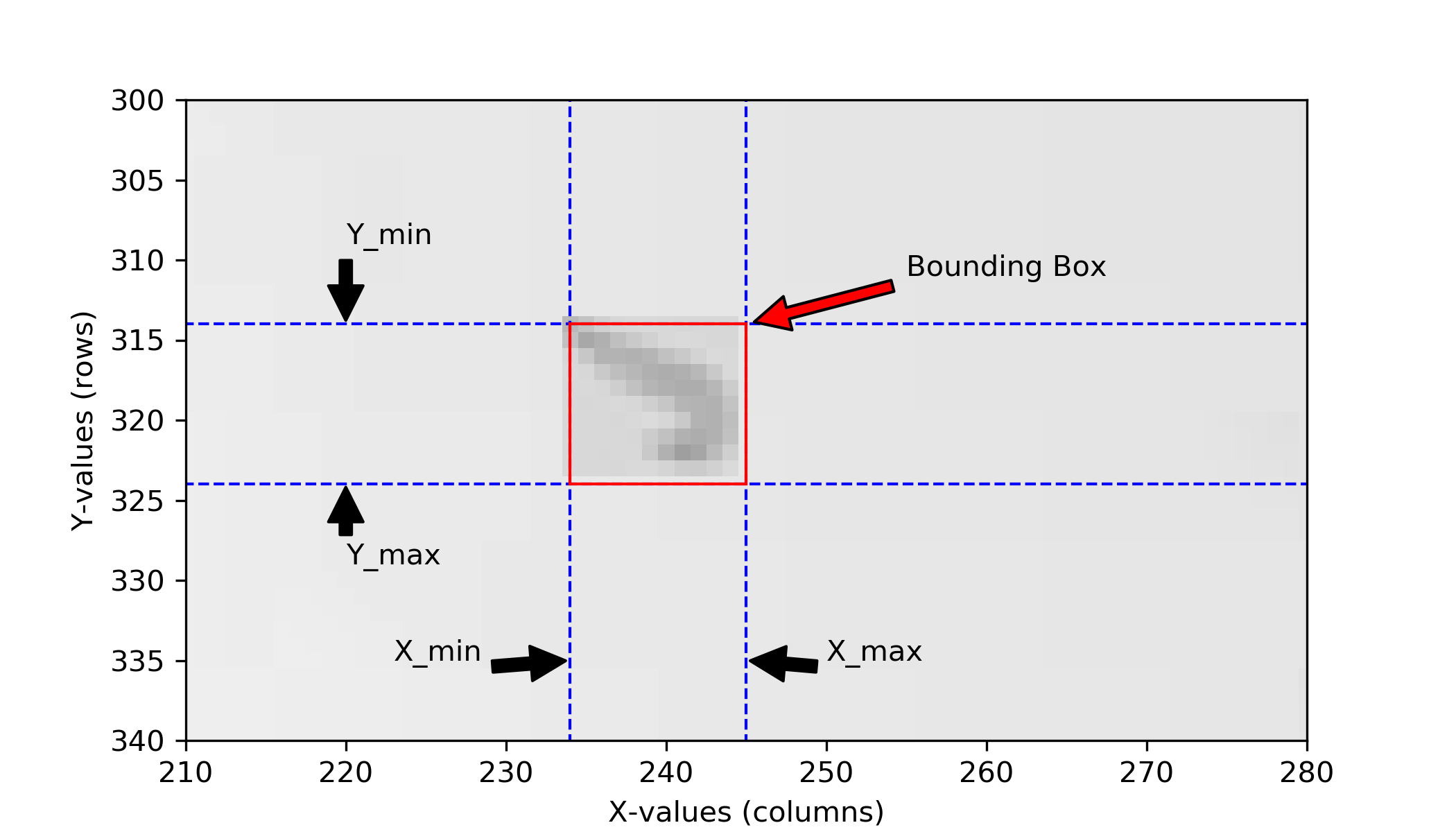

Supplement: S1 HTML — PiVR, Raspberry Pi Virtual Reality. (ZIP) [file pbio.3000712.s019.zip › S1HTLM/_images/bbox_illustration.png]

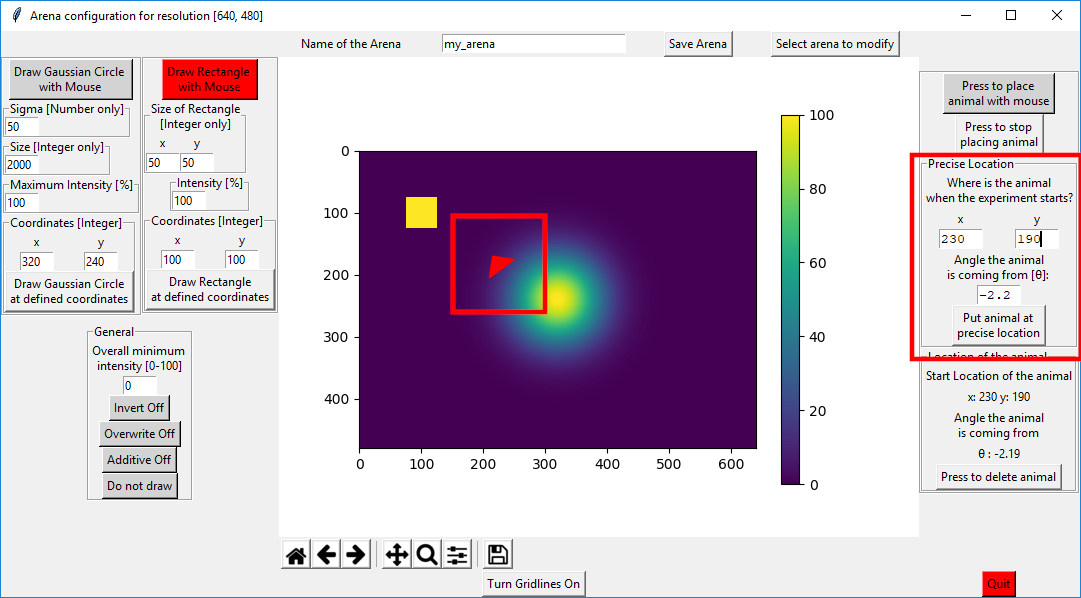

Supplement: S1 HTML — PiVR, Raspberry Pi Virtual Reality. (ZIP) [file pbio.3000712.s019.zip › S1HTLM/_images/9_2_DrawVRArena_example_2.png]

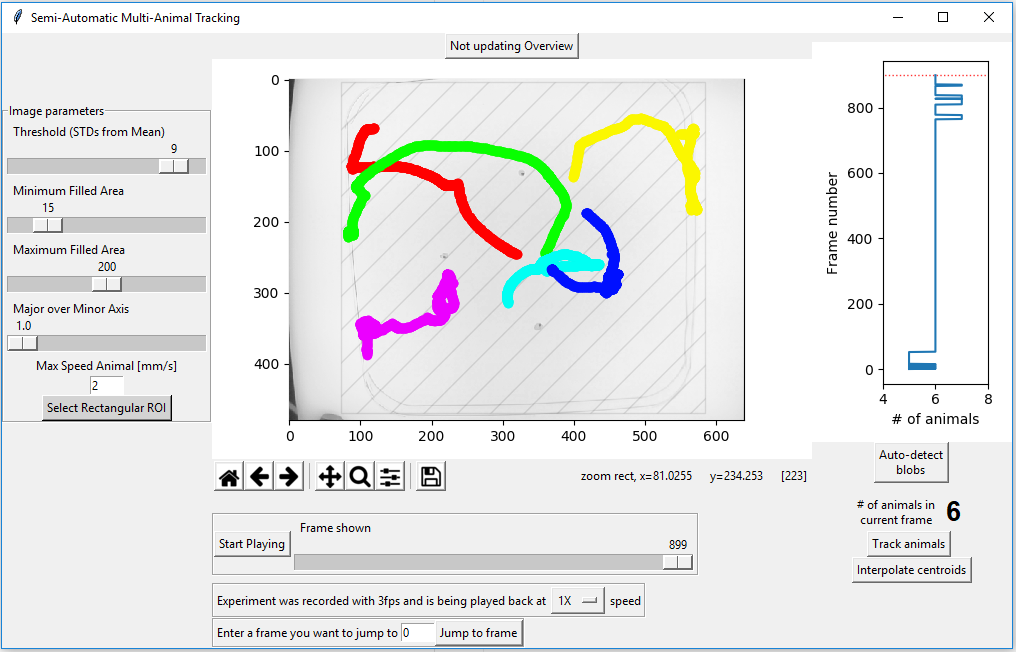

Supplement: S1 HTML — PiVR, Raspberry Pi Virtual Reality. (ZIP) [file pbio.3000712.s019.zip › S1HTLM/_images/6_8_MultiAnimalTrackerTrackingResult.png]

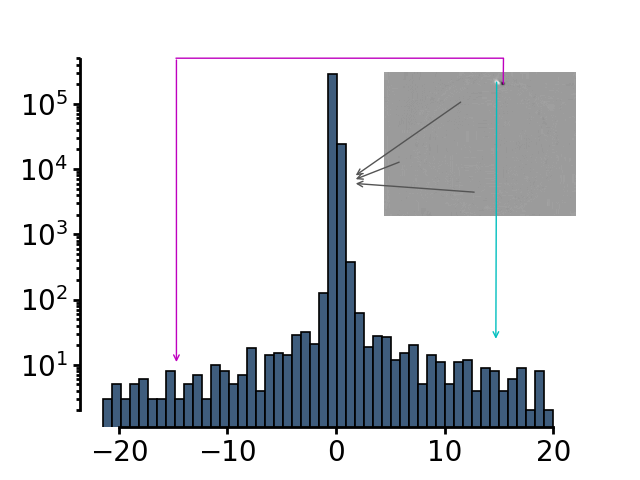

Supplement: S1 HTML — PiVR, Raspberry Pi Virtual Reality. (ZIP) [file pbio.3000712.s019.zip › S1HTLM/_images/HistogramMode1.png]

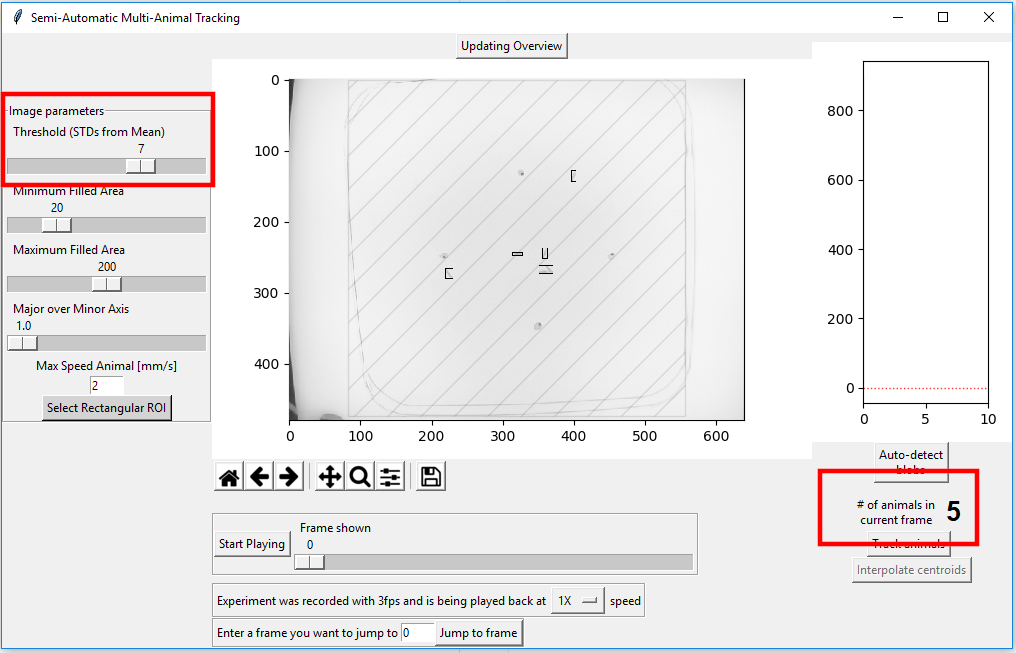

Supplement: S1 HTML — PiVR, Raspberry Pi Virtual Reality. (ZIP) [file pbio.3000712.s019.zip › S1HTLM/_images/6_3_MultiAnimalTrackerSelectTreshold.png]

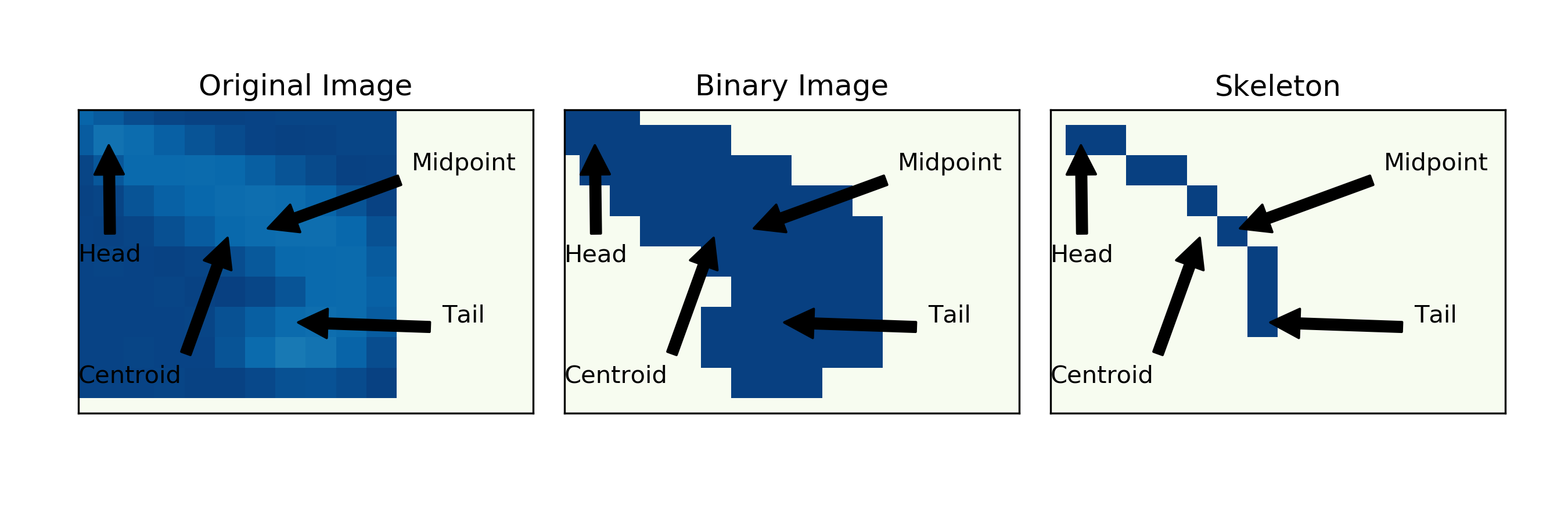

Supplement: S1 HTML — PiVR, Raspberry Pi Virtual Reality. (ZIP) [file pbio.3000712.s019.zip › S1HTLM/_images/CM_illustration.png]

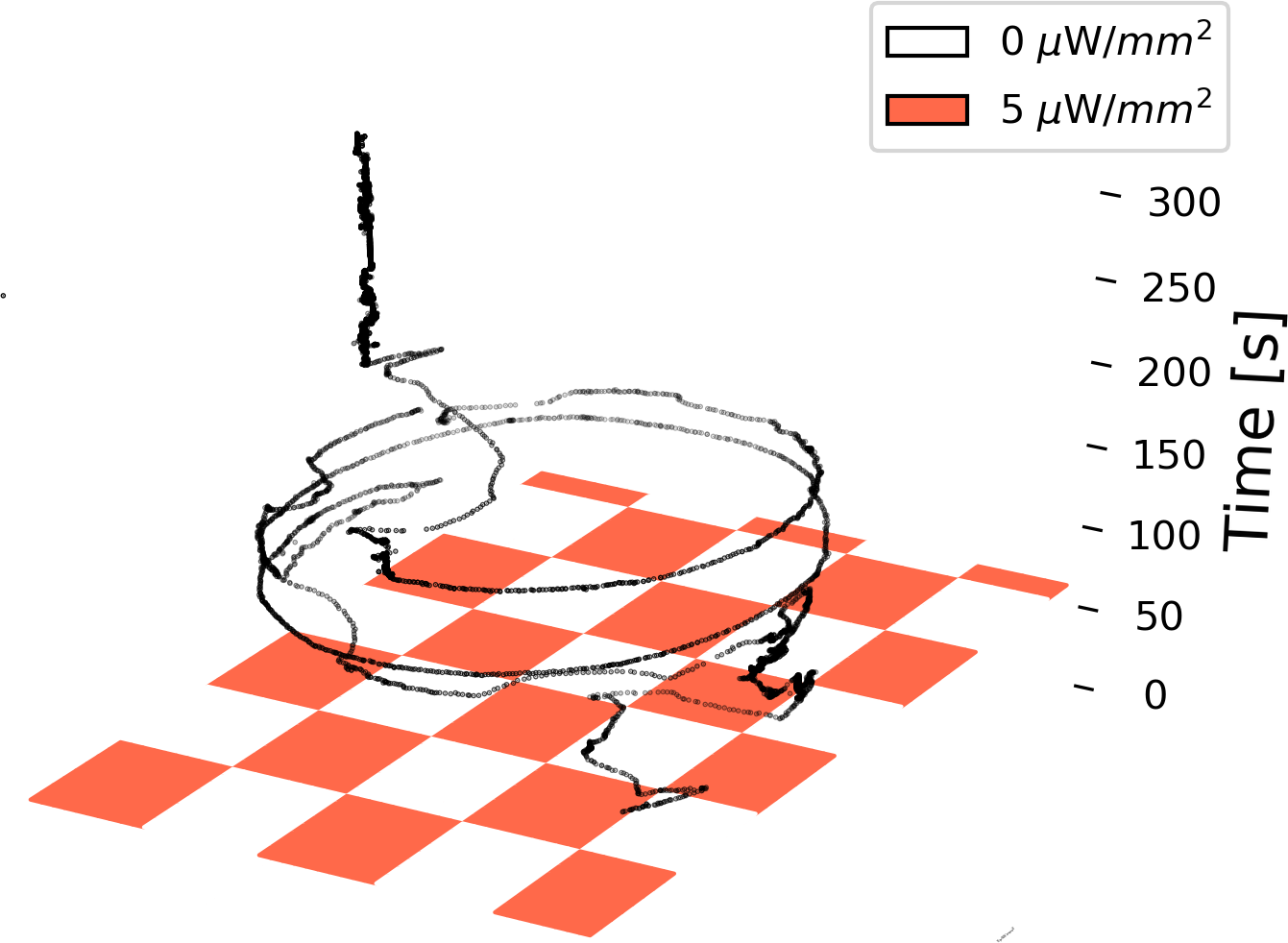

Supplement: S1 HTML — PiVR, Raspberry Pi Virtual Reality. (ZIP) [file pbio.3000712.s019.zip › S1HTLM/_images/fly_trajectory.png]

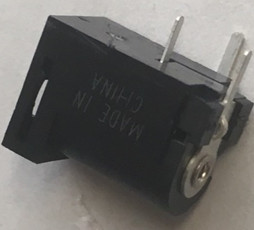

Supplement: S1 HTML — PiVR, Raspberry Pi Virtual Reality. (ZIP) [file pbio.3000712.s019.zip › S1HTLM/_images/2_3_Jack5_5.jpg]

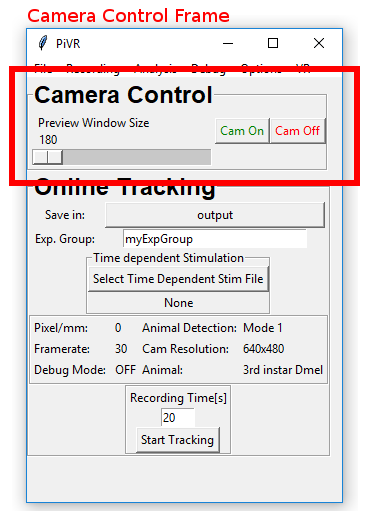

Supplement: S1 HTML — PiVR, Raspberry Pi Virtual Reality. (ZIP) [file pbio.3000712.s019.zip › S1HTLM/_images/CameraControlFrame.png]

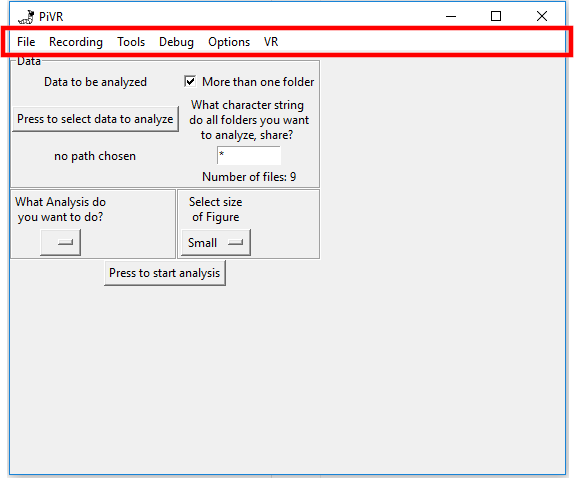

Supplement: S1 HTML — PiVR, Raspberry Pi Virtual Reality. (ZIP) [file pbio.3000712.s019.zip › S1HTLM/_images/1_menubar.png]

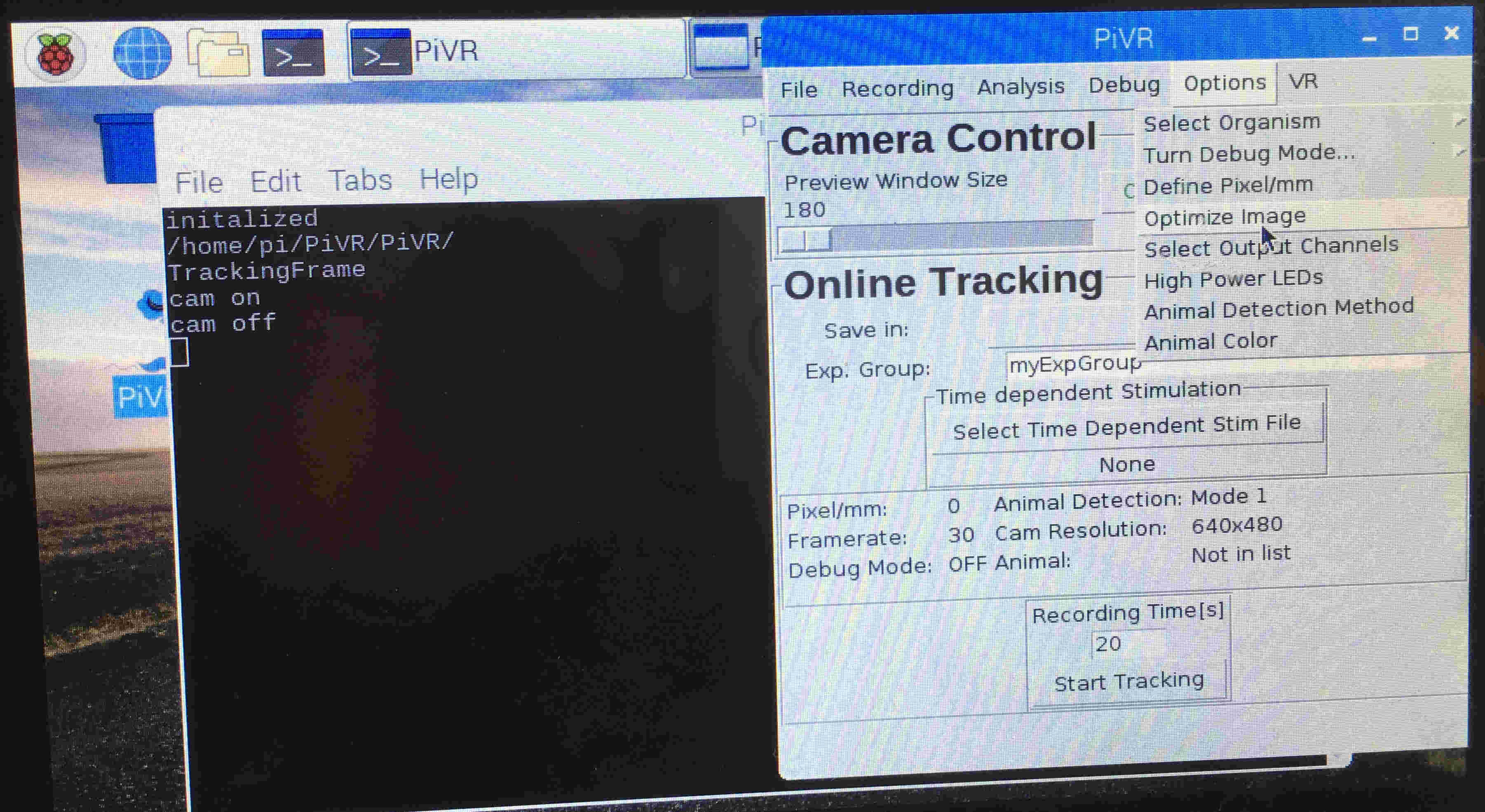

Supplement: S1 HTML — PiVR, Raspberry Pi Virtual Reality. (ZIP) [file pbio.3000712.s019.zip › S1HTLM/_images/29_Starting_Software.jpg]

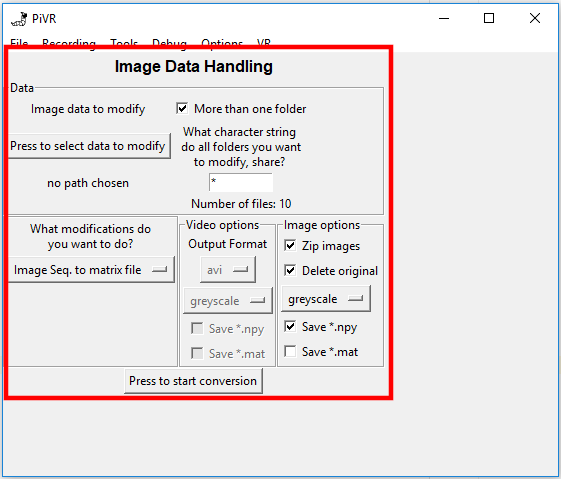

Supplement: S1 HTML — PiVR, Raspberry Pi Virtual Reality. (ZIP) [file pbio.3000712.s019.zip › S1HTLM/_images/4_ImageDataHandlingFrame.png]

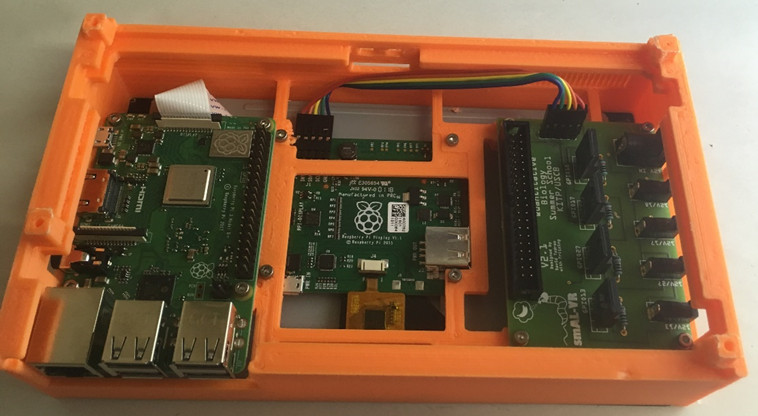

Supplement: S1 HTML — PiVR, Raspberry Pi Virtual Reality. (ZIP) [file pbio.3000712.s019.zip › S1HTLM/_images/10_PCB_RPi_on_casing.jpg]

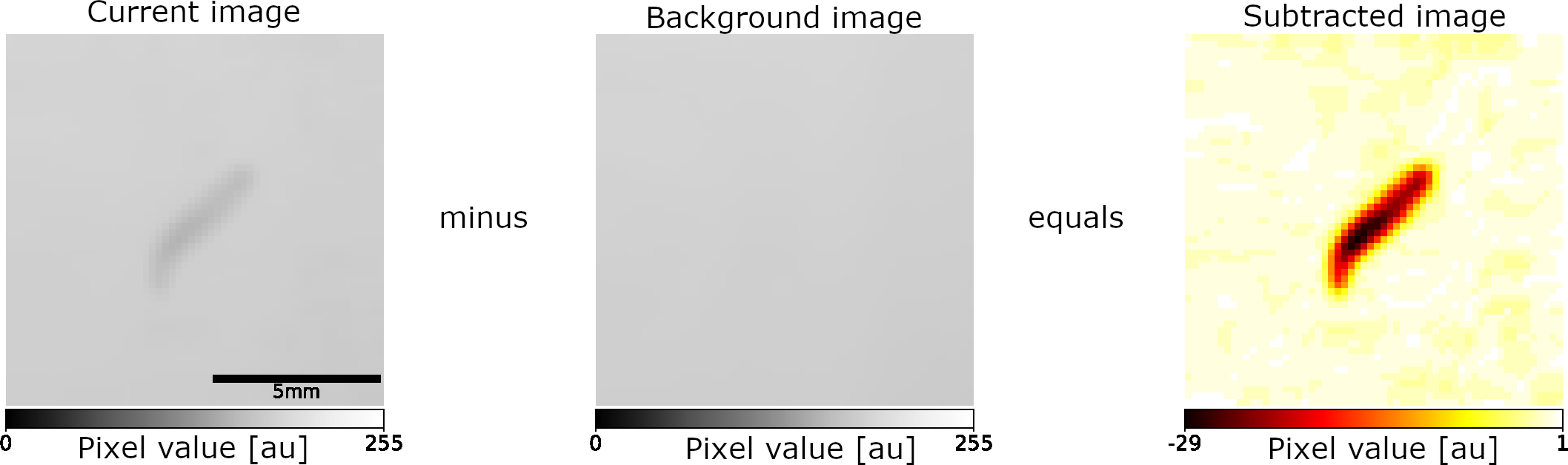

Supplement: S1 HTML — PiVR, Raspberry Pi Virtual Reality. (ZIP) [file pbio.3000712.s019.zip › S1HTLM/_images/FigS4Subtract.png]

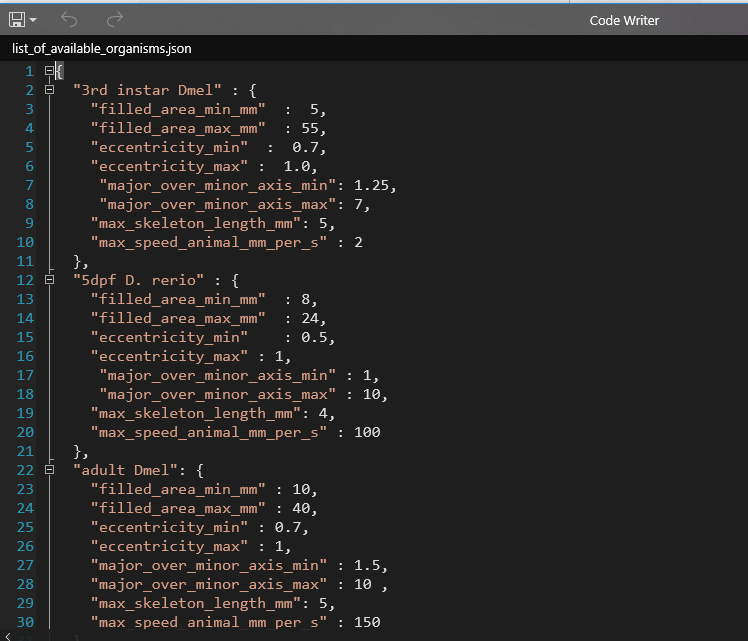

Supplement: S1 HTML — PiVR, Raspberry Pi Virtual Reality. (ZIP) [file pbio.3000712.s019.zip › S1HTLM/_images/8_open_organism_json.png]

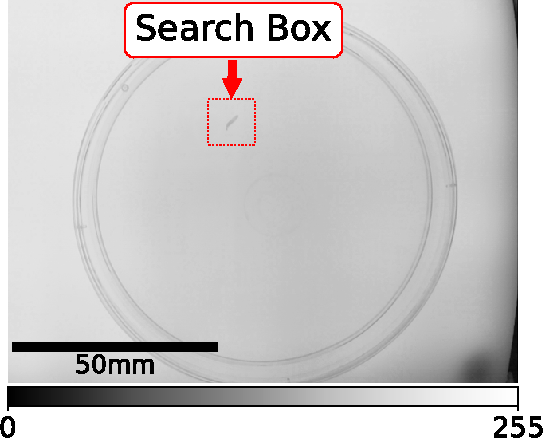

Supplement: S1 HTML — PiVR, Raspberry Pi Virtual Reality. (ZIP) [file pbio.3000712.s019.zip › S1HTLM/_images/FigS4a_FlowchartIndicateSearchBox.png]

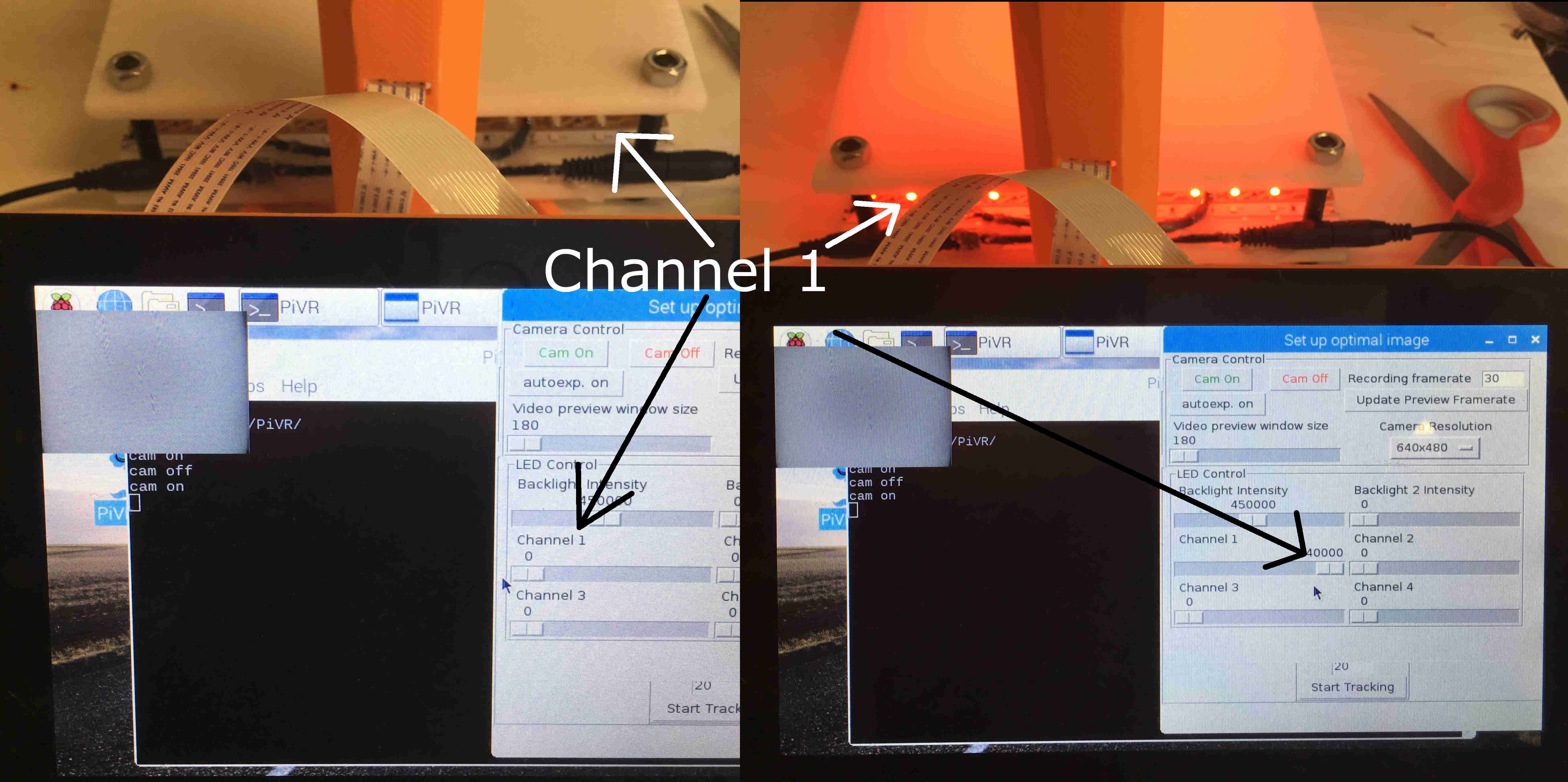

Supplement: S1 HTML — PiVR, Raspberry Pi Virtual Reality. (ZIP) [file pbio.3000712.s019.zip › S1HTLM/_images/31_1_testing_channel1.jpg]

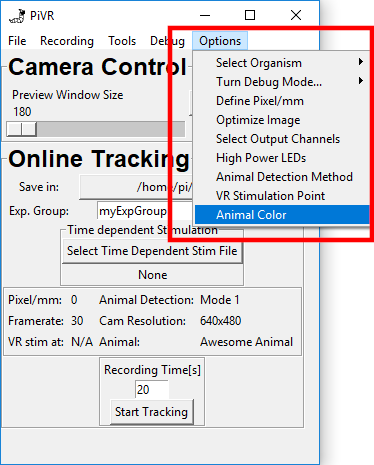

Supplement: S1 HTML — PiVR, Raspberry Pi Virtual Reality. (ZIP) [file pbio.3000712.s019.zip › S1HTLM/_images/OptionMenuAnimalColor.png]

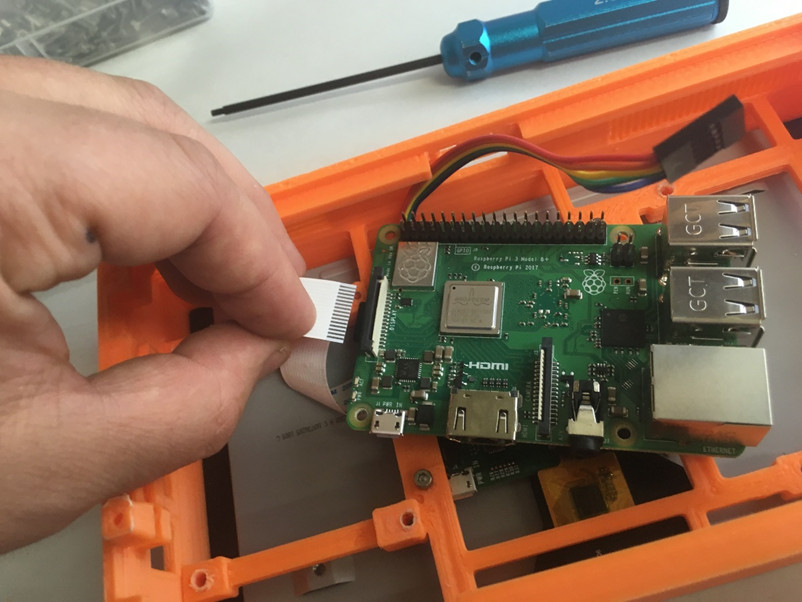

Supplement: S1 HTML — PiVR, Raspberry Pi Virtual Reality. (ZIP) [file pbio.3000712.s019.zip › S1HTLM/_images/8_0_Raspberry_monitor_cable.jpg]

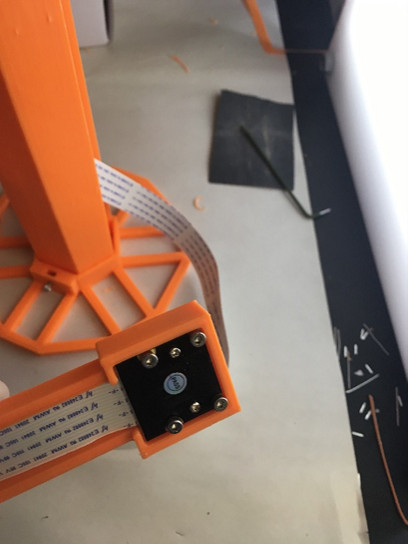

Supplement: S1 HTML — PiVR, Raspberry Pi Virtual Reality. (ZIP) [file pbio.3000712.s019.zip › S1HTLM/_images/18_Camera_attached_to_CamHolder.jpg]

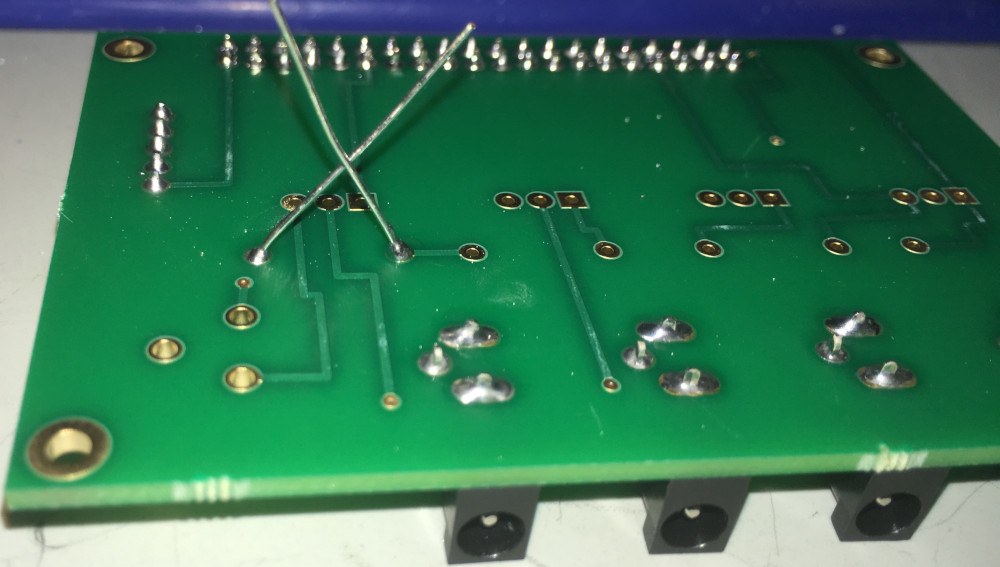

Supplement: S1 HTML — PiVR, Raspberry Pi Virtual Reality. (ZIP) [file pbio.3000712.s019.zip › S1HTLM/_images/S_9.jpg]

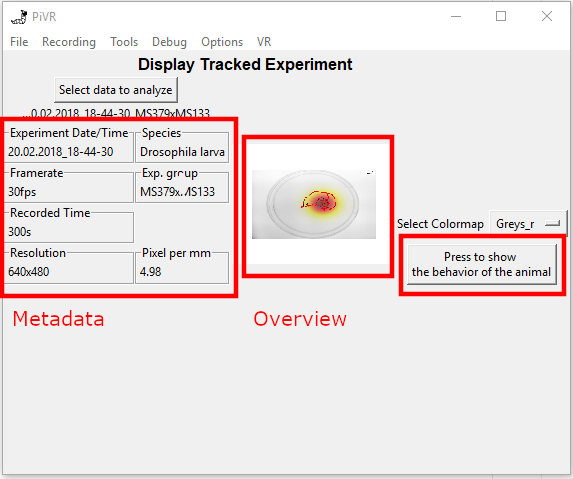

Supplement: S1 HTML — PiVR, Raspberry Pi Virtual Reality. (ZIP) [file pbio.3000712.s019.zip › S1HTLM/_images/5_1_ExprimentSelected.png]

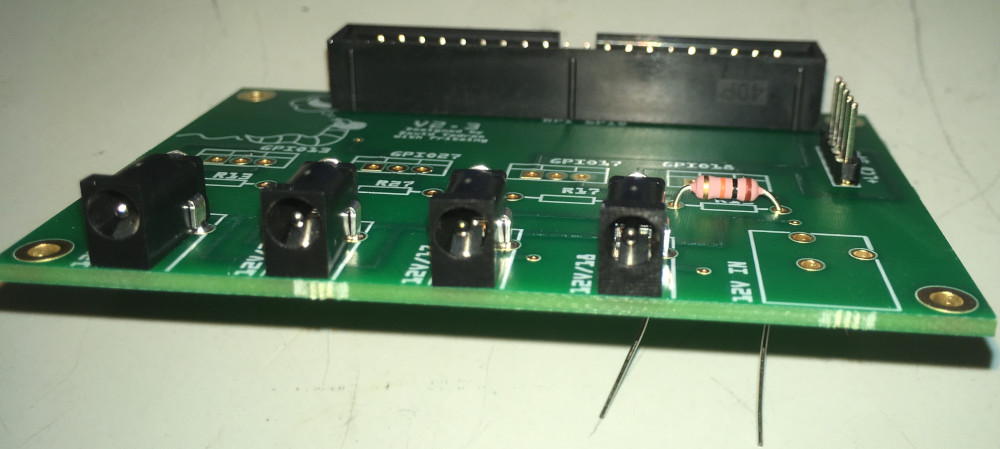

Supplement: S1 HTML — PiVR, Raspberry Pi Virtual Reality. (ZIP) [file pbio.3000712.s019.zip › S1HTLM/_images/S_8.jpg]

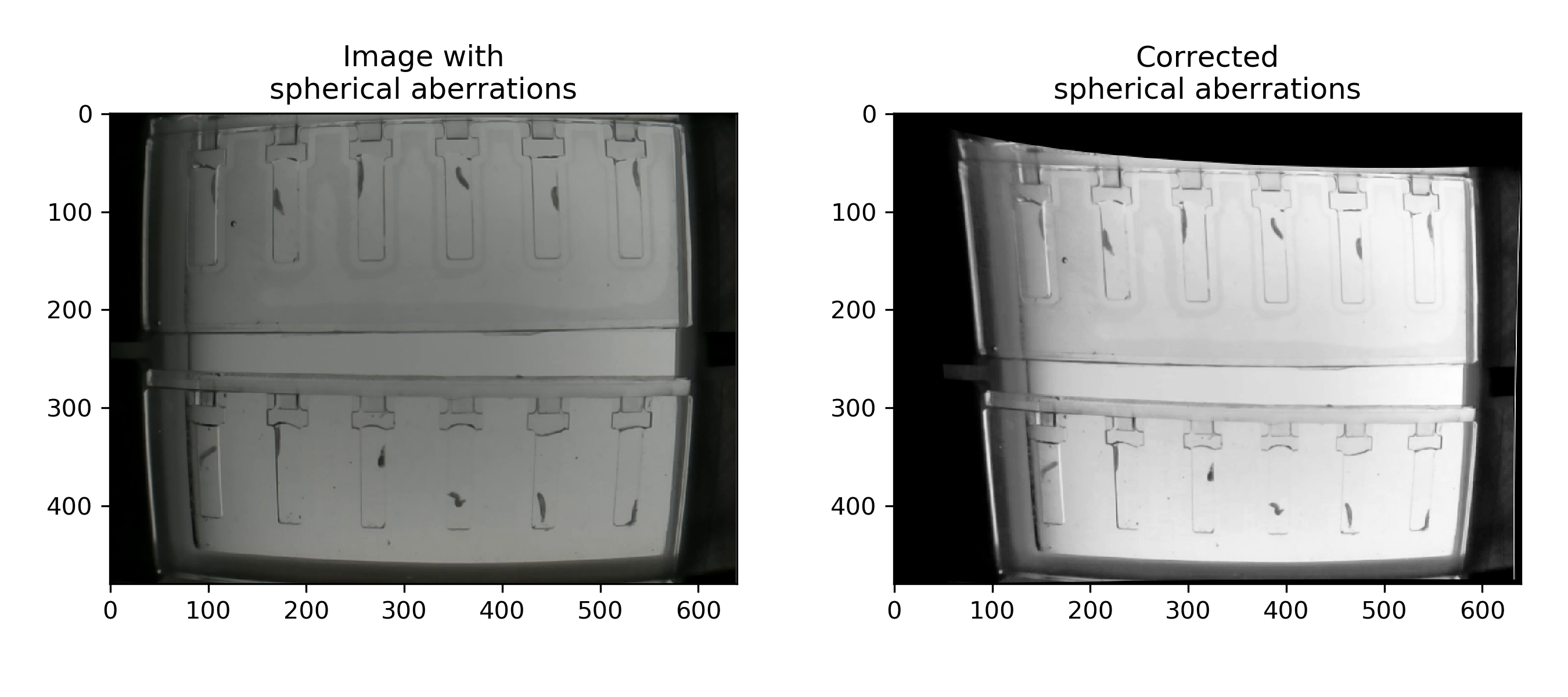

Supplement: S1 HTML — PiVR, Raspberry Pi Virtual Reality. (ZIP) [file pbio.3000712.s019.zip › S1HTLM/_images/undistortExample.png]

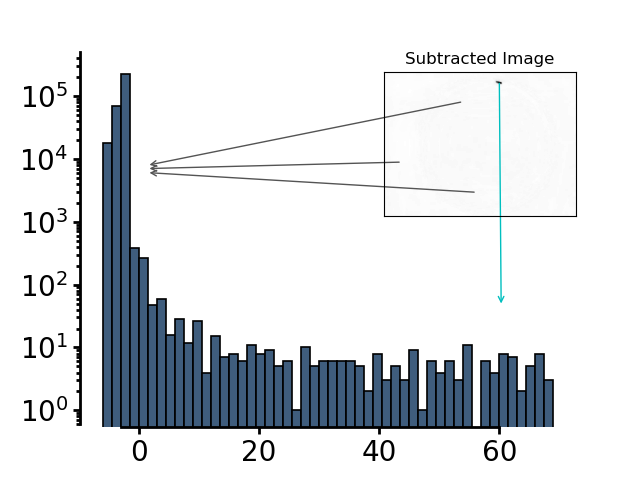

Supplement: S1 HTML — PiVR, Raspberry Pi Virtual Reality. (ZIP) [file pbio.3000712.s019.zip › S1HTLM/_images/Mode2Histogram.png]

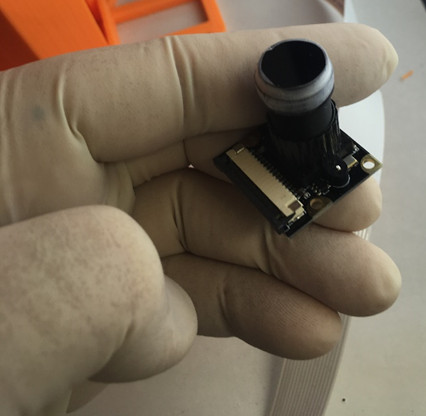

Supplement: S1 HTML — PiVR, Raspberry Pi Virtual Reality. (ZIP) [file pbio.3000712.s019.zip › S1HTLM/_images/16_Camera_with_LP_filter.jpg]

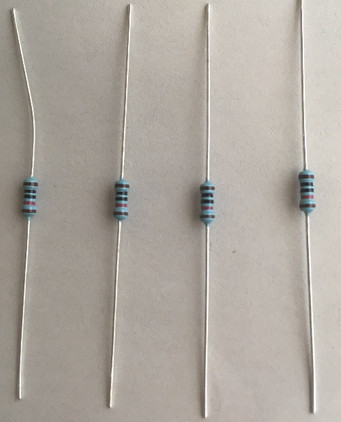

Supplement: S1 HTML — PiVR, Raspberry Pi Virtual Reality. (ZIP) [file pbio.3000712.s019.zip › S1HTLM/_images/2_5_resistors.jpg]

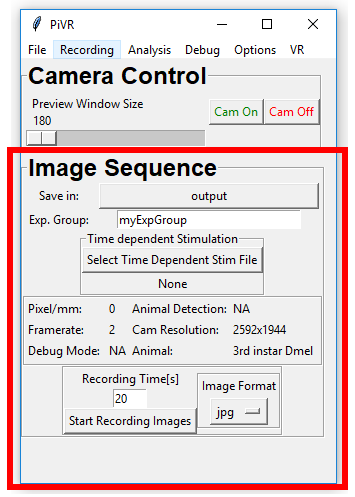

Supplement: S1 HTML — PiVR, Raspberry Pi Virtual Reality. (ZIP) [file pbio.3000712.s019.zip › S1HTLM/_images/ExperimentControlFrameImageSequence.png]

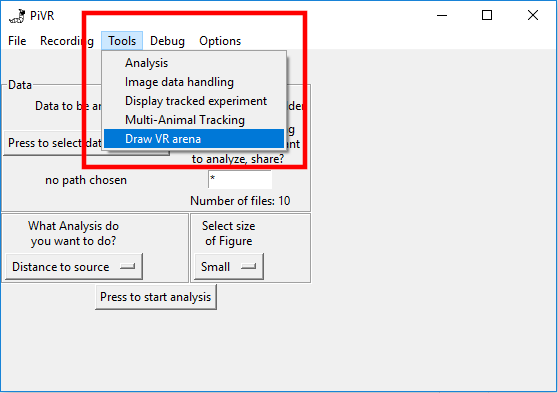

Supplement: S1 HTML — PiVR, Raspberry Pi Virtual Reality. (ZIP) [file pbio.3000712.s019.zip › S1HTLM/_images/8_DrawVRArena_menubar.png]

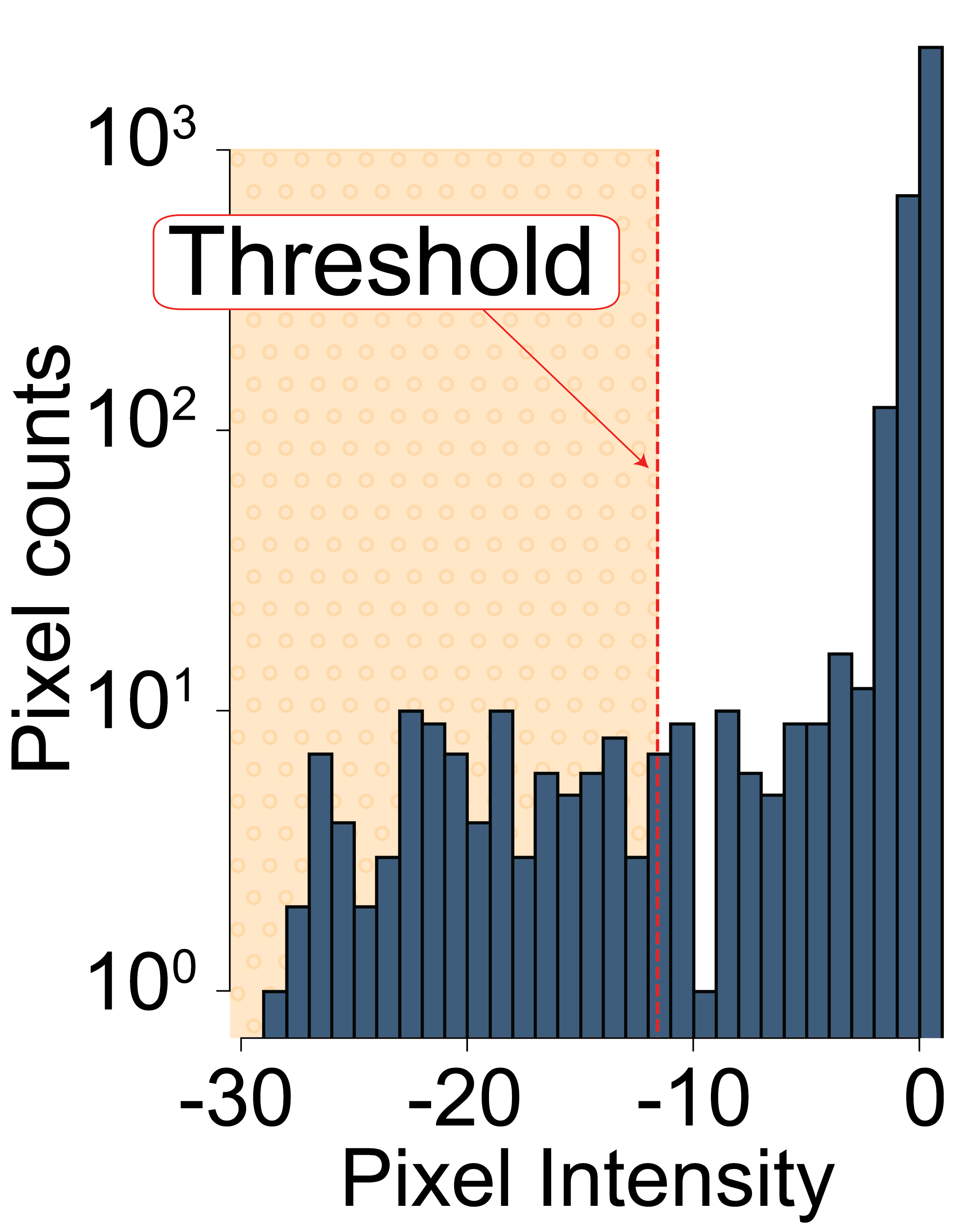

Supplement: S1 HTML — PiVR, Raspberry Pi Virtual Reality. (ZIP) [file pbio.3000712.s019.zip › S1HTLM/_images/FigS4_Histogram2.png]

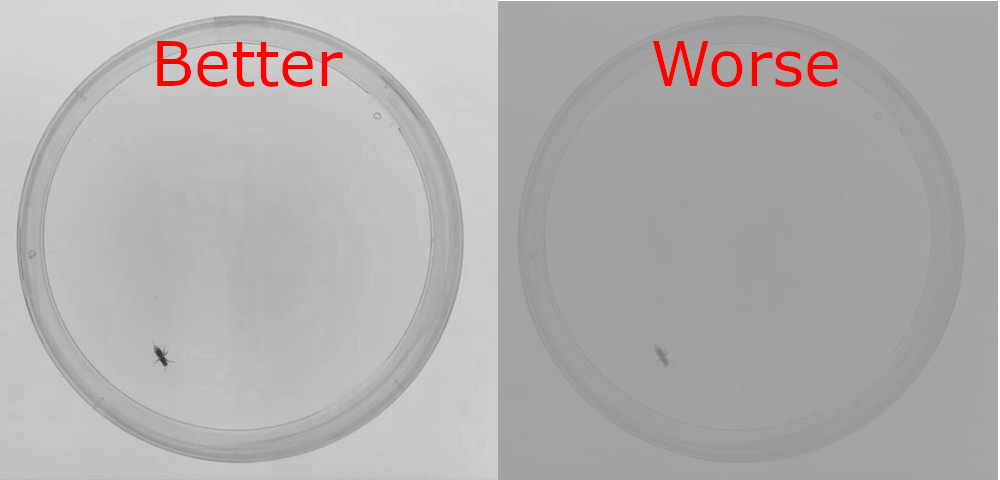

Supplement: S1 HTML — PiVR, Raspberry Pi Virtual Reality. (ZIP) [file pbio.3000712.s019.zip › S1HTLM/_images/ExampleContrast.png]

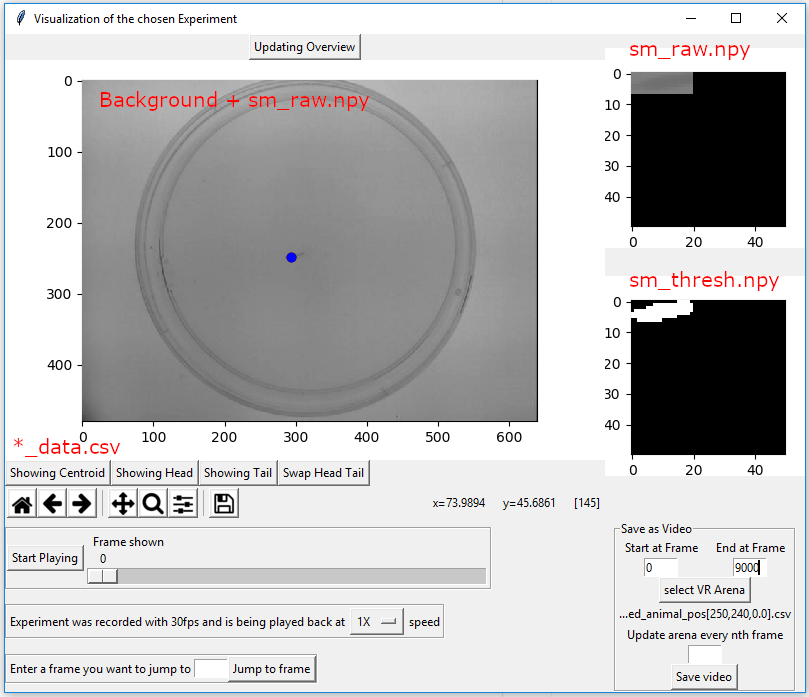

Supplement: S1 HTML — PiVR, Raspberry Pi Virtual Reality. (ZIP) [file pbio.3000712.s019.zip › S1HTLM/_images/5_2_ExperimentDisplay.png]

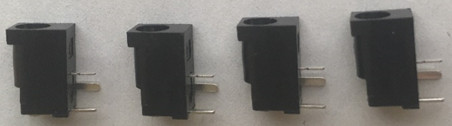

Supplement: S1 HTML — PiVR, Raspberry Pi Virtual Reality. (ZIP) [file pbio.3000712.s019.zip › S1HTLM/_images/2_4_Jack3_5.jpg]

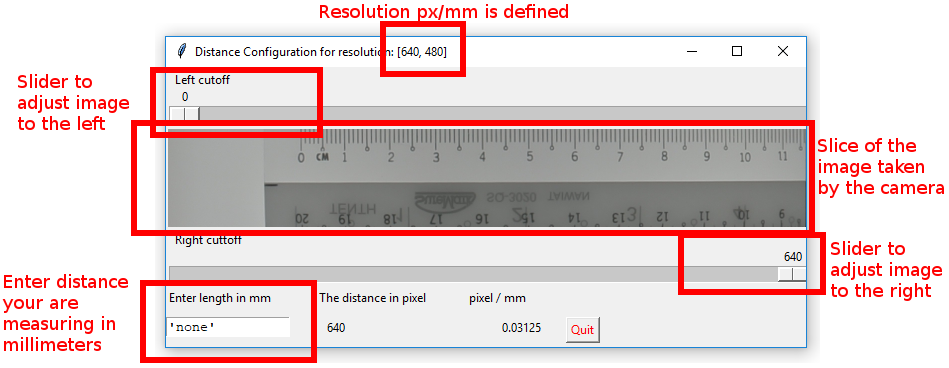

Supplement: S1 HTML — PiVR, Raspberry Pi Virtual Reality. (ZIP) [file pbio.3000712.s019.zip › S1HTLM/_images/DistanceConfigurationOverview.png]
