# Supplementary material for: PiVR: An affordable and versatile closed-loop platform to study unrestrained sensorimotor behavior
Source: PLoS Biol. 2020 Jul 14;18(7):e3000712. doi: 10.1371/journal.pbio.3000712 (PMC7360024; doi:10.1371/journal.pbio.3000712)
Supplement: S2 Table — (1) Optogenetic activation in closed-loop experiments. FreemoVR and Stytra are designed to present complex 3D and 2D visual stimuli, respectively. (2) Maximal frequency of the closed-loop stimulus. (3) Maximal time between an action of the tracked animal and the update of the hardware presenting the virtual reality. (4) Most tracking algorithms monitor the position of the centroid. Some tools will automatically detect other features of the tracked animal. (5) Some tracking algorithms are designed to specifically identify animals with a stereotypic shape, size, and movement. Other tracking algorithms are more flexible. (6) Assessed based on published information. (7) Bonsai was used in the optoPAD system, which uses optogenetics [53]. The information presented in this comparative table relies on the following publications: FlyPi [58]; Ethoscope [23]; FreemoVR [11]; Bonsai [57]; PiVR, present manuscript. PiVR, Pi Raspberry Virtual Reality. (PDF) [file pbio.3000712.s018.pdf]

	FlyPi	Ethoscope	FreemoVR	Stytra	Bonsai	PiVR
**Closed-loop capability**	NO	YES	YES	YES	YES	YES
**In-built optogenetic capability(1)**	N/A	YES	Not shown	Not shown	YES(7)	YES
**Minimum loop time(2)**	N/A	250 ms	10 ms	16 ms	8 ms	15 ms
**Minimum latency(3)**	N/A	Not documented	60-70 ms	20-50 ms	Not documented	<30 ms
**Multiple-point tracking(4)**	N/A	NO (centroid)	YES	YES (eye & tail tracking)	NO (centroid)	YES (head, centroid, tail)
**Versatility of in-built tracking algorithm(5)**	N/A	LOW (flies)	HIGH (small animals)	LOW (fish)	HIGH (fish, flies, rodents)	HIGH (small animals)
**Documentation for hardware assembly(6)**	Detailed	Detailed	Concise	Concise	N/A	Detailed
**Approximate cost of one setup**	<$200 + Monitor	$100 + $500 (reg. PC)	~$4000	$1000-3000 + $1000 (powerful PC)	>$1000 + $1000 (powerful PC)	$350-500
