# Supplementary material for: PiVR: An affordable and versatile closed-loop platform to study unrestrained sensorimotor behavior
Source: PLoS Biol. 2020 Jul 14;18(7):e3000712. doi: 10.1371/journal.pbio.3000712 (PMC7360024; doi:10.1371/journal.pbio.3000712)
Supplement: S1 HTML — PiVR, Raspberry Pi Virtual Reality. (ZIP) [file pbio.3000712.s019.zip › S1HTLM/index.html]

  


PiVR 1.3.12 documentation


PiVR

latest

- PiVR: virtual reality for small animals
- 1. Build your own PiVR
- 2. Bill of Materials (BOM)
- 3. Step-by-step experimental guide
- 4. Code explanation
- 5. PiVR Software Manual
- 6. Explanation of PiVR output
- 7. Tools
- 8. Advanced topics
- 9. PiVR software installation
- 10. PiVR software documentation
- 11. FAQ
- 12. Contact

PiVR

- Docs »
- PiVR 1.3.12 documentation
- Edit on GitLab

---

# PiVR: virtual reality for small animals¶

The Raspberry **Pi** based **V**irtual **R**eality system (PiVR) is
a virtual reality system for small animals. It has been developed by
David Tadres and Matthieu Louis (Louis Lab).

- The code is open source (BSD license)
- The source code for all the PiVR software can be found on Gitlab
- You can also find a Bug Tracker on Gitlab

Leonard the larva - always chasing that virtual banana smell.¶

## What can PiVR do?¶

### PiVR has been used to create virtual odor realities for fruit fly larvae.¶

Trajectory of a *Drosophila* larva in a virtual odor reality. The
larva expresses the optogenetic tool Chrimson in the *Or42a*
expressing olfactory sensory neuron.¶

### PiVR has also been used to create virtual taste realities for adult fruit flies.¶

Trajectory of an adult *Drosophila* fly in a virtual taste reality.
The fly expresses the optogenetic tool Chrimson in the *Gr66a*
expressing sensory neurons.¶

### PiVR was also used to create a virtual light bulb for a number of animals, including larval zebrafish.¶

Trajectory of a zebrafish (D. rerio) larva exposed to a virtual
white light source.¶

## Sounds great. I want one! How?¶

Please follow the Build your own PiVR

## I’ve got a setup. How do I use it?¶

If you are a first time use, check out the Step-By-Step Guide which will walk you through each of
the four recording modes:

1. Tracking single animal
2. Virtual Reality experiments
3. Image Sequence Recording
4. Video Recording

You have just run an experiment. What do make of the output data?
See here to understand what the files mean and what
the contain.

To see how PiVR can help you analyse data check out the
tools available on the PC version of PiVR.

### Advanced documentation¶

If you are running into trouble with the closed loop tracking, please
head over to How to simulate real time tracking.

If you want to track an animal that is not available under
Select Animal, please read the
How to define a new animal chapter.

If you want to understand what each button in the GUI is doing,
please see the PiVR Software Manual.

If you want to gain a high-level understanding on how the code
identifies the animal and tracks them please read the
Code Explanation

The annotated source code can be found here
and on the Gitlab page.

## Content¶

PiVR has been developed by David Tadres and Matthieu Louis (Louis Lab).

### Build your own PiVR¶

#### Standard and High Powered version¶

PiVR comes in two versions:

1. The Standard version: LED strips are used for both the
   backlight and stimulation light. We have measured around
   \(2{\mu}W/mm^2\) for red light intensity and around
   \(8000 Lux\) when measuring white light intensity.
   Please see the Bill of Materials for components you
   must buy to build the setup.
2. The High Powered Version: The stronger LEDs require not only a
   different arena but also dedicated LED drivers which provide a
   constant current. We have measured light intensities in the
   order of \(40 - 50{\mu}W/mm^2\) for the red LEDs.

   The detailed instructions to build a high powered PiVR version
   will be uploaded shortly.

#### Building the Standard version¶

Warning

You will be handling sensitive electronic equipment. As you might
be electrically charged, always touch something metallic that is
connected to the ground before unpacking anything from an
electrostatical shielding bag. A radiator will do fine.

Note

You can of course change the order of how to build the different
parts. I build them in this order as there are necessary delays
such as installing the operation system on the Raspberry Pi.

Note

We used an Ultimaker 3 with a Ultimaker Print Core AA (0.8mm) to
print the PLA and a Ultimaker Print Core BB (0.8) to print the
PVA support material. STL files were converted using Ultimaker
CURA software.

1. 3D print each part in the folder PiVR/Hardware/3D printer files.
   We used an Ultimaker 3 and printed with Standard PLA. We used PVA as
   support material. For best results print Casing, CameraHolder,
   TowerBottom and TowerTop with support material.
2. Obtain the Printed Circuit Board (PCB). Find the blueprint in
   PiVRHardwarePCB. I have used the software
   Fritzing to design the board. I
   have used the company Aisler.net to print
   the circuit boards.
3. Get the following items to solder the PCB board:

   > Important
   >
   > Before touching electrical components always touch something
   > metalic that is connected to the ground. For example a radiator.
   >
   > 1. Four (4) Transistors: 30N06L
   > 2. One (1) GPIO Header
   > 3. One (1) Barrel connector, 5.5mm Jack with 2.1mm center pole
   >    diameter
   > 4. Four (4) Barrel connectors, 3.5mm Jack with 1.35mm center pole
   > 5. Four (4) \(10k{\Omega}\) resistors
   > 6. Break off a 5 pin stretch from the Breakaway Headers. This can
   >    be done using scissors or pliers.
4. Solder the components on the PCB. See
   this section for
   detailed soldering instructions.

   > Important
   >
   > The correct **orientation** of the **GPIO header** and the
   > **Transistors** is crucial for PiVR to work correctly.
5. Cut off the excess wire of the resistors and the Transistors, e.g.
   with scissors.
6. Unpack the Touchscreen, remove the stand-off screws. Attach the
   monitor cable that came with the Touchscreen and the 4”
   5 pin cable.

   > Important
   >
   > The monitor cable must be inserted in the correct orientation.
   > When you look into the receptacle you’ll see that only one
   > side has connectors. Make sure that you insert the cable’s
   > connectors on the same side.
   >
   > Important
   >
   > Note the orientation of the 4” 5pin cable! Left is (+) while
   > Right is (-).
7. Place the Casing on top of the Touchscreen (it will only fit in
   the shown orientation). Organize the 4” 5pin cable and the
   monitor cable as shown in the picture. Use the M2.5x10mm screws to
   fix the casing to the touchscreen.
8. Prepare the SD card: Format the SD card using SD Formatter and load with
   NOOBs installation files as instructed here:
9. Connect monitor cable with the Raspberry Pi (with inserted SD
   card). Again, make sure you insert the cable in the correct
   orientation. Use M2.5x10 screws to attach the Raspberry Pi to the
   Casing.
10. Attach the PCB board on the right side of the casing using M2.5x10mm
    screws. Plug the 4” 5pin cable into the PCB in the correct
    orientation
11. Use the GPIO Ribbon cable to connect the PCB board with the
    Raspberry Pi. Thread the long camera cable through the slit as
    shown in the image below. Connect it to the Raspberry Pi Camera port.
12. Slide the CasingBackside (with attached pedestal) into the casing.
13. TODO show an image how you put the casing backside on casing
14. Drop a 2.5mm nut in each hole in the TowerPedestal. Use the M2.5x10
    screws to attach the TowerBottom to the Tower Pedestal
15. Using a hammer, drive the dowel pins into the TowerBottom. Then
    attach the TowerTop to it. In principle you can stack more TowerTops
    on top.
16. Attach the 800nm Longpass Filter to the Camera using Parafilm. It
    is best to wear gloves for this step.
17. Thread the camera cable from the Casing through the slit in the
    TowerBottom and through the slit of the Camera Holder.

    > Important
    >
    > Note the orientation to avoid having to curl the camera cable
    > in the camera holder
18. Attach the Camera Cable to the Camera in the **correct**
    orientation. Then screw the camera to the Camera Holder using the
    M2.5x10 screws. It is **not** necessary to fixate the screws with
    nuts!
19. Drop a 2.5mm nut in the hole in the Camera holder and use it to
    fasten the M2.5x10 screw. Then attach the CameraHolder to the
    Tower.
20. Plug the 5V power source into the micro USB slot of the Raspberry
    Pi(right side). After a couple of seconds the monitor should
    display a colorful image. Then the operating system installation
    will commence. Select the *Recommened* OS.
21. On the first startup the OS asks a couple of questions. The
    most important one is the language - make sure you choose the
    correct Keyboard layout. Make sure the Raspberry Pi is
    connected to the internet and download the
    `PiVR installation file`
22. Open the terminal. Then change directory to the ‘Downloads’ folder
    (or wherever you downloaded the file) and type:

    ```
    bash install_PiVR.sh
    ```
23. Now the arena will be built. In the folder PiVR/Lasercutter\_Files/
    you can find two vector graphic files that can be used to Lasercut
    a 20cm or 30cm arena, circular holes for M8 screws and small lines
    indicating the distance of 1cm on each side. For one arena you
    will need two acrylic plates.
24. Cut the 850nm (infrared) LED strips to the desired length (e.g. 20cm
    on a 20cm Arena) and attach them to the arena. You can choose the
    horizontal distance yourself. I usually use a distance of 3cm.

    > Important
    >
    > It will make soldering much easier if you make sure the
    > (+) and the (-) between the LED strips is consistent!

    Solder (+) to (+) from one side of the arena to the other
25. Attach the female Barrel Jack to a convenient copper dot on the
    LED strip. Then fix the Female Barrel Jack using a Hot Glue gun.
    Make sure you are leaving space for the M8 screw to pass through.

    > Important
    >
    > Usually the red wire of the Barrel Jack indicates (+)!
26. If you want to add a Stimulation LED strip (e.g. Red Stimulation
    light), just attach it in between the infrared LED strips, solder
    it as you did the 850nm LED strips and attach the female Barrel
    connector at a convenient location and fix it using the hot glue
    gun.
27. After inserting the M8 screws into the holes, thread a M8 nut on
    each of the screws about 2cm in. Put the second plate on top of
    the first and fasten it by threading a second M8 nut on top of the
    plate. Make sure the top plate is completely level by using a spirit
    level!
28. To connect PiVR with the arena a cable needs to be constructed.
    You will need two 5.5mm Male Barrel Jack, two 3.5 male solderable
    Barrel Jacks and around 20 Gauge wire.
29. Start by cutting a reasonable long piece of the wire, e.g. 50cm,
    but this depends on your application. Attach one side of the cable
    to the 3.5mm barrel jack. You may solder it, but be careful to
    only use minute amounts of solder. Then solder on the 5.5mm barrel
    jack on the other side, fixing it using the shrinking tubes.
30. Start the PiVR software by double clicking on the shortcut on the
    Desktop. Under ‘Options’ Select
    ‘Optimize Image’.
31. Connect the 12V power source (make sure you have an appropriate
    Ampere rating for the amount of LEDs you use!) to the 5.5mm Input
    on the setup. Do not plug it into the wall socket just yet!

    Warning

    Do not plug the 12V power source into the wall socket while you
    are handling the arena wires.

    Then connect the 3.5mm cable with the appropriate receptacle
    closest to the 5.5mm plug. Then plug the other side into the IR
    LEDs on the arena.

    Now you can plug in the 12V power source into the wall socket.
32. Turn the camera on (‘Cam On’). Then move the ‘Backlight Intensity’
    slider to something like 400‘000. You should see how the image on
    the top left of the screen lights up.

    Note

    Since the camera has a 800nm Longpass filter you shouldn’t see
    anything in the camera preview as long as the infrared light of
    the arena is off, **unless** you have a strong source of
    infrared radiation around, e.g. the Sun.
33. Connect a second 3.5mm cable just below the first. The other side
    goes into the first Stimulation Light in the arena.
34. When moving the slider labelled ‘Channel 1’ the stimulation LED
    should light up.
35. If these tests have been successful, congratulations, you’ve built
    your own PiVR

#### Detailed PCB soldering instructions (Standard Version)¶

Warning

Important! Make sure that the pins are not connected due to
imprecise soldering!

1. I prefer to solder the components on the PCB board in this
   particular sequence as I find it easiest to keep the components in
   place. Otherwise there is no reason to not solder components in
   any sequence you prefer!
2. To solder the PCB board you will need the following elements:

   > 1. Four (4) Transistors: 30N06L
   > 2. One (1) GPIO Header
   > 3. One (1) Barrel connector, 5.5mm Jack with 2.1mm center pole
   >    diameter
   > 4. Four (4) Barrel connectors, 3.5mm Jack with 1.35mm center pole
   > 5. Four (4) \(10k{\Omega}\) resistors
   > 6. Break off a 5 pin stretch from the Breakaway Headers. This can
   >    be done using scissors or pliers.
3. Take one of the small barrel plug and place it into the
   leftmost possible spot on the PCB board as shown.
4. Flip the PCB board while holding the small barrel plug in
   place. By placing it on the table, it should not move and allow
   you to easily solder the three pins of the barrel plug to the PCB
   as shown.
5. Continue to solder the other three small barrel plugs, one by one,
   onto the the PCB board.
6. Next, place the GPIO header in **exactly** the orientation shown in
   the image below onto the PCB board.
7. Flip the PCB board with the GPIO header around. As it now stands
   on the table it should be easy to solder. You do not have to
   solder every single pin to the PCB (minimum is shown on top
   picture) but it is recommended to solder more, ideally all. **Be
   sure the solder between the pins does not touch**
8. Place the 5-pin stretch of breakaway headers into the holes on
   the far right on the PCB. Make sure to place them in the correct
   orientation as shown in the picture.
9. Flip the PCB with the 5-pin stretch of breakaway headers around
   and solder the header to the PCB board.
10. Now to the resistors. Place a resistor in the indicated position:
11. Flip the PCB board around. If the resistor falls out, just fixate
    it by bending the wire as indicated here. Then solder it the the
    PCB board.
12. Do the same for the other three resistors.
13. Now take the large barrel connector and place it on the PCB at the
    indicated position
14. Flip the PCB board around and solder the large barrel connector to
    the PCB board.
15. Next, take one of the transistors and place it **exactly** as
    shown onto the PCB board.
16. Flip the PCB board around and solder the transistor to the board.
    Make sure the solder of the different pins does not touch the
    contact of one of the other pins! **Warning: Transistors are more
    heat sensitive compared to the other components you have used so
    far. Make sure to not let them heat up too much!**
17. Do the same for the other three transistors.
18. You must get rid of the elongated wiring of the transistors and
    especially the resistors as not doing so will 1) increase risk of
    shorting components and 2) it will physically be very hard to put
    the PCB board into the casing. While it is probably best to use
    the shown wire clipper, it is also possible to do that using
    normal scissor.

PiVR has been developed by David Tadres and Matthieu Louis (Louis Lab).

### Bill of Materials (BOM)¶

All components you need to build your

1. Standard PiVR
2. High Powered PiVR

#### Standard PiVR setup¶

For detailed information, scroll to the right.

This table is identical to the one found on
Gitlab which might be more convenient
when ordering parts.

Table Title¶


| Short Name | price 1x | price 7x | where | Full Name | NOTES | what for | company | part number | 1x what exactly? | price 1x | 7x what exactly? | price 7x |
| --- | --- | --- | --- | --- | --- | --- | --- | --- | --- | --- | --- | --- |
|  |  |  |  |  |  |  |  |  |  |  |  |  |
| General |  |  |  |  |  |  |  |  |  |  |  |  |
| RPi3 | $35.00 | $245.00 | https://www.newark.com/raspberry-pi/rpi3-modbp/sbc-arm-cortex-a53-1gb-sdram/dp/49AC7637 | Raspberry Pi 3 - Model B+ - 1.4GHz Cortex-A53 with 1GB RAM | Link is for RasPi 3 B+ maybe out of stock, then buy model B | brain of the setup | Adafruit | 3775 | 1xRasperry | $35.00 | 7x Rasperry | $245.00 |
| SD Card | $19.95 | $139.65 | https://www.adafruit.com/product/2693 | SD/MicroSD Memory Card - 16GB Class 10 - Adapter Included | Class 10 > at least write 10Mb/s | 16Gb to start up RPi (download Noobs for each) and to store some data | Adafruit | 2693 | 1x16Gb | $19.95 | 7x16Gb | $139.65 |
| RPi3 PSU | $8.99 | $62.93 | http://www.newark.com/stontronics/t5989dv/psu-rpi-5v-2-5a-multi-plug-blk/dp/81Y7474?MER=sy-me-pd-mi-acce | T5989DV-PSU, RPI, 5V, 2.5A, MULTI PLUG BLK |  | Power supply for the RPi | Newark element14 | 81Y7474 | 1xPower supply | $8.99 | 7xPower supply | $62.93 |
| Touchscreen | $60.00 | $420.00 | http://www.newark.com/raspberry-pi/raspberrypi-display/display-7-touch-screen-rpi-sbc/dp/49Y1712?st=%20raspberry%20%20pi%20%20boards | Raspberry Pi 7” Touchscreen LCD Display |  | Interact with PiVR | Newark element14 | 49Y1712 | 1xtouchscreen | $60.00 | 7xtouchscreen | $420.00 |
| Camera | $22.99 | $160.93 | http://www.waveshare.com/rpi-camera-f.htm | RPi Camera (F), Supports Night Vision, Adjustable-Focus |  | IR capable low cost camera | Waveshare | SKU: 10299 | 1xTypeF camera | $22.99 | 7xTypeF camera | $160.93 |
| Camera cable | $3.95 | $27.65 | https://www.adafruit.com/product/2143 | Flex Cable for Raspberry Pi Camera or Display - 1 meter |  | connect Rasbperry with camera, higher distance | Adafruit | PRODUCT ID: 2143 | 1x | $3.95 | 7x | $27.65 |
| 12V PSU | $7.99 | $55.93 | https://www.amazon.com/inShareplus-Mounted-Switching-Connector-Adapter/dp/B01GD4ZQRS/ref=pd\_sbs\_86\_1?\_encoding=UTF8&pd\_rd\_i=B01GD4ZQRS&pd\_rd\_r=AGMHAS5C8Q8PTSGCM955&pd\_rd\_w=Tq3dQ&pd\_rd\_wg=2uLF6&psc=1&refRID=AGMHAS5C8Q8PTSGCM955 | Power Supply Transformers LED Adapter 12V 2A Max 24 Watt for LED Strip Lights SMD 5050 3528 |  | 12V power source for LEDs | Amazon |  | 1x2A Power supply | $7.99 | 7x2A Power supply | $55.93 |
| LP filter | $22.50 | $157.50 | https://www.edmundoptics.com/optics/optical-filters/longpass-edge-filters/ir-80-800nm-12.5mm-dia.-longpass-filter/ | 800nm LP Filter |  | Restrict camera input to >800nm | Edmond Optics | 66-048 | 1x | $22.50 | 7x filter | $157.50 |
| 850nm LED | $9.90 | $69.80 | http://www.ledlightsworld.com/dc12v-smd3528300ir-infrared-850nm940nm-single-chip-flexible-led-strips-60leds-48w-per-meter-p-1000598.html | 850nm LED strip |  | Backlight lighting | LEDslightsworld | HK-F3528IR30-X | 1x100cm | $9.90 | 1x5m + 1x2m | $69.80 |
| 625nm LED | $21.74 | $152.18 | https://www.mouser.com/ProductDetail/Optek-TT-Electronics/OVQ12S30R7?qs=agb4oTp1Mj9i3RuPFjrhLw%3D%3D | 625nm LED strip |  | Activation of CsChrimson | Mouser | 828-OVQ12S30R7 | 2x500mm | $21.74 | 2\*7\*50cm | $152.18 |
| Diffusors | $65.01 | $82.80 | https://www.professionalplastics.com/PLEXIGLASS-ACRYLICSHEET-EXTRUDED | Plastic sheets | Thickness: 0.250 THICK, Type: WHITE#7328 EXTRUDED ACRYLIC FILM MASKED SHEET, Cut Size (inches): 7.9x7.9, Cut Tolerance: +/- 0.125 | Base, and diffuser for all the light | https://www.professionalplastics.com |  | 3x | $65.01 | 18x | $82.80 |
| Barrel plugs | $6.99 | $20.97 | https://www.amazon.com/2-1x5-5mm-Pigtails-Security-Rearview-Application/dp/B071GV5XDD/ref=pd\_day0\_hl\_60\_3/143-1881930-5573125?\_encoding=UTF8&pd\_rd\_i=B071GV5XDD&pd\_rd\_r=74e52552-8718-11e9-b1ac-6bd4bdce431e&pd\_rd\_w=oddiv&pd\_rd\_wg=YxNMs&pf\_rd\_p=ad07871c-e646-4161-82c7-5ed0d4c85b07&pf\_rd\_r=MXPEEFC04PYTR04R30Q1&psc=1&refRID=MXPEEFC04PYTR04R30Q1 | Barrel connectors, male & female |  | power the arena | amazon.com |  | 1x (4\*1) | $6.99 | 3x (4\*7=28) | $20.97 |
| Small screws | $8.05 | $40.25 | https://www.mcmaster.com/91290a103 | M2.5x10 |  | fix different components to 3D printed parts | McMaster |  | 1x (28screws/setup) | $8.05 | 5x (7\*28screws = 196) | $40.25 |
| Small nuts | $7.34 | $7.34 | https://www.mcmaster.com/90695a031 | M2.5 nuts |  | fix cam holder | McMaster |  | 1x (3nuts/ setups) | $7.34 | 1x (7x3nuts=21) | $7.34 |
| Large Screws | $6.91 | $13.82 | https://www.mcmaster.com/91290a448 | M8x50 |  | Keep arena plates in place | McMaster |  | 1x (1x4) | $6.91 | 2x (7x4=28) | $13.82 |
| Large Nuts | $8.82 | $8.82 | https://www.mcmaster.com/90592a022 | M8 nuts |  | Keep arena plates in place | McMaster |  | 1x (1x8) | $8.82 | 1x (7\*8=72) | $8.82 |
| Wire | 15.99 | $31.98 | https://www.amazon.com/StrivedayTMFlexible-Silicone-Electric-electronic-electrics/dp/B01LH1FR6M/ref=sr\_1\_4?s=hi&ie=UTF8&qid=1527729717&sr=1-4&keywords=gauge+22+wire | electrical wire, 22-gauge |  | wire arena | Amazon |  | 1x | 15.99 | 2x | $31.98 |
| HS tubing | $8.99 | $17.98 | https://www.amazon.com/560PCS-Heat-Shrink-Tubing-Eventronic/dp/B072PCQ2LW/ref=pd\_sim\_469\_2?\_encoding=UTF8&pd\_rd\_i=B072PCQ2LW&pd\_rd\_r=514JB4QKSGX13RT7XS76&pd\_rd\_w=yX88x&pd\_rd\_wg=YkZqg&psc=1&refRID=514JB4QKSGX13RT7XS76 | Heat shrink tubing |  | safely connect custom wires to each other | Amazon |  | 1x | $8.99 | 2x | $17.98 |
| Keyboard/Mouse | $22.95 | $160.65 | https://www.adafruit.com/product/2129 | Keyboard and mouse |  | On-screen keyboard hard to use! | Adafruit | 2876 | 1x | $22.95 | 7x | $160.65 |
| Dowel pins | $4.20 | $4.20 | https://www.mcmaster.com/97155a639 | Acetal Dowel Pins, 1/4” Diameter, 3/4” Long |  | Connect Tower parts | McMaster | 97155A639 | 1x pack of 50 | $4.20 | 1x pack of 50 (3x7=21) | $4.20 |
|  |  |  |  |  |  |  |  |  |  |  |  |  |
| PCB Board stuff |  |  |  |  |  |  |  |  |  |  |  |  |
| Pins | $4.95 | $4.95 | https://www.adafruit.com/product/392 | Break Away Headers - Straight |  | PCB 5V connector | Adafruit | 392 | 1x | $4.95 | 1x | $4.95 |
| Header | $0.75 | $5.25 | https://www.adafruit.com/product/1993 | Raspberry Pi GPIO Shrouded Header - 2x20 |  | connect PCB to Raspberry Pi | Adafruit | 1993 | 1x | $0.75 | 7x | $5.25 |
| Cable | $2.95 | $20.65 | https://www.adafruit.com/product/1988 | Raspberry Pi GPIO Ribbon Cable |  | cable to connect PCB to Raspberry Pi | Adafruit | 1988 | 1x | $2.95 | 7x | $20.65 |
| Cable | $0.95 | $5.70 | https://www.sparkfun.com/products/10365 | Jumper Wire - 0.1”, 5-pin, 4 | Shorter might be better |  | Sparkfun | PRT-10365 | 1x | $0.95 |  | $5.70 |
| Power plug | $0.95 | $5.70 | https://www.sparkfun.com/products/10811 | large power connector, on PCB |  | connect wall outlet to PCB, inner pole diamete or 2.1mm makes it incomaptible with the large power connector between PiVR and the arena | Sparkfun | PRT-10811 | 1x | $0.95 |  | $5.70 |
| Transitor | $4.88 | $29.28 | https://www.mouser.com/ProductDetail/ON-Semiconductor-Fairchild/FQP30N06L?qs=sGAEpiMZZMshyDBzk1%2fWi1oKJWRB0GXw5ym5GJISPKM%3d | Transistor |  | control LEDs | Mouser | 512-FQP30N06L | 4x | $4.88 | 24x | $29.28 |
| Resistor | $0.68 | $4.08 | https://www.mouser.com/ProductDetail/Yageo/FMP100JR-52-10K?qs=%2fha2pyFadugHhX%2fbCuGNwYsjyxsUwwbCIALRKO4JZecDxEf1h08Etw%3d%3d | Resistance |  | pull down ground | Mouser | FMP100JR-52-10K | 4x | $0.68 | 24x | $4.08 |
| Power plug | $2.52 | $15.12 | https://www.digikey.com/products/en?keywords=CP-2519-ND | small power connector, on PCB |  | connect PiVR to arena | DigiKey | CP-2519-ND | 4x | $2.52 | 24x | $15.12 |
| Power cable | $4.16 | $24.96 | https://www.digikey.com/products/en?keywords=CP3-1003-ND | small power connector, cable |  | connect PiVR to arena | DigiKey | CP3-1003-ND | 4x | $4.16 | 24x | $24.96 |
|  |  |  |  |  |  |  |  |  |  |  |  |  |
| To print |  |  |  |  |  |  |  |  |  |  |  |  |
| 3d Print | ? 20-100$ |  |  |  |  |  |  |  |  | ? 20-100$ |  |  |
| PCB board | $24.00 | $48.00 | https://aisler.net/ |  |  |  |  |  | 1x | $24.00 | 6x | $48.00 |
|  |  |  |  |  |  |  |  |  |  |  |  |  |
|  | 1 Setup | 7 Setups |  |  |  |  |  |  |  |  |  |  |
| Total Price | $415.05 | $2,044.07 |  |  |  |  |  |  |  | $415.05 |  | $2,044.07 |
|  |  |  |  |  |  |  |  |  |  |  |  |  |
| Price per setup | $415.05 | $340.68 |  |  |  |  |  |  | 1x | $415.05 |  | $340.68 |
|  |  |  |  |  |  |  |  |  |  |  |  |  |
| Optional Components |  |  |  |  |  |  |  |  |  |  |  |  |
| 530nm LED | $29.14 | $152.18 | https://www.mouser.com/ProductDetail/Optek-TT-Electronics/OVQ12S30G7?qs=agb4oTp1Mj9I3edgHo6%252bZg%3d%3d | 530nm LED strip |  | Activation of GtACR | Mouser | 828-OVQ12S30G7 | 2\*50cm | $29.14 | 14x (2\*7\*50cm) | $152.18 |
| Boxes | $33.50 | $33.50 | https://www.uline.com/Product/Detail/S-11370/Corrugated-Boxes-200-Test/10-x-10-x-20-Corrugated-Boxes | 10 x 10 x 20” Corrugated Boxes |  | some light blocking carton to protect animals from light | S-11370 |  | 1x | $33.50 | 1x | $33.50 |
|  |  |  |  |  |  |  |  |  |  |  |  |  |
| Useful Tools |  |  |  |  |  |  |  |  |  |  |  |  |
| Hex screwdriver | $14.39 |  | https://www.amazon.com/Aiskaer-full-color-lightweight-titanium-Helicopter/dp/B01FK6TJ9G/ref=sr\_1\_8?s=hi&ie=UTF8&qid=1527709922&sr=1-8&keywords=m3+hex+wrench |  | Need M2.5 |  |  |  |  | $14.39 |  |  |
| Hex allen wrench | $11.99 |  | https://www.amazon.com/dp/B071NBGBQD/ref=sspa\_dk\_detail\_2?psc=1&pd\_rd\_i=B071NBGBQD&pd\_rd\_wg=kmByc&pd\_rd\_r=H240CYDM209AMVWTG5PS&pd\_rd\_w=9dkZE |  | Need M2.5 |  |  |  |  | $11.99 |  |  |
| Hex screwdriver bits | $12.50 |  | https://www.amazon.com/Toolcool-Broppe-Magnetic-Screwdriver-Length/dp/B06ZZJ9YT2/ref=pd\_sim\_21\_9?\_encoding=UTF8&pd\_rd\_i=B06ZZJ9YT2&pd\_rd\_r=MBHSJR7A3AFE8B0ZNFP8&pd\_rd\_w=TvLBR&pd\_rd\_wg=O3FQt&psc=1&refRID=MBHSJR7A3AFE8B0ZNFP8 |  | Need M2.5 M8 |  |  |  |  | $12.50 |  |  |
| Metric screw drill bits | $13.89 |  | https://www.amazon.com/Migiwata-Metric-Spiral-Combination-M3-M10/dp/B07122KD34/ref=sr\_1\_9?s=hi&ie=UTF8&qid=1527739748&sr=1-9&keywords=m3+drill+bit |  | Need M2, M4, M6 and M8 |  |  |  |  | $13.89 |  |  |
| Metric drill bits | $9.56 |  | https://www.amazon.com/dp/B0732RX5ZF/ref=twister\_B075PSJR66?\_encoding=UTF8&psc=1 |  |  |  |  |  |  | $9.56 |  |  |
| Parafilm | $21.00 |  | https://www.amazon.com/Parafilm-PM-996-Purpose-Laboratory-Film/dp/B004VQP6CQ/ref=asc\_df\_B004VQP6CQ/?tag=hyprod-20&linkCode=df0&hvadid=198056450525&hvpos=1o1&hvnetw=g&hvrand=17778218174422644869&hvpone=&hvptwo=&hvqmt=&hvdev=c&hvdvcmdl=&hvlocint=&hvlocphy=9031645&hvtargid=aud-801738734305:pla-350206680429&psc=1 |  |  | attach filter to camera |  |  |  | $21.00 |  |  |
| solder station | $138.95 |  | https://www.amazon.com/dp/B00C2BHTBI/ref=sspa\_dk\_detail\_3?psc=1&pd\_rd\_i=B00C2BHTBI&pd\_rd\_wg=QoSMF&pd\_rd\_r=GY053WQMKCPT0MQS706Z&pd\_rd\_w=xlq2S |  |  |  |  |  |  | $138.95 |  |  |
| solder | $7.59 |  | https://www.amazon.com/WYCTIN-Solder-Electrical-Solderding-0-11lbs/dp/B071G1J3W6/ref=sr\_1\_5?s=hi&ie=UTF8&qid=1527741062&sr=1-5&keywords=solder |  |  |  |  |  |  | $7.59 |  |  |
| solder smoke absorber | $71.74 |  | https://www.amazon.com/dp/B00FZPSEY4/ref=twister\_B07CZ2B5L2?\_encoding=UTF8&th=1 |  |  |  |  |  |  | $71.74 |  |  |
| Wire stripper | $19.95 |  | https://www.amazon.com/dp/B07FP1XXQM/ref=sspa\_dk\_detail\_1?psc=1&pd\_rd\_i=B07FP1XXQM |  |  |  |  |  |  | $19.95 |  |  |
| power drill | $24.97 |  | https://www.amazon.com/BLACK-DECKER-DR260C-Drill-Driver/dp/B00T2VJ93C/ref=sr\_1\_8?s=hi&ie=UTF8&qid=1527741224&sr=1-8&keywords=power+drill |  |  |  |  |  |  | $24.97 |  |  |
| Hot Glue gun + glue | $23.99 |  | https://www.amazon.com/Cobiz-Premium-Sticks-Christmas-Decoration/dp/B0721PTD5B/ref=sr\_1\_1?s=hi&ie=UTF8&qid=1527741416&sr=8-1&keywords=Cobiz+Full+Size |  |  |  |  |  |  | $23.99 |  |  |

#### High Powered PiVR setup¶

Coming soon…

PiVR has been developed by David Tadres and Matthieu Louis (Louis Lab).

### Step-by-step experimental guide¶

Read this guide if you:

1. Just want to run an experiment
2. Do not (yet) want to go too much into the details of how the
   tracking algorithm works.

#### Single animal tracking¶

Press here to learn how to select the Single
Animal tracking menu. Use this option to track single animals. It is
possible to provide a time-dependent stimulation. See here to learn how to present a single animal with a virtual
reality.

Important

Several options will open a pop-up. You must close the pop-up in
order to interact with the main window.

##### Select Organism¶

For proper identification and tracking you first have to define which
organism you are using. See here to
learn how to select your organism.

Important

If you are using an animal that is **not** in the List of
Organisms, please see
here.

##### Define pixel per mm¶

As you can put the camera at a variety of distances to the arena it
is **essential** to define the pixel per mm ratio. Please see
here to learn how to do that.

##### Setting up the arena and the camera¶

It is important to understand how PiVR is able to detect and track an
animal in order to master this method.

The tracking software can only track what is in the field of view of
the camera. If your animal can leave the field of view of the camera,
tracking will stop and you will see an error.

The animal can run outside of the Field of View (FOV) during the
experiment as the petri dish is not entirely visible using the
camera. Adjust the dish that constrains animal movement to be in
the FOV of the camera.¶

The algorithm works best if the background has as little contrast as
possible

The background is very uneven as the screw of the arena is
visible (bottom left) and large portions of the image are just
black while others are white. Adjust the camera and/or the arena
so that the image only consists of the
white background illumination (and the animal).¶

The algorithm works best if the animal has a **high contrast**
relative to the rest of the structure in the image.

Left: The fly can clearly be seen relative to the background.
Right: The fly can be seen, but does not have a lot of contrast
relative to the background. Try to improve the image so that it looks more like the one on the left.¶

To set up PiVR to get an image as shown on the left, please follow
these Instructions.

##### Animal detection¶

The animal needs to be detected **before** the actual data collection
starts. It is also necessary to save a *Background* image for later
use. Ideally this Background image does not contain the animal that
should be tracked.

There are three different animal detection modes
(press here how to select them), each with
it’s own advantages and drawbacks. See
here for a an illustration of
the different methods.

1. **Standard - Mode#1:** This mode will allow you to track a wide
   variety of animals without a lot of optimization of the
   camera nor image.

   We have used this mode to track adult flies

   **Advantages**:

   Should work with pretty much any organism if it has been defined
   before.

   Straightforward to run: Place animal, press start tracking, done

   **Disadvantages**:

   The Background image will have a partially visible animal.

   You can’t align any virtual reality arenas relative to the inital
   movement direction of the animal.

   See details here
2. **Pre-define Background - Mode#2:** If you need to define a cleaner
   background image, this mode can be useful. You take an image
   before the animal is placed, then add **only** the animal.

   We have used this mode to track zebrafish larvae

   **Advantages**:

   Can track any animal (probably better than Mode#1) if it has been
   defined before.

   Will give a clean background image

   **Disadvantages**:

   As with Mode#1 you can’t align any virtual reality arenas relative
   to the inital movement direction of the animal.

   Extremely sensitive: If you have to move anything (such as holding
   up a lid of a petri dish where the animal is supposed to behave)
   this Mode probably won’t work well.

   See details here
3. **Reconstruct Background by Stitching - Mode#3:** Should produce the
   same clean background image as Mode#2. Only works if animal
   clearly stands out (has high contrast) in its local environement.

   We have used this mode with fruit fly larvae.

   **Advantages**:

   Allows the usage of aligned virtual reality arenas as the initial
   movement direction of the animal is detected.

   Will give a clean background image.

   **Disadvantages**:

   High contrast requirement hard to fullfill, therefore this mode
   does **not** work well with fast moving animals. Both because
   animals move quickly to the edge and because the area that must be
   taken into account by the detection algorithm increases with the
   speed of the animal.

   Can be difficult to use.

   See details here.

**To Summarize**

If you choose Mode 1 or Mode 3:

1. Place the animal in the arena, taking the guide above into account.
2. Press ‘Start Tracking’

If you choose Mode 2:

1. Prepare the arena *without* the animal, taking the guide
   above into account.
2. Press ‘Start Tracking’
3. Place the animal into the arena.
4. Press ‘Ok’.

##### Animal tracking¶

After successful detection, the tracking algorithm starts following
the animal automatically without the user having to do anything.

While tracking is in progress, the preview window will overlay most
of the monitor and the GUI is not responsive.

There is no status bar available that could be shown during the
tracking.

It is also not possible to cancel the experiment using a
button.

##### After Animal Tracking¶

Once tracking is finished (either because the animal was tracked for
the defined time or due to an error), the preview window will become
much smaller again.

After saving all the data (which can take a couple of seconds) the
GUI becomes responsive again.

#### Single animal Tracking with Virtual Reality¶

Press here to learn how to select the VR Arena option.

Besides selecting a virtual arena (as described in the link) everything is identical to Single Animal Tracking.

#### Taking full frame images¶

Press here to learn how to select the single
image recording option.

This option is useful if you have several animals that you want to
record simultanously at a low framerate. It is possible to provide a
time-dependent stimulation. Compared to the video option the resulting images are uncompressed. The
disadvantage is the lower framerate and the additional hard drive
space necessary to save all the images.

1. Place your arena with the animals you would like to observe into
   the field of view of the camera.
2. Use this guide to get the optimal
   image for your experiment.
3. Press ‘Start Recording Images’

Important

This option uses a lot of hard drive space. Make sure there is
enough space left on your SD card before doing such an experiment.

#### Recording a video¶

Press here to learn how to select the Video
Recording option.

This option is useful if you have several animals that you want to
record simultanously at a high (determined by your resolution, but at
640x480 you should be able to sustain >80 fps) framerates. It is
possible to provide a time-dependent stimulation. Compared to the
full frame option the video will allow for
much higher framerates while using a fraction of hard drive space.
Videos are encoded in the h264 format. They can be converted into
any other format using ffmpeg.


1. Place your arena with the animals you would like to observe into
   the field of view of the camera.
2. Use this guide to get the optimal
   image for your experiment.
3. Press ‘Start Video Recording’

PiVR has been developed by David Tadres and Matthieu Louis (Louis Lab).

### Code explanation¶

Read this guide if you:

1. Want to gain a high-level understanding of how the detection
   and tracking algorithm work.
2. Do not (yet) want to look into the source code

#### Animal detection¶

The tracking algorithm depends the
approximate location of the animal and a background image in order to
identify the animal. The *Animal Detection* modes
(guide, and
selection) allow the user to choose
between one of three Modes depending on the experimental
setup and question asked. (Source Code)

##### Standard - Mode 1¶

**User perspective:**

1. Place animal in the final dish on the arena, taking the
   optimal image parameters into account
2. Press *Start*

**Behind the scenes:**

1. When the user presses *Start* the camera starts sending pictures
   to the tracking software.
2. For the first frame, the image is just filtered using a gaussian
   filter with sigma depending on the properties saved in
   ‘list\_of\_available\_organisms.json’. In short, the sigma will be
   half the minimal expected cross-section of the animal. Then it
   takes the next frame.
3. Starting with the second frame, the mean of the current and all
   previously taken images is taken.
4. The current frame is then subtracted from the mean of all previous
   frames
5. The subtracted image has a trimodal distribution of pixels - The
   main peak is the background (grey arrows). The intensity values
   for pixels where the animal was before but isn’t anymore has
   positive values (cyan arrow). The intensity values for the pixels
   where the animal only recently moved in has negative values
   (magenta arrow)
6. The threshold to define the animal is defined by subtracting 2 \*
   the standard deviation of the smoothed image from the mean value
   of pixel intensities
7. The current image gets thresholded using the threshold defined above
8. Using this binary image, the function
   label and then the function
   regionprops of the scikit-image library
   is applied.
9. Using the *filled\_area* parameter of the regionprops function, the
   blobs are checked for minimal size defined in the
   ‘list\_of\_available\_organisms.json’ file for the animal in
   question. As soon as an image is found where a single blob is
   larger than the minimal size, the animal counts as being identified.
10. The first picture is then filtered again with a gaussian filter
    with a sigma of 1. This is defined as the background image. This
    image of course contains the animal as it was at the original
    position. For many experiments it is unlikely that the animal can
    be at exactly the same position as in the first frame. If your
    experiment makes it likely that the animal is in the exact same
    position more than once, you might want to look at Animal
    Detection Mode#2 and
    Mode#3.
11. All of this is done in the function
    `pre_experiment.FindAnimal.find_roi_mode_one_and_three()`
12. Finally, another frame is taken from the camera. *Importantly, the
    threshold is now calculated locally!* This allows for a better
    separation of the animal and the background by defining a local
    treshold. The image gets filtered using the local threshold and
    then subtracted from the first (filtered) image. After applying
    the label and then the regionprops functions of the scikit-image library the
    largest blob is defined as the animal. The centroid, the filled
    area and the bounding box are saved for the tracking algorithm.
13. Example data collected for this particular example with Animal
    Detection Mode#1:

    > **Result of Animal Detection Mode 1:** *Top Left:* Background
    > image saved for the rest of the experiment. Using Mode 1 the
    > animal will always be present in the background image used during
    > the actual experiment. If this is a problem for your experiment,
    > please check Mode#2 and
    > Mode#3.
    > *Top Right:* Animal identified during Animal Detection
    > Mode#1. The red box indicates
    > the bounding box of the animal.
    > *Bottom Left:* Close-up of the detected animal. The bounding box
    > is defined with 4 coordinates: The smallest row (Row Min, or
    > Y-min), the smallest column (Col Min, or X-min), the largest row
    > (Row Max, or Y-max) and the largest column (Col Max, or X-Max).
    > Also see the regionprops
    > function documentation (*bbox*). *Bottom Right:* The binary image
    > used by the algorithm to define the animal. The centroid is
    > indicated as well as the filled area. Both parameters are defined
    > using the regionprops function.
14. This is done using:
    `pre_experiment.FindAnimal.define_animal_mode_one()`
15. Next, the actual tracking algorithm will be called and
    run until the experiment is over.

##### Pre-Define Background - Mode 2¶

**User perspective:**

1. Prepare PiVR for the experiment by putting everything *except* the
   animal **exactly** at the position where it will be during the
   experiment. Take the optimal image parameters into account
2. Press *Start*
3. User will be asked to take a picture by clicking ‘OK’.
4. Then user will be asked to put the animal **without changing
   anything else in the Field of View of the camera**
5. Press *Start*

**Behind the scenes:**

1. The image taken without the animal is being used as the background
   image.
2. After the user puts the animal and hits ‘Ok’ the same algorithm as
   in Mode 1 and 3 is searching for the animal: First a new picture
   is taken and filtered using a gaussian filter with Sigma = 1:
3. This image is subtracted from the background image:
4. The pixel intensity values are a bimodal distribution.
5. The threshold is defined as all values larger 2 times the Standard
   deviation of the the image + the mean pixel intensity values of
   the image (usually 0)

   > **Threshold to identify animal:** On the left the background
   > pixels with a approximate value of zero are seen. Note the
   > logarithmic scale. On the right the yellow rectangle indicates
   > all the pixel intensity values that will count as the identified
   > animal
6. The threshold is used to binarize the image.
7. Using this binary image, the function label and
   then the function regionprops of the scikit-image library is applied.
8. Using the *filled\_area* parameter of the regionprops function, the
   blobs are checked for minimal size defined in the
   ‘list\_of\_available\_organisms.json’ file for the animal in question.
   As soon as an image is found where a single blob is larger than
   the minimal size, the animal counts as being identified.
9. This is done in `pre_experiment.FindAnimal.find_roi_mode_two()`
10. Unlike to Mode1 it is *not*
    necessary to take local thresholding as the animal should be
    clearly visible compared to the background. The global threshold
    is used to create a binary image. The largest blob is defined as
    the animal.
11. Example data collected for this particular example with Animal
    Detection Mode#2:

    > **Result of Animal Detection Mode 1:** *Top Left:* Background
    > image saved for the rest of the experiment. *Top Right:* Animal
    > identified during Animal Detection Mode 2. The red box indicates the bounding
    > box of the animal. *Bottom Left:* Close-up of the detected
    > animal. The bounding box is defined with 4 coordinates: The
    > smallest row (Row Min, or Y-min), the smallest column (Col Min,
    > or X-min), the largest row (Row Max, or Y-max) and the largest
    > column (Col Max, or X-Max). Also see the regionprops
    > function documentation (*bbox*). *Bottom Right:* The binary image
    > used by the algorithm to define the animal. The centroid is
    > indicated as well as the filled area. Both parameters are defined
    > using the regionprops function.
12. This is done in
    `pre_experiment.FindAnimal.define_animal_mode_two()`
13. Next, the actual tracking algorithm will
    be called and run until the experiment is over.

##### Reconstruct Background by Stitching - Mode 3¶

**User perspective:**

1. Place animal in the final dish on the arena, taking the
   optimal image parameters into account
2. Press *Start*

Behind the scenes:

1. As soon as the user hits “Start”, the camera will start streaming
   images from the camera. Here a fruit fly larva can be seen in the
   center of the image
2. A second image then taken.
3. The new image is now subtracted from previous image(s).
4. The histgroam of this image exhibits a trimodal distribution - a
   very large peak at around 0 indicating all the background pixels,
   positive pixel intensities indicating coordinates that the
   animal occupied in the past and has left and negative pixel values
   indicating coordinates that the animal has not occupied in the
   first frame but does now.
5. The threshold is defined as 4 minues the mean pixel intensity of
   the subtracted.
6. The threshold is then used to binarize the first image:
7. Using this binary image, the function
   label and then the function
   regionprops of the scikit-image library
   is applied.
8. Using the *filled\_area* parameter of the regionprops function, the
   blobs are checked for minimal size defined in the
   ‘list\_of\_available\_organisms.json’ file for the animal in
   question. As soon as an image is found where a single blob is the
   region of interest in calculated depending on the maximum speed,
   maximum size and pixel/mm.
9. So far this has been identical to
   Mode 1 - both Modes use the
   identical function up to this point:
   `pre_experiment.FindAnimal.find_roi_mode_one_and_three()`
10. Now comes the trickiest part of Mode 3: The animal must be
    identified as complete as possible. For this the region of
    interest is used:
11. The histogram of this region of interest looks very different
    compared to the histogram of the whole frame. Importantly, the
    larva now clearly stands out from the background as can be seen in
    the histogram.
12. The threshold is now adjusted until only **a single object with
    animal-like properties** is left in the thresholded region of
    interest. ^

    ^animal like properties are defined in the
    “list\_of\_available\_organisms.json” file
13. **It is important to understand the limitations of this approach!**
    This will animal identification will only work if the animal is
    clearly separated from the background. It will not work, for
    example if the animal is close to the edge of the petri dish, if
    there are other structures in the arena etc…!

    All this is done using the function:
    `pre_experiment.FindAnimal.define_animal_mode_three()`
14. After identifying the animal, the algorithm waits until the animal
    has left the inital position.

    To do this it will continue capturing images, binarizing them
    and finally subtracting them from the identfied animal. Only when
    the original animal is 99.9% reconstructed has the animal left the
    original position.

    This is done using the function
    `pre_experiment.FindAnimal.animal_left_mode_three()`
15. When the animal has left, the region that was occupied by the
    animal in the first frame is replaced by the pixels at the same
    coordinates of the image where the animal has left the original
    position. This should lead to a “clean” background image, meaning
    the animal shouldn’t be present (as it would be in Mode 1).

    This is done using the function:
    `pre_experiment.FindAnimal.background_reconstruction_mode_three()`
16. Before the tracking can start the location of the animal
    after background reconstruction must be saved. This is done using
    the function:
    `pre_experiment.FindAnimal.animal_after_box_mode_three()`

#### Animal Tracking¶

**Todo**: Go through this again with the source code in an open
window to make sure nothing important is being missed. Currently
there’s not enough explanatory text (just a lot of “look in the
source code!”) **Todo**

After the detection of the animal with any of the described animal
detections modes (Mode 1,
Mode 2 or
Mode 3) the tracking algorithm
(`fast_tracking.FastTrackingVidAlg.animal_tracking()` **FIX**)
starts.

Note

The camera will start recording at the pre-set framerate. If the
framerate exceed the time it takes for PiVR to process the frame,
the next frame will be dropped. For example, if you are recording
at 50 frames per second, each frame has to be processed in 20ms
(1/50=0.02 seconds). If the frame processing takes 21ms, the
next frame is dropped and PiVR will be idle for the next 19ms
until the next frame arrives.

1. Once the tracking algorithm starts, the camera and GPU start sending
   images at the defined framerate to the CPU, i.e. at 30fps the CPU
   will receive one new image ever 33ms. Source code here:
   `fast_tracking.FastTrackingControl.run_experiment()`
2. The images are then prepared of tracking. Source code here:
   `fast_tracking.FastTrackingVidAlg.write()`
3. Then the animal\_tracking function, which does all the heavy lifting
   described below,is called:
   `fast_tracking.FastTrackingVidAlg.animal_tracking()`.
4. The Search Box is defined depending on the previous animal
   position, the selected organism and that organisms specific
   parameters. For details check source code here:
   `start_GUI.TrackingFrame.start_experiment_function()`
5. The content of the Search Box of the current frame is then
   subtracted from the background frame (generated during
   animal detection).
6. Upon inspection of the histogram of the subtracted image it becomes
   clear that the animal has clearly different pixel intensity values
   compared to the background.
7. The treshold is determined by calculating the mean pixel intensity
   of the subtracted image and subtracting 3 times the standard
   deviation.
8. This threshold is then used to binarize the current small image:
9. Using that binary image, the function
   label
   and then the function
   regionprops
   of the scikit-image library is applied. This way all ‘blobs’ are
   identified. Using the animal parameters defined before, the blob
   that looks the most like the sought after animal is assigned to
   being the animal.

   After detection of the animal, the image of the animal is saved
   (blue rectangle) and the Search Box for the next frame prepared
   (red rectangle)

#### Head Tail Classification¶

The Head Tail classification is based upon an Hungarian algorithm.

1. First, the binary image is skeletonized (either with thin
   function
   or the skeletonize function)
2. Using the rule that the endpoints of that skeleton must only have
   one neighbour, the endpoints are defined.
3. To continue and define head and tail the following conditions must
   be met:

   1. The aspect ratio of the long axis over the short axis must be
      at least 1.25
   2. The skeleton must have exactly 2 endpoints
   3. The length of the skeleton must be larger than half the
      mean length of the previous 3 frames.
4. Next, the distance of each endpoint to a reference point is
   calculated:

   1. In case the tail has not yet been assigned (happens in the first
      frame) use the centroid of the previous frame as the reference
      point.
   2. In case of not having been able to assign a tail in the
      previous frame, e.g. due to the violation of any of the rules
      shown above, also use the centroid of the previous frame as the
      reference point.
   3. Otherwise (in most cases) the endpoint that has been
      assigned the tail in the previous frame is used as the
      reference point.
5. Whichever endpoint has the shorter distance the previous reference
   point is assigned the label ‘Tail’.

PiVR has been developed by David Tadres and Matthieu Louis (Louis Lab).

### PiVR Software Manual¶

Warning

If you have the High LED power version of PiVR you **must** take
care to properly shield yourself and others from the potentially
very strong LED light to protect eyes and skin!

Important

Several options will open a pop-up. You must close the pop-up in
order to interact with the main window.

Important

The software has different functionality if run on a Raspberry Pi
as compared to any other PC. This software manual is for the
Raspberry Pi version of the software

#### The Menubar¶

To select a different window use the Menu Bar at the top of the window

#### The Recording Menu¶

The Recording Menu lets you choose between different recording
options. There are currently 4 different methods:

> 1. Tracking – Online tracking of a single animal. Possibility of
>    delivering a time dependent stimulus.
> 2. VR Arena – Online tracking of a single animal. Present a
>    virtual arena that will define how the stimulus is present in
>    response to the position of the animal.
> 3. Full Frame Recording – Record an image sequence. Possibility
>    of delivering a time dependent stimulus.
> 4. Video – Record a video (h264 format). Possibility of
>    delivering a time dependent stimulus.

##### Camera Control Frame¶

In all of the recording options you have access to the Camera control
frame. It can be used to turn the camera preview on (Cam On) and off
(Cam Off). You can also control the size of the preview Window size.

Warning

The Camera preview is always on top of everything else of the
screen. Use the Preview Window carefully!

##### Experiment Control Frame – Tracking¶

The ‘Recording’ Option you choose is printed in Bold on top of the
Experiment Control Frame. In this example it is ‘Online Tracking’.

Online tracking tracks a **single** animal.

You have to select a folder in which the experiment will be saved by
clicking on the button to the right of ‘Save in:’

You can then give your experiment an identifier. Examples include
genotypes or an experimental treatment. This information will be
saved in your experiment folder.

If you want to present a Time Dependent Stimulus you can press the
Button ‘Select Time Dependent Stim File’. Please make sure you follow
the guidelines to learn how to prepare
the file.

The figure below gives you a quick overview over the parameters used
by the program:

1. Pixel/mm: **Essential**: This value has to be set by you
   before you run your first experiment! See set
   Pixel/mm. You must change it after changing
   resolution or adjusting height of the camera relative to the
   arena!
2. Framerate: The framerate you will be tracking the animal. See
   adjust image to see how to adjust
   the framerate.

   Warning

   There is a difference between the framerate the camera
   can deliver and the framerate the Raspberry Pi can handle. If
   you select a very high framerate you might get a
   lower framerate than expected. Always check the
   timestamps in the ‘data.csv’ if you are trying a new,
   higher framerate than before!
3. VR stim at: N/A
4. Animal Detection Mode: Either Mode 1, Mode 2 or Mode 3. See
   Select Animal Detection Mod.
5. Cam Resolution: Indicates the resolution you selected. See
   adjust image to see how to change
   the resolution.

   Important

   For Online Tracking you can only use
   640x480.
6. Animal: **Essential**: for Online Tracking. See here for
   how to select an animal. See Define new
   animal in case you are working with an
   animal which is not listed. If you are having problems detecting
   your animal see here

Next, please enter the time you want to track the animal in the field
below ‘Recording Time[s]’. Then hit ‘Start Tracking’

##### Experiment Control Frame – VR Arena¶

The ‘Recording’ Option you choose is printed in Bold on top of the
Experiment Control Frame. In this example it is ‘Closed Loop
Stimulation’.

Closed Loop Stimulation tracks a **single** animal.

You have to select a folder in which the experiment will be saved by
clicking on the button to the right of ‘Save in:’

You can then give your experiment an identifier. Examples include
genotypes or an experimental treatment. This information will be
saved in your experiment folder.

To present a virtual arena (stimulation depending on the position of
the animal) press the ‘Select VR Arena’ Button and select an arena.
Static virtual arenas are csv files. Dynamical virtual arenas are
currently npy files. To learn how to create a new arena please see
Create new VR Arena.

The box below gives you a quick overview over the parameters used by
the program:

1. Pixel/mm: **Essential**: This value has to be set by you
   before you run your first experiment! See set Pixel/mm. You must change it after changing resolution
   or adjusting height of the camera relative to the arena!
2. Framerate: The framerate you will be using to track the
   animal. See adjust image to see how
   to adjust framerate.

   Warning

   There is a difference between the framerate the camera can
   deliver and the framerate the Raspberry Pi can handle. If you
   select a very high framerate you might get a lower framerate
   than expected. Always check the timestamps in the ‘data.csv’
   if you are trying a new, higher framerate than before!
3. VR stim at: Either Head, Centroid, Midpoint or Tail. See
   here how to turn it on.
4. Animal Detection Mode: Either Mode 1, Mode 2 or Mode 3. See
   Select Animal Detection Mod.
5. Cam Resolution: Indicates the resolution you selected. See
   adjust image to see how to change
   the resolution.

   Important

   For Closed Loop Experiments you can only use
   640x480.
6. Animal: **Essential**: for Closed Loop Experiments. See here for how to
   select an animal. See Define new
   animal in case you are working with an
   animal which is not listed. If you are having problems detecting
   your animal see here

Next, please enter the time you want to track the animal in the field
below ‘Recording Time[s]’. Then hit ‘Start Tracking VR’

##### Experiment Control Frame – Full Frame Recording¶

The ‘Recording’ Option you choose is printed in Bold on top of the
Experiment Control Frame. In this example it is ‘Image Sequence’.

Image Sequence just records still images without tracking anything.
The advantage over video is that no compression of the image data is
done. The disadvantage is that it is limited by the time it takes the
Raspberry Pi to write the file on the SD card. If you are using a
higher quality SD card, you will be able to write at a higher the
framerate. However, it will probably always be lower than video.

You have to select a folder in which the experiment will be saved by
clicking on the button to the right of ‘Save in:’

You can then give your experiment an identifier. Examples include
genotypes or an experimental treatment. This information will be
saved in your experiment folder.

If you want to present a Time Dependent Stimulus you can press the
Button ‘Select Time Dependent Stim File’. Please make sure you follow
the guidelines to learn how to prepare
the file.

The box below gives you a quick overview over the parameters used by
the program:

1. Pixel/mm: This value indicates how many pixels are in one mm.
   You will need this value to be correct to calculate anything
   with distance afterwards (speed, distance to source etc.) See
   set Pixel/mm. You must change it after
   changing resolution or adjusting height of the camera
   relative to the arena!
2. Framerate: The framerate you will be tracking the animal. See
   adjust image to see how to adjust
   framerate.

   Warning

   There is a difference between the framerate the camera
   can deliver and the framerate the Raspberry Pi can handle.
   If you select a very high framerate you might get a lower
   framerate than expected. Always check the timestamps in
   the ‘data.csv’ if you are trying a new, higher framerate
   than before!
3. VR stim at: N/A
4. Animal Detection Mode: NA.
5. Cam Resolution: Indicates the resolution you selected. See
   adjust image to see how to change
   the resolution.
6. Animal: Value that will be saved in ‘experiment\_settings.json’.

Select the image format you want your images to be in: jpg, png, rbg,
yuv or rgba. See here for details
on the different formats.

Next, please enter the time you want to track the animal in the field
below ‘Recording Time[s]’.

Then hit ‘Start Recording Images

##### Experiment Control Frame – Video¶

The ‘Recording’ Option you choose is printed in Bold on top of the
Experiment Control Frame. In this example it is ‘Video’.

As the name indicates, use this option to record videos. The
advantage of this method over image sequence is it’s superior speed.
The disadvantage, especially for scientific questions, might be that
it compresses the image file in the temporal domain. See here
for an introduction and the Wikipedia page for more details.

You have to select a folder in which the experiment will be saved by
clicking on the button to the right of ‘Save in:’

You can then give your experiment an identifier. Examples include
genotypes or an experimental treatment. This information will be
saved in your experiment folder.

If you want to present a Time Dependent Stimulus you can press the
Button ‘Select Time Dependent Stim File’. Please make sure you follow
the guidelines to learn how to prepare
the file.

The box below gives you a quick overview over the parameters used by
the program:

1. Pixel/mm: This value indicates how many pixels are in one mm.
   You will need this value to be correct to calculate anything
   with distance afterwards (speed, distance to source etc.) See
   set Pixel/mm. You must change it after
   changing resolution or adjusting height of the camera
   relative to the arena!
2. Framerate: The framerate you will be tracking the animal. See
   adjust image to see how to adjust
   the framerate.

   Warning

   There is a difference between the framerate the camera
   can deliver and the framerate the Raspberry Pi can handle.
   If you select a very high framerate you might get a lower
   framerate than expected. Always check the timestamps in
   the ‘data.csv’ if you are trying a new, higher framerate
   than before!
3. VR stim at: N/A
4. Animal Detection Mode: NA.
5. Cam Resolution: Indicates the resolution you selected. See
   adjust image to see how
   to change the resolution.

   Important

   For video you cannot use 2592x1944.
6. Animal: Value that will be saved in ‘experiment\_settings.json’.

Next, please enter the time you want to track the animal in the field
below ‘Recording Time[s]’. Then hit ‘Start Recording Images

#### Preparing a Time Dependent Stimulus File¶

In your PiVR folder you can find a folder called
‘time\_dependent\_stim’. On a fresh install it is supposed to contain a
single file: blueprint\_stim\_file.csv

When you open it with, e.g. excel or your csv viewing program of
choice you’ll see that there are 5 columns and many rows:

The first Column (A) is the frame number. E.g. if you are recording at
30 frames per second the row 2-32 will define what’s going on in
that time.

The second column defines what Channel 1 is doing at a given frame. 0
means the light is completely OFF. 100 means the light is completely
ON. A number in between, e.g. 50 means that the light is on at
50/100=50%

The third (Channel 2), the fourth (Channel 3) and the fifth (Channel
4) use the same principle for the other channels.

It is important to notice that the stimulation file needs to be
defined on a very low level: Frame Number. The same stimulus file
will give different stimulations depending on the framerate. Therefore:

> 1. Decide on a framerate for you experiment, as an example we’ll
>    say you decide on 30fps
> 2. Decide on a length of your experiment, for example 20 seconds
> 3. Decide on the stimulation pattern, e.g. you want Channel 1 to
>    be OFF for the first second and
>    Channel 2 to be ON for the first second. Then you want to
>    switch, Channel 1 is ON for 1 sec, Channel 2 is OFF for 1 sec
> 4. You will need to set the first 30 (framerate \* length of
>    stimulus) rows of Channel 1 to 0
> 5. And you will need to set the first 30 (framerate \* length of
>    stimulus) rows of Channel 2 to 100
> 6. As you don’t care about Channel 3 and 4 you can leave it at zero
> 7. At row # 2 (since you start at row #2 in excel) or frame #
>    30 (first column) you set Channel 1 to 100 for 30 rows
>    (framerate \* length of stimulus) to turn it ON and Channel 2
>    to 0 to turn it OFF

Notes:
:   1. If you do not define enough rows for your experiment, e.g. if
       you want to run the 20 seconds experiment at 30frames per
       second but you only define what happens during the first 15
       seconds (by only going to row 15\*30=450 instead of row
       20\*30=600) the last value for each channel will be
       propagated, e.g. if row 450 is set to 100 and row 451 to
       600 are not defined the value 100 will be used for the rest
       of the experiment.
    2. If you define more rows than you need for your experiments
       only the stimulation up to the point you record are used
       (this will behave as you probably expect)

#### Set Pixel/mm¶

In order to set Pixel/mm for your resolution, press the ‘Options’
Menu in the Menu Bar. Then select ‘Define Pixel/mm’

In the popup window you will see features:

> 1. The resolution you are currently using. The defined value
>    will only be valid for this resolution
> 2. The Left and right cutoff slider. By moving them you can
>    measure the distance.
> 3. A slice of the image taken by the camera. You want to put
>    something you can measure horizontally before the camera.
> 4. A text field to enter a length you want to measure.

Below an example of an adjusted distance configuration window.
Once you are satisfied with the adjustments you’ve made hit the quit
button.

#### Adjust image¶

In order to set any options related to the image, press the ‘Options’
Menu in the Menu Bar. Then select ‘Optimize Image’.

This popup should being used to set up the image in the optimal way:

> 1. Turn the camera on (‘Cam On’) if it’s not on already
> 2. Adjust the preview size so that you can comfortably see both
>    the preview and the popup.
> 3. Set the framerate as desired.
> 4. Press the ‘Update Preview Framerate’ button
> 5. Set the resolution you’d like to use for the recording.
>
>    Important
>
>    For Online Tracking and
>    Closed Loop Experiments only 640x480
>    is possible. For video you cannot
>    use 2592x1944.
> 6. Make sure the autoexposure button says ‘autoexp on’.
> 7. Turn the Backlight Intensity up. It is normal to only see
>    something above 150’000. 400’000-500’000
>    is often a good value to choose.
> 8. If you have Backlight 2 intensity on one of the GPIOs (see
>    define GPIO output channels) you can
>    also adjust Backlight 2 intensity at this point.
> 9. To test your output channels, slide the appropriate slider to
>    the right. At the beginning of any experiments these will be
>    turn off again. To keep a stimulus ON for the duration of the
>    experiment use the Backlight 2 intensity.

##### Set up optimal image¶

In order to set up optimal image parameters I usually do the following:

> 1. Turn ‘Cam On’
> 2. Set ‘autoexp on’
> 3. Pull ‘Backlight Intensity’ slider all the way to the left
>    (Image will be dark)
> 4. Now pull the ‘Backlight Intensity’ slider to the right. As
>    soon as I see an image in the camera I go another 100‘000 to
>    the right - this way I’m not at the lower detection limit of the camera.
> 5. Then I turn ‘autoexp off’
> 6. Often it can improve the image if I pull the ‘Backlight
>    Intensity’ slider a bit more to the right, effectively
>    overexposing the image a bit.

#### Define GPIO output channels¶

In order to define GPIO output channels for your resolution, press
the ‘Options’ Menu in the Menu Bar. Then select ‘define GPIO output
channels’.

The images on the far left indicate which of the outputs on the left
of your setups are which GPIO (e.g. the one closest to the LED power
input is GPIO#18).

There are 4 GPIO’s that can be used to control LEDs: GPIO#18,
GPIO#17, GPIO#27 and GPIO#13. GPIO#18 and
GPIO#13 are special as they are the only ones that are capable of
using PWM frequencies above the kilohertz range.

Channel 1 is always defined as the channel that is used for the
Virtual Arena experiments.

Channel 1, Channel 2, Channel 3 and Channel 4 can be seperately
adressed using the time dependent stimulus files.

#### Turn Debug Mode ON/OFF¶

In order Turn Debug Mode On or Off press ‘Options’ Menu in the Menu
Bar. Then go on ‘Turn Debug Mode…’ and select either ‘OFF’ or ‘ON’.

#### Select Animal Detection Mode¶

In order Define the Animal Detection Method press ‘Options’ Menu in
the Menu Bar. Then press ‘Animal Detection Method’.

When in either ‘Online Tracking’ or ‘Closed Loop Stimulation the
animal needs to be detected. There are 3 modes that can be used to
detect the animal. For most cases Mode 1 (Standard) will be fine. If
you need a clear background image consider Mode 2 or Mode 3.

#### Select Organism¶

In order select an organism press ‘Options’ Menu in the Menu Bar.
Then go on ‘Select Animal’ and select your animal.

#### Updating the software¶

In order to update the software on your RaspberryPi, press the ‘File’
Menu in the Menu Bar. Then go on ‘Update Software’.

Note

Please make sure you are connected to the Internet when updating.

Technicalities:

This will first update our Linux by calling:

```
sudo update
```

Next, it will download the newest version from the gitlab repository by calling:

```
git pull
```

#### High/Low Power LED switch¶

In order to choose between High and low Power LED setups press
‘Options’ Menu in the Menu Bar. Then go on ‘High Power LEDs’.

Select either Standard or High power version depending on the setup
you have.

#### Select Body Part for VR stimulation¶

When running virtual reality experiments the cells you are interested
in could be at different places of the animal.

PiVR allows you to present the virtual reality depending on
different body parts identified during
tracking.

You may choose different body parts that are defined during tracking.

Note

As the difference between *centroid* and *midpoint* is not
straightforward, please see here for an
explanation.

1. The Head (standard) will probably make a lot of sense in many
   experiments as a lot of sensory neurons of many animals are
   located there. However be aware that the Head/Tail classification
   algorithm is not perfect and does make mistakes. There is no
   option to correct for wrong head/tail assignment during the
   experiment!
2. The Centroid is probably the most consistently correct point
   during tracking. Please see
   here to see how it is defined.
3. The Midpoint is similar to the centroid, but can be different in
   flexible animals such as fruit fly larvae.
4. The tail is is the final option to choose from. We have used the
   presentation of the virtual reality based on tail position as a
   control in the past.

#### Animal Color Selection¶

Depending on your experimental setup, the animal can either be *dark
on white* background due to transillumination, or *white on dark*
background due to side illumination.

The standard setting is *dark on white*. If you need to change this
setting, go to Options->

Now just press the button above the image that describes your
experiment.

PiVR has been developed by David Tadres and Matthieu Louis (Louis Lab).

### Explanation of PiVR output¶

#### Tracking¶

After running a tracking experiment you will
find a folder with the “DATE\_TIME\_EXP.GROUP” as its name. An example
would be “2019.01.11\_14-00-05\_CantonS”. This is an experiment
conducted on the 11th of January 2019. “CantonS” is the value that
was entered in the field “Exp. Group”.

This folder will contain the following files:

##### “DATE\_TIME\_data.csv”¶

is probably the most important file. It contains the following data
for each frame of the experiment:

> 1. The frame (=image) number into the experiment
> 2. The time in seonds since the experiment started
> 3. The X (column) coordinate of the Centroid (Check
>    here for comparison with midpoint)
> 4. The Y (row) coordinate of the Centroid
> 5. The X (column) coordinate of the head
> 6. The Y (row) coordinate of the head
> 7. The X (column) coordinate of the tail
> 8. The Y (row) coordinate of the tail
> 9. The X (column) coordinate of the midpoint (Check
>    here for comparison with centroid)
> 10. The Y (row) coordinate of the midpoint

##### “Background.jpg”¶

contains the reconstructed background image. See
here for explanation
where it is coming from and what it means.

##### “bounding\_boxes.npy”¶

is a Numpy file. It contains the coordinates
of the bounding box of the small image. The bounding box defines the
Y/X coordinates of the small image

This file comes in
shape
[4, # of frames] with:

|  |  |
| --- | --- |
| [0, :] | contains the Y\_min values |
| [1, :] | contains the Y\_max values |
| [2, :] | contains the X\_min values |
| [3, :] | contains the X\_max values |

These values are necessary to describe where in the full image
frame the small image that has been saved during the experiment is
located. The bounding box is the rectangle that contains all image
information used during this frame. Below an illustration on how
the different values are used to construct the bounding box.

Note

Why Y/X and not X/Y? In image processing the convention is to
reference points in (Rows, Columns) which translates to Y/X. The
underlying image processing libraries work with the (Rows,
Columns) convention. See for example here.
PiVR therefore follows this convention.

##### “centroids.npy”¶

is a Numpy file. It contains the coordinates
of the centroid of the blob identified during the experiment.
See here to see the centroid compared to the
midpoint.

The file comes in shape
[# of frames, 2] with:

|  |  |
| --- | --- |
| [:, 0] | contains the centroid Y values |
| [:, 1] | contains the centroid X values |

These values are identical to what you will find in the
“DATE\_TIME\_data.csv” file

##### “midpoints.npy”¶

is a Numpy file. It contains the coordinates
of the midpoint extracted from the skeleton during the experiment.
See here to see the midpoint compared to the
centroid.

The file comes in shape
[# of frames, 2] with:

|  |  |
| --- | --- |
| [:, 0] | contains the midpoint Y values |
| [:, 1] | contains the midpoint X values |

These values are identical to what you will find in the
“DATE\_TIME\_data.csv” file

##### “heads.npy”¶

is a Numpy file. It contains the coordinates
of the head position assigned during tracking.

The file comes in shape
[# of frames, 2] with:

|  |  |
| --- | --- |
| [:, 0] | contains the head Y values |
| [:, 1] | contains the head X values |

These values are identical to what you will find in the
“DATE\_TIME\_data.csv” file

##### “tails.npy”¶

is a Numpy file. It contains the coordinates
of the tail position assigned during tracking.

The file comes in shape
[# of frames, 2] with:

|  |  |
| --- | --- |
| [:, 0] | contains the tail Y values |
| [:, 1] | contains the tail X values |

These values are identical to what you will find in the
“DATE\_TIME\_data.csv” file

##### “experiment\_settings.json”¶

is a json file and contains a
lot of useful experimental information:

1. Search box size: The Search box used to locate the animal during
   the experiment
2. Exp. Group: The string that was entered by the user during the
   experiment
3. Experiment Date and Time: exactly that
4. Framerate: The frequency at which PiVR tracked the animal
5. Model Organism: While tracking, PiVR used the parameters of this
   animal to optimize tracking. See **Todo** here for how to modify
   this parameter.
6. Pixel per mm: For PiVR to be able to track the animal, it needs
   to know how many pixels indicate one mm. This has been set by the
   user as described here.
7. Recording time: The time in seconds that PiVR was tracking the
   animal
8. Resolution: The camera resolution in pixel that PiVR used while
   tracking. Currently only 640x480 is possible.
9. Time delay due to Animal Detection[s]: For the
   autodetection the animal
   must move. The time it taook between pressing “start” and successful
   animal detection is saved here.
10. Virtual Reality arena name: As no virtual arena was presented,
    it will say ‘None’
11. backlight 2 channel: If Backlight 2 has been defined (as
    described here) the chosen GPIO (e.g. 18)
    and the maximal PWM frequency (e.g. 40000) is saved as a [list].
12. backlight channel: If Backlight 1 has been defined (as
    described here) the chosen GPIO (e.g. 18)
    and the maximal PWM frequency (e.g. 40000) is saved as a [list].
    This would normally be defined as [18, 40000].
13. output channel 1: If Channel 1 has been defined (as
    described here) the chosen GPIO (e.g. 17)
    and the maximal PWM frequency (e.g. 40000) is saved as a [list].
14. output channel 2: If Channel 2 has been defined (as
    described here) the chosen GPIO (e.g. 27)
    and the maximal PWM frequency (e.g. 40000) is saved as a [list].
15. output channel 3: If Channel 3 has been defined (as
    described here) the chosen GPIO (e.g. 13)
    and the maximal PWM frequency (e.g. 40000) is saved as a [list].
16. output channel 4: If Channel 4 has been defined (as
    described here) the chosen GPIO (e.g. 13)
    and the maximal PWM frequency (e.g. 40000) is saved as a [list].

##### “first\_frame\_data.json”¶

is a json file and contains
information that collected during
animal detection (Source
code `pre_experiment.FindAnimal`.)

1. bounding box col max: The X\_max value of the bounding box of the
   animal detected in the first frame during animal detection.
2. bounding box col min: The X\_min value of the bounding box of the
   animal detected in the first frame during animal detection.
3. bounding box row max: The Y\_min value of the bounding box of the
   animal detected in the first frame during animal detection.
4. bounding box row min: The Y\_max value of the bounding box of the
   animal detected in the first frame during animal detection.
5. centroid col: The X value of the centroid of the animal detected
   in the first frame during animal detection.
6. centroid row: The Y value of the centroid of the animal detected
   in the first frame during animal detection.
7. filled area: The filled area in pixels of the blob defined as
   the animal in the first frame during animal detection

#### VR Arena and Dynamic VR Arena¶

After running a VR Arena experiment you will
find a folder with the “DATE\_TIME\_EXP.GROUP” as its name. An example
would be “2019.01.11\_14-00-05\_CantonS”. This is an experiment
conducted on the 11th of January 2019. “CantonS” is the value that
was entered in the field “Exp. Group”.

This folder will contain the following files:

##### “DATE\_TIME\_data.csv”¶

is probably the most important file. It contains the following data
for each frame of the experiment:

> 1. The frame (=image) number into the experiment
> 2. The time since the experiment started
> 3. The X (column) coordinate of the Centroid (Check
>    here for comparison with midpoint)
> 4. The Y (row) coordinate of the Centroid
> 5. The X (column) coordinate of the head
> 6. The Y (row) coordinate of the head
> 7. The X (column) coordinate of the tail
> 8. The Y (row) coordinate of the tail
> 9. The X (column) coordinate of the midpoint (Check
>    here for comparison with centroid)
> 10. The Y (row) coordinate of the midpoint
> 11. The stimulus (in PWM dutycycle **todo** decide on 100% or
>     40000) delivered.

##### “RESOLUTION\_NAME.csv”¶

for example “640x480\_checkerboard.csv”. This is the virtual arena
presented to the animal. In case the virtual arena is positioned
relative to the starting position and the movement of the animal
(such as the “640x480\_gaussian\_centred\_animal\_pos[250,240,0.0].csv”
arena), this file will *final* translated and rotated arena as it was
presented to the animal.

Note

If a dynamic virtual reality has been presented, this file will
not be present - it would simply take too long and take up too
much space. This is one reason why dynamic virtual realities can
not be translated and rotated at the moment.

##### “stimulation.npy”¶

is a Numpy file. It contains the stimulus
delivered to the animal during the experiment.

##### “Background.jpg”¶

contains the reconstructed background image. See
here for explanation
where it is coming from and what it means.

##### “bounding\_boxes.npy”¶

is a Numpy file. It contains the coordinates
of the bounding box of the small image. The bounding box defines the
Y/X coordinates of the small image

This file comes in
shape
[4, # of frames] with:

|  |  |
| --- | --- |
| [0, :] | contains the Y\_min values |
| [1, :] | contains the Y\_max values |
| [2, :] | contains the X\_min values |
| [3, :] | contains the X\_max values |

These values are necessary to describe where in the full image
frame the small image that has been saved during the experiment is
located. The bounding box is the rectangle that contains all image
information used during this frame. Below an illustration on how
the different values are used to construct the bounding box.

Note

Why Y/X and not X/Y? In image processing the convention is to
reference points in (Rows, Columns) which translates to Y/X. The
underlying image processing libraries work with the (Rows,
Columns) convention. See for example here.
PiVR therefore follows this convention.

##### “centroids.npy”¶

is a Numpy file. It contains the coordinates
of the centroid of the blob identified during the experiment.
See here to see the centroid compared to the
midpoint.

The file comes in shape
[# of frames, 2] with:

|  |  |
| --- | --- |
| [:, 0] | contains the centroid Y values |
| [:, 1] | contains the centroid X values |

These values are identical to what you will find in the
“DATE\_TIME\_data.csv” file

##### “midpoints.npy”¶

is a Numpy file. It contains the coordinates
of the midpoint extracted from the skeleton during the experiment.
See here to see the midpoint compared to the
centroid.

The file comes in shape
[# of frames, 2] with:

|  |  |
| --- | --- |
| [:, 0] | contains the midpoint Y values |
| [:, 1] | contains the midpoint X values |

These values are identical to what you will find in the
“DATE\_TIME\_data.csv” file

##### “heads.npy”¶

is a Numpy file. It contains the coordinates
of the head position assigned during tracking.

The file comes in shape
[# of frames, 2] with:

|  |  |
| --- | --- |
| [:, 0] | contains the head Y values |
| [:, 1] | contains the head X values |

These values are identical to what you will find in the
“DATE\_TIME\_data.csv” file

##### “tails.npy”¶

is a Numpy file. It contains the coordinates
of the tail position assigned during tracking.

The file comes in shape
[# of frames, 2] with:

|  |  |
| --- | --- |
| [:, 0] | contains the tail Y values |
| [:, 1] | contains the tail X values |

These values are identical to what you will find in the
“DATE\_TIME\_data.csv” file

##### “experiment\_settings.json”¶

is a json file and contains a
lot of useful experimental information:

1. Search box size: The Search box used to locate the animal during
   the experiment
2. Exp. Group: The string that was entered by the user during the
   experiment
3. Experiment Date and Time: exactly that
4. Framerate: The frequency at which PiVR tracked the animal
5. Model Organism: While tracking, PiVR used the parameters of this
   animal to optimize tracking. See **Todo** here for how to modify
   this parameter.
6. Pixel per mm: For PiVR to be able to track the animal, it needs
   to know how many pixels indicate one mm. This has been set by the
   user as described here.
7. Recording time: The time in seconds that PiVR was tracking the
   animal
8. Resolution: The camera resolution in pixel that PiVR used while
   tracking. Currently only 640x480 is possible.
9. Time delay due to Animal Detection[s]: For the
   autodetection the animal
   must move. The time it taook between pressing “start” and successful
   animal detection is saved here.
10. Virtual Reality arena name: As no virtual arena was presented,
    it will say ‘None’
11. backlight 2 channel: If Backlight 2 has been defined (as
    described here) the chosen GPIO (e.g. 18)
    and the maximal PWM frequency (e.g. 40000) is saved as a [list].
12. backlight channel: If Backlight 1 has been defined (as
    described here) the chosen GPIO (e.g. 18)
    and the maximal PWM frequency (e.g. 40000) is saved as a [list].
    This would normally be defined as [18, 40000].
13. output channel 1: If Channel 1 has been defined (as
    described here) the chosen GPIO (e.g. 17)
    and the maximal PWM frequency (e.g. 40000) is saved as a [list].
14. output channel 2: If Channel 2 has been defined (as
    described here) the chosen GPIO (e.g. 27)
    and the maximal PWM frequency (e.g. 40000) is saved as a [list].
15. output channel 3: If Channel 3 has been defined (as
    described here) the chosen GPIO (e.g. 13)
    and the maximal PWM frequency (e.g. 40000) is saved as a [list].
16. output channel 4: If Channel 4 has been defined (as
    described here) the chosen GPIO (e.g. 13)
    and the maximal PWM frequency (e.g. 40000) is saved as a [list].

##### “first\_frame\_data.json”¶

is a json file and contains
information that collected during
animal detection (Source
code `pre_experiment.FindAnimal`.)

1. bounding box col max: The X\_max value of the bounding box of the
   animal detected in the first frame during animal detection.
2. bounding box col min: The X\_min value of the bounding box of the
   animal detected in the first frame during animal detection.
3. bounding box row max: The Y\_min value of the bounding box of the
   animal detected in the first frame during animal detection.
4. bounding box row min: The Y\_max value of the bounding box of the
   animal detected in the first frame during animal detection.
5. centroid col: The X value of the centroid of the animal detected
   in the first frame during animal detection.
6. centroid row: The Y value of the centroid of the animal detected
   in the first frame during animal detection.
7. filled area: The filled area in pixels of the blob defined as
   the animal in the first frame during animal detection

##### “DATE\_TIME\_data.csv”¶

contains the following data for each frame of the video:

> 1. Frame (=image) number into the experiment
> 2. Time in seconds since the experiment started
> 3. Channel 1 stimulus delivered
> 4. Channel 2 stimulus delivered
> 5. Channel 3 stimulus delivered
> 6. Channel 4 stimulus delivered

#### Full Frame Recording¶

After taking a lot of images with
Full Frame Recording, find a folder with the
“DATE\_TIME\_EXP.GROUP” as its name. An example would be
“2019.01.11\_14-00-05\_CantonS”. This is an experiment conducted on the
11th of January 2019. “CantonS” is the value that was entered in the field
“Exp. Group”.

##### Image files¶

Usually lots upon lots of them. Each image is saved separately directly
into this folder.

##### “experiment\_settings.json”¶

is a json file and contains a
lot of useful experimental information:

1. Experiment Date and Time: Exactly as advertised
2. Framerate: The framerate the video was recorded in
3. Exp. Group: The string that was entered by the user during the
   experiment
4. Model Organism: If selected, what animal has been indicated
   during the experiment.
5. Pixel per mm: If defined (see here) a useful
   parameter for analysis.
6. Recording time: The time in seconds that PiVR was recording
   this video.
7. ResolutionL The camera resolution in pixel that PiVR used while
   recording the video.
8. Virtual Reality arena name: As no virtual arena was presented,
   it will say ‘None’
9. backlight 2 channel: If Backlight 2 has been defined (as
   described here) the chosen GPIO (e.g. 18)
   and the maximal PWM frequency (e.g. 40000) is saved as a [list].
10. backlight channel: If Backlight 1 has been defined (as
    described here) the chosen GPIO (e.g. 18)
    and the maximal PWM frequency (e.g. 40000) is saved as a [list].
    This would normally be defined as [18, 40000].
11. output channel 1: If Channel 1 has been defined (as
    described here) the chosen GPIO (e.g. 17)
    and the maximal PWM frequency (e.g. 40000) is saved as a [list].
12. output channel 2: If Channel 2 has been defined (as
    described here) the chosen GPIO (e.g. 27)
    and the maximal PWM frequency (e.g. 40000) is saved as a [list].
13. output channel 3: If Channel 3 has been defined (as
    described here) the chosen GPIO (e.g. 13)
    and the maximal PWM frequency (e.g. 40000) is saved as a [list].
14. output channel 4: If Channel 4 has been defined (as
    described here) the chosen GPIO (e.g. 13)
    and the maximal PWM frequency (e.g. 40000) is saved as a [list].

#### Video¶

After recording a video, you will
find a folder with the “DATE\_TIME\_EXP.GROUP” as its name. An example
would be “2019.01.11\_14-00-05\_CantonS”. This is an experiment
conducted on the 11th of January 2019. “CantonS” is the value that
was entered in the field “Exp. Group”.

##### “EXPGRP\_VIDEO.h264”¶

the video file. This video file on its own is not perfectly useful
(at least in my hands) as h264 seems to be a bit of an exotic file
format that many video players can not handle without problems.

In order to directly convert this file check out the script
“convert\_h264\_to\_AVI.py” at https://gitlab.com/davidtadres/pivr\_bonus

Note

I have tried to directly convert the image using ffmpeg. I believe
there is a bug somewhere in the encoder of the camera as ffmpeg
reads that the video is “inf” long. The scripts above take the
video metadata from “experiment\_settings.json” to properly convert
the video.

The standard lens introduces a lot of radial aberrations at the
edges! To fix them have a look at the “CameraCalibrations” repository
I use to correct them:
https://gitlab.com/davidtadres/cameracalibrations

##### “DATE\_TIME\_data.csv”¶

contains the following data for each frame of the video:

> 1. Frame (=image) number into the experiment
> 2. Time in seconds since the experiment started
> 3. Channel 1 stimulus delivered
> 4. Channel 2 stimulus delivered
> 5. Channel 3 stimulus delivered
> 6. Channel 4 stimulus delivered

##### “experiment\_settings.json”¶

is a json file and contains a
lot of useful experimental information:

1. Experiment Date and Time: Exactly as advertised
2. Framerate: The framerate the video was recorded in
3. Exp. Group: The string that was entered by the user during the
   experiment
4. Model Organism: If selected, what animal has been indicated
   during the experiment.
5. Pixel per mm: If defined (see here) a useful
   parameter for analysis.
6. Recording time: The time in seconds that PiVR was recording
   this video.
7. ResolutionL The camera resolution in pixel that PiVR used while
   recording the video.
8. Virtual Reality arena name: As no virtual arena was presented,
   it will say ‘None’
9. backlight 2 channel: If Backlight 2 has been defined (as
   described here) the chosen GPIO (e.g. 18)
   and the maximal PWM frequency (e.g. 40000) is saved as a [list].
10. backlight channel: If Backlight 1 has been defined (as
    described here) the chosen GPIO (e.g. 18)
    and the maximal PWM frequency (e.g. 40000) is saved as a [list].
    This would normally be defined as [18, 40000].
11. output channel 1: If Channel 1 has been defined (as
    described here) the chosen GPIO (e.g. 17)
    and the maximal PWM frequency (e.g. 40000) is saved as a [list].
12. output channel 2: If Channel 2 has been defined (as
    described here) the chosen GPIO (e.g. 27)
    and the maximal PWM frequency (e.g. 40000) is saved as a [list].
13. output channel 3: If Channel 3 has been defined (as
    described here) the chosen GPIO (e.g. 13)
    and the maximal PWM frequency (e.g. 40000) is saved as a [list].
14. output channel 4: If Channel 4 has been defined (as
    described here) the chosen GPIO (e.g. 13)
    and the maximal PWM frequency (e.g. 40000) is saved as a [list].

#### Get started¶

Different experiments necessitate different analysis. In the original
PiVR publication **link** a number of different experiments were run
and the analysis **pivrpublication link** and data of those has been
made public **DRYAD LINK**. These scripts are all annotated and you
should be able to run them on your computer with the original data
to understand what is happening in them. Then you can adapt them/use
them with your data.

In addition, the PiVR software has a couple of built in analysis
tools when run on a PC (i.e. not on a Raspberry Pi):

#### Visualization of different points on the animal¶

What exactly do the terms “Centroid” and “Midpoint” mean? I will try
to illustrate the difference so that you may choose the appropriate
parameter for your experiment:

1. To identify the animal the tracking algorithm identifies a “blob”
   that has significantly different pixel intensity values compared
   to the background.
2. The centroid is the center of mass (in 2D) of these pixels.
3. The midpoint is the center of the skeletonized blob.

PiVR has been developed by David Tadres and Matthieu Louis (Louis Lab).

### Tools¶

Important

The software has different functionality if run on a Raspberry Pi
as compared to any other PC. This manual is for the PC (Windows,
MacOS and Linux if *not* run on a Raspberry Pi) version of the
software

#### The Menubar¶

To select a different window use the Menu Bar at the top of the window

#### The Analysis Menu¶

The Analysis Menu lets you choose a folder (either with one
experiment or a folder containing several experiments) and run
different analyses.

To analyze an experiment (or several) first press the “Press to select
data to analyze” button on the top left. Select a folder. You may:

1. Select a single experiment. In that case you **must** uncheck the
   button on the right next to “More than one folder”.
2. Select a folder containing several experiments. If this folder
   only contains folders you want to apply the same analysis, you do
   not have to do anything. If there are other files or folders,
   please indicate a commonality between all the folders in the entry
   field below. This can be the date (e.g. 2019) a genotype (e.g.
   Or42a) or..

   The “Number of files” below the entry field indicates how many
   files and folders in the folder you selected is taken into account
   if the current input is used.

Next, select what analysis you want to do. Currently there are several
options:

##### Distance to source¶

If you are interested in the distance the animal has to a point in
its environment try this option.

We used it in our publication to produce Figure 2C (**TODO link**): A
fruit fly larva was behaving in an environment with a single odor
source. We were interested in the distance to this odor source during
the experiment.

To use this analysis program, you must have used the
Tracking tab on PiVR **or** you must have
recorded a Video or taken
Full Frame Images with subsequent
Post-Hoc Single Animal Analysis.

Important

If you want to use the multiple experiment options, please ensure
that you have **identical** Framerates and **identical** Recording
length

After selecting a folder and pressing “Press to start analysis” a
popup will emerge that will ask you to “Select a source”. Do the
following:

1. Press on the button that says “Select Source”
2. Click on the image where the “Source” is. In our case this would
   be an odor source roughly in the center of the dish. In your case
   it could be any *single* point
3. Once you are satisfied with the arrow placement, press “Analyze”

Once the analysis has finished you will find:

1. “distance\_to\_source.csv” in each experimental folder. This
   csv file contains the calculated distance to source in mm for each
   frame.
2. “Distance\_to\_source.png” in each experimental folder. This plot is
   intended to give a quick overview about the distance to source of
   this experiment.

If you have analyzed multiple experiments you will also find:

1. “all\_distance\_to\_source.csv” in the parental folder. This file
   contains the calculated distance to source in mm for each frame
   (rows) for all the analyzed experiments (columns)
2. “Median\_Distance\_to\_source.png” in the parental folder. This plot
   is intended to give a quick overview about the distance to source
   of all the analyzed experiments. Individual experiments are
   plotted in light grey and the median in red.

See here for actual code:
`analysis_scripts.AnalysisDistanceToSource`

##### Distance to source, VR¶

If you ran an virtual reality experiment with a single maximum point
and you wish to calculate the distance to that point, try this
option.

We used this analysis in our publication to produce Figure 2D (**TODO
link**): A fruit fly larva was behaving in an virtual
odor reality. We were interested in the distance to this virtual odor
source during the experiment.

To use this analysis program, you must have used the
Virtual Reality tab on PiVR.

Important

If you want to use the multiple experiment options, please ensure
that you have **identical** Framerates and **identical** Recording
length

After selecting a folder and pressing “Press to start analysis” the
script will automatically read the virtual reality arena presented
during the experiment and calculate the distance of the animal to the
maximum point of stimulus intensity in the virtual reality.

Once the analysis has finished you will find:

1. “distance\_to\_VR\_max.csv” in each experimental folder. This
   csv file contains the calculated distance to the maximum point of
   stimulus intensity in the virtual reality in mm for each frame.
2. “Distance\_to\_VR\_max.png” in each experimental folder. This plot is
   intended to give a quick overview about the distance to source of
   this experiment.

If you have analyzed multiple experiments you will also find:

1. “all\_distance\_to\_VR\_max.csv” in the parental folder. This file
   contains the calculated distance to the maximum point of stimulus
   intensity in the virtual reality in mm for each frame
   (rows) for all the analyzed experiments (columns)
2. “Median\_Distance\_to\_VR\_max.png” in the parental folder. This plot
   is intended to give a quick overview about the distance to source
   of all the analyzed experiments. Individual experiments are
   plotted in light grey and the median in red.

See here for actual code:
`analysis_scripts.AnalysisVRDistanceToSource`

##### Single animal tracking (post-hoc)¶

If you have recorded image sequences or videos of single animals
behaving and you wish to track their position, try this option.

While it works best if the PiVR
Full Frame Recording or the PiVR
Video recording option was used, the analysis
program should be able to handle other image sequences and video files
as well.

Important

Make sure that **one experiment/trial is in one folder**. For
example, if you have recorded three videos, *‘Video\_1.mp4’*,
*‘Video\_2.mp4’* and *‘Video\_3.mp4’* each video **must** be in its own
folder (e.g. *‘Video\_1.mp4’* goes into *‘Folder\_1’*, *‘Video\_2.mp4’*
goes into *‘Folder\_2’* etc.),for the analysis software to work.

Once you press the ‘Press to start analysis’ button, the software
will check whether it can find metadata in order to perform the
tracking. If it does not find it, it will ask for user input.
Specifically, it needs:

1. The framerate the video/image sequence was recorded in.
2. How many pixel are one mm. The software will use the
   identical popup as on PiVR with the only
   difference being that you have to select a file with a known
   distance.
3. An estimate of the maximum animal speed. If unsure, it is better
   to overestimate the speed. If the tracking algorithm does not
   produce the desired result the maximum animal speed should be
   lowered.
4. An estimate of the maximum length of the animal. If unsure, it is
   better to overestimate the length. If the tracking algorithm does
   not produce the deired result, the maximum lenght should be lowered.

This tracking algorithm is identical to the tracking algorithm used for
live tracking in PiVR is used to track the animal.

The output is therefore almost identical to a tracking experiment run on
PiVR. See here for an explanation.

The only two differences are:

> 1. You will find a file called “DATE\_Time\_heuristics.csv” in the
>    analyzed folder. This file contains useful information in order
>    for you to define a new animal in “list\_of\_available\_organisms
>    .json”.
> 2. The “DATE\_Time” for bot the heuristics.csv and the data.csv
>    indicate the time of the analysis **NOT** the time of recording
>    the data.

#### The Image Data Handling Menu¶

The Image Data Handling Menu lets you choose a folder (either with
one experiment or a folder containing several experiments) and
convert the image data.

To convert image data (either a series of full frame images or
videos) first press the “Press to select data to modify” button on
the top left. Select a folder. You may:

1. Select a single experiment/folder. In that case you **must**
   uncheck the button on the right next to “More than one folder”.
2. Select a folder containing several experiments. If this folder
   only contains you want to apply the same image data conversion,
   you do not have to do anything. If there are other files or
   folders, please indicate a commonality between all the folder in
   the entry field below. This can be the data (e.g. 2019) a genotpe
   (e.g. Or42a) or…

   > The “Number of files” below the entry field indicates how many
   > files and folder in the folder you selected will be taken into
   > account if the current input is used.

Next, you have to choose the image conversion you want to perform
using the dropdown menu under “What modifications do you want to do?”
. Currently there are the following options:

##### Image Seq. to matrix¶

This is intended to be used after recording a series of images
with the Full Frame Recording Option option of
PiVR. The disadvantage of having a large number of single image files
instead of one large file (with the identical size) is the time it
takes the PC to read and write (e.g. copy, manipulate etc.) many
single image files. This program will help you to “pack up” your
image files. There are a variety of options you can choose from on
the right side of the GUI:

1. Zip Images: If this checkbox is marked, the images will be zipped.
   You will find a zip file called ‘images.zip’ in the folder where
   the original images were located. The zip file is uncompressed -
   the goal here is to have one file instead of many single files,
   not to save disk space!
2. Delete Original: If this checkbox is marked, the script will
   delete all images that are considered to be part of the recorded
   image series. After zipping the images, it is useful to delete the
   original images as they will only make data handling slow, but:
   **Make sure to only have this option on if at least one of the
   other options is selected, otherwise your data will be lost!**
3. Greyscale/Color: This dropdown menu lets you choose whether you
   want the images to be saved in greyscale (standard, especially if
   a standard PiVR version is being used) or if the input colors are
   color images and you want the output to be saved as color images.
   *Be careful with the color option, this has not been fully tested
   yet-.*
4. Save \*.npy: If this checkbox is marked, the script will save the
   images in a
   Numpy array.
   This can be very handy if you want to use python to run the
   downstream analysis of the data.
5. Save \*.mat: If this checkbox is marked, the script will save the
   images in a
   Matlab like array.
   This can be very handy if you want to use Matlab to run downstream
   analysis of the data.

##### Video conversion¶

This is intended to be used after recording a video with the
Video option of PiVR. Many user will find the h264
video file not convenient to work with as: (1) I find many video
players (e.g. VLC) have problems decoding the video and (2) the
metadata of the video seem to not always be correct.

Note

I found that the metadata of the videos recorded on the Raspberry
Pi are not completely correct. For example, when reading a video
file with the imageio module (using ffmpeg) the number of frames
is given as “inf” and the framerate seems to be always at 25, even
though the video was recorded at a different framerate. This
script takes care of this bug by using the ‘experiment\_settings
.json’ file created when using PiVR to record a video.

There are several options available to define the desired output:

1. avi/mp4/None: If you want to watch the video, it is probably a
   good idea to convert the video either into avi or mp4 as your
   video player will be better able to handle these formats (and the
   metadata of this file will be correct, see above). If “None” is
   chosen, the video will *not* be converted
2. Greyscale/Color: This dropdown menu, lets you choose whether you
   want the video to be in color (only works if original video is in
   color, of course) or converted to greyscale.
3. Save \*.npy: If this checkbox is marked, the video will save each
   frame in a Numpy array.
   This can be very handy if you want to use python to run the
   downstream analysis of the data.

   > **Note: Video encoding has been perfected over the years.
   > Decompressing a video often leads to surprisingly large files,
   > especially for long videos, or videos with a high framerate. If
   > the uncompressed video is larger than your computer has RAM this
   > script will most likely fail**
4. Save \*.mat: If this checkbox is marked, the script will save the
   images in a
   Matlab like array.
   This can be very handy if you want to use Matlab to run downstream
   analysis of the data.

   > **Note: Video encoding has been perfected over the years.
   > Decompressing a video often leads to surprisingly large files,
   > especially for long videos, or videos with a high framerate. If
   > the uncompressed video is larger than your computer has RAM this
   > script will most likely fail**

#### Display tracked experiment¶

This tool allows you to display the tracked animal in its arena
similar to a video player.

The experiment must have been generated using PiVR: either on on the
Raspberry Pi using the Tracking or
Virtual reality or by using the
post-hoc analysis
option in the ‘Tools’ Menu.

Besides enabling you to conveniently see where the animal was in each
frame, this tools allows you to:

1. Correct false Head/Tail assignments by swapping them
2. Save a video (in mp4 format) of the experiment.

To display an experiment, press on the “Select data to analyze”
button and select a folder containing a single experiment.

The software will automatically read the medatdata of the experiment
and will be displayed on the left side of the GUI.

In the center, the ‘Overview of tracking.png’ file is shown.

On the right you may choose a colormap before pressing “Press to show
behavior of the animal”.

The window above will emerge as a popup. While it is open the main
GUI is unavailable!

This window has the following functionality:

1. Updating Overview: This button will allow you to turn the main
   window off when playing back the experiment. This can be useful if
   you are only interested in the small images on the right.
2. The main figure in the center of the window is created by placing
   the raw small image (sm\_raw.npy) into the reconstructed background
   image (Background.jpg) using the bounding box coordinates
   (bounding\_boxes.npy). It also displays the detected Centroid, Tail
   and Head (\*\_data.csv) directly on the animal.
3. The three buttons below the main window on the left, “Showing
   Centroid”, “Showing Head” and “Showing Tail” allow you to turn off
   the different parts shown in the main figure.
4. If the head/tail assignment has been made incorrectly, chose a
   timepoint where head has been assigned the tail and press “Swap
   Head Tail”. This will reverse the head/tail classification between
   the previous and the next point in the experiment where no head
   and no tail can be assigned.
5. The toolbar below allows you to interact with the main window
   (zoom etc.).
6. The slider below lets you scroll through the experiment. Pressing
   the “Start Playing” button will display the experiment.
7. The Dropdown menu below the slider lets you play back the
   experiment at a variety of speeds you can choose from.
8. If there is a particular frame you want to go to, you can enter
   that number in the field below and press “Jump to frame”.
9. On the top right, the small raw image (sm\_raw.npy) is being
   displayed.
10. Below, the binary small image (sm\_thresh.npy) is being displayed.
11. In the “Save as Video” box at the bottom right you may create a
    video of the experiment. You can define:

    1. The start frame of the video
    2. The end frame of the video
    3. You may have a virtual arena in he background by selecting the
       appropriate csv file after pressing “select VR arena”.
    4. If you have been presenting a dynamic virtual arena, you can
       indicate the update rate in the entry box below. If you
       presented a static arena, you can leave it blank.
    5. When pressing “Save video” the video will be saved as “Video
       .mp4” in the experimental folder. **This usually takes a
       significant amount of time, even on a fast computer**.

#### Multi-Animal Tracking¶

This tools allows you to identify more than one animal in a video or
in a series of images.

The identification of the animals is achieved via background
subtraction of the mean image. Each animal is given an arbitrary
number. In the next frame, the animal closest to that number in the
previous frame is assigned.

This guide is intended to get you started quickly. If you are
interested in the more technical aspects, please see
`multi_animal_tracking.MultiAnimalTracking`

Note

Identifying more than one animal in an experiment is
computationally challenging. There are several specialized tools
such as the multiworm tracker,
Ctrax,
idtracker,
idtracker.ai and
MARGO. The PiVR
multi-animal tracking software has **not** been benchmarked
against these tools. This software has several limitations. It is
probably ok to use in cases where you are interested in counting
how many animals are in a general area. It is not recommend to use
the tracker for other parameters, such as calculating animal speed
(due to loss of identity after collision and ‘jumps’ in the
trajectory) and similar parameters.

After selecting a folder containing a video of image sequence of an
experiment containing multiple animals, press the “Press to show the
behavior of the animals” button.

The software will now load the image data (which can take a
considerable amount of time) and display a new window which will help
you to optimize the tracking parameters.

Note

You might notice that the image is distorted. This is due to the
Raspberry Pi camera lens. See here.

First, have a look at the all important “# of animals in the current
frame” on the bottom right of the popup. In this video there are only
6 animals, but the algorithm detects 10 objects as “animals”. The
blobs identified as animals are indicated using small rectangles in
the main figure.

Important

The goal is to have have as many frames as possible contain only the
expected amount of animals.

To achieve this goal, start by defining the area in which animals can
move into. In this particular experiment, the outline of the petri
dish can be clearly seen:

1. Press the “Select Rectangular ROI”. The button will turn red.
2. Using the mouse, create a rectangle in the main window. The result
   is indicated. The “# of animals in the current frame” is
   immediately updated

While this is already better, there are still two blobs wrongly
identified as animals, one at (y=300,x=100) and the other at (y=270,
x=480).

By increasing the “Threshold (STD from Mean) number these
mis-identified blobs are not mistaken for animals animal:

There are now no mis-identified animals. While there are a total of 6
animals in the image, the algorithm can only detect a 5. To
understand why that is the case, press the magnifying class symbol
below the main figure and draw a rectangle around the region you want
to enlarge.

It is now obvious that 4 out of 6 animals are properly identified.
Two of the animals, at (y=260, x=360) are very close to each other
and are identified as one animals, however. *There is no way for this
tracking algorithm to separate animals that are touching each other!*.

Now you need to make sure that the image parameters are such that you
get the expected animal number in all frames. To not have to go
through each of the frames manually, you can just press “Auto-detect
blobs” on the bottom right. This will run a script that will take the
current image detection parameters into account and just count how
many blobs are counted as animals. The result is plotted in the
figure on the top right.

This result indicates that at the beginning of the video there are
several frames where only 5 animals can be detected. By visually
inspecting these frames it becomes obvious that this is due to the
two animals touching each other as they already do in the first frame.

Next, find frames that have the wrong amount of animals. Try to fix
them using the image parameter settings.

There can be situations where the animal number will just be wrong
and it can not be fixed. The algorithm can handle this if the number
of those frames is low.

Once you have optimized the image parameters, go to frame where the
correct amount of animals is detected. **This is crucial as it tell
the algorithm how many animals to expect!**. Then press “Track animals”.

A new popup will open. It indicates how many animals (and where) are
detected in this frame. Each animal gets an arbitrary number. If the
number of animals is correct, press “Looks good, start tracking”.
Else press “Not good, take me back”.

The tracking itself is computationally quite expensive and therefore
usually takes a while to complete. To speed up tracking, you can
press the “Updating Overview” button above the main window.

Once tracking has concluded the result will be displayed in the main
window as shown below.

If there are huge gaps in the trajectory, for example because an
animal could not be detected for a while, you can press the
“interpolate centroids” button. This will calculate realistic
possible distances travelled (based on ‘Max Speed Animal[mm/s])
between frames and try to connect trajectories. This is a
**untested** feature - use at your own risk. Ideally you should not
have to use this option.

##### Multi Animal Tracking Output¶

After using the Multi-Animal Tracker, you will find two new files in
the experimental folder:

1. The “Background.npy” file which is just the mean of all images,
   resulting in the background image used during tracking.
2. The “XY\_positions.csv” file contains the X and Y coordinates for
   each identified animal for each frame. For frames with not enough
   animals, the corresponding row will be empty.

#### Creating a new Virtual Arena¶

How to create a new virtual arena, the essential tool that gives
Pi\*\*VR\*\* its name?

First, a brief overview of what the different elements of an arena
file mean:

You can find several example virtual arena files in the
PiVR/VR\_arenas folder: As an example lets analyze
“640x480\_checkerboard.csv”

The file itself is a 2D matrix with 640 columns and 480 rows. Each
value in the matrix defines what happens on that pixel on the camera.

For example, if you define the value 100 at position column = 75
and row = 90 here in the virtual arena and then present this virtual
arena to the animal using the Closed Loop stimulation
tab on PiVR, if the animal is at pixel column = 75 and row = 90 the
intensity of 100 will be played back.

A big advantage of virtual realities over real environments is the
control the experimenter has over the experimental conditions. When
running an experiment, one often has to repeat trials for many times.
In real environments, the experimenter can never have identical
initial conditions, e.g. the animals is placed at a slightly
different position, the animal moves in different conditions before
the tracking even starts etc. With virtual reality, this factor
(which often introduces variability into data) can be alleviated: The
experimenter can define a virtual reality arena and the animal will
always be presented with the identical initial conditions.

To do this, lets examine another example virtual arena file you can
find int PiVR/VR\_arenas: “640x480\_gaussian\_centred\_animal\_pos[250,
240,0.0].csv”

This file has a string at the end of its filename: *animal\_pos[250,
240,0.0]*. This string indicates where the animal must start in
relation to the virtual reality. In this example, wherever the animal
is in the real image when the experiment starts, the virtual reality
will be translated so that it is at x-coordinate 250 and y coordinate
240.

In addition, if the third value (here 0.0) is defined, the movement
of the animal during detection is taken into account: The virtual
arena is rotated so that the animal always starts going into the same
direction relative to the virtual arena. The angle you may use goes
from -pi to +pi (see atan2.

If you want to create a virtual reality arena from scratch, for
example in python, all you need to do is create a matrix with the
correct dimension, fill it with values between 0 and 100 as you see
fit and export the file as csv.

```
import numpy as np

virtual_arena = np.zeros((480,640),dtype=np.float64)
# Define parts of the arena where the animal is supposed to be
# stimulated e.g. by typing
virtual_arena[:,0:100] = 100
virtual_arena[:,101:200] = 75
virtual_arena[:,201:300] = 50
# this will give you a very coarse grained virtual arena that will
# stimulate strongly if the animal is on the left side, stimulate
# 75% if the animal is a bit more on the right and 50% if it is
# still on the left but almost in the middle. The rest is
# unstimulated as of yet.

# Now you need to save the arena. Let say you want to have the animal
# start ascending the gradient from the middle (essentially animal
# has to move to the left in the virtual arena)
np.savetext("Path/On/Your/PC/640x480_my_awesome_arena_animal_pos[300,240,0.0].csv")
```

Alternatively, you can use the “Tools->Draw VR Arena” option on the
PC version of PiVR.

You will find the following empty canvas. You can open a previously
defined virtual arena or work on the blank canvas. Either way, you
have the the option to create *gaussian shaped 2D circles* and
*rectangles*. To “draw” such a gaussian shaped 2D circle, you can
either press on “Draw Gaussian Circle with Mouse” (and then click
somewhere on the canvas) or you can press on “Draw Gaussian Circle at
defined coordinates”.

You can change the Gaussian shaped 2D circle by changing its Sigma,
its size and the intensity.

Analogous, you can define the size and the intensity of the rectangle
by entering the desired value.

In the example below, I used the standard settings for the gaussian
circle size but changed the “Coordinates” to the values shown. Then I
pressed on “Draw Gaussian Circle at defined coordinates”(red squares).
Then I modified the “Coordinates” of the rectangle on the right to
x=100 and y=100 and pressed on “Draw Rectangle at defined
coordinates” (green squares).

There are 3 additional buttons that expand the possibilities of drawing
virtual arenas:

1. Invert: If this is on, whenever you draw a circle or rectangle, it
   subtracts the values from the intensity that is already present.
   This option was used to create the following virtual arena:
   “640x480\_volcano\_animal\_pos[320,240,0.0].csv”
2. Overwrite: will just overwrite the previous pixel values with the
   new values with no regard to the previous value (as opposed to
   “Invert” and “Additive”)
3. Additive: If you place a 50% rectangle somewhere and then place
   another on top of it, usually nothing will happen as the absolute
   value is being drawn. If this is on, the values are “added”.

   Note

   If you go above 100%, everything will be normalized.

On the right side of the canvas you can define the starting position
of the animal in the virtual arena. Besides just x and y position,
you can define the direction from which the animal is coming from.

You can use the mouse, either by clicking (just x and y position) or by
clicking and dragging (x, y and angle). Alternatively, you can use
precise coordinates.

Note

Angle can go from -pi to +pi. See
atan2 for visualization.

Once you are done with the arena, make sure to save it. Then you can
just quit the window.

PiVR has been developed by David Tadres and Matthieu Louis (Louis Lab).

### Advanced topics¶

#### Simulate Real-Time Tracking¶

Imagine setting up your experiment: preparing the animals, booking
the setup/room for a whole afternoon…and then the tracker does not
track the animal half of the time!

It is quite frustrating sitting in a dark room trying to troubleshoot
these kind of problems.

To alleviate situations like these, there is the option to *simulate*
real time tracking after installing the PiVR software on a
PC (=not on the Raspberry Pi).

1. At the PiVR setup, double check that:

   1. you have set the resolution to
      640x480,
   2. that the pixel/mm is set correctly,
   3. that the framerate is identical to
      the framerate you are trying to achieve with real-time tracking.
   4. that you have selected the correct animal
2. Then, record a video with these settings. Then
   record some more.
3. Transfer the video data to your PC where you have installed the
   PiVR software and select the Debug->Simulate Online Tracking
4. Make sure the Animal Detection Method is the same as the one you want to use
   during Real-Time tracking.

   Note

   This has not been tested with Mode 2
5. Select a single folder. You will now see the metadata created
   while the video was taken. Carefully inspect it to see if the
   settings are as you expect them.
6. Press the ‘Track Single Animal’ button - you will get a popup as
   soon as the animal detection algorithm detects a moving object.
7. After pressing ok, you will see what the animal detection
   algorithm has defined as the animal.

   It is obvious something has gone wrong here as the image on the
   right (the binary image) has a lot of spots where the image is
   white (=areas which are considered to be the animal)
8. After pressing Ok, the tracking algorithm starts - as the animal
   has not been properly identified in the first frame, the tracking
   algorithm is unable to identify the animal during tracking as well:
9. After going through the simulated tracking, the potential source
   of the problem has been identified: The animal can not be detected
   correctly. There are a many reason why this could be:

   1. The edge of the dish seems to have moved a bit during the first
      couple of frames (red rectangle). If you are able to stabilize
      the setup to ensure no movement while doing experiments, this
      problem should be solved.
   2. The fact that several spots in the middle of the dish are
      wrongly binarized as the potential animal, indicates that the
      detection algorithm has trouble setting the threshold correctly.
      This problem arises because the threshold is calculated as 2
      standard deviations from the mean of the pixel intensities in
      the subtracted image `pre_experiment.FindAnimal.define_animal_mode_one()`.
      While the animal is the darkest spot in the image, the whole
      petri dish is darker than the background which might lead to
      this problem.

      There are two general ways to solve this problem:

      > 1. Optimize the imaging conditions
      >    so that the animal has a higher contrast to the
      >    background, which should be as homogenous as possible.
      >    See here for an example.
      > 2. Optimize the animal parameters. You can follow this
      >    guide to set stringent animal
      >    parameters for tracking.
10. There are many ways how tracking can fail. Only a single
    example is described above. I hope the walkthrough will enable
    you to generally get an idea where during tracking the
    algorithm fails.

#### Tracking of a new animal¶

PiVR has been used to track a variety of animals: Walking adult fruit
flies, fruit fly larvae, spiders, fireflies, pillbugs and zebrafish.

For the tracking algorithm to function optimally, it takes several
“animal parameters” into account:

1. The amount of pixels the animal will fill in the image.
2. The “roundness” of the animal.
3. The proportions of the animal.
4. The length of the animal.
5. The speed of the animal.

For each animal you can choose in
Options->Select Organism these parameters
were defined. You can find them in the file
“list\_of\_available\_organisms.json” in source code.

If you want to track an animal that is **not** on the list you can
always try to use the “Not in list” option. However, the tracking
algorithm might not work optimally.

There is a straightforward pipeline to collect the necessary animal
parameters to optimize real-time tracking:

1. Place your (**single!!**) animal in the arena you want to use for
   your experiment.
2. As always, do not forget to define the pixel/mm.
3. Select “Not in List” under Options->Select
   Organism.
4. Record a video. If you use a fast animal, make
   sure to select a sufficiently high framerate. **You must use
   640x480 resolution**. As always it is imperative that the camera
   and the arena are stable during recording, i.e. nothing in the
   image should move except the animal!
5. Record for a couple of minutes, i.e. 5 minutes.
6. Make sure you have videos with animals moving as fast as they
   might in your actual experiment.
7. It is also necessary that the animals move for a large fraction of
   the video!
8. Take the videos to your PC on which you have installed the
   PiVR software.
9. To observe what the algorithm is doing, turn the Debug mode
   on. This is recommend as you will see immediately
   if and where something goes wrong. This can help you to solve
   tracking problems.
10. Analyze each video using the Tools->Analysis: Single Animal
    Tracking option.
11. If using the Debug mode, you will get informed as soon as the
    algorithm detects an object that is moving. It will also inform
    you how much space (in pixels) the detected animal occupies, its
    eccentricity (‘roundness’) and a parameter for proportions (Major
    axis over minor axis). If the identified object clearly is **not**
    the animal answer the question with “No” and the algorithm will
    look in the next frame the largest moving object.
12. Next, you will be shown a side by side comparison of the original
    picture (with a box drawn around the detected animal and the binary
    image you have seen in the previous popup.
13. The algorithm will then start tracking. You will see an overview
    of how the algorithm detects the animal: On the left you can see
    the original image. In the center you can see the binary image:
    The grey area indicates the search box (which depends on defined
    max speed of animal, pixel/mm and framerate) and in white the
    pixels that are below threshold. The black area is not considered
    as it is too fare away from the position of the animal in the
    previous frame. On the right, you can see the result of the
    tracking: A box drawn around the identified animal. In addition,
    you can see the animal parameters you are looking for. These are
    just for your information, read below to see how to comfortably
    get the list of these parameters.
14. After running the Single Animal Tracking algorithm, you will find
    a number of new files in each experimental folder. To get to the
    animal parameters, open the file “DATE\_TIME\_heuristics.csv”, for
    example with excel.
15. Each row in the table stands for one frame. The title of the
    column describes the value.
16. You need to get the following values to get all animal parameters:

    1. A minimum value for filled area (in mm)
    2. A maximum value for filled area (in mm)
    3. A minimum value for eccentricity
    4. A maximum value for eccentricity
    5. A minimum value for major over minor axis
    6. A maximum value for major over minor axis
    7. Maximum skeleton length
    8. Maximum speed of the animal (mm/s)
17. As the tracking algorithm needs the extreme values to function
    properly, I have found it easiest to plot a Line Plot for each
    experiment for each of the relevant parameters. For example for
    the filled area:
18. Write down the maximum and minimum value for each of relevant
    parameters. In this example, the minimum value for filled area in
    mm would be ~25 and the maximum would be ~90.
19. Do the same for eccentricity, major over minor axis, skeleton
    length and maximum speed (mm/s)
20. Then do the same for a few other videos. The goal is to get
    extreme values without having to put 0 as minimum and infinity as
    maximum.
21. In this example I have found the following parameters:

    1. Minimum value for filled area (in mm): 20
    2. Maximum value for filled area (in mm): 90
    3. Minimum value for eccentricity: 0.4
    4. Maximum value for eccentricity: 1
    5. Minimum value for major over minor axis: 1
    6. Maximum value for major over minor axis: 3.5
    7. Maximum skeleton length: 14
    8. Maximum speed of the animal (mm/s): 350
22. Now go to the PiVR software folder on your PC and find the file
    named: “list\_of\_available\_organisms.json”:
23. Open it with an text editor. I often use “Code Writer” that ships
    with Windows. You will see that there are repeating structures: A
    word, defining the name of the animal, then a colon and then some
    image parameters in brackets.

    Note

    Json files require correct formatting. Be careful to not accidentally deleting
    commas etc.
24. To enter your animal parameters you have two options: The easiest
    (and safest) option is to choose an animal in the list that you
    are certain to never use and just enter your parameters:

    Alternatively, you may also enter a new “cell” at the end of the
    list. There is no limit on the number of different animals that
    can be entered in this list.
25. Now save the file (do **not** rename it - If you want to keep a
    backup, rename the original, i.e. to
    “list\_of\_available\_organisms\_original.json”.)
26. Restart the PiVR software (so that it reads the newly defined
    animal parameters).
27. If you want to know whether PiVR is able to perform real-time
    tracking, you can open the “experiment\_settings.json” files in one
    of the video folders you used to find the animal parameters (or a
    newly created video) and change the “Model Organism” cell name to
    your animal name
28. Now, select the “Debug->Simulate Online Tracking” window, select a
    video and check whether the algorithm can track the animal in
    real-time. If not, you might have to select more
    stringent animal parameters and/or you have to optimize imaging
    conditions.

PiVR has been developed by David Tadres and Matthieu Louis (Louis Lab).

### PiVR software installation¶

#### Install PiVR on the Raspberry Pi¶

The easiest way to install PiVR on your Raspberry Pi is to just
follow the instructions during hardware construction.

If you just want to download the installation script to your
Raspberry Pi, `press
here`

#### Install PiVR on a PC¶

1. Install miniconda
   on your computer.
2. Install git on your computer

Note

If you have Windows, you may try this
guide which will install the software
more or less automatically.

Note

If you have Ubuntu, you may try
this guide which will install the
software more or less automatically.

1. Now, create an empty conda environment:

   ```
   conda create --name PiVR_environment
   ```
2. Activate the environment you just created by typing:

   Linux/Mac:

   ```
   source activate PiVR_environment
   ```

   Windows:

   ```
   activate PiVR_environment
   ```
3. Install the a number of packages which are necessary to run the PiVR
   software by copying each line of code into the Terminal

   ```
   conda install -y python=3.7

   conda install -y matplotlib

   conda install -y pandas

   conda install -y scipy

   conda install -y natsort

   conda install -y -c conda-forge scikit-image
   ```
4. You have now prepared the
   virtual environment PiVR will be running in.
5. Using the anaconda terminal, change the working directory to a
   folder where you want to store the actual PiVR software.

   > ```
   > cd C:\Users\UserA\Documents>
   > ```
   >
   > Note
   >
   > You might want to write down the exact path so that you will
   > find it again in the future!
6. Download the software by typing:

   > ```
   > git clone https://gitlab.com/louislab/PiVR
   > ```
7. Now navigate into the folder you have just downloaded by typing:

   ```
   cd PiVR
   ```
8. To start the PiVR software type:

   ```
   python start_GUI.py
   ```

#### Install PiVR on a Windows 10 PC¶

Important

If you are having trouble with this installation procedure, do the
manual install.

Warning

Only Win10, 64bit tested!

1. Open the Anaconda prompt
2. Navigate into a folder where you want to store the PiVR software,
   for example:

   ```
   cd C:\Users\UserA\Documents>
   ```
3. Download the software by typing:

   ```
   git clone https://gitlab.com/louislab/PiVR
   ```
4. Navigate into the installation folder by typing:

   ```
   cd PiVR\Installation_update
   ```
5. Create the Windows 10 virtual environment for the PiVR software to
   run using the provided package list by typing:

   ```
   conda create --name PiVR_environment --file PiVR_Win64.txt
   ```
6. Once done, activate the virtual environment by typing:

   ```
   activate PiVR_environment
   ```

   You know you successfully activated the virtual enviroment if it
   says ‘(PiVR)’ at the beginnig of the line in the terminal.
7. Start the software by going into the folder where the file
   “start\_GUI.py” can be found, which is the parent folder of the
   installation folder you should be in now. So just type:

   ```
   cd ..
   ```
8. And to finally start PiVR, type:

   ```
   python start_GUI.py
   ```

#### Install PiVR on a Linux PC¶

Important

If you are having trouble with this installation procedure, do the
manual install.

Warning

Only Ubuntu, 64bit tested)

1. Open the Terminal
2. Navigate into a folder where you want to store the PiVR software,
   for example:

   ```
   cd /home/UserA
   ```
3. Clone the repository by typing:

   ```
   git clone https://gitlab.com/louislab/PiVR
   ```
4. Navigate to the “Installation\_update” folder of the repository you
   just cloned:

   ```
   cd /home/UserA/PiVR/PiVR/Installation_update
   ```
5. Create the Linux virtual environment for the PiVR software to
   run using the provided package list by typing:

   ```
   conda create --name PiVR_environment --file PiVR_Linux64.txt
   ```
6. Once done, activate the virtual environment by typing:

   ```
   source activate PiVR_environment
   ```

   You know you successfully activated the virtual enviroment if it
   says ‘(PiVR)’ at the beginnig of the line in the terminal.
7. Start the software by going into the folder where the file
   “start\_GUI.py” can be found, which is the parent folder of the
   installation folder you should be in now. So just type:

   ```
   cd ..
   ```
8. Start the program by typing:

   ```
   python start_GUI.py
   ```

#### Start PiVR on a PC¶

Note

To run PiVR, you of course need to first
install the software.

1. Open the Anaconda terminal (Windows) or Terminal (MacOS/Linux)
2. Activate the virtual environment you have created during the
   installation. If you followed these instructions type:

   Windows:

   ```
   activate PiVR_environment
   ```

   Linux/MacOS:

   ```
   source activate PiVR_environment
   ```
3. Change directory to the folder where you downloaded the PiVR
   software into. In the example here we used:

   ```
   cd C:\Users\UserA\Documents\PiVR\PiVR
   ```
4. Start PiVR software by typing:

   ```
   python start_GUI.py
   ```

PiVR has been developed by David Tadres and Matthieu Louis (Louis Lab).

### PiVR software documentation¶

- Graphical User interface
- Tracking software
- Analysis
- Virtual Arena drawing
- Image Data Handling

#### PiVR GUI source code¶

This page contains the classes used to construct the graphical user
interface (GUI).

*class* `start_GUI.``PiVR`(*\*args*, *\*\*kwargs*)[source]¶
:   This class initializes the GUI the user will see. There
    are several different frames (e.g. “Tracking” vs “Virtual
    Arena”) that all are created differently.

    To do this, the “PiVR” class instantiates (=calls) a number of
    other classes. To help with this the following three “helper”
    classes are important:

    > 1. “CommonVariables” contains variables that are true
    >    between frames,
    > 2. “SubFrames” helps with the creation of
    >    the different frames and finally
    > 3. “CommonFunction”
    >    which contains functions that are called in different
    >    frames.

    The actual frames (e.g. “TrackingFrame”) are then created by
    “constructor” classes which call different components of the
    three classes described
    above.

    The “helper” classes are necessary as they can save variables
    and functions between different frames (similar to global
    variables). For example, if the user would select at
    particular folder to save all the experimental data,
    the “CommonVariables” class saves this folder when the user
    is then switching from e.g. the “Tracking” frame to the
    “Virtual Arena” frame.

    `access_subframes`(*page\_name*)[source]¶
    :   This function just return the instance of the currently
        active (=in foreground) window

    `call_start_experiment_function`(*page\_name*)[source]¶
    :   This function will be called by the button that says
        ‘start experiment’. It will look in the currently active
        frame for a function called ‘start\_experiment\_function’.

    `show_frame`(*page\_name*)[source]¶
    :   This function is called when the user presses on a different
        frame. It takes the selected frame and raises it to the top.

        In addition, it saves the current page name which is needed
        to pass around as a reference to the currently active frame
        when calling functions that are generally called, such as
        starting an experiment!

*class* `start_GUI.``DynamicVirtualRealityFrame`(*parent*, *controller*, *camera\_class=None*)[source]¶
:   The constructor class used to create the “Dynamic VR Arena” frame.

    `start_experiment_function`()[source]¶
    :   Each constructor class which is used to start an experiment
        has this function. They all have the same name. This is the
        one in the “DynamicVirtualRealityFrame”

        It checks for:

        > 1. correct camera resolution,
        > 2. that the user has specified the pixel per mm
        > 3. that a copy of the dynamic virtual reality fits into
        >    RAM memory.

        If one of these tests fails, the user gets an error message
        and the experiment will not start.

        If the experiment does start, the
        `control_file.ControlTracking` is called that then
        handles the detection and tracking of the animal.

*class* `start_GUI.``TrackingFrame`(*parent*, *controller*, *camera\_class=None*)[source]¶
:   The constructor class used to create the “Tracking” frame.

    `start_experiment_function`()[source]¶
    :   Each constructor class which is used to start an experiment
        has this function. They all have the same name. This is the
        one in the “TrackingFrame”

        It checks for:

        > 1. correct camera resolution,
        > 2. that the user has specified the pixel per mm

        If one of these tests fails, the user gets an error message
        and the experiment will not start.

        If the experiment does start, the
        `control_file.ControlTracking` is called that then
        handles the detection and tracking of the animal.

#### PiVR Tracking source code¶

##### Detection¶

*class* `pre_experiment.``FindAnimal`(*boxsize, signal, debug\_mode, stringency\_size=0.01, stringency\_centroid=0.01, cam=None, resolution=[640, 480], display\_framerate=2, model\_organism=None, offline\_analysis=False, pixel\_per\_mm=None, organisms\_and\_heuristics=None, post\_hoc\_tracking=False, animal\_detection\_mode='Mode 1', simulated\_online\_analysis=False, datetime='not defined'*)[source]¶
:   Before the algorithm can start tracking it first needs to
    **identify the animal** and create a **background image** that
    can be used for the rest of the experiment. Three “Animal Detection
    Modes” are available. See
    here for a high
    level description which one should consult to understand the
    advantages and limitations of each Mode.

    **Mode 1:**

    If the background is not evenly illuminated or if the animal
    moves fast and often goes to the edge
    Mode 1 is a
    safe and easy choice.

    > 1. Identify the region of the picture where the animal is
    >    located by detecting movement. For this
    >    `find_roi_mode_one_and_three()` is called.
    > 2. Reconstruct the background image from the mean image
    >    constructed while the animal was identified. For this
    >    `define_animal_mode_one()` is called.

    **Mode 2:**

    Mode 2 can be used if the
    animal can be added to the arena without changing anything in the
    field of view of the camera while doing so.

    > 1. Takes a picture before the animal is placed and a
    >    second picture after the animal is placed. This approach was
    >    used before in the SOS tracker (Gomez-Marin et al.,
    >    2012).
    >    This is called animal detection Mode 2. This
    >    only works if the only object that is different in
    >    the image is the animal that one wants to track. If the user
    >    needs to put a lid it the resulting image is often
    >    too different and breaks this approach! For this
    >    `find_roi_mode_two()` is called.
    > 2. Computationally the identification of the animal in two
    >    images, one with and one without, is very simple. Just
    >    subtract the two images, and what is standing out must be
    >    the object the user wants to track. For this
    >    `define_animal_mode_two()` is called

    **Mode 3:**

    This methods is bit more complicated compared to Mode 1 and Mode 2.
    It attempts to combine the ease of use of Mode 1 with the
    perfectly “clean” background image produced by Mode 2.

    This method only works well if several conditions are
    met. We only used this
    method with the slow fruit fly larva. See
    here for a detailed high level
    description

    > 1. Identify the region of the picture where the animal is
    >    located by detecting movement. This is in fact identical
    >    to Mode 1, as the same function is called:
    >    `find_roi_mode_one_and_three()`
    > 2. Then the animal must be defined using a binary image. This
    >    is a critical step and necessitates that the animal
    >    clearly stands out compared to the background. The
    >    function is: `define_animal_mode_three()`.
    > 3. To reconstruct the background (for the experiment) the
    >    animal must leave the original position. The relevant
    >    function: :func:`animal\_left\_mode\_three’
    > 4. Then the background reconstruction is done by taking the
    >    image after the animal has left the original position
    >    and just taking the area the animal originally occupied
    >    and inserting it into the first picture taken. The
    >    function doing that is
    >    `background_reconstruction_mode_three()`
    > 5. For the tracking algorithm to start it needs to know
    >    where the animal went while all of the above was going on.
    >    `animal_after_box_mode_three()`

    Finally, this class also holds a offline animal detection
    function: `find_roi_post_hoc()`. This is used when running
    **Todo-Link** Post-Hoc Single animal tracking. For example for
    debugging or to define a new model organism the user wants to
    track in the future.

    `animal_after_box_mode_three`()[source]¶
    :   After making sure that the animal left the original position,
        we have to find it again.

    `animal_left_mode_three`()[source]¶
    :   This function compares the first image the algorithm has
        taken with the current image. It always subtracts the two
        binary (thresholded using the mean +3\*STD) images.
        The idea is that as soon as the animal has left the original
        position, the subtracted image will only have the original
        animal left. In other words, the closer this subtracted
        image is to the first binary image, the more of the animal
        has already left the initial bounding box.

    `background_reconstruction_mode_three`()[source]¶
    :   After the animal left the original position, take another
        picture. Use the bounding box coordinates defined for the first
        animal to cut out that area of the new image. Then paste it
        into the original position where the animal was.

        This leads to an almost perfect background image.

    `cancel_animal_detection_func`()[source]¶
    :   When user presses the cancel button turn the bool to
        true to cancel the animal detection at the next possible
        step

    `define_animal_mode_one`()[source]¶
    :   This function is called when the user uses Animal Detection
        Mode #1

        This function does not do local thresholding of the first
        frame. Instead it just reconstructs the background image from
        the mean image it has constructed while identifying the
        animal. This will almost always leave part of the animal in
        the background image. Usually this is not a problem as the
        whole animal is larger than just a part of it.

    `define_animal_mode_three`()[source]¶
    :   With the information where to look we identify the larva
        using local thresholding (we couldn’t do that before
        with the whole image)

        This only works if the animal is somewhere where the
        background illumination is relatively even and the
        animal stands out clearly relative to it’s immediate background!

    `define_animal_mode_two`()[source]¶
    :   With the information where to look we identify the animal
        using local thresholding (we couldn’t do that before
        with the whole image)

        This is only saved if the animal is somewhere where the
        background illumination is relatively even and the
        animal stands out clearly relative to it’s immediate background!

    `define_animal_post_hoc`()[source]¶
    :   With the information where to look, the animal can be
        identified using local thresholding.

    `error_message_pre_exp_func`(*error\_stack*)[source]¶
    :   Whenever something goes wrong during animal detection this
        function is called. It writes the traceback of the
        error into a file called \_ERROR\_animal\_detection.txt in the
        experimental folder.

    `find_roi_mode_one_and_three`()[source]¶
    :   Identification of the original region of interest (ROI):

        This function identifies a region in the image that contains
        pixels that change over time. The assumption is that the only
        object moving in the field of view should be the animal the
        user is interested in.

        To achieve this, the camera provides images. This function
        will take the mean of the images taken so far. It will then,
        starting from the second frame, start to subtract the newest
        frame from the previously taken images. In the resulting
        image, anything that moves will clearly stand out compared
        to the background. A region of interest is then drawn around
        those pixels to be used later on.

    `find_roi_mode_two`()[source]¶
    :   Sometimes the user can not use the automatic animal
        detection Method 3 because the background is not completely
        homogeneous. If the user still needs to have a clear
        background image without any trace of the animal this
        Methods can be used. It is similar to the one used in
        Gomez-Marin et al., 2011.

        > 1. Take an image before placing the animal
        > 2. Place the animal
        > 3. Take another picture and subtract from the first

    `find_roi_post_hoc`()[source]¶
    :   Identifies animal in post-hoc analysis.

        Normally used when user defines a new animal The workflow
        consists of the user taking a
        video and then running the **TODO**-Link
        *Post-Hoc Single Animal Analysis*. This function
        identifies the animal before the actual tracking starts.

        It first reads all the images (user should provide which file
        format the images are in) and zips them up so that the folder
        gets easier to copy around. It also creates a numpy array with
        all the images for this script to use.

        It then takes the mean of all the
        images to create the background image.

        It then smoothens the background image using a gaussian
        filter with sigma 1.

        It then starts to loop over as many images as necessary by:

        > 1. subtracting the mean (background) image from the
        >    background.
        > 2. Calculate the threshold by defining everything below
        >    or above (depending on **TODO** link *signal*) 2\*std from
        >    the mean as signal.
        > 3. Use the regionprop function
        >    to measure the properties of the labeled image regions.
        > 4. Depending on the amount of props, different rules apply:
        >
        >    > 1. If more than one props, cycle through them testing
        >    >    if they are fullfilling the minimal requirements to
        >    >    count as potential animals: *Filled Area* Min and
        >    >    Max, *Eccentricity* Min and Max, and *major over
        >    >    minor axis* Min and Max.
        >    >
        >    >    > 1. If one found > That’s the animal
        >    >    > 2. Else, go to next image
        >    > 2. If only one props, that’s the animal and break out
        >    >    of the loop
        >    > 3. If no blob, go to next image

##### Tracking¶

*class* `control_file.``ControlTracking`(*boxsize=20, signal=None, cam=None, base\_path=None, genotype=None, recording\_framerate=30, resolution=[640, 480], recordingtime=20, pixel\_per\_mm=None, model\_organism='Not in list', display\_framerate=None, vr\_arena=None, pwm\_object=None, placed\_animal=None, vr\_arena\_name=None, offline\_analysis=False, time\_dependent\_stim\_file=None, time\_dependent\_stim\_file\_name=None, vr\_arena\_multidimensional=False, high\_power\_led\_bool=False, minimal\_speed\_for\_moving=0.25, observation\_resize\_variable=1, organisms\_and\_heuristics=None, post\_hoc\_tracking=False, debug\_mode='OFF', animal\_detection\_mode='Mode 1', output\_channel\_one=[], output\_channel\_two=[], output\_channel\_three=[], output\_channel\_four=[], simulated\_online\_analysis=False, overlay\_bool=False, controller=None, background\_channel=[], background\_2\_channel=[], background\_dutycycle=0, background\_2\_dutycycle=0, vr\_update\_rate=1, pwm\_range=100, adjust\_intensity=100, vr\_stim\_location='NA'*)[source]¶
:   Whenever the tracking algorithm is called, this class controls
    first the detection algorithm, prepares the virtual arena if
    necessary, and then calls the tracking algorithm.

    `adjust_arena`()[source]¶
    :   This function translates and rotates the virtual reality if
        necessary. It also adjusts the desired stimulus intensity.

        For both translation and rotation the
        scipy.ndimage.affine\_transform function is used:
        https://docs.scipy.org/doc/scipy-0.19.1/reference/generated/scipy.ndimage.affine\_transform.html

        For the translation, the following transformation matrix is
        used with

        \({\zeta}\) being the difference between the animal
        position and the desired animal position:

        \[\begin{split}\begin{bmatrix}
        Y' \\
        X' \\
        0
        \end{bmatrix} =
        \begin{bmatrix}
        1 & 0 & Y \zeta \\
        0 & 1 & X \zeta \\
        0 & 0 & 0
        \end{bmatrix}\end{split}\]

        To translate and rotate the arena, the following is done:

        > 1. Take the position of the animal in the real world and
        >    the position of the animal in the virtual reality.
        >    Translate the arena by the difference, effectively
        >    using the placed animal coordinates as the origin
        >    around which the arena is rotated.
        > 2. Then translate the arena to the origin of the array at
        >    [0,0]
        > 3. Rotate the arena by the difference in real movement
        >    angle and the desired angle
        > 4. Finally, translate the arena back to the desired
        >    position, defined by both the real position of the
        >    animal and the desired position.

        This is implemented by the following linear transformation
        where:

        \({\zeta}\) is the difference between the animal
        position and the desired animal position and,

        \({\eta}\) is the desired animal position

        \[\begin{split}\begin{bmatrix}
        Y' \\
        X' \\
        0
        \end{bmatrix} =
        \begin{bmatrix}
        1 & 0 & Y \zeta \\
        0 & 1 & X \zeta \\
        0 & 0 & 0
        \end{bmatrix}
        \cdot
        \begin{bmatrix}
        1 & 0 & Y \eta \\
        0 & 1 & X \eta \\
        0 & 0 & 0
        \end{bmatrix}
        \cdot
        \begin{bmatrix}
        \cos & -\sin & 0 \\
        \sin & \cos & 0 \\
        0 & 0 & 1
        \end{bmatrix}
        \cdot
        \begin{bmatrix}
        1 & 0 & -Y \eta \\
        0 & 1 & -X \eta \\
        0 & 0 & 0
        \end{bmatrix}\end{split}\]

    `high_power_LED_arena_inversion_func`()[source]¶
    :   When the high powered PiVR version is used, the software has
        to handle the unfortunate fact that the LED controller of the
        high powered PiVR version is completely ON when the GPIO is
        OFF and vice versa. This of course is the opposite of what
        happens in the normal version.

        Internally, the software must therefore invert the arena if
        that’s the case. This function takes care of this.

        The end user does not need to know this. From their
        perspective they are able to use the same input arena they
        would use for the standard version while getting the expected
        result.

    `show_dynamic_vr_arena_update_error`()[source]¶
    :   This function warns the user that an incompatible
        framerate/dynamic arena update frequency has been
        chosen. For example, if the framerate is 30frames
        per second and the update rate is 10Hz the arena
        will be updated every 3rd frame (30/10=3). This is
        of course possible.
        If the framerate is 30frames per second and the
        update rate is set to 20Hz the arena should be
        updated every 1.5th frame (30/20=1.5). This is not
        possible. What will happen is that for every other
        frame the arena will be updated for each frame and
        the other it will take two frames to update. This
        will lead to a mean of 1.5 but it’s not continuous,
        of course.
        As this can easily lead to bad data being produced
        without the user knowing (no explict error will be
        thrown) this function informs the user of the
        mistake so that they can change the settings to
        either 40frames per second to keep the 20Hz update
        rate or to change the update rate.

    `start_experiment`()[source]¶
    :   This function is called at the end of the initialization of
        the `control_file.ControlTracking` class.

        It creates the folder where all the experimental data is
        being saved using a timestamp taken now.

        It then saves the “experiment\_settings.json” file which
        contains a lot of important information of the current
        experiment.

        Then it starts the detection algorithm in
        `pre_experiment.FindAnimal`.

        If the animal has been detected, the arena will be translated
        and rotated if requested using the `adjust_arena()`
        function.

        Then the tracking algorithm is called:
        `fast_tracking.FastTrackingControl`

*class* `fast_tracking.``FastTrackingControl`(*genotype='Unknown'*, *recording\_framerate=2*, *display\_framerate=None*, *resolution=None*, *recordingtime=None*, *initial\_data=None*, *boxsize=20*, *signal=None*, *frames\_to\_define\_orientation=5*, *debug\_mode=None*, *debug\_mode\_resize=1*, *repair\_ht\_swaps=True*, *cam=None*, *dir=None*, *pixel\_per\_mm=None*, *model\_organism='Not in List'*, *vr\_arena=None*, *pwm\_object=None*, *time\_dependent\_file=None*, *high\_power\_led\_bool=False*, *offline\_analysis=False*, *minimal\_speed\_for\_moving=0.5*, *organisms\_and\_heuristics=None*, *post\_hoc\_tracking=False*, *datetime=None*, *output\_channel\_one=[]*, *output\_channel\_two=[]*, *output\_channel\_three=[]*, *output\_channel\_four=[]*, *simulated\_online\_analysis=False*, *overlay\_bool=False*, *controller=None*, *time\_delay\_due\_to\_animal\_detection=0*, *vr\_update\_rate=1*, *pwm\_range=100*, *video\_filename='test.yuv'*, *pts\_filename='pts\_test.txt'*, *pi\_time\_filename='system\_time\_test.txt'*, *vr\_stim\_location='NA'*)[source]¶
:   This class controls the tracking algorithm.

    It was necessary to create a second class as the ‘record\_video’
    function of picamera needed it’s own class to deliver images to.

    This script needs to be cleaned up.

    I’m sure I can get rid of quite a bit of variables or at least
    quite a bit of variable passing around!

    `after_tracking`()[source]¶
    :   When live tracking is done, the GPIOs must be turned off.

        Then save the data that was just collected by calling the
        function ‘save’ in tracking\_help\_classes.

    `error_message_func`(*error\_stack*)[source]¶
    :   This function is called if the recording can not continue
        until the end as defined by framerate \* recording\_length.

        It will write the error into a file called “DATE-ERROR.txt”
        and put it in the experimental folder.

    `offline_analysis_func`()[source]¶
    :   This function is called when the user selects either the
        “Tools->Analysis->Single Animal tracking” or the
        “Debug->Simulate Online Tracking” option. It calls the
        identical animal tracking function as the live version,
        the only difference being the way the images are being provided.

        While in the live version, the images are streamed from the
        camera, in the simulated online version the images are
        provided as a numpy array.

    `on_closing`()[source]¶
    :   Function to use when the user clicks on the X to close the
        window.

        This should never be called in a live experiment as there is
        simply no option to click to close a window.

        Will ask if user wants to quit the experiment.
        Will save the experiment so far

    `run_experiment`()[source]¶
    :   This function is called during live tracking on the PiVR.

        Essentially, it start to record a video but provides a custom
        output. See here.

        The video records frames in the **YUV** format. See here.
        for explanation of that particular format.

        YUV was chosen as it encodes a greyscale version of the image
        (the Y’ component) at full resolution (e.g. 307‘200bytes for
        a 640x480) image while the U and the V component, which
        essentially encode the color of the image only have a quarter
        of the resolution (e.g. 76‘800bytes for a 640x480 image). As
        the color is anyway discarded, this allows a more efficient
        usage of the Raspberry Pi’s buffer compared to using,
        for example RGB.

*class* `fast_tracking.``FastTrackingVidAlg`(*genotype='Unknown'*, *recording\_framerate=2*, *display\_framerate=None*, *resolution=None*, *recordingtime=None*, *initial\_data=None*, *boxsize=20*, *signal=None*, *frames\_to\_define\_orientation=5*, *debug\_mode=None*, *debug\_mode\_resize=1*, *repair\_ht\_swaps=True*, *cam=None*, *dir=None*, *pixel\_per\_mm=None*, *model\_organism='Not in List'*, *vr\_arena=None*, *pwm\_object=None*, *time\_dependent\_file=None*, *high\_power\_led\_bool=False*, *offline\_analysis=False*, *minimal\_speed\_for\_moving=0.5*, *organisms\_and\_heuristics=None*, *post\_hoc\_tracking=False*, *datetime=None*, *output\_channel\_one=[]*, *output\_channel\_two=[]*, *output\_channel\_three=[]*, *output\_channel\_four=[]*, *simulated\_online\_analysis=False*, *overlay\_bool=False*, *controller=None*, *time\_delay\_due\_to\_animal\_detection=0*, *vr\_update\_rate=1*, *pwm\_range=40000*, *video\_filename='test.yuv'*, *real\_time=None*, *i\_tracking=None*, *total\_frame\_number=10*, *search\_boxes=None*, *image\_raw=None*, *image\_thresh=None*, *image\_skel=None*, *bounding\_boxes=None*, *centroids=None*, *midpoints=None*, *length\_skeleton=None*, *tails=None*, *heads=None*, *endpoints=None*, *ht\_swap=None*, *stimulation=None*, *heuristic\_parameters=None*, *time\_remaining\_label=None*, *child\_canvas\_top\_left=None*, *child\_canvas\_top\_middle=None*, *child\_canvas\_top\_right=None*, *child=None*, *loop\_time\_measurement=None*, *canvas\_width=None*, *canvas\_height=None*, *below\_detected=None*, *pause\_debug\_var=None*, *vr\_stim\_location='NA'*)[source]¶
:   This class takes either a camera object (so far only from the
    RPicamera) or images in a 3D numpy array (y,x and time). When run
    on the RPi it is assumed it’s running a live experiment. The camera
    framerate will be set to the framerate the user want (if user
    asks for higher framerate than the camera can give the program will
    throw an error directly in the GUI). The camera will then deliver
    each image into an in-memory stream. The images will then be
    formatted to be in 2D with the right resolution.

    (For future improvement: To increase speed one could only take
    the bytes that are actually needed (we do have the search\_box)).

    `animal_tracking`()[source]¶
    :   Main function in single animal tracking. After detection in
        `Pre-Experiment()` of the animal this function will be
        called on each frame to:

        > 1. Identify the animal,
        > 2. Define where to look for the animal in the next frame
        > 3. Define head, tail, centroid and midpoint
        > 4. If requested, present a stimulus by changing the
        >    dutycycle on the requested GPIO

        Below a the list in a bit more detail:

        1. Ensure that the search box is not outside the image.
        2. Subtract the current search box image from the background
           search box.
        3. Calculate the threshold to binarize the subtracted image.
        4. Use the regionprops function of the scikit-image library
           to find blobs
           http://scikit-image.org/docs/dev/api/skimage.measure.html#skimage.measure.regionprops
        5. Select the largest blob as the animal
        6. Define the NEXT Search Box
        7. Save the current bounding box, centroid position and the
           raw image.
        8. Skeletonize the binary image and find the endpoints.
        9. By comparing the endpoint positions to the position of the
           previous tail position, assign the closer endpoint as the
           the tail.
        10. If virtual reality experiment: Use the head position to
            define position in virtual space and update stimulus in
            Channel 1 accordingly using a change in dutycycle of the
            GPIO.
        11. If time dependent stimulus: Update the dutycyle for all
            the defined channels.

    `close`()[source]¶
    :   Unsure if needed. Test if can do without

    `error_message_func`(*error\_stack*)[source]¶
    :   Let user know that something went wrong!
        :return:

    `flush`()[source]¶
    :   Unsure if needed. Test if can do without

    `update_debug`()[source]¶
    :   This will only work in post-hoc analysis, NOT on the
        Raspberry Pi. In principle we could implement a ton more
        information, specifially we can always print:
        1) filled area
        2) eccentricity
        3) major over minor axis
        Might be good for visualization, but these parameters are
        anyway saved if the user wants them.

    `update_pwm_dutycycle_time_dependent`(*previous\_channel\_value*, *output\_channel\_list*, *output\_channel\_name*)[source]¶
    :   A convenience function for the timedependent stimulation.
        Takes the list with the gpios for a given channel, and,
        in a for loop, updates gpios according to a given channel.
        In the first iteration of the loop it will just set the pwm
        dutcycle according to whatever dutycycle is specified.
        As this function is called as ‘previous\_channel\_x\_value
        = update\_pwm\_dutcycle..’ it then updates the
        previous\_channel\_x\_value for the next iteration.
        :param previous\_channel\_value: As the GPIO dutcycle should
        only be updated when the value changes, this holds the
        previous value
        :param output\_channel\_list: list of gpio for a given channel,
        e.g. GPIO 17 would be [[17,1250]] (1250 is the frequency,
        not used here)
        :param output\_channel\_name: the channel as a string, e.g. ‘Channel 1’
        :return:

    `write`(*buf*)[source]¶
    :   This function is called by the Custom output of the
        picamera video recorder.
        and (1) prepares the image for the tracking algorithm and (2)
        calls the the tracking function: `animal_tracking()`.

        **Image preparation**

        1. Receive the buffer object prepared by the GPU which
           contains the YUV image and put it into an numpy array in
           uint8 number space.
        2. Shorten the array to the Y values. As currently only
           640x480px images can be used the array is shortened to
           307‘200bytes (from 460‘800byes)
        3. The image, which so far has just been a 1D stream of uint8
           values is then organized into the 2D image.
        4. Save the (GPU -> real time) timestamp of the current frame.
        5. Call the `animal_tracking()` function.

##### Detection and Tracking Helpers¶

*class* `tracking_help_classes.``FindROI`(*regionproperties*, *boxsize*, *size\_factor*, *image*)[source]¶
:   This class is used to define the region of interest from a given
    regionproperties class.

    It also makes sure that the box is never outside of the frame.

*class* `tracking_help_classes.``MeanThresh`(*image*, *signal*, *sigma*, *roi=None*, *invert=False*)[source]¶
:   This class takes an image and calculates the mean intensities and
    standard deviation to calculate a threshold which can be use to
    segment the image.

    If no roi is given, the take whole image is taken into account.

    roi must be a roi class object

    `calculate_threshold`()[source]¶
    :   Calculate threshold by:
        Depending on animal signal subtracting (white) or adding (dark)
        the mean of the pixel intensities in the ROI
        with a sigma (provided when class is called)
        times the standard deviation of the pixel intensities in the ROI

*class* `tracking_help_classes.``CallImageROI`(*image*, *roi*, *boxsize=None*, *sliced\_input\_image=None*)[source]¶
:   This class consolidates the different calls to provide the ROI of
    the animal in a single class.

    Different frames of references are being used in the detection
    and tracking algorithm: The absolute pixel coordinates and the
    search\_box.

    In different parts of the code different frames of references are
    used to call the image ROI:

    > 1. If only the image and the roi are given, the roi
    >    coordinates are given in the absolute frame of reference (
    >    the 640x480 pixels of the image).
    > 2. If the boxsize parameter is given, the input image is
    >    **not** the full image. Instead, it is only the image of
    >    the search box, defined by the boxsize parameter! This is
    >    for example called in
    >    `pre_experiment.FindAnimal.animal_after_box_mode_three()`
    > 3. If the slice\_input\_image parameter is given, the input
    >    image is **not** the full image. Instead, it is only the
    >    image of the search box. In
    >    `fast_tracking.ReallyFastTracking.animal_tracking()`
    >    the algorithm
    >    only looks for the animal in a region defined by search\_boxes.
    >    When providing the sliced\_input\_image this is taken into
    >    account.

    In order to keep the code as tidy as possible this class will
    help calling the ROI using an roi object

    `call_image`()[source]¶
    :   Depending on the input (full image, only search box) the
        ROI of the image is extracted.

*class* `tracking_help_classes.``CallBoundingBox`(*image*, *bounding\_box*)[source]¶
:   This class is heavily used in the
    `fast_tracking.ReallyFastTracking.animal_tracking()` function!

    It takes the full image and search\_boxes coordinates and returns
    only the search\_box (or ROI) of the image.

*class* `tracking_help_classes.``DescribeLargestObject`(*regioproperties*, *roi*, *boxsize=None*, *animal\_like=False*, *filled\_area\_min=None*, *filled\_area\_max=None*, *eccentricity\_min=None*, *eccentricity\_max=None*, *major\_over\_minor\_axis\_min=None*, *major\_over\_minor\_axis\_max=None*)[source]¶
:   This class takes a skimage regionprops object (
    https://scikit-image.org/docs/dev/api/skimage.measure.html#skimage.measure.regionprops)

    These regionprops objects have a list labelled image regions.

    If “animal\_like” is False, the largest labelled image region (
    defined by filled\_area) is defined as the animal.

    If “animal\_like” is True, each labelled image region checked
    against the following parameters taken from
    “available\_organisms.json”

    > 1. A certain range of filled area
    > 2. A certain ratio of the long\_axis over the short\_axis
    > 3. A certain range of eccentricity

    This class analyzes a binary image and defines the largest object and saves it’s bounding box,
    its major and minor axis and its centroid coordinates

    `animal_like_object`()[source]¶
    :   This function tests each labelled image region for “animal
        likeness”.

        The largest of these labelled image regions is defined as the
        animal

    `largest_object`()[source]¶
    :   This function is just defining the largest labelled image
        region as the animal.

*class* `tracking_help_classes.``DrawBoundingBox`(*image*, *roi*, *value*)[source]¶
:   Used only during debug mode when user can see the tracking
    algorithm in action.

    Indicates the ROI (search box) where the algorithm has detected
    the animal.

    `draw_box`()[source]¶
    :   Draws the bounding box directly into the numpy array

*class* `tracking_help_classes.``Save`(*heads*, *tails*, *centroids*, *image\_skel*, *image\_raw*, *image\_thresh*, *background*, *real\_time*, *pixel\_per\_mm*, *bounding\_boxes*, *midpoints*, *stimulation=None*, *arena=None*, *heuristic\_data=None*, *datetime=None*, *time\_delay\_due\_to\_animal\_detection=0*, *loop\_time=None*, *recording\_time=None*, *framerate=None*, *time\_dep\_stim\_file=None*)[source]¶
:   Used to Save experimental data after the experiment concluded -
    for both the case where the experiment finished as expected and
    also if crashed!

##### DefineOutputChannels¶

*class* `output_channels.``DefineOutputChannels`(*path*, *controller*)[source]¶
:   Let user define which output channel (GPIO18, GPIO17 etc…)
    corresponds to which Channel (1,2 etc..)

    The Raspberry Pi has a number of addressable GPIOs. PiVR currently
    uses 4 of them: GPIO18, GPIO17, GPIO27 and GPIO13. The software
    has a total of 6 output channels: Background, Background 2,
    Channel 1, Channel 2, Channel 3 and Channel 4. The user therefore
    has to decide which GPIO# is addressed by which channel.

    Warning

    Only GPIO18 and GPIO13 are capable of hardware PWM.
    The other GPIOs are limited to a maximum frequency of 40‘000Hz

    Warning

    The transistor on the PCB has a finite rise and fall
    time. In theory the transistor should be able to be turned on
    and off every us (10e-6 seconds) which translates to 1
    Million Hz (10e6). This will not enable the usage of PWM to
    control light intensity, however. For example, if a
    dutycycle of 10% is chosen, it will lead to the transistor
    being on for only 10% of 1 us, which will lead to
    unspecified behavior. We usually use 40‘000Hz even on the
    high speed GPIOs.

    Background and Background 2 are intended to be used as constant
    light sources during a recording. Typically one of the two will
    be used to control illumination for the camera to record in optimal
    light conditions. As PiVR normally uses infrared light to
    illuminate the scene many animals wont be able to see at this
    wavelength. If the experimenter wants to use light of a
    wavelength that the animal can see (or white light) while using
    infrared illumination for the camera, the other background
    channel can be used.

    Channels 1, 2, 3 and 4 are addressable during a recording.
    Channel 1 will always be used for Virtual Arenas. The other
    channels are only useful for time dependent stimulation. In
    principle each GPIO can have its own Channel. This is only
    useful if illumination (via background, see above) is optimal
    without fine grained control.

    `cancel`()[source]¶
    :   Function is called when user presses the ‘cancel’ button.
        Destroys the window without saving anything.

    `confirm`()[source]¶
    :   Function is called when user presses the ‘confirm’ button.
        Collects the channels and frequencies and associates it with
        the proper variable.

        Specifically it creates one list per channel. Each GPIO in
        that channel is a nested list. For example, if the user
        assigns GPIO27 and GPI17 to Channel 1 (with frequency 1250),
        the channel\_one variable will be a nested list in the
        following form: [[27, 1250][17,1250]]

        Variables are modified in the instance of the original GUI

    `gpio13_high_speed`()[source]¶
    :   Function is called when user uses checkbutton High Speed for
        GPIO13. This updates the window for the user to either
        manually enter a frequency (if High Speed PWM is On) or use
        the list of available frequencies (not High Speed PWM). In
        principle identical to the gpio18\_high\_speed function

    `gpio18_high_speed`()[source]¶
    :   Function is called when user uses checkbutton High Speed for
        GPIO18. This updates the window for the user to either
        manually enter a frequency (if High Speed PWM is On) or use
        the list of available frequencies (not
        High Speed PWM)

##### Error Messages¶

`tracking_help_classes.``show_vr_arena_update_error`(*recording\_framerate*, *vr\_update\_rate*)[source]¶
:   This function warns the user that an incompatible framerate/dynamic
    arena update frequency has been chosen.

    For example, if the framerate is 30frames per second and
    the update rate is 10Hz the arena will be updated every 3rd
    frame (30/10=3). This is of course possible.

    If the framerate is 30frames per second and the update rate is
    set to 20Hz the arena should be updated every 1.5th frame (
    30/20=1.5). This is not possible. What will happen is that for
    every other frame the arena will be updated for each frame and
    the other it will take two frames to update. This will lead to a
    mean of 1.5 but it’s not continous, of course.

    As this can easily lead to bad data being produced without the
    user knowing (no explict error will be thrown) this function
    informs the user of the mistake so that they can change the
    settings to either 40frames per second to keep the 20Hz update
    rate or to change the update rate.

#### Virtual Arena drawing¶

*class* `VR_drawing_board.``VRArena`(*resolution=None*, *path\_of\_program=None*)[source]¶
:   Users need to be able to “draw” virtual reality arenas as many
    users will not be able to just pull up matlab or python to draw a
    virtual arena as a 2D matrix and save it as csv.

    This class is intended to let users draw virtual realities
    painlessly.It opens a blank image with the x/y size of the camera
    resolution. It lets the user ‘draw’ a VR arena with the mouse.

    In general it has two options: One can draw gaussian circles
    while being able to define sigma.

    The user also has the option to draw step functions without a
    gradient at the edge. In both of those geometric objects the user
    can define the intensity. It is also possible to define a general
    minimal stimulation.

    See the subclasses for detailed information
    It then saves the arena (probably in a subfolder) to be used
    again. It should also be saved with any experiment that is
    conducted with it

    `addition_of_intensity_func`()[source]¶
    :   A callback function for the button ‘additive on/off’.
        The user can toogle between adding intensities and not

    `delete_animal_func`()[source]¶
    :   If the user wants to get rid of the animal after drawing
        one,they can press this button. It will just give the string
        ‘NA’ to all the animal position variables which tells the
        program that no animal has been selected.

    `dont_draw_func`()[source]¶
    :   If the user first wants to draw and then e.g. place an animal
        or zoom into a part of the figure, they need to first call
        this function by pressing the so called button. It will
        disconnect the event handler.

    `draw_gaussian_func`()[source]¶
    :   This function call the GaussianSlope class - just here to
        save lines in the program.
        Also changes the last\_drawing\_call variable to make the user
        experience more intuitive.

    `draw_linear_func`()[source]¶
    :   This function calls the Step class - just to save some lines
        in the code.
        Also changes the last\_drawing\_call variable to make the user
        experience more intuitive.

    `gridlines_func`()[source]¶
    :   When the user presses the Gridlines button, this function
        turns them on or off.

    `invert_func`()[source]¶
    :   This function is bound to the invert\_button.
        It changes the text on the invert\_button,
        it also changes the color of both the background and the
        text of the button for visual help it also changes the
        boolean invert variable to True or False and it also updates
        the drawing class call.

    `modify_exsiting_func`()[source]¶
    :   This function first opens a filedialog with the directory
        that this module saves the arenas normally.
        It then reads the file and directly draws in on the canvas.
        It also updates the name with the name of the file selected.

    `overwrite_func`()[source]¶
    :   This function is bound to the overwrite\_button.
        It changes the text on the overwrite button,
        it also changes the boolean variable ‘overwrite’ to True or
        False and it updates the drawing class call

    `place_animal_func`()[source]¶
    :   After calling this function by pressing the appropriate
        button, the user is able to draw in the canvas where the
        animal shall be located.
        If mouse press and release are at the same position, a circle
        will be drawn. If not, an arrow will be drawn. The (inverted)
        arrowhead indicates the position of the animal at the beginning
        of the expriment while the other side indicates the angle the
        animal was last seen.

    `place_animal_precise_func`()[source]¶
    :   This function calls the PlaceAnimal class which will either
        draw a circle (if only x and y are given) or an arrow (if x,
        y and theta are given).
        Before it does so it tries to set any existing arrows invisible.
        It also changes a button color and changes the boolean switch
        ‘animal\_draw\_selected’.

    `precise_gaussian_draw_func`()[source]¶
    :   In order to precisely draw a gaussian gradient, the user has
        the option of defining the x/y coordinate and then pressing
        a button. It then calls this function which will give the
        precise x/y coodinates (along with all the other arguments
        that are called when drawing by mouse) to the GaussianSlope
        Class.

    `precise_rectangle_draw_func`()[source]¶
    :   In order to precisely draw a step rectangle, the user has the
        option of defining the x/y coordinate of the center and then
        pressing a button. It then calls this function which will
        give the precise x/y coordinates along with all the other
        arguments that are called when drawing by mouse) to the Step
        Class.

    `quit_func`()[source]¶
    :   In order to quit this window and go back to the main GUI,
        the user needs to press the ‘quit’ button and this function
        will be called.

    `save_arena`()[source]¶
    :   This function is called when the user clicks the ‘save’ button.
        The function should work both on Linux based systems and
        Windows.
        It checks whether the file already exists and asks the user
        if it should be overwritten if it exits.
        Otherwise it just saves, without any confirmation etc.
        The areas are always saved with the resolution as they are
        not interchangable.

    `stop_animal_drawing_func`()[source]¶
    :   After placing an animal with the mouse the user might want to
        draw more or just zoom into a part of the figure.
        Pressing the appropriate button will call this function
        which disconnects the event handler.

    `update_animal_position`(*x*, *y*, *angle*)[source]¶
    :   After drawing either a circle or an arrow, the PlaceAnimal
        Class calls this function to let the main class know what the
        x/y and theta of the latest animal was
        :param x: x coordinate of the animal
        :param y: y coordinate of the animal
        :param angle: the angle (calculated by arctan2 function) that
        describes where the animal was before the start of the
        experiment

    `update_drawing`()[source]¶
    :   If it is not clear on which geometric form the user is
        working, this function is called (e.g. when changing the
        ‘overwrite’ button or the ‘invert’ button

    `update_values`()[source]¶
    :   This funcion runs as a loop in the background after the VR
        drawing board has been constructed. It listens to changes in
        the Entry fields by the user and calls appropriate functions.

    `arena` *= None*¶
    :   CENTER
        call the plotting library and plot the empty arena into a figure

*class* `VR_drawing_board.``GaussianSlope`(*ax*, *arena*, *plot\_of\_arena*, *size*, *sigma*, *max\_intensity*, *overwrite*, *invert*, *addition\_of\_intensity*, *mouse\_drawing*, *x\_coordinate=None*, *y\_coordinate=None*)[source]¶
:   This class is bound to the canvas.

    There are two ways this class can behave:

    1. When the user clicks somewhere on the canvas, the x and y
       coordinates are collected using the
       `GaussianSlope.on_press()` function. This function then
       calls the `GaussianSlope.draw_gradient()` function. In
       that function the gaussian gradient with the entered size is
       created. Then the size of the gaussian gradient arena is
       matched to the size of the image which is given by the
       resolution and plotted. This makes for a interactive experience
       for the user who can ‘point and click’ on the area where a
       gaussian gradient should be created.
    2. If the “Draw Gaussian Circle at defined coordinates” buttons
       is pressed, the x and y coordinate are collected from the
       “Coordinates” entry boxes. Then the
       `GaussianSlope.draw_gradient()` is called and the gradient
       is created identical to the mouse click version.

    By varying the size of the gaussian gradient it is also possible
    to have more than one gradient. If only one gradient is to be
    created the user should just leave the original setting in place
    (2000) as this is way larger than the resolution used to record
    the behavior. By varying sigma the user can choose the steepness
    of the gradient

    `disconnect`()[source]¶
    :   Disconnect the mouse button clicks from the canvas

    `draw_gradient`()[source]¶
    :   This class collects the size and sigma of the gradient and
        draws it at the coordinates where the user pressed on the
        canvas.

    `gkern`(*kernlen=20*, *std=3*)[source]¶
    :   Returns a 2D Gaussian kernel array.
        Taken from: https://stackoverflow.com/questions/29731726
        /how-to-calculate-a-gaussian-kernel-matrix-efficiently-in-numpy

*class* `VR_drawing_board.``Step`(*ax*, *arena*, *plot\_of\_arena*, *size\_x*, *size\_y*, *intensity*, *overwrite*, *invert*, *addition\_of\_intensity*, *mouse\_drawing*, *x\_coordinate=None*, *y\_coordinate=None*)[source]¶
:   This class is bound to the canvas. This class is called to draw
    precise Rectangles using coordinates *and* when drawing Rectangles
    with the mouse.

    Behavior in “precise” mode:

    > Collect the x and y coordinate from the entry field

    Behavior in “mouse” mode:

    > When the user clicks somewhere on the canvas, the x and y
    > coordinates are collected `Step.on_press()`.

    The rest of the behavior is identical as the function
    `Step.draw_rectangle()` is called

    This class does essentially the same as the `GaussianSlope`
    class with the difference of not calling the gkern function.
    Source code is quite explicit and heavily annotated.

    `disconnect`()[source]¶
    :   This function is called directly from the master and
        disconnects the event handler

    `draw_rectangle`()[source]¶
    :   This function is called either after the user pressed the
        mouse button on the canvas or if the x/y coordinates have
        been entered manually.

    `on_press`(*event*)[source]¶
    :   This function just collectes the x/y coordinate of where the
        user pressed the mouse button

*class* `VR_drawing_board.``PlaceAnimal`(*ax*, *plot\_of\_arena*, *master*, *start\_x=None*, *start\_y=None*, *theta=None*, *precise=False*)[source]¶
:   This class is bound to the image that has been plotted to show
    the arena. There are three ways this class can behave:

    1. If no x/y and theta values are passed:
       When the user clicks somewhere first the x and y coordinates
       are collected. After the user has released the mouse button,
       the coordinates for the press and release are compared. If they
       are identical, the animal will not have a directionality
       which is displayed as a circle. If they are not identical,
       the point of release is seen as the point where the animal
       will be when the experiment starts. The point where the user
       pressed is the direction where the animal is coming from.
    2. If x/y but not theta are provided:
       The class will just draw a circle (no directionality of the
       animal is assumed).
    3. If x/y and theta are provided, an arrow is drawn with the
       given coordinates as the place where the animal will be when
       the experiment starts and theta as the direction where the
       animal was before.

    `disconnect`()[source]¶
    :   Disconnect all mouse buttons from canvas. Called directly from
        the master

    `draw_arrow`()[source]¶
    :   This function is called either after the mouse button is
        released and x/y at press and release are not identical
        or if the x/y and theta are provided.
        First it will try to remove the arrow or circle that is
        already present, then it’ll draw a new arrow.

    `draw_point`()[source]¶
    :   This function is called either after the mouse button is
        released and x/y at press and release are identical
        or if the x/y coordinates (but not theta) are provided.
        First it will try to remove the arrow or circle that is
        already present, then it’ll draw a new circle.

#### PiVR Analysis source code¶

*class* `analysis_scripts.``AnalysisDistanceToSource`(*path*, *multiple\_files*, *string*, *size\_of\_window*)[source]¶
:   For our lab, a typical experiment would be the presentation of an
    odor source to an animal. By analyzing the behavior, for example the
    attraction of the animal towards the source, we can learn a lot
    about the underlying biology that manifests itself in that
    behavior.

    To easily enable the analysis of such an experiment, the user has
    the option to automatically analyze these experiments. This class
    is at the heart of the analysis.

    As each experiment (across trials) can have the source at different
    positions in the image, the user is first presented with the
    background image. The user then selects the source upon which the
    distance to the source is calculated for each timepoint of the
    experiment.

    The output is a csv file with the distance to source for each
    analyzed experiment and a plot indicating the median and the
    indvidual trajectories.

*class* `analysis_scripts.``AnalysisVRDistanceToSource`(*path*, *multiple\_files*, *string*)[source]¶
:   After running a virtual reality experiment with a **single point
    source** we are often interested in the distance to this source.
    For example, when expressing the optogenetic tool Chrimson in the
    olfactory system of fruit fly larva, they will ascend a virtual
    odor gradient which is similar to real odor source.

    To easily enable the analysis of such an experiment, the user has
    the option to automatically analyze these experiments. This class
    is at the heart of the analysis.

    The user just has to select the folder containing the
    experiments. This class will automatically detect the maximum
    intensity point in virtual space and calculate the distance to
    that point for the duration of the experiment.

    The output is a csv file with the distance to the **single**
    point of maximum virtual stimulus for each analyzed experiment
    and a plot indicating the median and the indvidual trajectories.

*class* `multi_animal_tracking.``MultiAnimalTracking`(*data\_path*, *colormap*, *recording\_framerate*, *organisms\_and\_heuristics*)[source]¶
:   The Multi-Animal Tracker allows the identification and
    tracking of several animals in a video or image series.

    This tracker depends on user input, specifically:

    1. The user should identify the region in the frame where the
       animals are to be expected. This helps reduce
       mis-identification of structures outside that area as
       animals.
    2. The user should optimize the detection by using the
       ‘Treshold (STD from Mean)” slider. When doing background
       subtraction, the current image is subtracted from the mean
       image. The treshold defined using this slider defines how
       many standard deviations (e.g. 5 x Standard Deviation)
       from the mean value of pixel intensities of the subtracted
       image the animals are expected. In other words - the
       clearer your animals stand out (large contrast) the higher
       the treshold can be set.
    3. The “Minimum filled area” slider gives the user a handle
       on the animal size: After background subtraction and
       applying the threshold (see above) the algorithm goes
       through all the
       “blobs”.
       To determine whether a given blob counts as an animal it
       compares the number of fixels and compares it to this
       Minimum filled area. A blob will only count as an animal
       if it contains equal or more pixels as defined here.
    4. The “Maximum filled area” slider gives the user a handle
       on the animal size by defining the maximum area (in
       pixels) the animal has (see above).
    5. The “Major over Minor Axis” slider lets the user select
       for “elongated” objects. The Major and Minor axis are
       properties of the
       “blob”.
       For animal that are often round (such as fruit fly larva)
       it is best to keep this parameter at zero. For animals
       that are rigid such as adult fruit flies, it can be useful
       set this slider to a number higher than one.
    6. The “Max Speed Animal [mm/s]” is used during tracking to
       define realistic travelled distances between two frames.
       To calculate this, the script takes the pixel/mm and the
       framerate as recorded in “experiment\_settings.json” into
       account.

       For example, if you have a fruit fly larva that
       moves not faster than 2mm/s and you have recorded a video
       at 5 frames per second at a distance (camera to animals)
       translating to 5pixel/mm at your chosen resolution a blob
       can not move more than (2mm/s\*5pixel/mm)/5 frames per
       second = 2 pixel per frame.

       Warning

       This feature can lead to unexpected results. If your
       trajectories look unexpected, try relaxing this parameter
       (=put a large number, e.g. 200)
    7. The “Select Rectangular ROI” is a important feature: it
       allows the selection of a rectangular area using the mouse in
       the main window. When looking for animals, only the area
       inside this area is taken into consideration.
    8. The main window displays the current frame defined by pulling
       the slider next to “Start Playing”. This can be used to
       optimize the “Image parameters” described above. To just watch
       the video you can of course also press the “Start Playing”
       button.

    The multi-animal tracking algorithm critically depends on optimal
    image parameters which means that for optimal results **each
    frame should contain the expected number of animals**. For
    example, if you are running an experiment with 5 animals the goal
    is to adjust the image parameters such that for each frame you
    will have 5 animals. See here
    on how to best achieve this.

    To help the user find frames where the number of animals is
    incorrect, the button “Auto-detect blobs” can be very useful. It
    detects, in each frame, the number of
    “blobs”.
    that fit the image parameters irrespective of distance travelled.
    See `MultiAnimalTracking.detect_blobs()` for details on what
    that function is doing exactly.

    Once the user presses the “Track Animals” button, the
    `MultiAnimalTracking.ask_user_correct_animal_classification()`
    function is called. This function uses the current frame and
    applies the user defined image parameters to determine the number
    of animals used in the experiment. It then shows a popup
    indicating the blobs identified as animals and ask the user if
    this is correct.

    If the user decides to go ahead with tracking, the actual
    tracking algorithm starts. The principle of this multi-animal
    tracker is the following:

    1. User has defined the number of expected animals by choosing a
       frame (i.e. Frame # 50) where the correct number of animals
       can be identified.
    2. A numpy array with the correct space for storing X and Y
       coordinates for all these animals for each frame is
       pre-allocated
    3. In the user defined frame (i.e. Frame # 50), the position of
       each animal is identified.
    4. The centroid position for each animal is stored in the
       pre-allocated array. The order is from identified animal top
       left to bottom right. I.e. the animal that is top left in the
       image in i.e. Frame #50 will be in position #1 in the numpy
       array.
    5. As the user defined frame does not have to be the first frame,
       the tracking algorithm can run “backwards”, i.e. identifying
       animals in frame 50, 49, 48… and once it reaches zero it
       will run forward, in our example 51, 52 …
    6. In the next frame (which can also be the previous frame as the
       analysis can run backwards), the blobs that can be animals are
       again identified using animal parameters. In our example where
       the starting frame was 50, the “next” frame to be analyzed is 49.
    7. The centroids in frame 49 are assigned to the previously
       identified frame by calculating the distance of each centroid
       to each of the previously identified centroids. Centroids with
       the smallest distance are assumed to be from the same animal.
    8. In many multi-animal experiments, animal can touch each other
       which makes it impossible for the algorithm to distinguish
       them. For a frame where 2 (or more) touch each other,
       only one centroid can be assigned to the touching animals.
    9. Once the animals do not touch anymore, they can be re-idenfied
       as single animals. To assign them to their previous
       trajectory the distance to the previously known position of
       the animal that was lost before.
       However, for the time that the animal is missing,
       no assumptions are made and the data is just missing.

    `ask_user_correct_animal_classification`()[source]¶
    :   This function is called after the user presses “Track Animals”.

        1. Creates a popup window to show the current frame
        2. Subtracts the current image from the background image
        3. Thresholds (binarizes) the subtracted image with the user
           defined Treshold.
        4. Identifies all blobs in the current image by calling the
           “regionprops”.
           function.
        5. For each identified blob, determine whether it counts as
           an animal according to the user defined image parameters.
        6. If yes, draw a box around that blob.
        7. Display the resulting image and ask the user if the
           identified and numbered blobs are indeed animals and if the
           tracking algorithm should start.

        Important

        The number of animals identified here is used as the
        ‘ground truth’ of how many animals are present during the
        experiment.

    `detect_blobs`()[source]¶
    :   This function is intended to be used “pre-tracking”: If the
        user thinks the Image parameters are ok and they press
        “Detect blobs” this function is called. It checks for the
        number of blobs fitting the Image parameters for each frame.
        This will make it obvious where the image parameters are
        producing incorrect results.

        The function does the following:

        1. Subtract all images from the background image.
        2. Threshold (binarize) the subtracted image using the user
           defined Threshold Image parameter.
        3. Loop through the subtracted frames and call the
           “regionprops”.
           function on each frame.
        4. Loop through each of the blobs and determine if they are
           counting as animals, i.e. by comparing their filled area
           to the user defined minimum and maximum filled area.
        5. If they count as animals, just count how many per frame do
           count.
        6. Plot the blobs identified as animals in the plot on the
           right side of the main window.

    `draw_rectangle`()[source]¶
    :   When the user presses the “Select rectangle” Button,
        this function is called.

        It connects the mouse button press and release events.

        Call `MultiAnimalTracking.on_press()` and
        `MultiAnimalTracking.on_release()`

    `interpolate`()[source]¶
    :   During tracking it can happen that animals are not identified
        in every frame.

        This function allows to interpolate the trajectories.

        Warning

        This is an experimental feature. It can produce very wrong
        results

        For each identified animal there is “last frame” where it has
        been identified and a “new frame” where it is identified
        again. This function assumes that the animal moved with a
        constant speed and in linear fashion and just does a linear
        interpolation between these coordinates.

        Important

        An important assumption is that the initial assignment was
        relatively correct. Small errors can lead to huge effects
        when using the interpolation function

    `manually_jump_to_frame_func`()[source]¶
    :   Function is called when user presses the “Jump to frame” button.

    `on_press`(*event*)[source]¶
    :   Saves x and y position when user presses mouse button on main
        window

    `on_release`(*event*)[source]¶
    :   Saves x and y position when user releases mouse button on
        main window.

        Also takes care of updating the main window with the new ROI

    `play_func`()[source]¶
    :   Function is called when user presses the “Start playing” button.

    `quit_func`()[source]¶
    :   In order to quit this window and go back to the main GUI,
        the user needs to press the ‘quit’ button and this function
        will be called.

    `tracking_start`()[source]¶
    :   This function organizes the tracking of the animals.

        It pre-allocates the numpy array for the centroid positions
        after identifying the correct number of animals in the
        current frame.

        The actual tracking function, the tracking\_loop(), is defined
        locally in this function. the tracking\_loop() function is
        called in the correct order in here.

        If the details in the documentation of this class are not
        sufficient please have a look at the heavily annotated source
        code of tracking\_loop() function (line 1228)

    `update_overview_func`()[source]¶
    :   Function is called when user presses the “Update Overview
        Button. Just changes the bool used in
        `update_visualization()`

    `update_visualization`(*scale\_input=None*)[source]¶
    :   Updates the embedded matplotlib plots by setting the data to
        the current image\_number

#### PiVR Image Data Handling source code¶

*class* `image_data_handling.``PackingImages`(*controller*, *path*, *multiplefolders*, *folders*, *zip*, *delete*, *npy*, *mat*, *color\_mode*)[source]¶
:   After running an experiment with the full frame recording option,
    it is often problemtatic to move the folder around.

    The reason is that for the OS it is usually harder (i.e. slower)
    to move thousands of small files around compared to a single file
    with the same size.

    This class collects images and essentially creates a single file
    from them.

*class* `image_data_handling.``ConvertH264`(*path*, *multiplefolders*, *folders*, *save\_npy*, *save\_mat*, *color\_mode*, *output\_video\_format*)[source]¶
:   When recording a video using PiVR there seems to be a problem
    with the encoder: Some of the metadata is not correctly stored,
    most importantly the framerate is usually given as ‘inf’.

    This class enables the user to convert the recorded h264 video to
    another video format. This happens by completely decoding the video

PiVR has been developed by David Tadres and Matthieu Louis (Louis Lab).

### FAQ¶

#### Distorted Images¶

*Question:*
:   Why do my images/videos look distorted?

*Answer:*
:   Every lens will introduce radial distortions to the image.
    Since the Raspbbery Pi Camera lens is not a high quality lens the
    radial distortion can become very obvious. The distortion is a
    function of the lens you are using meaning the distortion is
    identical for all videos/images taken by the same camera.

    I have composed a number of script that should help with finding
    the relevant coefficient and then ‘undistort’ videos and images.
    Please check out
    this repository

#### Progress Bar¶

*Question:*
:   When running a tracking/virtual reality/video experiment on PiVR, why
    is there no progress bar?

*Answer:*
:   PiVR was designed to process each frame as quickly as it can. This is
    necessary to produce realistic virtual realities. Having a progress
    bar undermines this goal as the act of updating the progress bar
    increase latency.

    @ Developers: If you think this problem can be solved, please
    let us know - if there
    is a way to update a progress bar in less than a
    millisecond, this could and should be implemented.

PiVR has been developed by David Tadres and Matthieu Louis (Louis Lab).

### Contact¶

#### Questions?¶

If something does not work as you expect, one of the best ways to get
information is to open an “issue” on the PiVR gitlab repository.

#### Found a Bug?¶

Please open an “issue” on the PiVR gitlab repository. It is imperative to
give as much information in when you encountered the bug. Ideally,
you are able to let us know exactly how to reproduce the bug. At
least you need to let us know:

1. The OS (on the Raspberry Pi it is probably Raspbian)
2. What exactly you were doing when the error occured.

#### Other questions (scientific, technological, copyright…)¶

Please contact the corresponding author of this work, Matthieu Louis.

---

© Copyright 2019 The Regents of the University of California. All rights reserved. Software authored by David Tadres and Matthieu Louis
Revision `9c4a7f2f`.

Built with Sphinx using a theme provided by Read the Docs.

Read the Docs
v: latest

Versions
:   latest
:   stable

Downloads
:   pdf
:   html
:   epub

On Read the Docs
:   Project Home
:   Builds

---

Free document hosting provided by Read the Docs.
