# Supplementary figures and images for: PiVR: An affordable and versatile closed-loop platform to study unrestrained sensorimotor behavior (part 2 of 2)
Source: PLoS Biol. 2020 Jul 14;18(7):e3000712. doi: 10.1371/journal.pbio.3000712 (PMC7360024; doi:10.1371/journal.pbio.3000712)

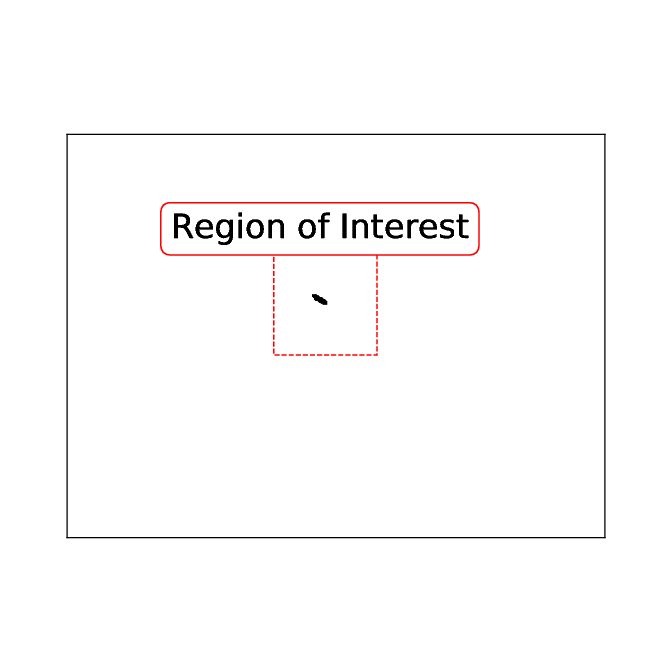

Supplement: S1 HTML — PiVR, Raspberry Pi Virtual Reality. (ZIP) [file pbio.3000712.s019.zip › S1HTLM/_images/Mode3ROIthresh.png]

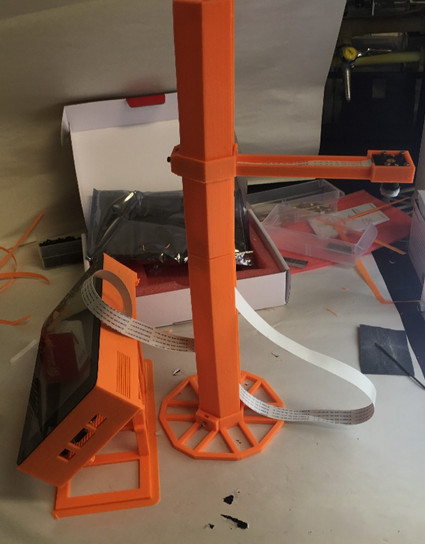

Supplement: S1 HTML — PiVR, Raspberry Pi Virtual Reality. (ZIP) [file pbio.3000712.s019.zip › S1HTLM/_images/19_PiVR_complete.jpg]

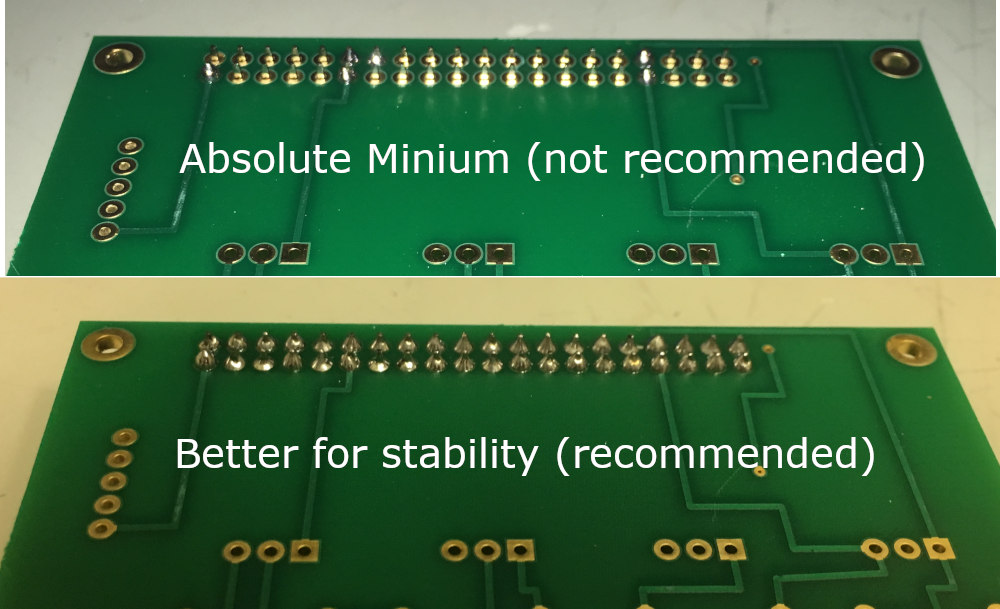

Supplement: S1 HTML — PiVR, Raspberry Pi Virtual Reality. (ZIP) [file pbio.3000712.s019.zip › S1HTLM/_images/S_5.jpg]

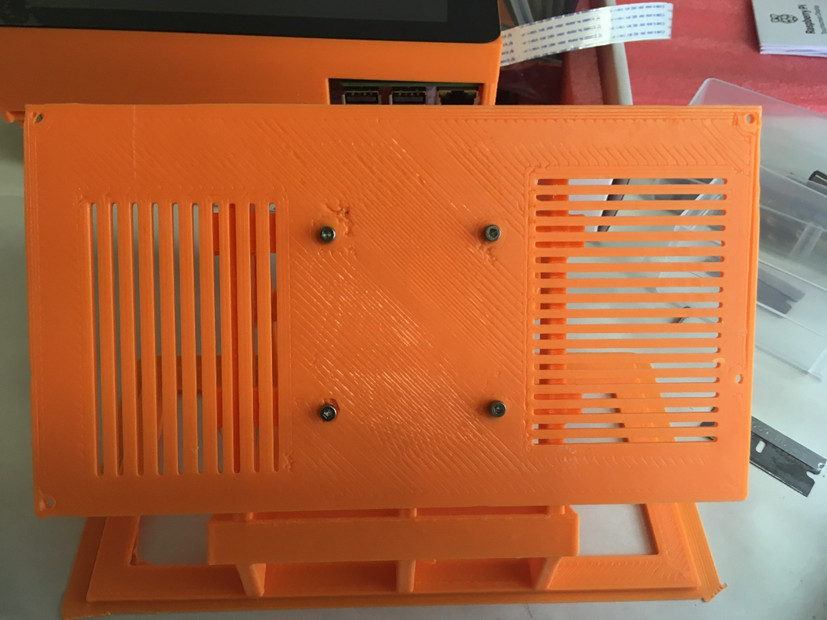

Supplement: S1 HTML — PiVR, Raspberry Pi Virtual Reality. (ZIP) [file pbio.3000712.s019.zip › S1HTLM/_images/13_Backside_attached_to_Pedestal.jpg]

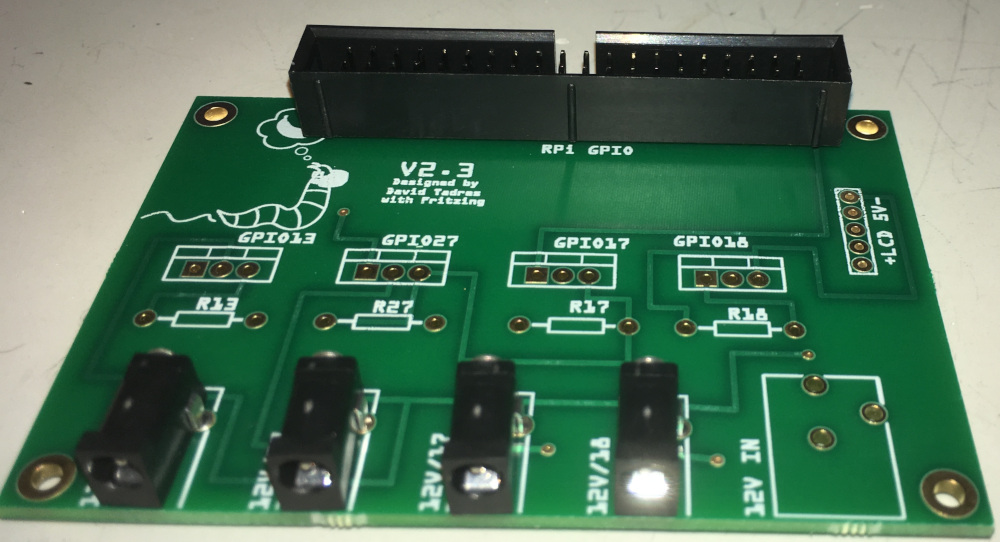

Supplement: S1 HTML — PiVR, Raspberry Pi Virtual Reality. (ZIP) [file pbio.3000712.s019.zip › S1HTLM/_images/S_4.jpg]

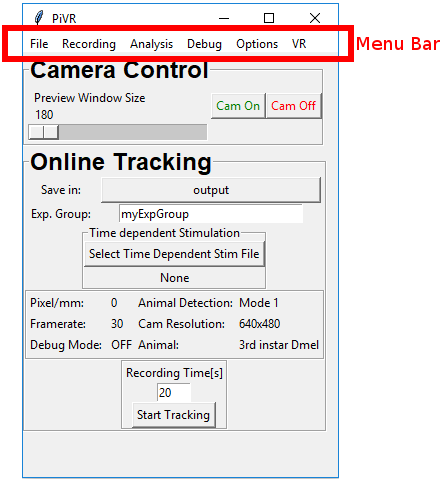

Supplement: S1 HTML — PiVR, Raspberry Pi Virtual Reality. (ZIP) [file pbio.3000712.s019.zip › S1HTLM/_images/Menubar.png]

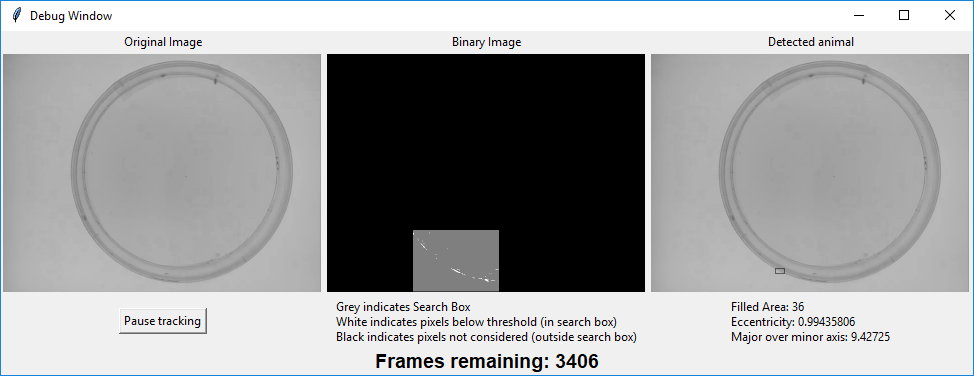

Supplement: S1 HTML — PiVR, Raspberry Pi Virtual Reality. (ZIP) [file pbio.3000712.s019.zip › S1HTLM/_images/15_simulated_tracking_broken.png]

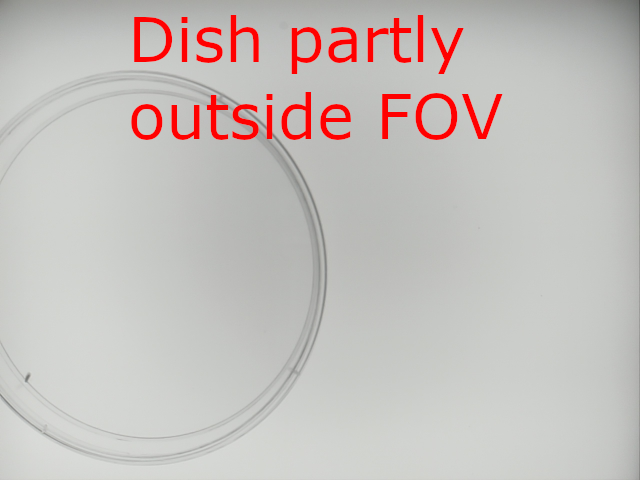

Supplement: S1 HTML — PiVR, Raspberry Pi Virtual Reality. (ZIP) [file pbio.3000712.s019.zip › S1HTLM/_images/ExampleOutsideFOV.png]

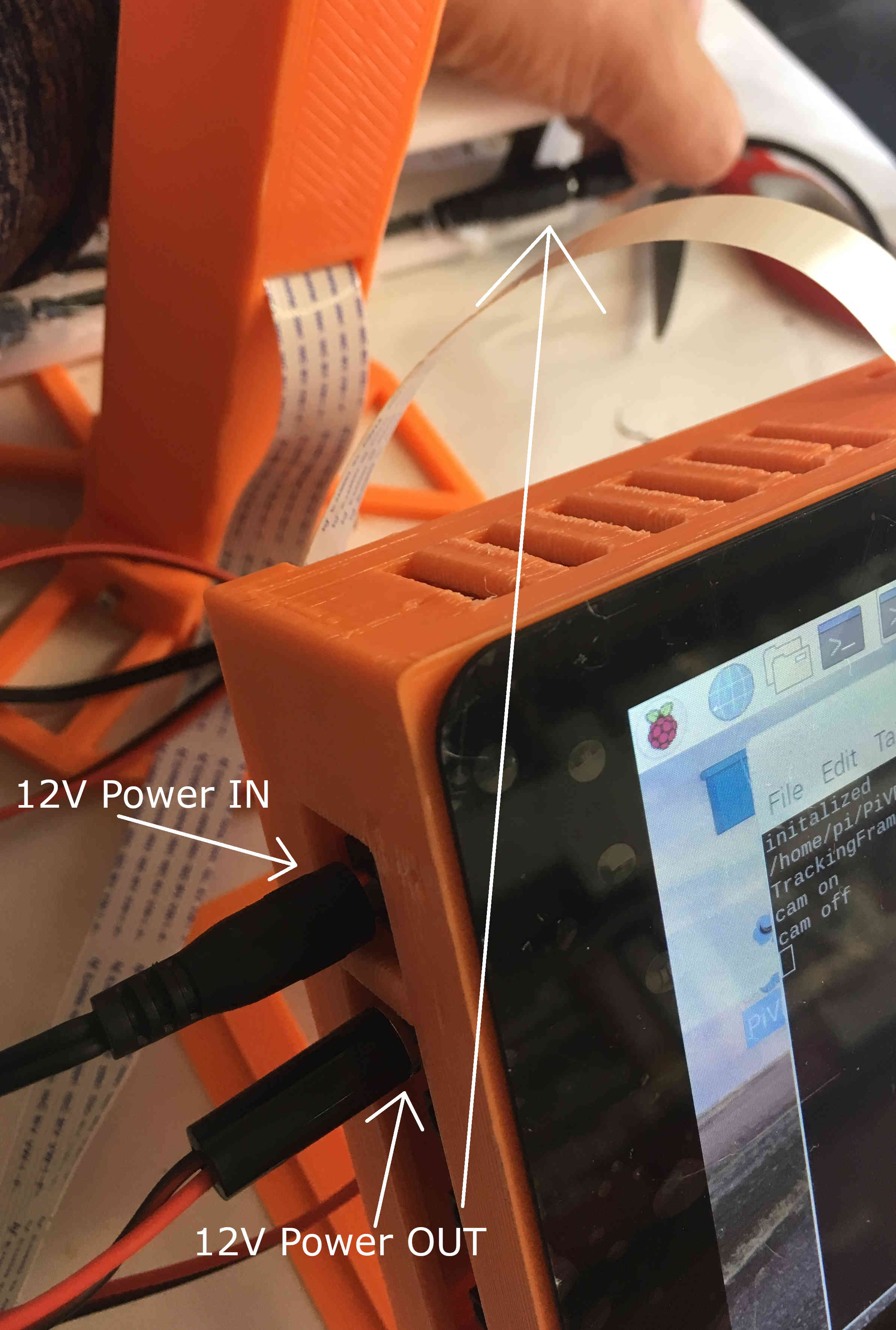

Supplement: S1 HTML — PiVR, Raspberry Pi Virtual Reality. (ZIP) [file pbio.3000712.s019.zip › S1HTLM/_images/30_connecting_IR_cables.jpg]

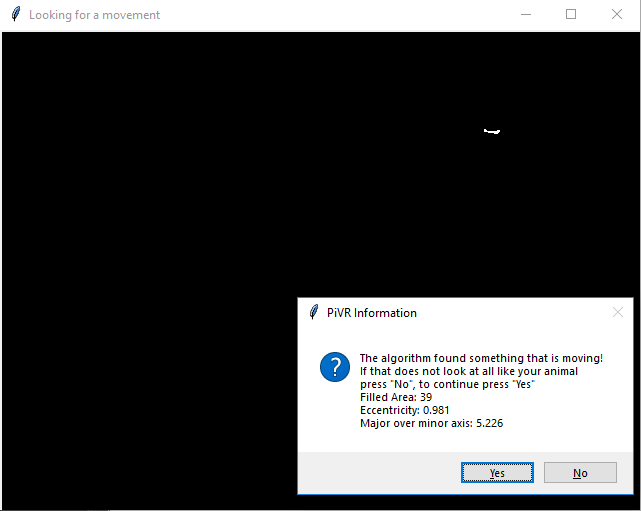

Supplement: S1 HTML — PiVR, Raspberry Pi Virtual Reality. (ZIP) [file pbio.3000712.s019.zip › S1HTLM/_images/13_simulate_tracking_detection.png]

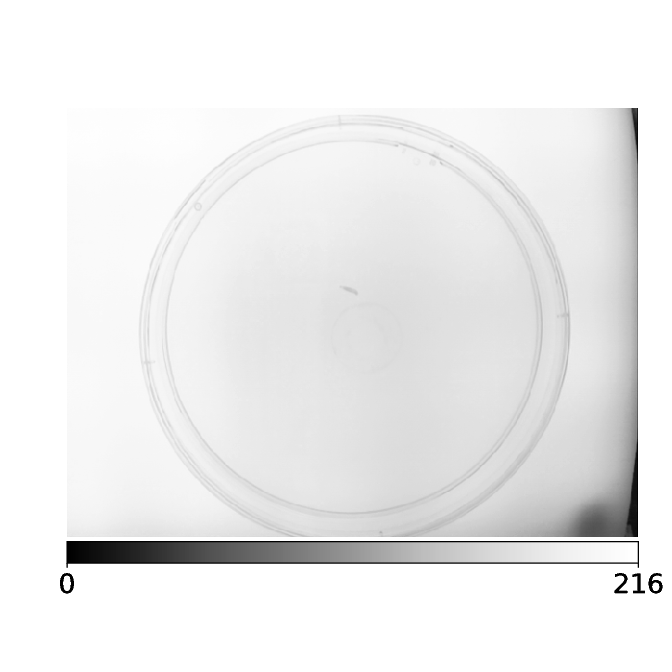

Supplement: S1 HTML — PiVR, Raspberry Pi Virtual Reality. (ZIP) [file pbio.3000712.s019.zip › S1HTLM/_images/Mode3SecondImage.png]

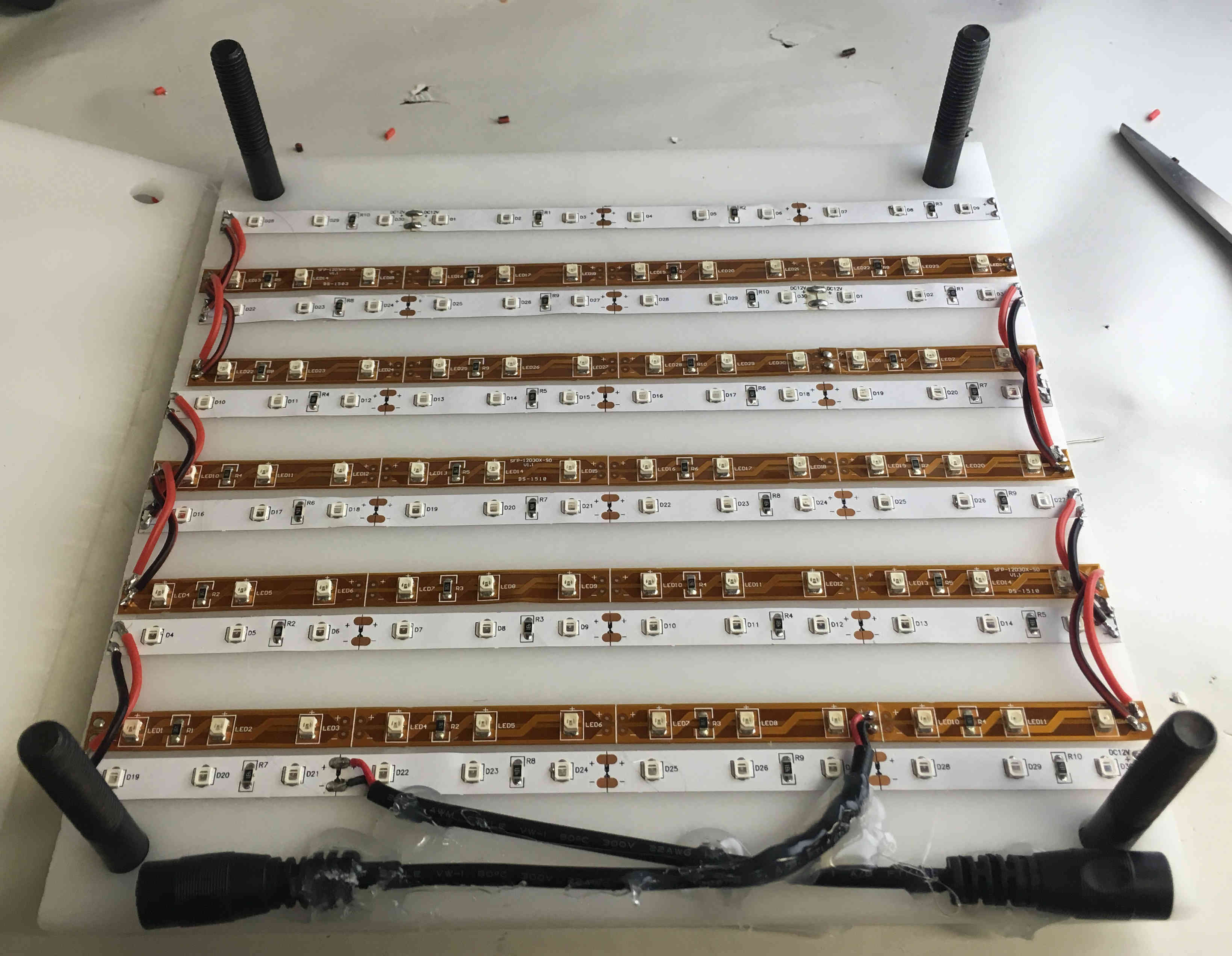

Supplement: S1 HTML — PiVR, Raspberry Pi Virtual Reality. (ZIP) [file pbio.3000712.s019.zip › S1HTLM/_images/25_arena_second_strip.jpg]

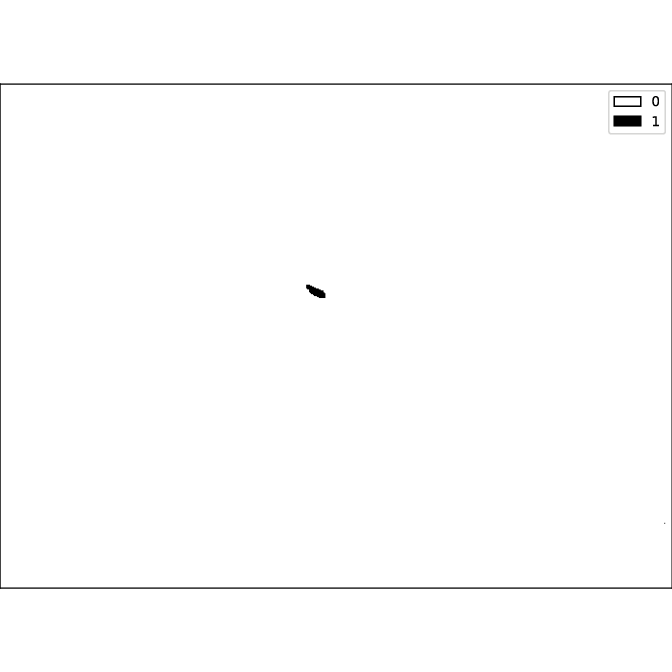

Supplement: S1 HTML — PiVR, Raspberry Pi Virtual Reality. (ZIP) [file pbio.3000712.s019.zip › S1HTLM/_images/Mode3Binary.png]

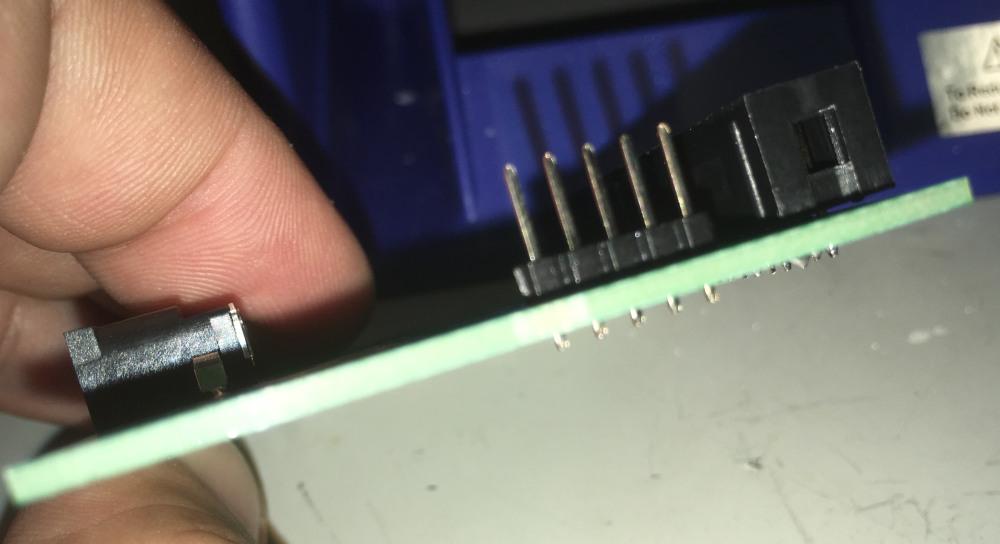

Supplement: S1 HTML — PiVR, Raspberry Pi Virtual Reality. (ZIP) [file pbio.3000712.s019.zip › S1HTLM/_images/S_6.jpg]

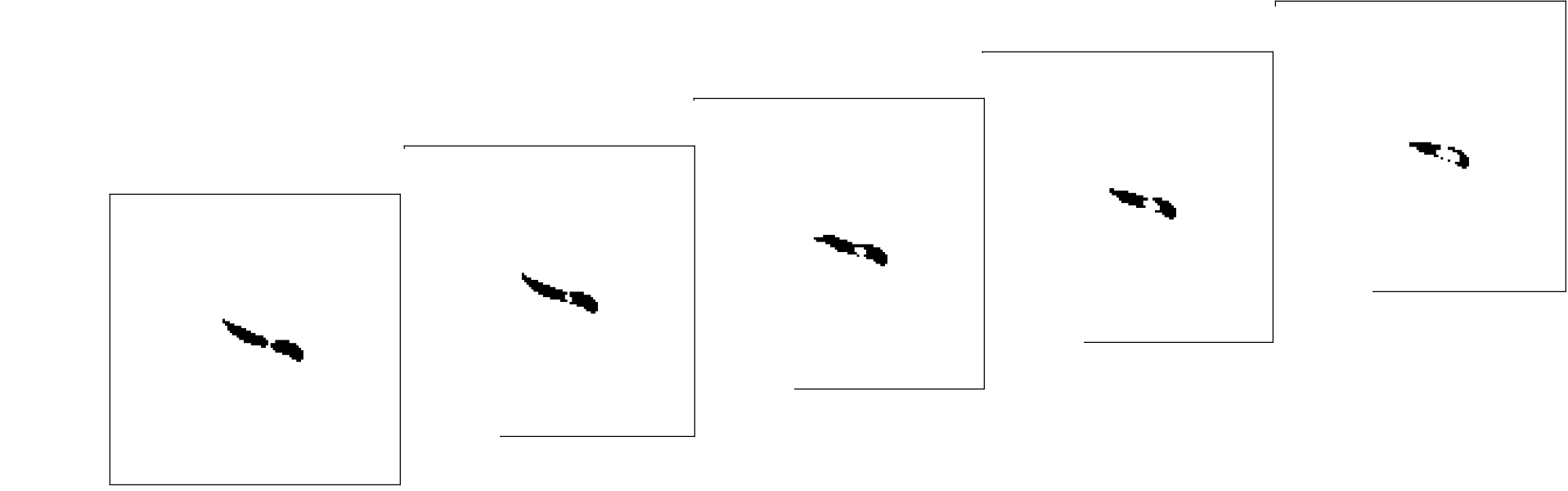

Supplement: S1 HTML — PiVR, Raspberry Pi Virtual Reality. (ZIP) [file pbio.3000712.s019.zip › S1HTLM/_images/Mode3Waiting.png]

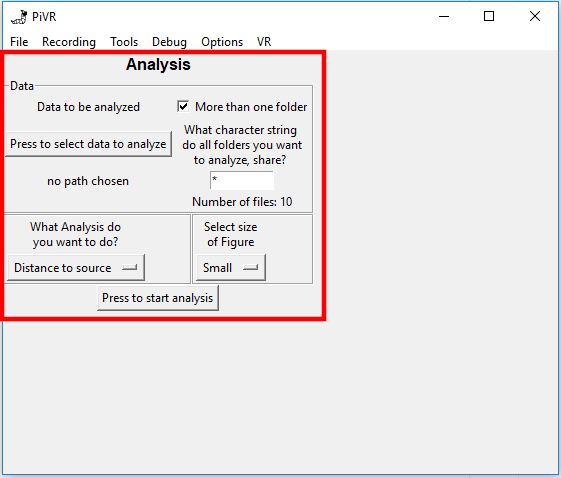

Supplement: S1 HTML — PiVR, Raspberry Pi Virtual Reality. (ZIP) [file pbio.3000712.s019.zip › S1HTLM/_images/2_AnalysisFrame.png]

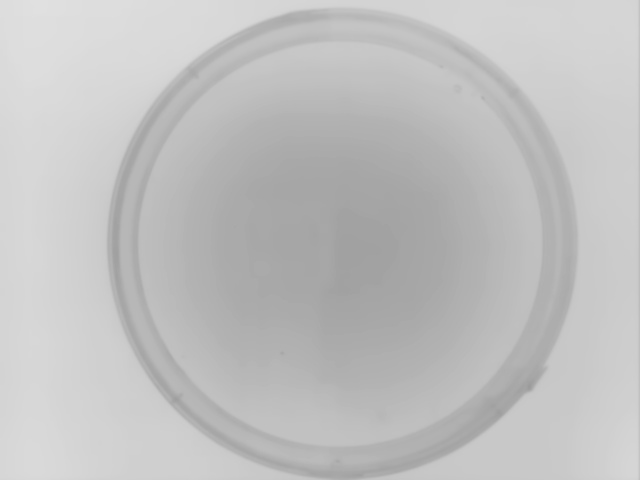

Supplement: S1 HTML — PiVR, Raspberry Pi Virtual Reality. (ZIP) [file pbio.3000712.s019.zip › S1HTLM/_images/Mode2Background.png]

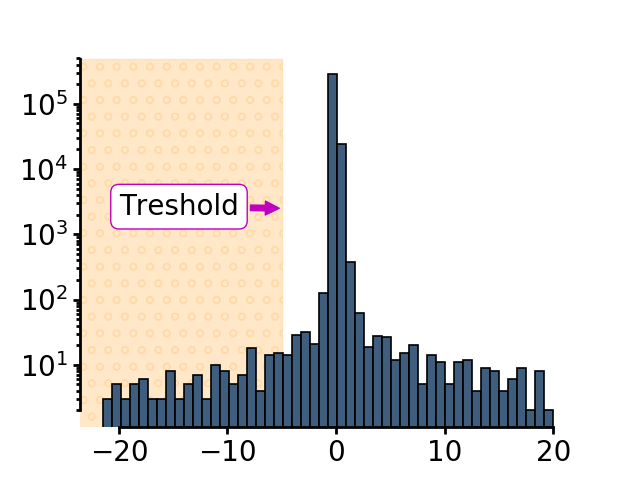

Supplement: S1 HTML — PiVR, Raspberry Pi Virtual Reality. (ZIP) [file pbio.3000712.s019.zip › S1HTLM/_images/HistogramMode1Tresh.png]

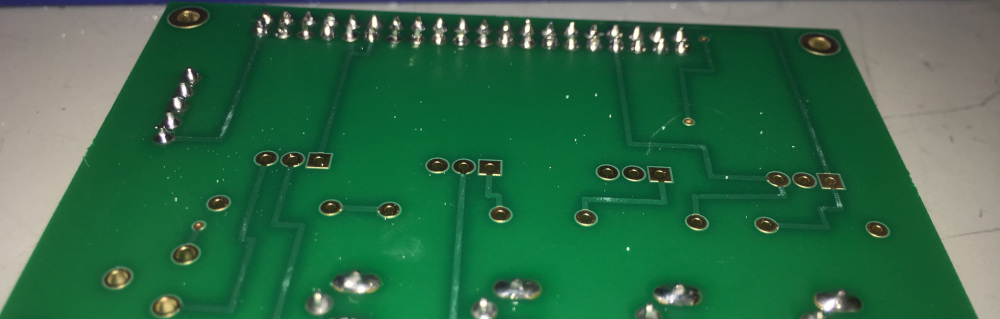

Supplement: S1 HTML — PiVR, Raspberry Pi Virtual Reality. (ZIP) [file pbio.3000712.s019.zip › S1HTLM/_images/S_7.jpg]

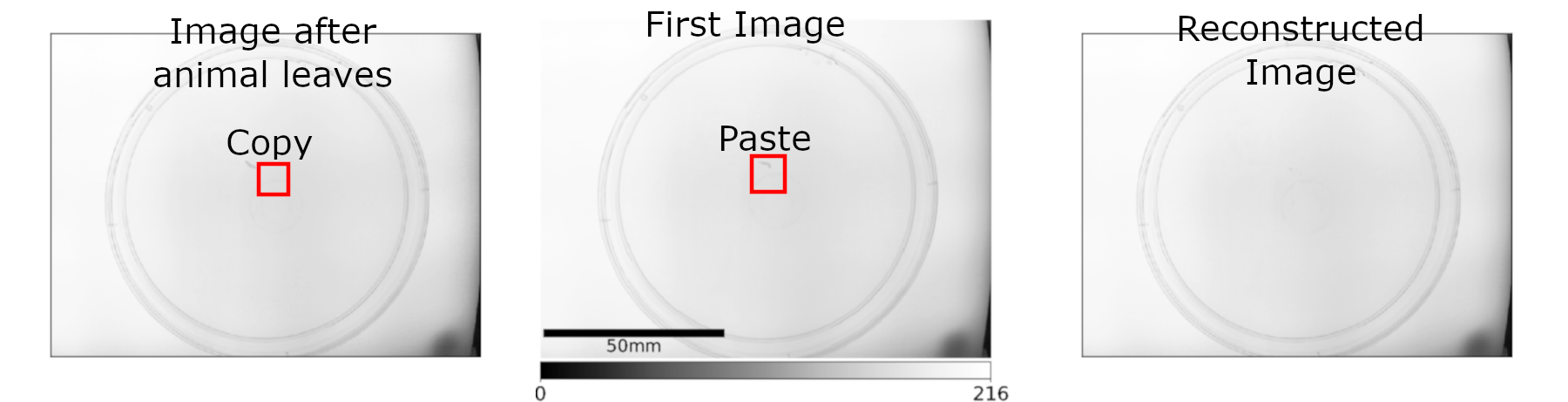

Supplement: S1 HTML — PiVR, Raspberry Pi Virtual Reality. (ZIP) [file pbio.3000712.s019.zip › S1HTLM/_images/Mode3reconstruction.png]

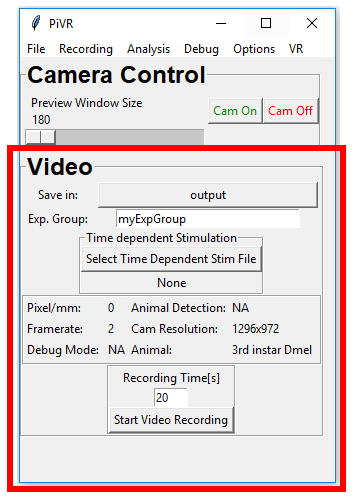

Supplement: S1 HTML — PiVR, Raspberry Pi Virtual Reality. (ZIP) [file pbio.3000712.s019.zip › S1HTLM/_images/ExperimentControlVideo.png]

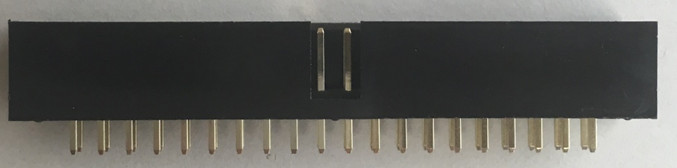

Supplement: S1 HTML — PiVR, Raspberry Pi Virtual Reality. (ZIP) [file pbio.3000712.s019.zip › S1HTLM/_images/2_2_GPIO-Header.jpg]

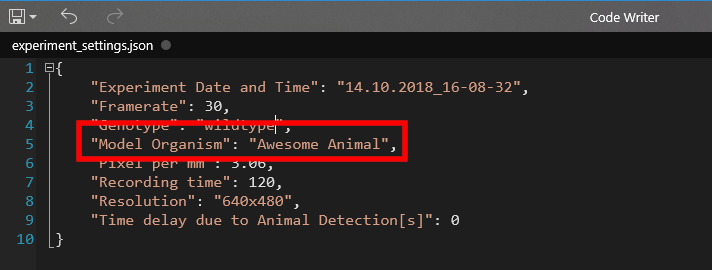

Supplement: S1 HTML — PiVR, Raspberry Pi Virtual Reality. (ZIP) [file pbio.3000712.s019.zip › S1HTLM/_images/10_change_experiment_settings.png]

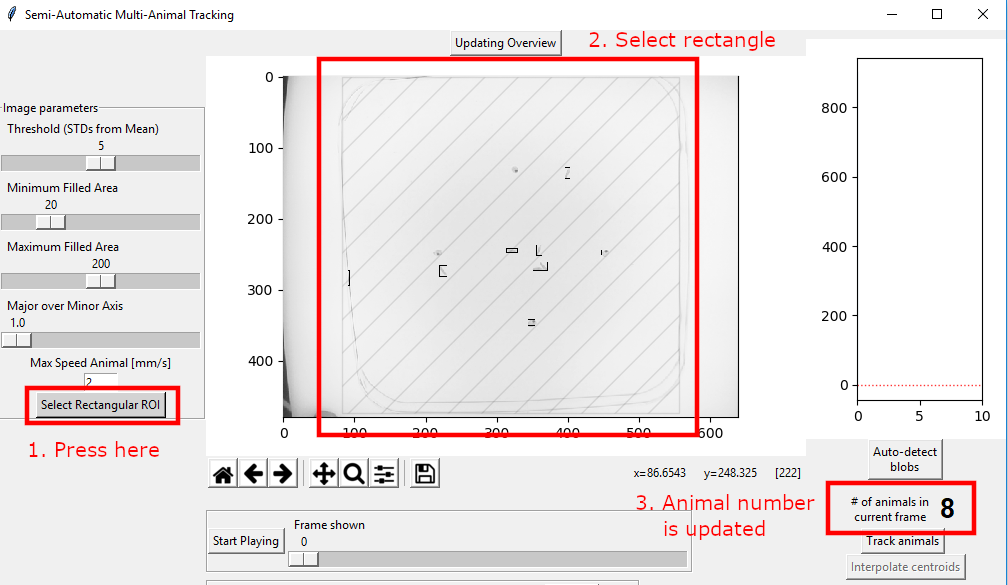

Supplement: S1 HTML — PiVR, Raspberry Pi Virtual Reality. (ZIP) [file pbio.3000712.s019.zip › S1HTLM/_images/6_2_MultiAnimalTrackerSelectRectangle.png]

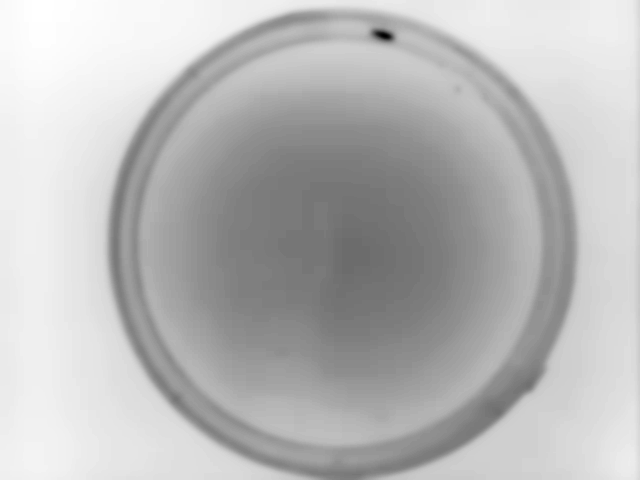

Supplement: S1 HTML — PiVR, Raspberry Pi Virtual Reality. (ZIP) [file pbio.3000712.s019.zip › S1HTLM/_images/Mode1Background.png]

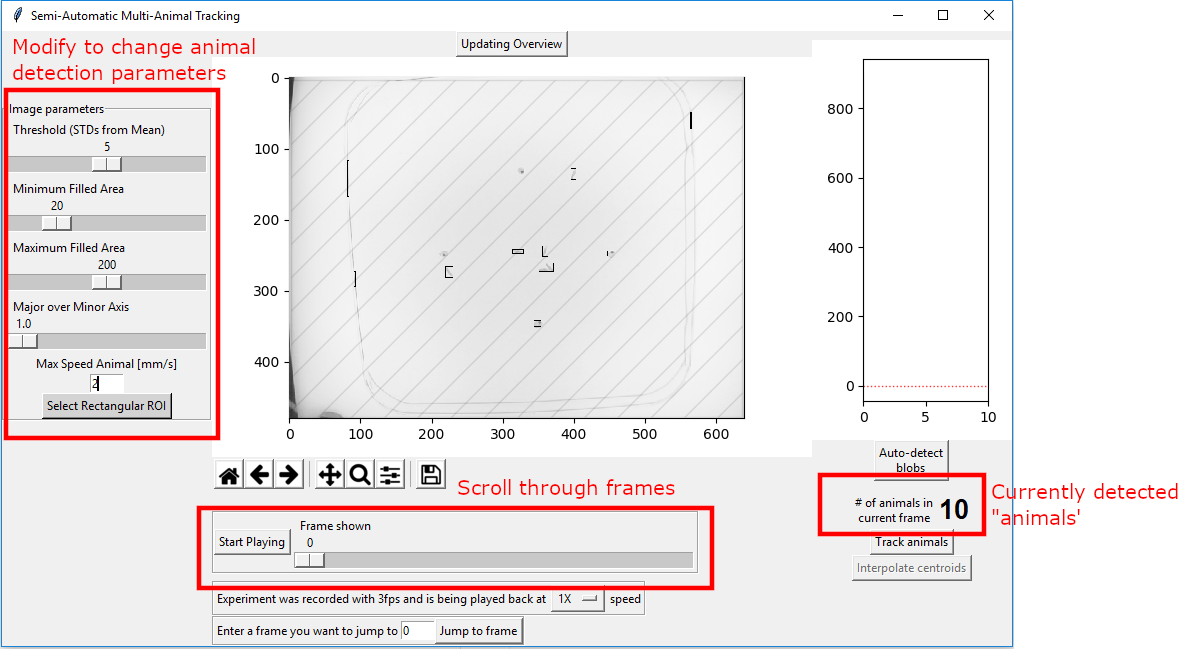

Supplement: S1 HTML — PiVR, Raspberry Pi Virtual Reality. (ZIP) [file pbio.3000712.s019.zip › S1HTLM/_images/6_1_MultiAnimalTrackerWindow.png]

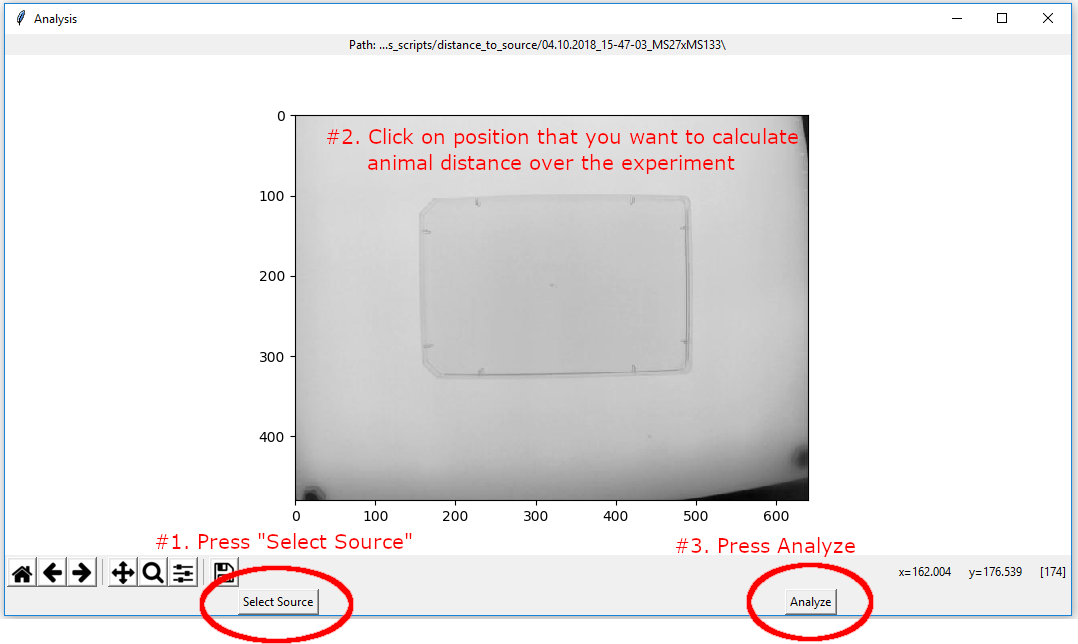

Supplement: S1 HTML — PiVR, Raspberry Pi Virtual Reality. (ZIP) [file pbio.3000712.s019.zip › S1HTLM/_images/3_DistanceToSource.png]

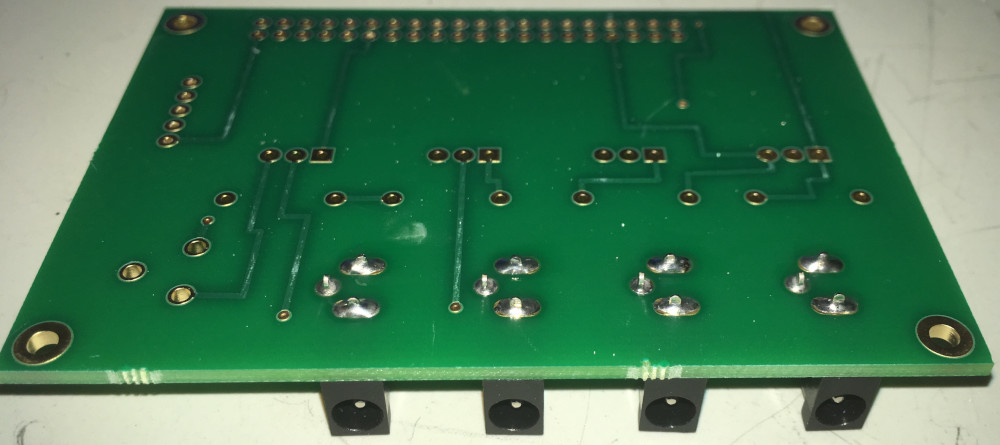

Supplement: S1 HTML — PiVR, Raspberry Pi Virtual Reality. (ZIP) [file pbio.3000712.s019.zip › S1HTLM/_images/S_3.jpg]

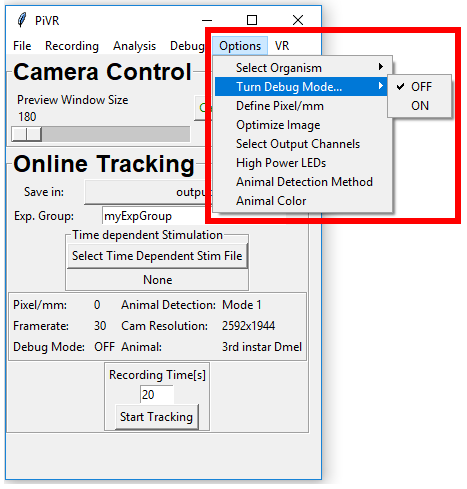

Supplement: S1 HTML — PiVR, Raspberry Pi Virtual Reality. (ZIP) [file pbio.3000712.s019.zip › S1HTLM/_images/OptionsDebugMode.png]

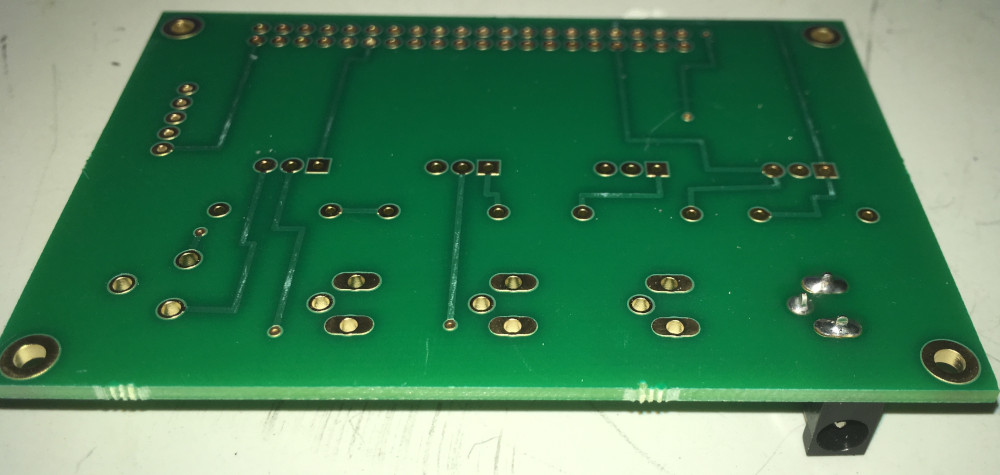

Supplement: S1 HTML — PiVR, Raspberry Pi Virtual Reality. (ZIP) [file pbio.3000712.s019.zip › S1HTLM/_images/S_2.jpg]

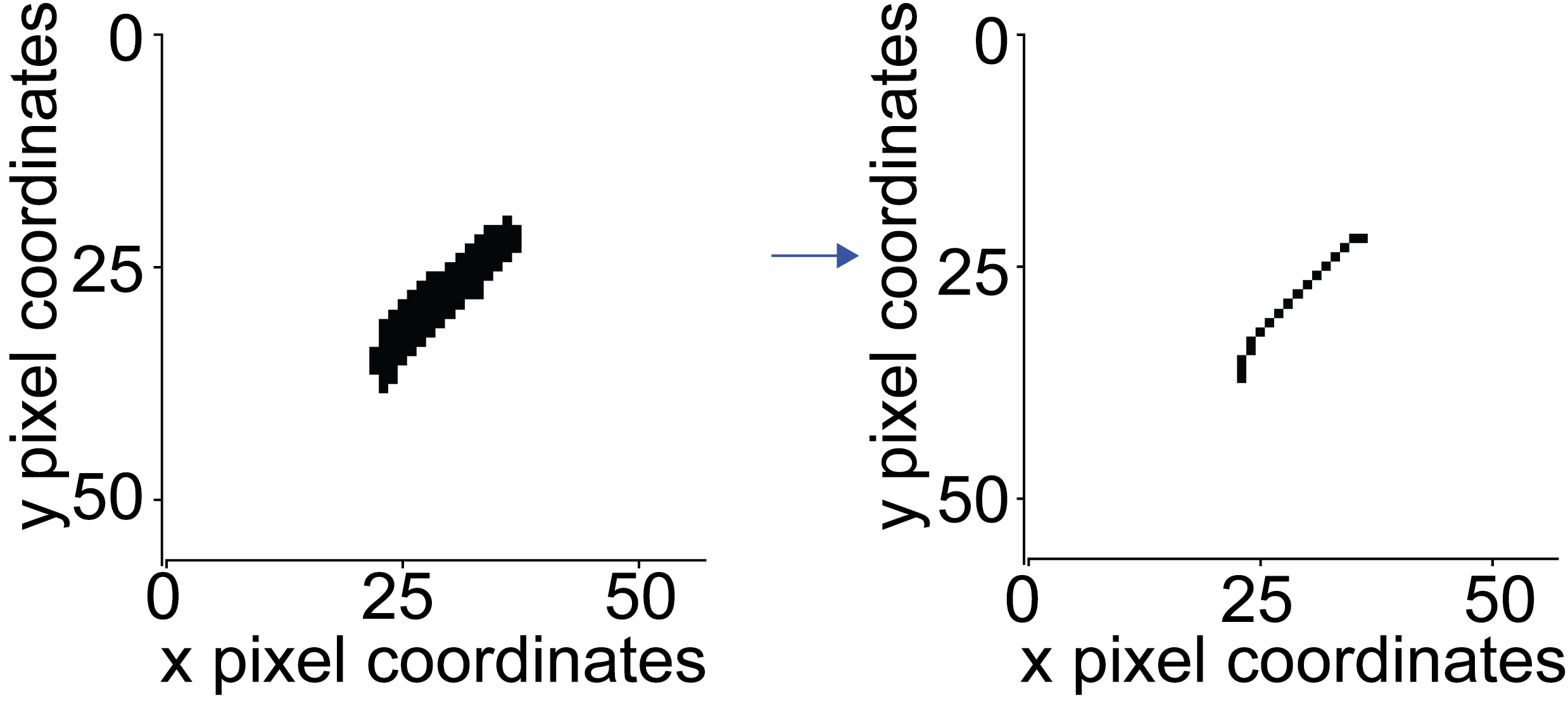

Supplement: S1 HTML — PiVR, Raspberry Pi Virtual Reality. (ZIP) [file pbio.3000712.s019.zip › S1HTLM/_images/FigS5_Skeletonize.png]

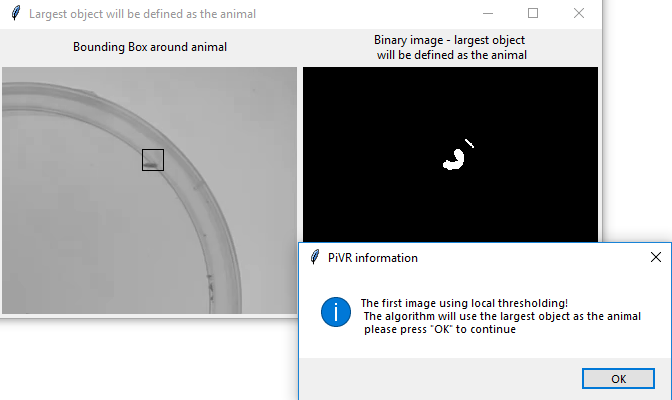

Supplement: S1 HTML — PiVR, Raspberry Pi Virtual Reality. (ZIP) [file pbio.3000712.s019.zip › S1HTLM/_images/2_debug_detection2.png]

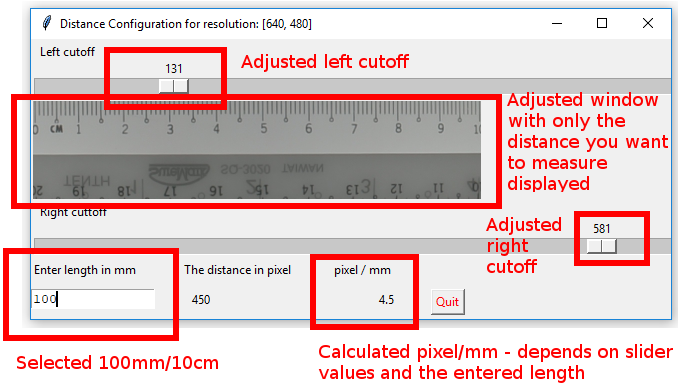

Supplement: S1 HTML — PiVR, Raspberry Pi Virtual Reality. (ZIP) [file pbio.3000712.s019.zip › S1HTLM/_images/DistanceConfigurationAdjusted.png]

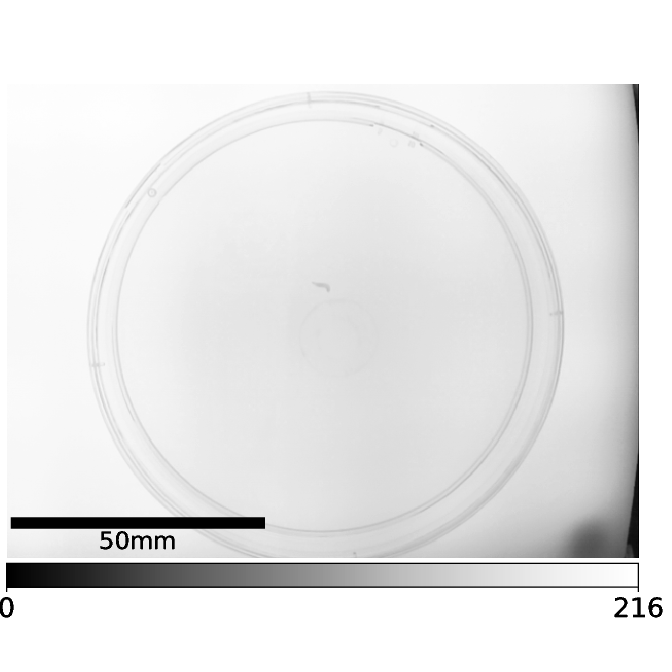

Supplement: S1 HTML — PiVR, Raspberry Pi Virtual Reality. (ZIP) [file pbio.3000712.s019.zip › S1HTLM/_images/Mode3FirstImage.png]

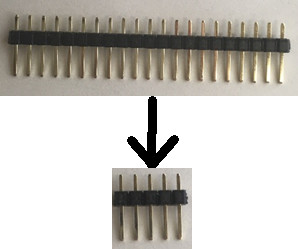

Supplement: S1 HTML — PiVR, Raspberry Pi Virtual Reality. (ZIP) [file pbio.3000712.s019.zip › S1HTLM/_images/2_6_Headers.jpg]

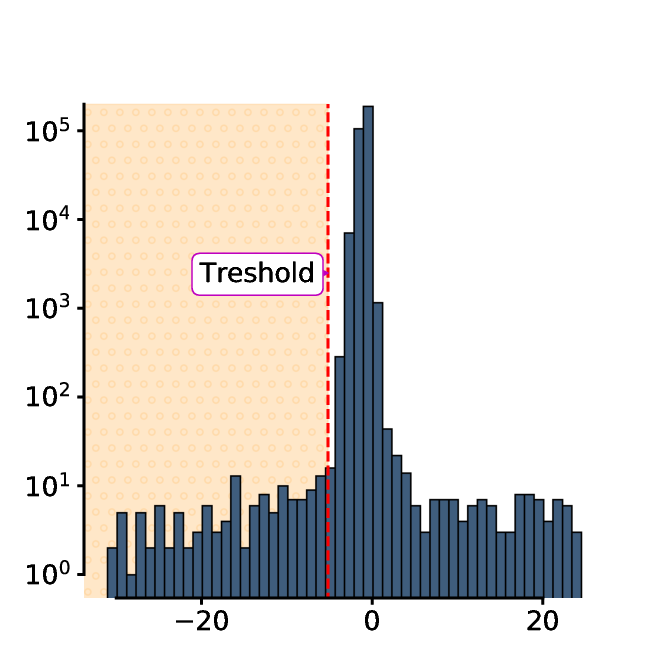

Supplement: S1 HTML — PiVR, Raspberry Pi Virtual Reality. (ZIP) [file pbio.3000712.s019.zip › S1HTLM/_images/Mode3HistogramMode1Tresh.png]

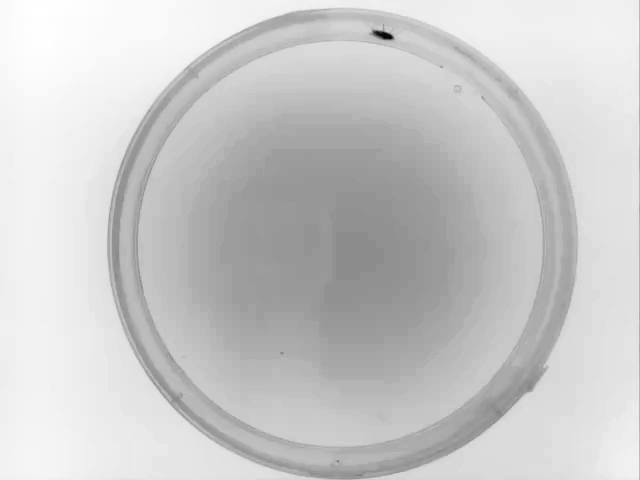

Supplement: S1 HTML — PiVR, Raspberry Pi Virtual Reality. (ZIP) [file pbio.3000712.s019.zip › S1HTLM/_images/FirstImage.png]

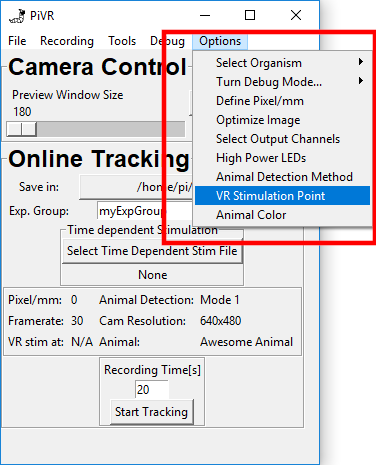

Supplement: S1 HTML — PiVR, Raspberry Pi Virtual Reality. (ZIP) [file pbio.3000712.s019.zip › S1HTLM/_images/OptionsVRStimulationPoint.png]

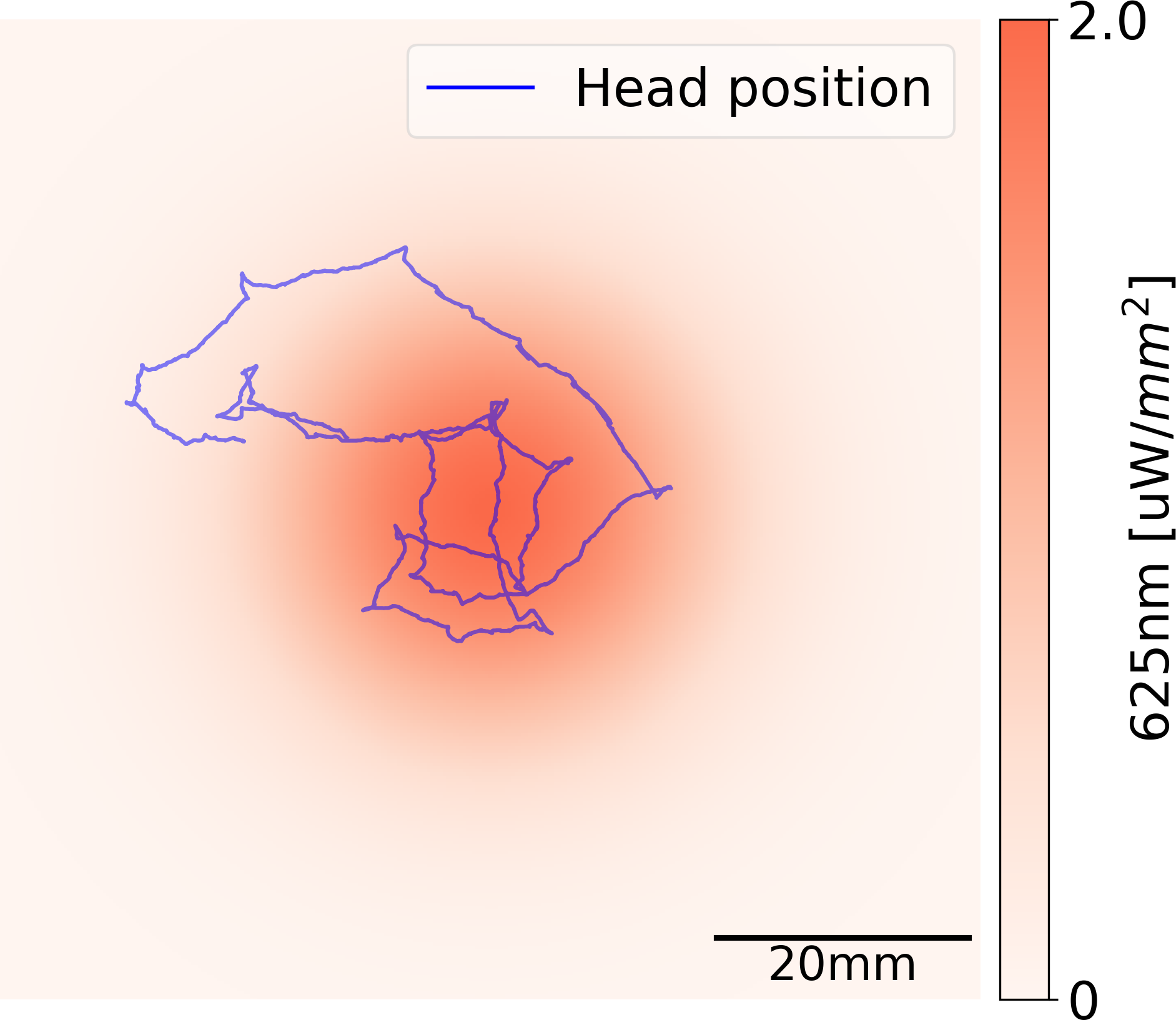

Supplement: S1 HTML — PiVR, Raspberry Pi Virtual Reality. (ZIP) [file pbio.3000712.s019.zip › S1HTLM/_images/larval_trajectory.png]

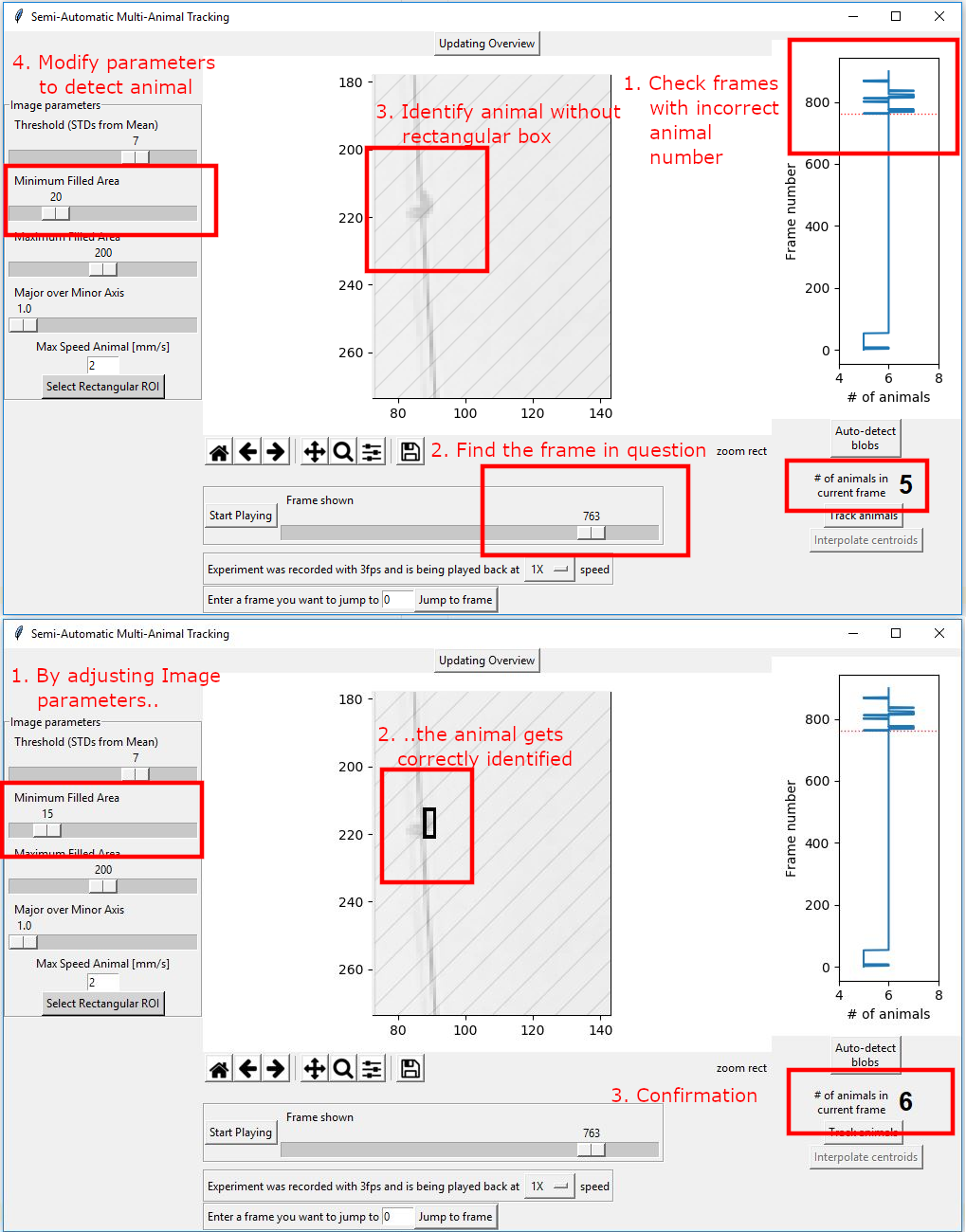

Supplement: S1 HTML — PiVR, Raspberry Pi Virtual Reality. (ZIP) [file pbio.3000712.s019.zip › S1HTLM/_images/6_6_MultiAnimalTrackerSelectSearchProblematicFrames1.png]

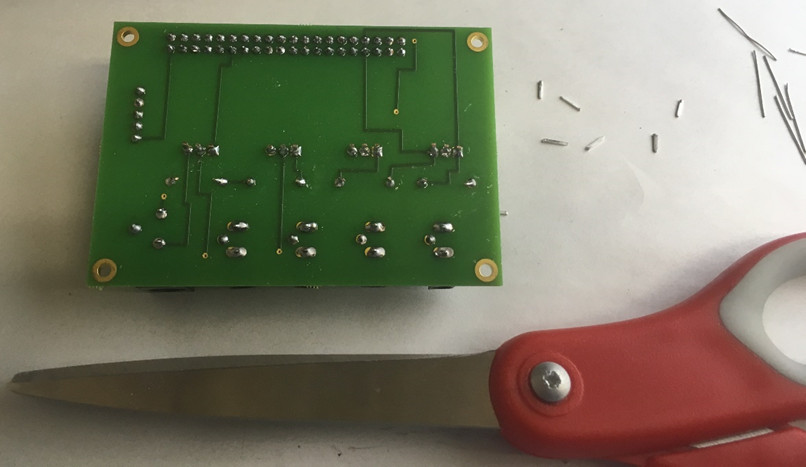

Supplement: S1 HTML — PiVR, Raspberry Pi Virtual Reality. (ZIP) [file pbio.3000712.s019.zip › S1HTLM/_images/4_PCB_removed_wire.jpg]

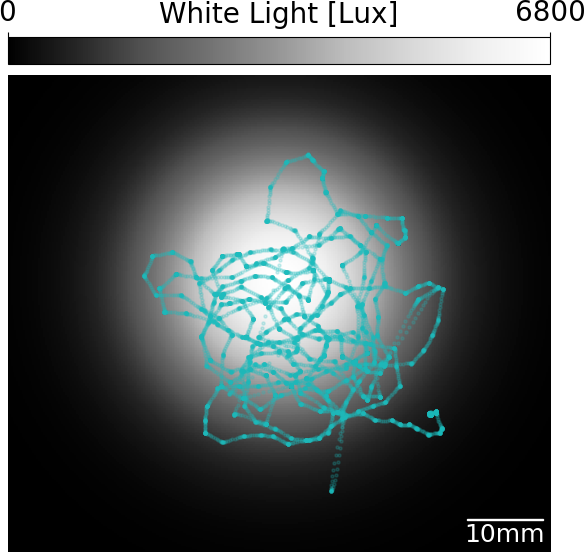

Supplement: S1 HTML — PiVR, Raspberry Pi Virtual Reality. (ZIP) [file pbio.3000712.s019.zip › S1HTLM/_images/fish_trajectory.png]

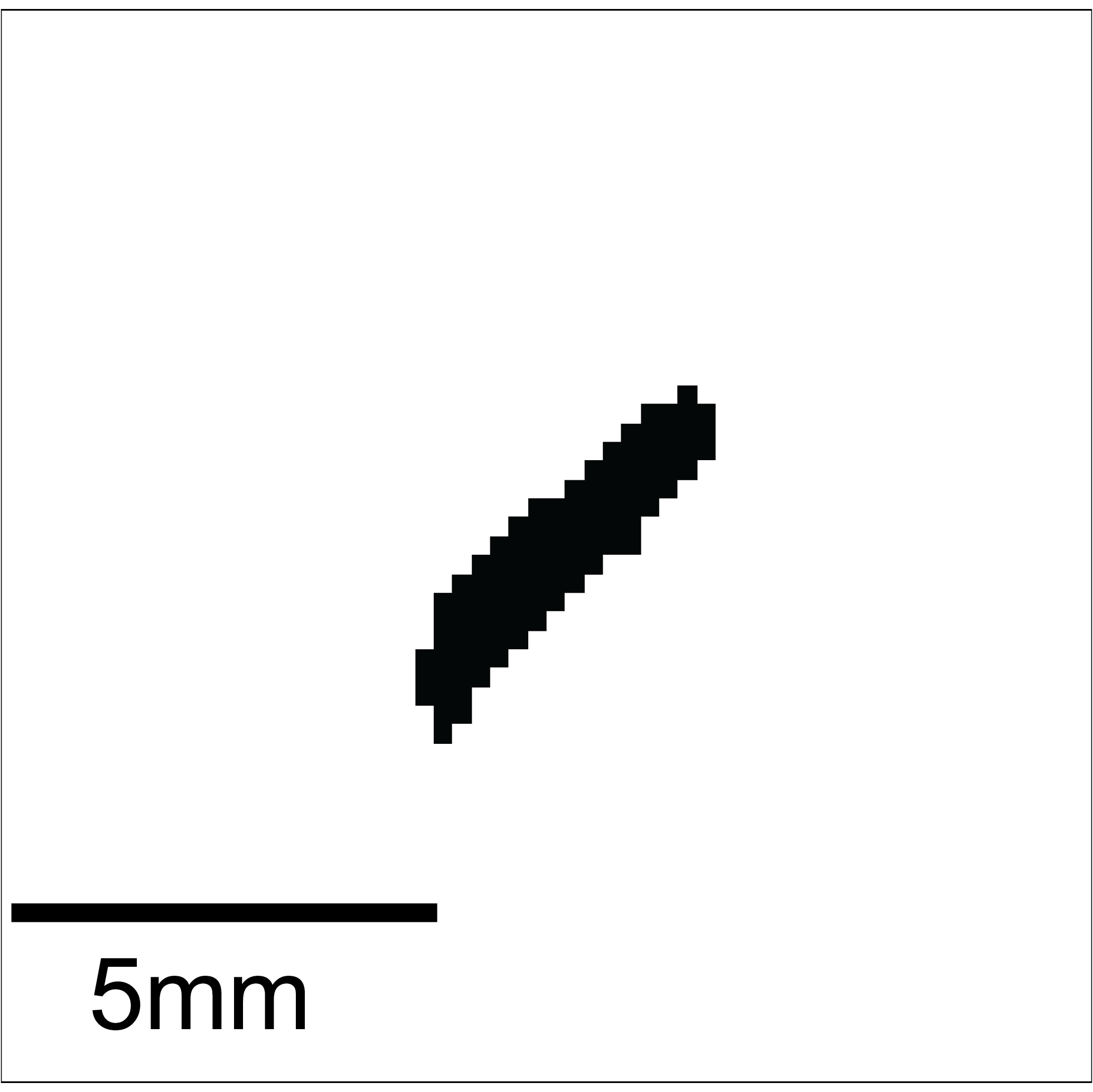

Supplement: S1 HTML — PiVR, Raspberry Pi Virtual Reality. (ZIP) [file pbio.3000712.s019.zip › S1HTLM/_images/FigS4_Binary.png]

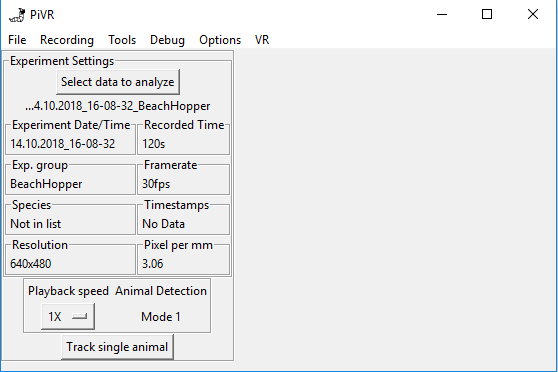

Supplement: S1 HTML — PiVR, Raspberry Pi Virtual Reality. (ZIP) [file pbio.3000712.s019.zip › S1HTLM/_images/12_read_metadata.png]

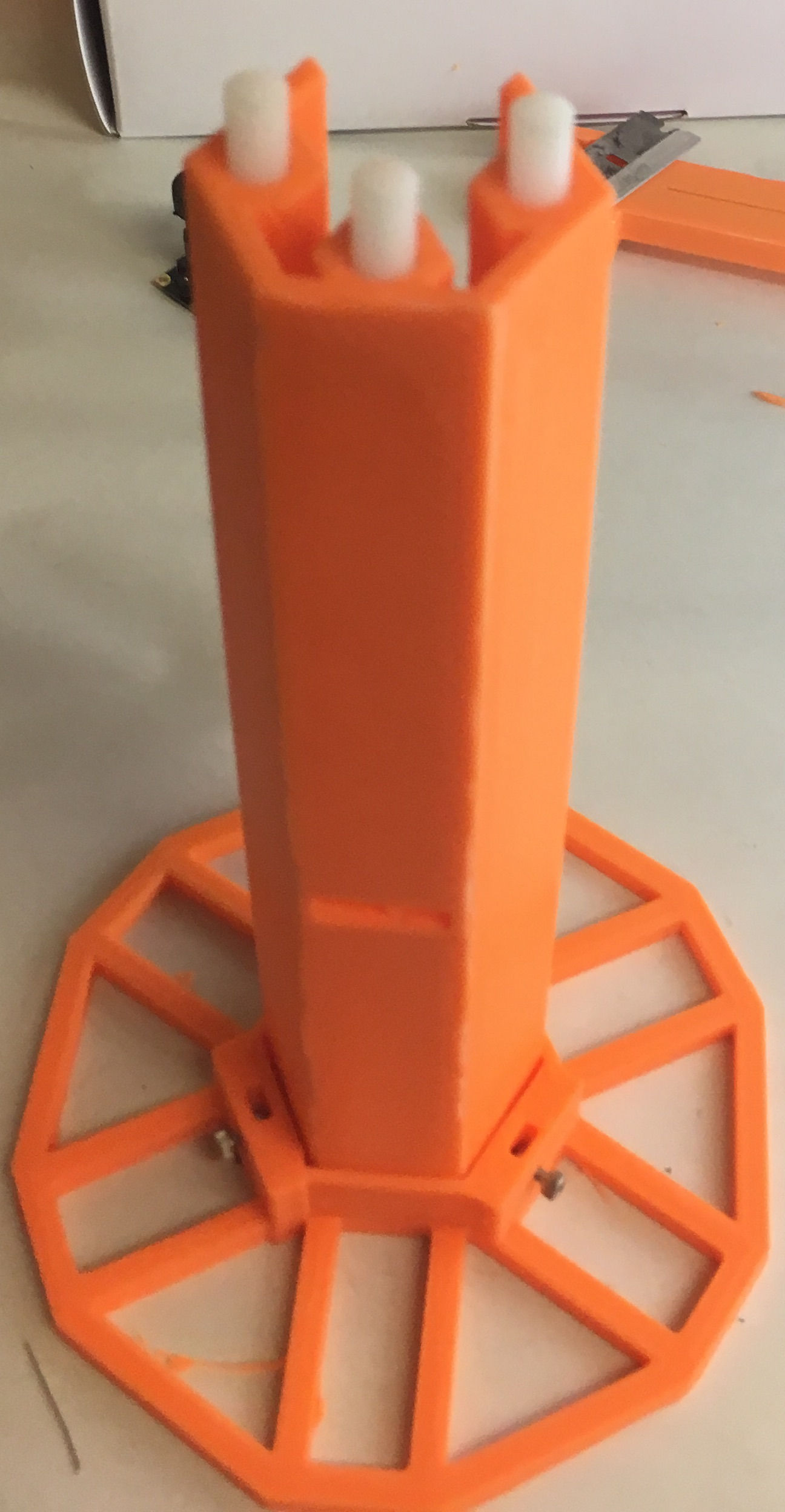

Supplement: S1 HTML — PiVR, Raspberry Pi Virtual Reality. (ZIP) [file pbio.3000712.s019.zip › S1HTLM/_images/15_TowerBottom_Dowel.jpg]

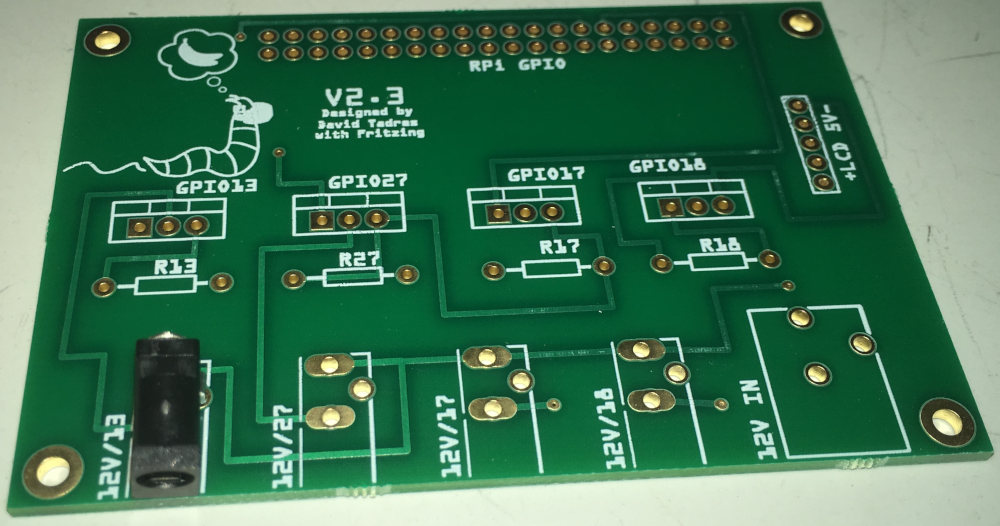

Supplement: S1 HTML — PiVR, Raspberry Pi Virtual Reality. (ZIP) [file pbio.3000712.s019.zip › S1HTLM/_images/S_1.jpg]

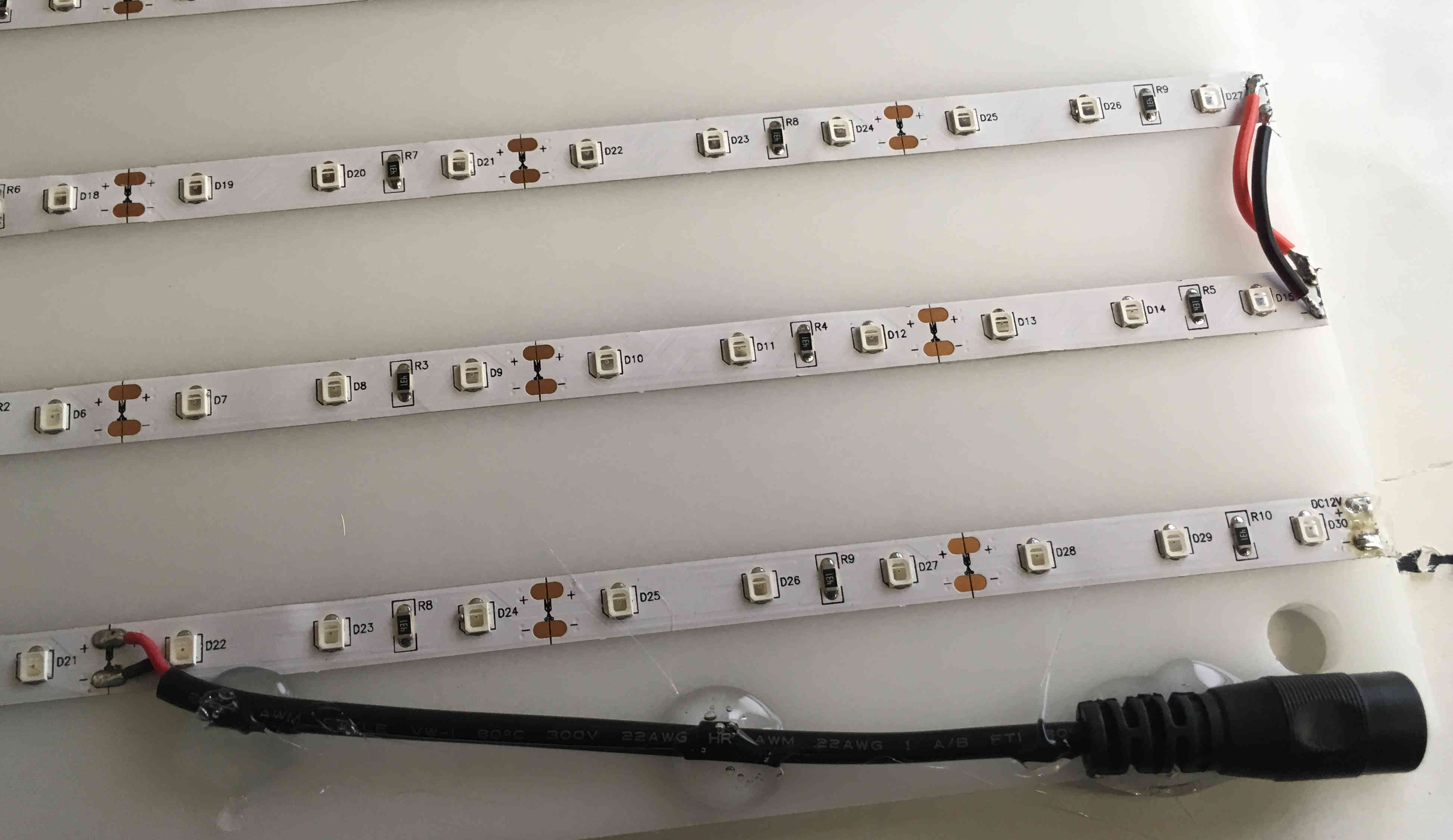

Supplement: S1 HTML — PiVR, Raspberry Pi Virtual Reality. (ZIP) [file pbio.3000712.s019.zip › S1HTLM/_images/24_arena_barrel_jack_attached.jpg]

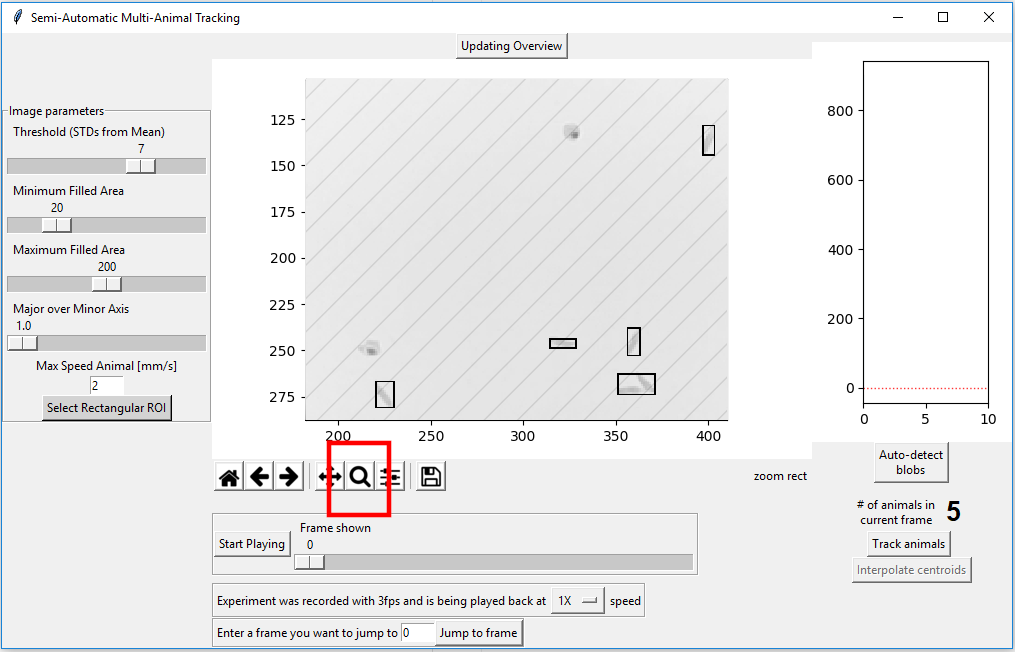

Supplement: S1 HTML — PiVR, Raspberry Pi Virtual Reality. (ZIP) [file pbio.3000712.s019.zip › S1HTLM/_images/6_4_MultiAnimalTrackerSelectZoom.png]

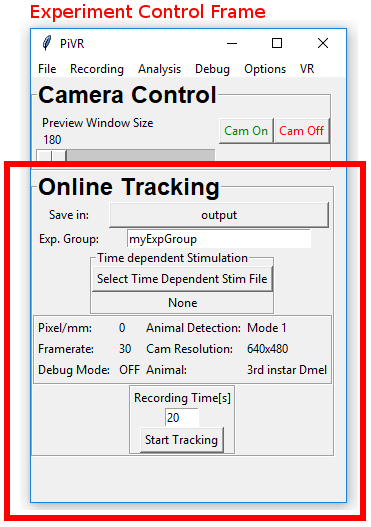

Supplement: S1 HTML — PiVR, Raspberry Pi Virtual Reality. (ZIP) [file pbio.3000712.s019.zip › S1HTLM/_images/ExperimentControlFrameTracking.png]

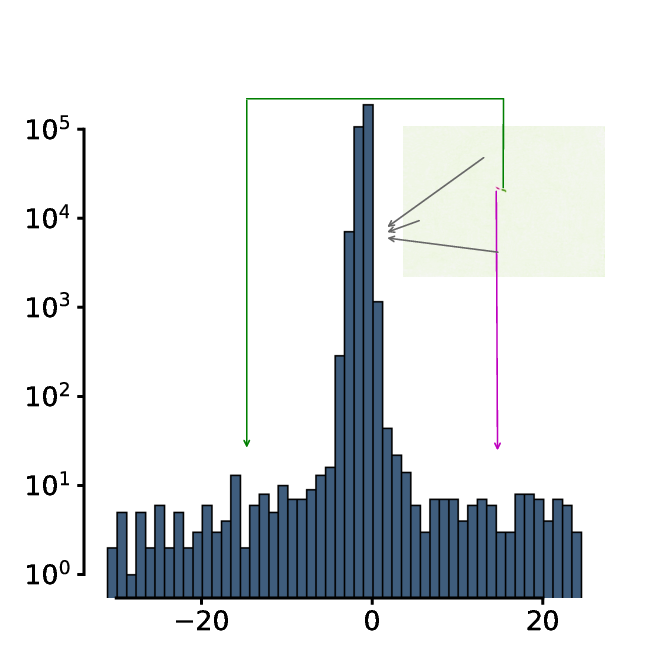

Supplement: S1 HTML — PiVR, Raspberry Pi Virtual Reality. (ZIP) [file pbio.3000712.s019.zip › S1HTLM/_images/Mode3HistogramSubtractedImage.png]

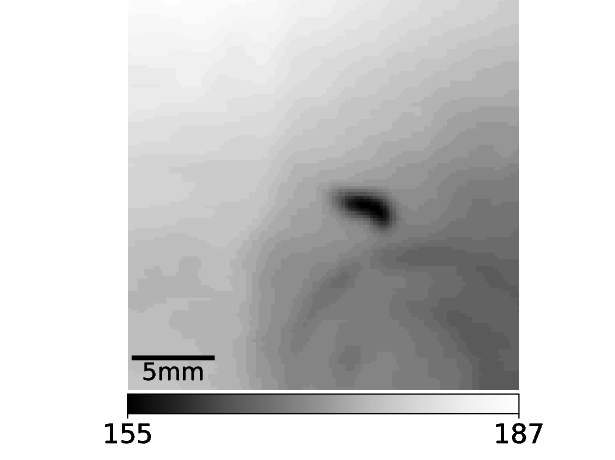

Supplement: S1 HTML — PiVR, Raspberry Pi Virtual Reality. (ZIP) [file pbio.3000712.s019.zip › S1HTLM/_images/Mode3ROI_Raw.png]

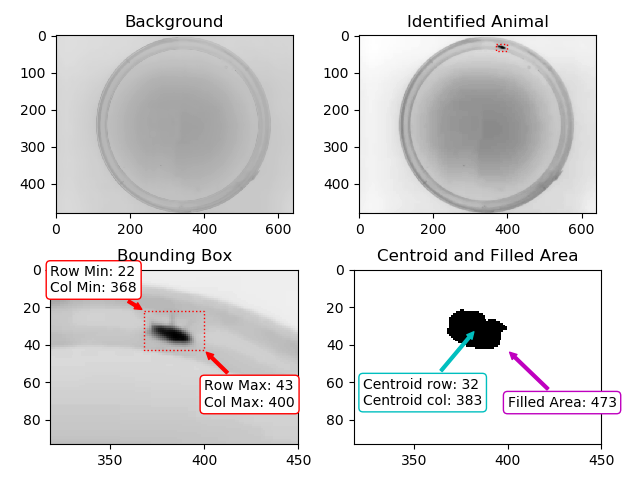

Supplement: S1 HTML — PiVR, Raspberry Pi Virtual Reality. (ZIP) [file pbio.3000712.s019.zip › S1HTLM/_images/Mode2Output.png]

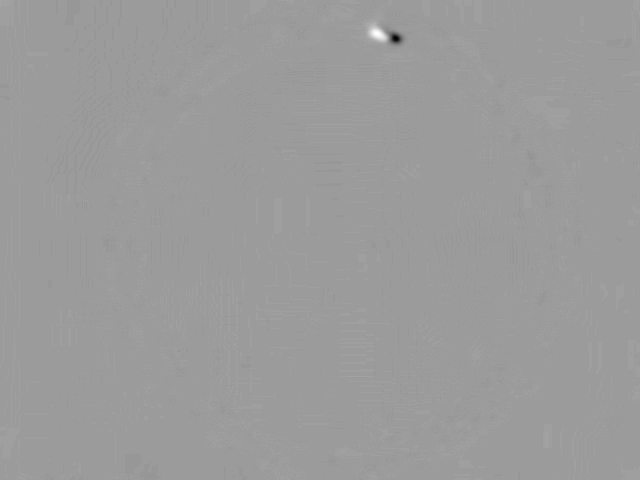

Supplement: S1 HTML — PiVR, Raspberry Pi Virtual Reality. (ZIP) [file pbio.3000712.s019.zip › S1HTLM/_images/SecondSubtractedImage.png]

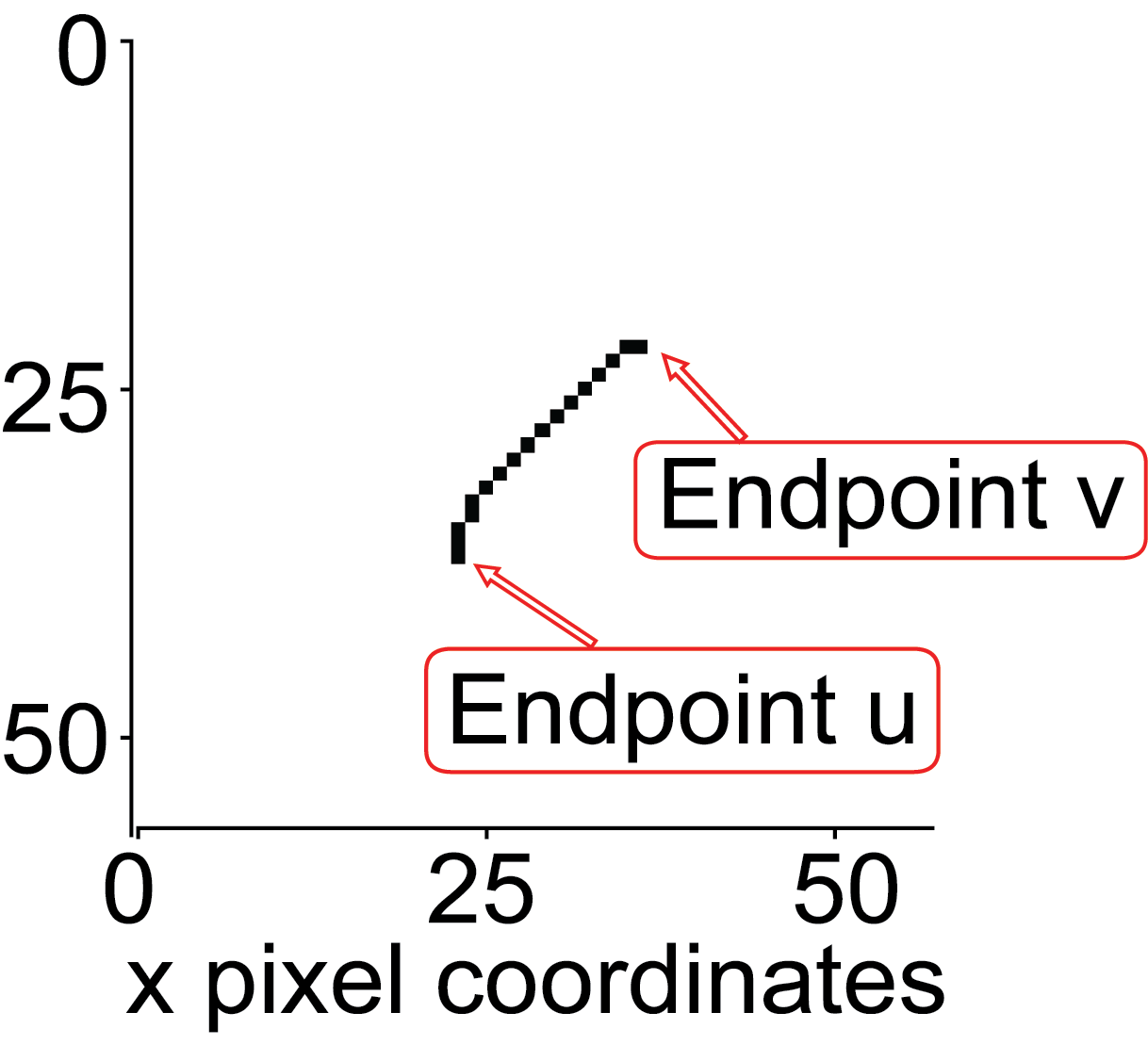

Supplement: S1 HTML — PiVR, Raspberry Pi Virtual Reality. (ZIP) [file pbio.3000712.s019.zip › S1HTLM/_images/FigS5_Endpoints.png]

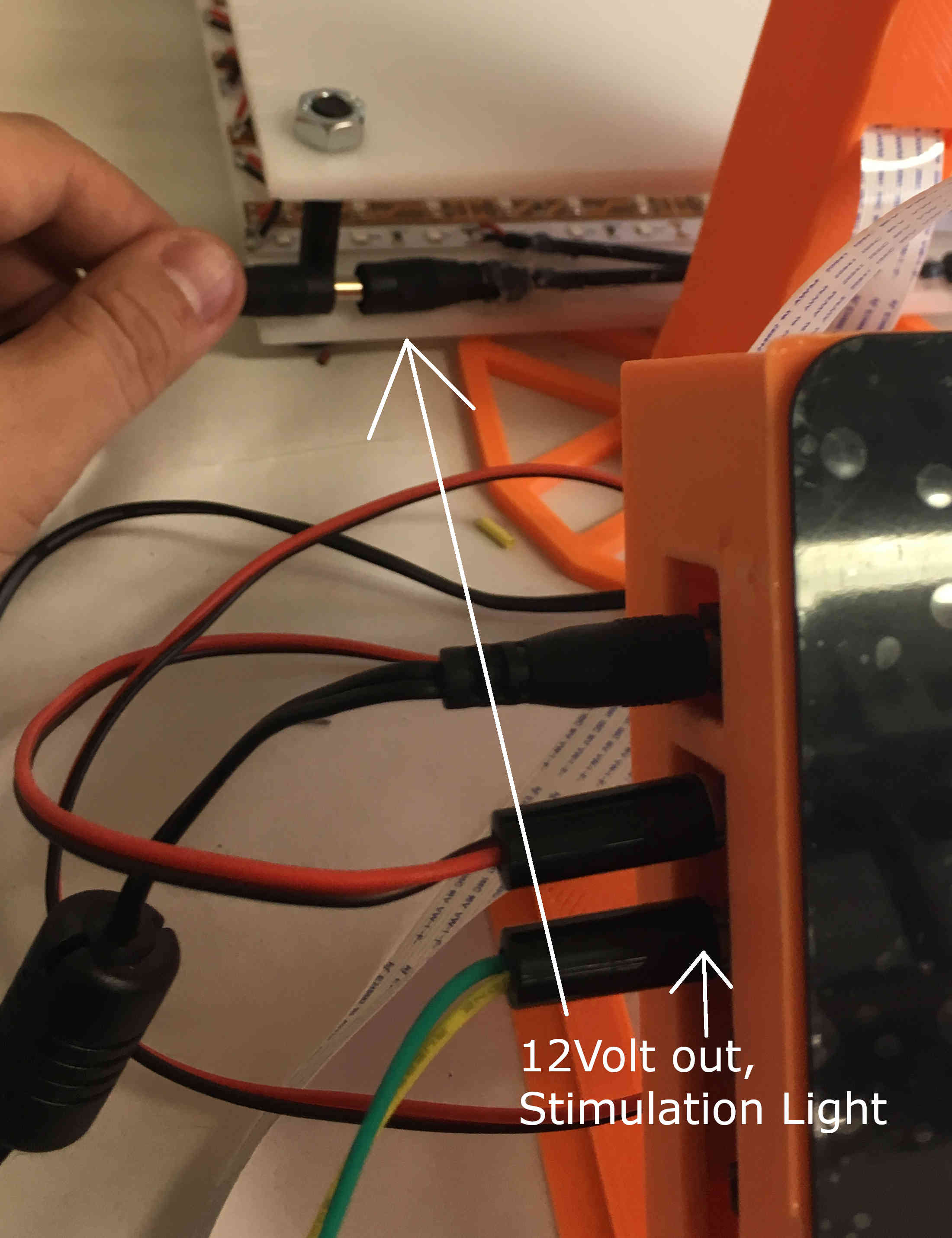

Supplement: S1 HTML — PiVR, Raspberry Pi Virtual Reality. (ZIP) [file pbio.3000712.s019.zip › S1HTLM/_images/31_connecting_redlight_cable.jpg]

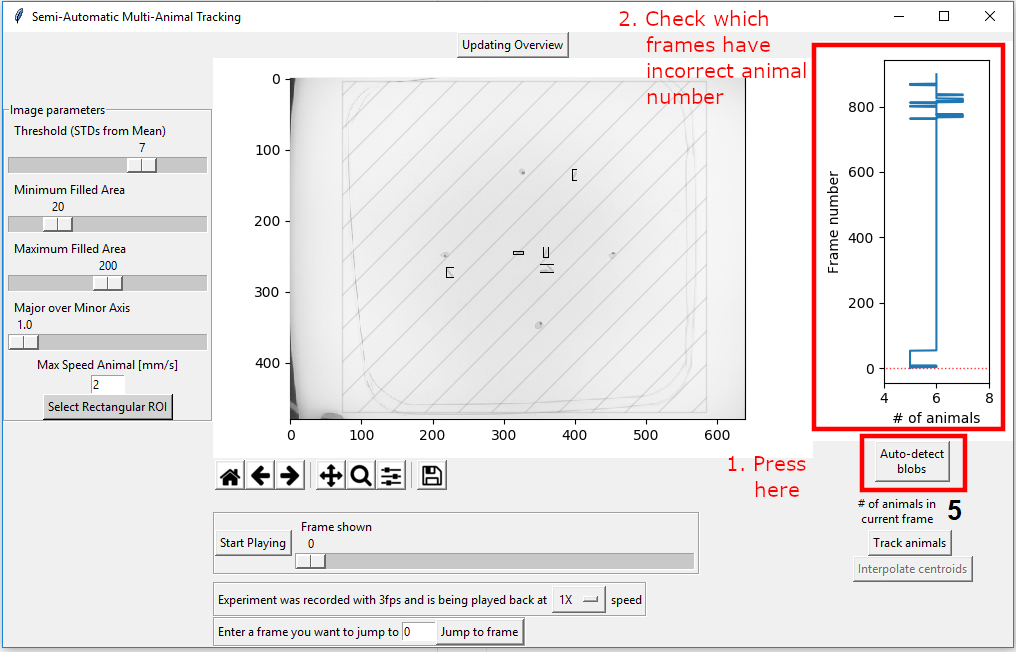

Supplement: S1 HTML — PiVR, Raspberry Pi Virtual Reality. (ZIP) [file pbio.3000712.s019.zip › S1HTLM/_images/6_5_MultiAnimalTrackerSelectDetectBlobs.png]

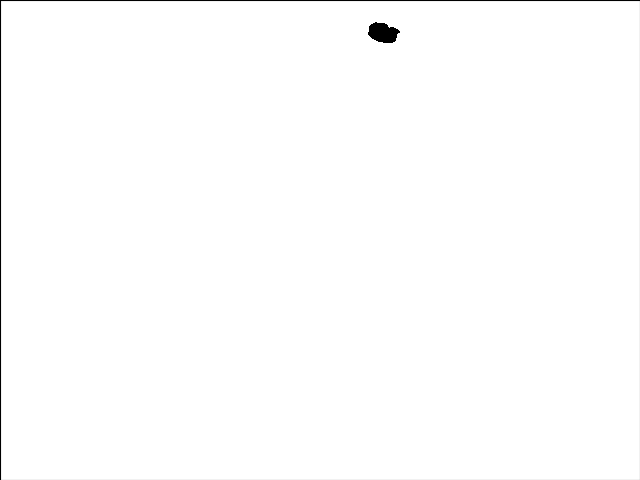

Supplement: S1 HTML — PiVR, Raspberry Pi Virtual Reality. (ZIP) [file pbio.3000712.s019.zip › S1HTLM/_images/Mode2Binary.png]

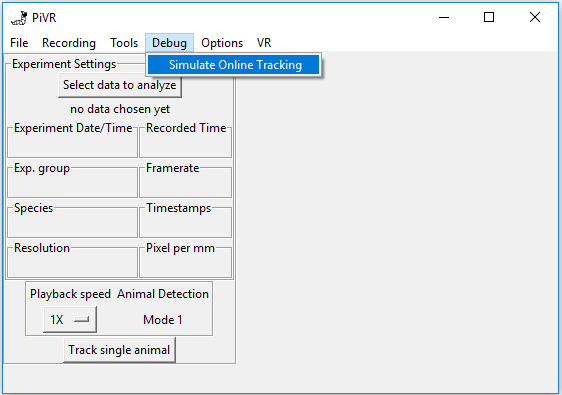

Supplement: S1 HTML — PiVR, Raspberry Pi Virtual Reality. (ZIP) [file pbio.3000712.s019.zip › S1HTLM/_images/11_simulate_online.png]

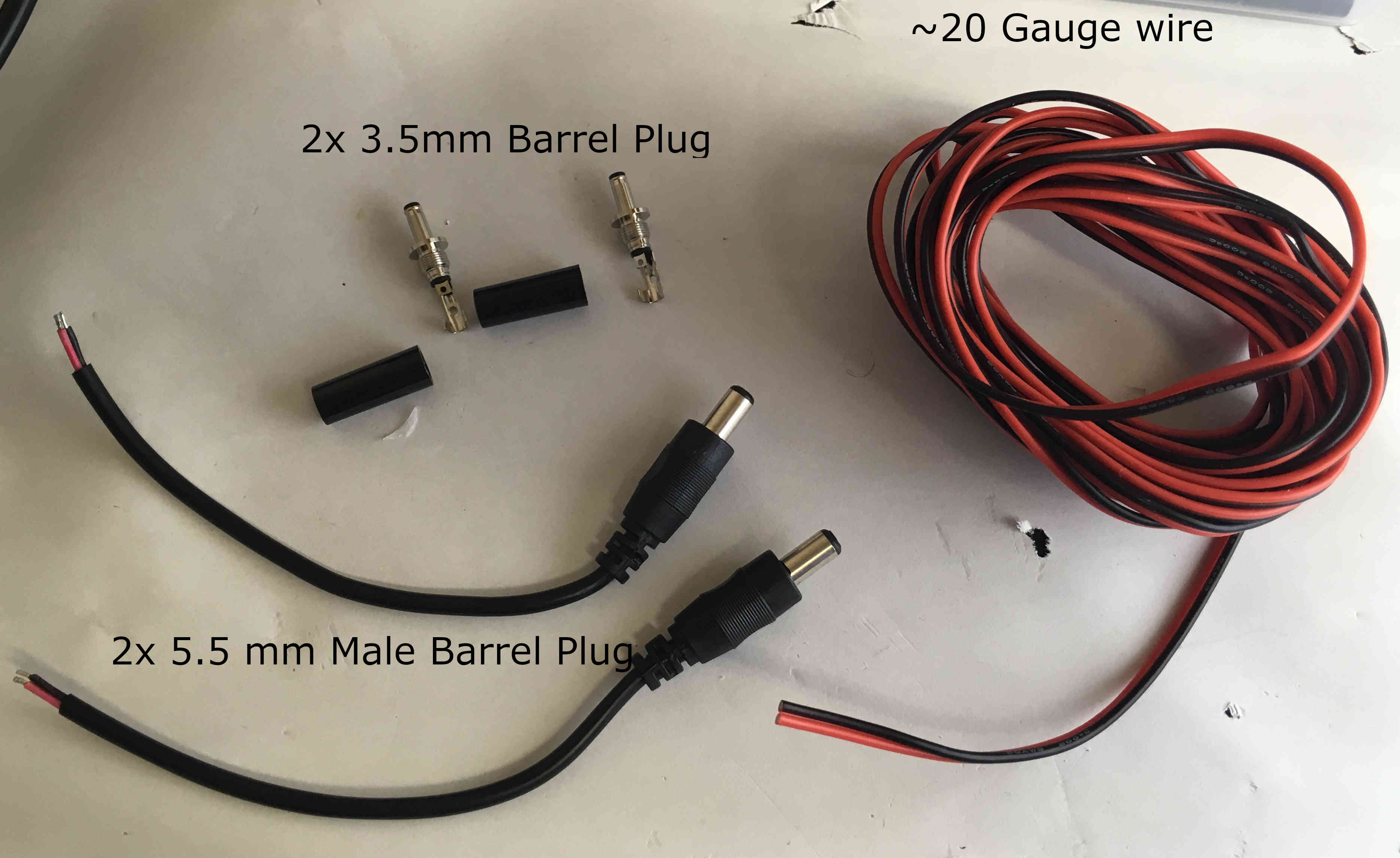

Supplement: S1 HTML — PiVR, Raspberry Pi Virtual Reality. (ZIP) [file pbio.3000712.s019.zip › S1HTLM/_images/27_cables_overview.jpg]

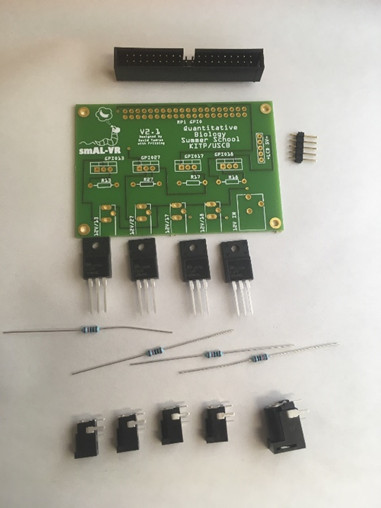

Supplement: S1 HTML — PiVR, Raspberry Pi Virtual Reality. (ZIP) [file pbio.3000712.s019.zip › S1HTLM/_images/2_7_PCB_components.jpg]

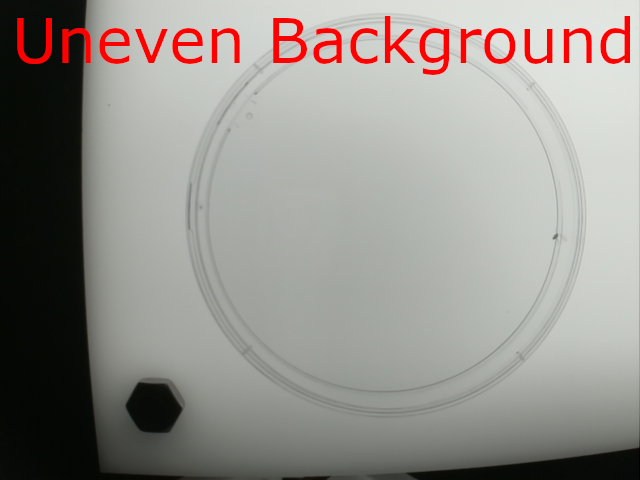

Supplement: S1 HTML — PiVR, Raspberry Pi Virtual Reality. (ZIP) [file pbio.3000712.s019.zip › S1HTLM/_images/ExampleUnevenBackground.png]

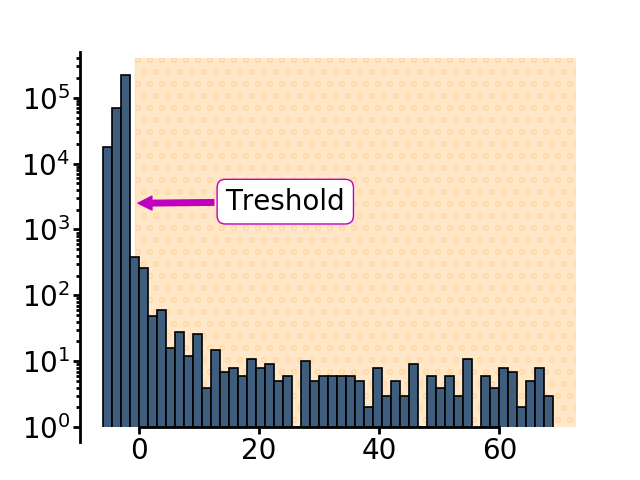

Supplement: S1 HTML — PiVR, Raspberry Pi Virtual Reality. (ZIP) [file pbio.3000712.s019.zip › S1HTLM/_images/Mode2HistogramThresh.png]

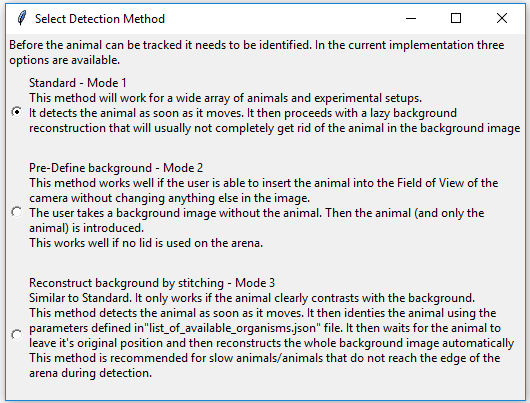

Supplement: S1 HTML — PiVR, Raspberry Pi Virtual Reality. (ZIP) [file pbio.3000712.s019.zip › S1HTLM/_images/SelectAnimalDetection.png]

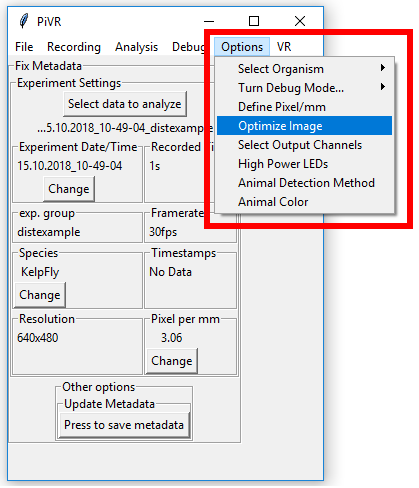

Supplement: S1 HTML — PiVR, Raspberry Pi Virtual Reality. (ZIP) [file pbio.3000712.s019.zip › S1HTLM/_images/OptionsOptimizeImage.png]

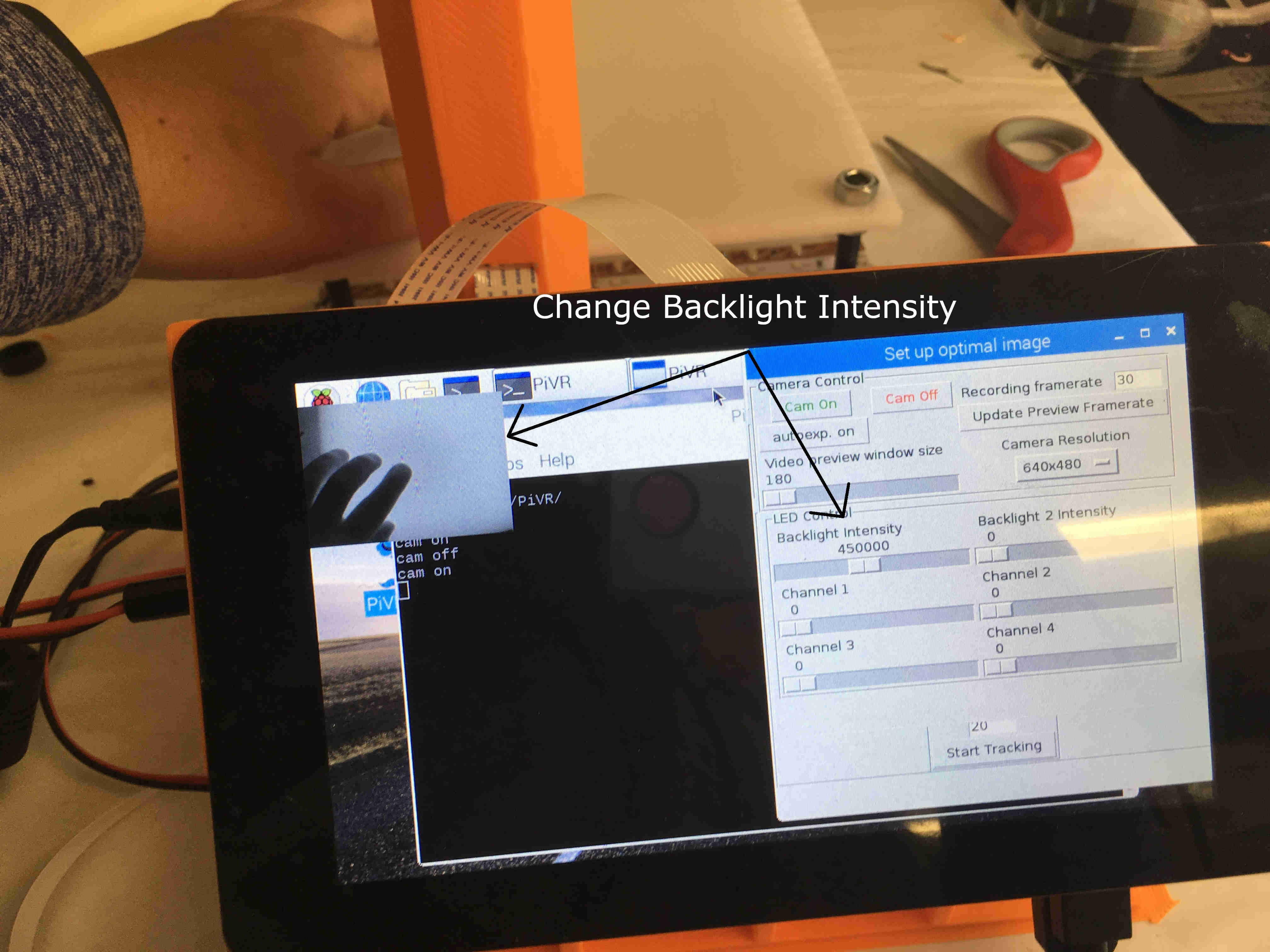

Supplement: S1 HTML — PiVR, Raspberry Pi Virtual Reality. (ZIP) [file pbio.3000712.s019.zip › S1HTLM/_images/30_1_testing_IR.jpg]

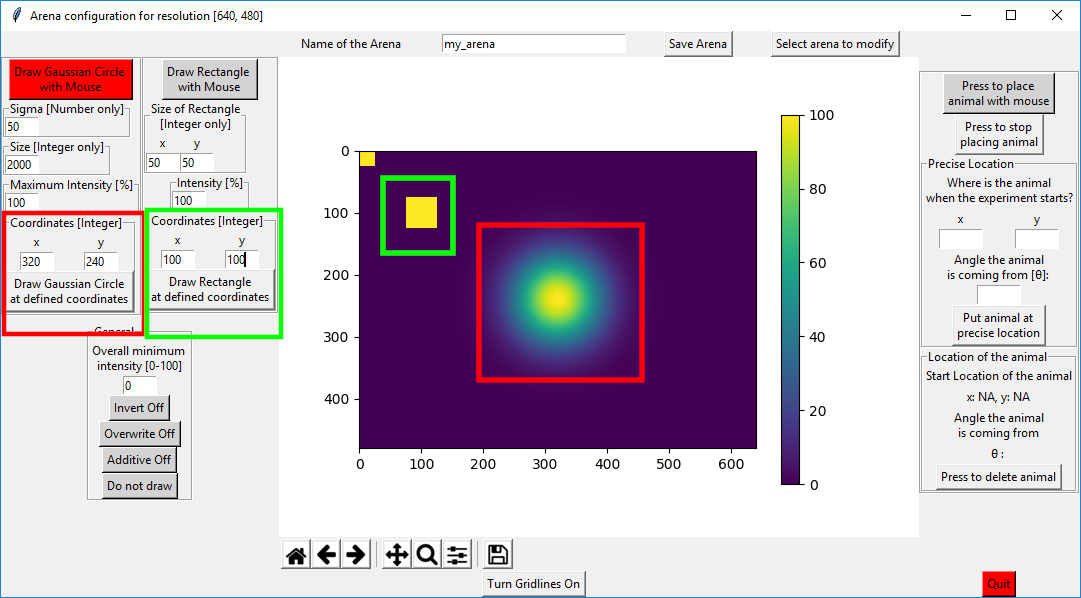

Supplement: S1 HTML — PiVR, Raspberry Pi Virtual Reality. (ZIP) [file pbio.3000712.s019.zip › S1HTLM/_images/9_1_DrawVRArena_example_1.png]

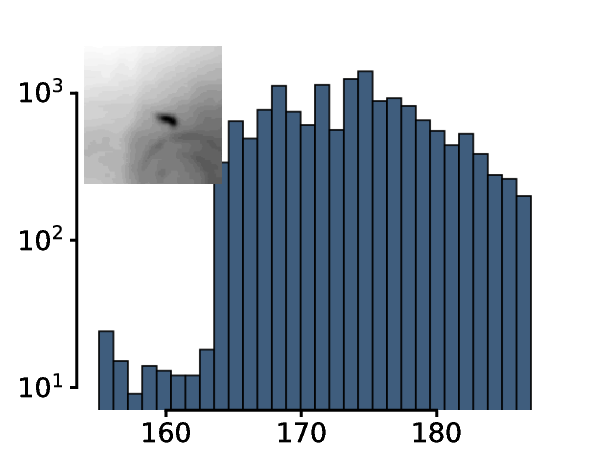

Supplement: S1 HTML — PiVR, Raspberry Pi Virtual Reality. (ZIP) [file pbio.3000712.s019.zip › S1HTLM/_images/Mode3ROI_histogram.png]

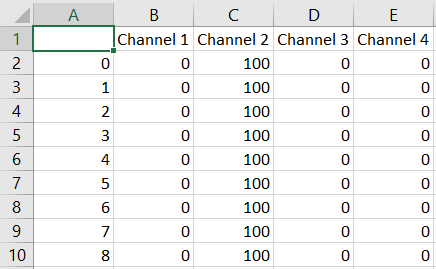

Supplement: S1 HTML — PiVR, Raspberry Pi Virtual Reality. (ZIP) [file pbio.3000712.s019.zip › S1HTLM/_images/TimeDepStimFile.png]

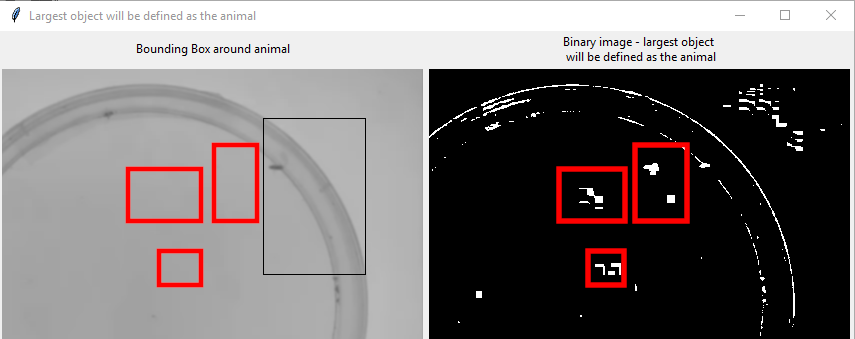

Supplement: S1 HTML — PiVR, Raspberry Pi Virtual Reality. (ZIP) [file pbio.3000712.s019.zip › S1HTLM/_images/17_detection_problem2.png]

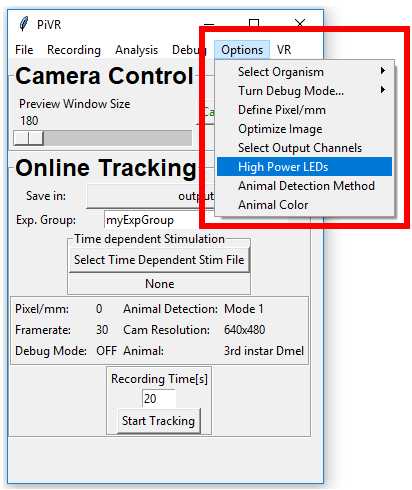

Supplement: S1 HTML — PiVR, Raspberry Pi Virtual Reality. (ZIP) [file pbio.3000712.s019.zip › S1HTLM/_images/OptionsHigLowPowerLEDSwitch.png]

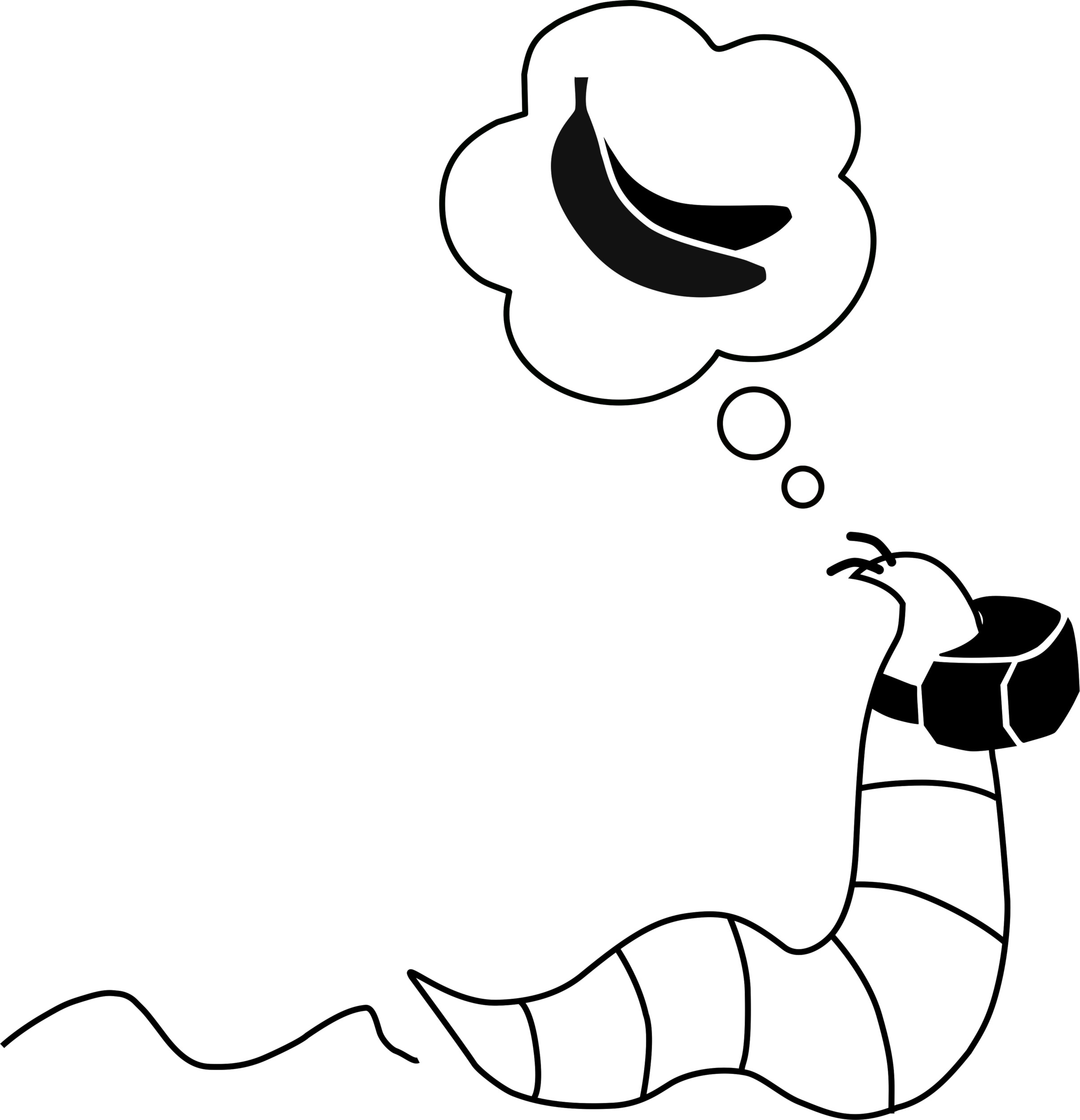

Supplement: S1 HTML — PiVR, Raspberry Pi Virtual Reality. (ZIP) [file pbio.3000712.s019.zip › S1HTLM/_images/PiVRLogo.jpg]

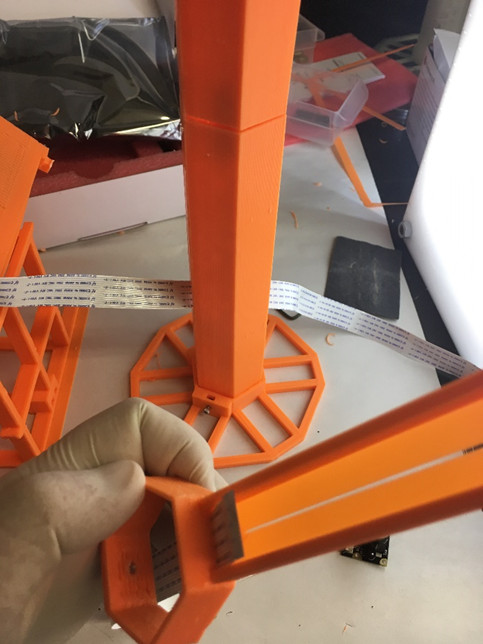

Supplement: S1 HTML — PiVR, Raspberry Pi Virtual Reality. (ZIP) [file pbio.3000712.s019.zip › S1HTLM/_images/17_Threading_of_Cam_cable.jpg]

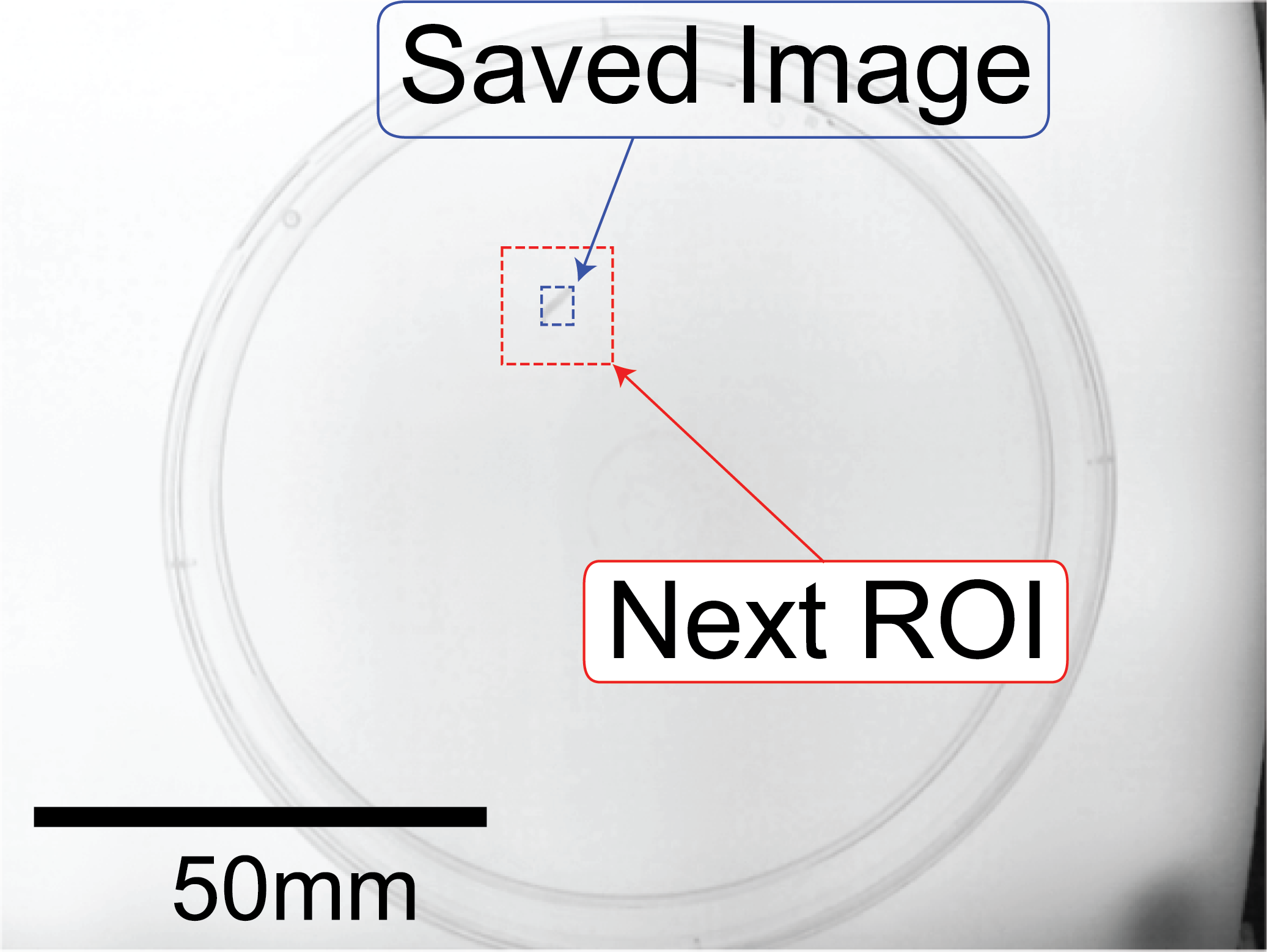

Supplement: S1 HTML — PiVR, Raspberry Pi Virtual Reality. (ZIP) [file pbio.3000712.s019.zip › S1HTLM/_images/FigS4_Animal_detected.png]

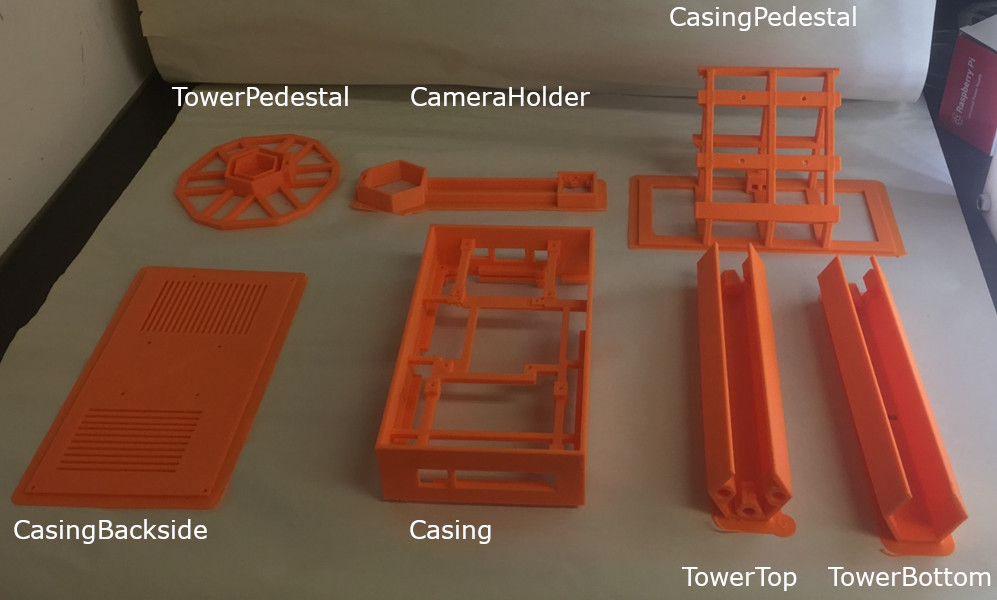

Supplement: S1 HTML — PiVR, Raspberry Pi Virtual Reality. (ZIP) [file pbio.3000712.s019.zip › S1HTLM/_images/1_3D_printed_parts.jpg]
