# Supplementary material for: PiVR: An affordable and versatile closed-loop platform to study unrestrained sensorimotor behavior
Source: PLoS Biol. 2020 Jul 14;18(7):e3000712. doi: 10.1371/journal.pbio.3000712 (PMC7360024; doi:10.1371/journal.pbio.3000712)
Supplement: Attachment — Submitted filename: 200306__PointResponse_v2.pdf [file pbio.3000712.s020.pdf]

Point-by-point responses to reviewers

We are grateful to the reviewers for their careful read and feedback on our work. As detailed below, we incorporated all their suggestions in the revised version of the manuscript. Reducing the discussion of the biological results streamlined the presentation of the methodology. We thank the reviewers for having encouraged us to implement this important change.

Rev. 1:
The present study by Tadres and Louis presents a useful and affordable open-source virtual reality setup based on the Raspberry Pi system. The authors present solid data using virtual chemotaxis and phototaxis experiments, confirming that their system works accurately for such purposes. I find this work valuable to the community, especially for the effort to design systems that can be implemented at low cost as well as for teaching platforms.

We thank the reviewer for highlighting that PiVR is a valuable tool for the community and a useful teaching platform.

I have a few comments that I believe would improve the presentation and message of this manuscript and one concern regarding the proposed speed of the system.

First, I have my doubts that the overall close-loop reaction time is correct. The shutter speeds and the image processing time were measured. However, due to the limitations of the bus, there is always a lag between image acquisition and the start of image processing. I have no experience with the Raspberry Pie system, but I have encountered these limitations in top of the line computing systems. Thus, I would test this directly. My suggestion is to use their system at maximal acquisition speed, image short LED flash, and use their computing platform to detect this flash and turn the same LED on for a second time. By looking at the number of frames between the two flashes, the authors could test the speed of the entire system. Although the sampling rate will be limited by the acquisition speed of the camera, the authors will be able to prove if their proposed 20ms processing time is accurate. This is important since the authors correctly claim that "the shorter the delay,
the more authentic the virtual reality is perceived." I don't see issues with the experiments presented, but new users will be warned in case the system is slower than the speeds required for their purposes.

We understand the authors' doubts about the short loop time that can be achieved with a Raspberry PI computer that costs $35. During the initial benchmarking of the tool, we were surprised by the short duration of the image acquisition and image processing time. Nonetheless, all our tests indicated that the processing time was shorter than 20 ms. The reviewer proposed a different and slightly more conservative approach that we implemented in the revised manuscript. By relying on the detection of light flashes in a video sequence recorded at high frame rate, an upper bound could be set on the overall loop time of PiVR. The only downside of this approach is that the loop time could only be resolved as multiples of the maximum frame rate. We empirically found that the maximum frame rate at which PiVR can reliably operate with a rate of skipped/dropped frames lower than 0.2% is 70 Hz (Supplementary

Figure S1Bii). At 70 Hz, we found that the latency (overall loop time) ranges between 14 ms and 28 ms and the manuscript now states the value “<30 ms” for latency. The methodology that led us to this conclusion is summarized in the new Supplementary Figure S1D-E. Further technical details are provided at the end of this point-by-point answer.

My second suggestion is regarding the current structure. The main claim of this paper is to have a VR system that is affordable and reliable; not the experimental results used to benchmark the system. Thus, I would remove the experimental sections from the discussion (Defining that nature of taste-driven responses in adult flies; Exploring the ability of zebrafish [larvae] to orient with minimal graded visual inputs; Exploring the ability of the fruit fly….) and merged them in a minimal form to the experimental results.

We have followed this suggestion. The discussion of the biological results has now been kept to a minimum (see fused section entitled “Exploring orientation behavior through virtual-reality paradigms”). This simplification makes it difficult to interpret and discuss the results presented in Figure 5 (larval chemotaxis in turbulent virtual odor plumes). Reviewer #2 noted that he/she had difficulties understanding Figure 5 in the original version of the manuscript. To place a clear focus on the methodology, we therefore decided to remove Figure 5 from the revised manuscript since this figure was not essential to benchmark PiVR.

Finally, although I truly appreciate and are convinced that the system is useful for particular experiments, it won't be for all. There are limitations on the FOV, camera resolution, speed, etc., that make it indispensable for many labs to engineering their systems. Thus, I would recommend adding this perspective to the discussion. I believe that this will be helpful for labs that thanks to the affordable design will start doing these kinds of VR experiments, but don't have the experience to judge all details.

We agree that the $23 CCD camera included the PiVR setup has clear limitations compared to CCD cameras that cost several hundred dollars. Therefore, we removed the sentence that pertained to the potential use of PiVR as a “cheap” video recording system to monitor behavior. Nonetheless, we added a video (S8 Movie) illustrating the grooming behavior of an adult fly with different lenses outfitting the PiVR setup. We will let the reader decide whether the resolution of this type of video is sufficient for applications that require higher precision than simply tracking the position of the head or the centroid.

Minor:
I would disagree with the first sentence: "Behavior emerges from the conversion of sensory input into motor output". Organisms are not automata, e.g.; there is a whole world of internal states that modulate behavior. Please rephrase.

Thank you for spotting this reductionistic perspective on sensorimotor control. We agree that internal states and memory play an important role in conditioning a behavioral response. Therefore, we remove the first sentence.

I would also make a stronger point on why VR systems are important (line 41). You don't need a VR system to stimulate an animal repeatedly. VR systems allow combining the stimulus to the behavior, enabling a precise exploration of response dynamics to sensory stimuli dependent on particular behavioral conditions.

We changed the text accordingly. Thank you.

- The paper sometimes uses colloquial terms, e.g., "crack the neuronal code," etc. I would change that language.

We have removed colloquial expressions from the revised version of the manuscript.

- Hz and frames per second are used, I would stick to Hz.

We changed the text to Hz accordingly. Thank you.

- Reference, e.g., page 213: Schulze, Gomez-Marin (17) -> Schulze et al. (17). Also line 307.

The formatting of the references was automatically generate by EndNote with one of the styles supported by PLoS. However, we converted the format of these two reference to regular numbers.

- Get rid of popular in line 220 and 256. Model organisms already imply that they are used widely.

We changed the text accordingly.

- Line 600 - 608, it is not clear that the (Ci-iii) refers to the supplemental figure.

We clarified the text. Thank you.

- Line 195, "field of the view", remove the.

We corrected this typo. Thank you.

Rev. 2:
In this manuscript, Tadres & Louis present PiVR, a novel hardware designed to perform closed-loop light-based stimulations on small animals. The authors present data based on optogenetic activation of genetically targeted neurons in Drosophila larvae and imagos; they also present a proof of principle using visible light on zebrafish larvae.

PiVR is certainly an interesting device and I can see it becoming popular in the field of Drosophila neuroscience, especially among people interested in developing new paradigms of learning.
The device has some clear strengths and some limitations. The strengths are discussed appropriately and fairly. The limitations not so much and it may be useful to have a more rounded discussion of both so that readers can immediately recognise whether this tool is appropriate for their uses.

Amongst the strengths I would count:

1. The documentation is outstanding.
2. The machine is relatively inexpensive.
3. The basic usage of the machine seems to be easy to implement

We believe that the reviewer has identified the main qualities of PiVR. This tool was designed in response to two needs: (1) a low-cost of production to make the parallel use of multiple setups possible for screening purposes; (2) a simple design and assembly to facilitate the use of PiVR for regular research by experimentalists who might not be comfortable with advanced programming and instrumentation or for teaching purposes. For this reason, we made significant efforts to document the construction of PiVR. As part of the revision, we have added a video illustrating the main steps, from 3D printing the parts to running an experiment (S7 Movie).

Amongst the weaknesses:

1. Some of the proposed usages of PiVR are suboptimal. It is stressed several times in the manuscript that PiVR can be used to acquire videos "offline", for them to be tracked by a different software (such as tracker.ai). I doubt this setup can compete, in terms of resolution, speed and even cost with the simpler solution of having an industrial CCD connected to an existing computer.

We have removed the statement about the use of PiVR to acquire videos with the in-built tracker (although this functionality has not been removed). While we agree with the reviewer that an industrial CCD camera connected to a powerful computer can produce videos with higher spatio-temporal resolution than PiVR, the point we wish to make with the off-line tracking mode is that the resolution of videos acquired with PiVR is sufficient perform identity tracking with the idtracker.ai software. This application is likely to be valuable to some users since idtracker.ai has become quite popular but its function requires images of good quality. The reader might want to know that PiVR is suitable for such applications at a cost that is 3 or 4 times lower than a setup with a good CCD camera connected to a regular computer.

2. While, in principle, the R in VR stands for any kind of Reality, in fact it is commonly associated to complex visual representations such as projections of objects, patterns, scenarios. PiVR is limited to providing different intensity of lights and therefore its main use-case is going to be optogenetics.

The reviewer is correct. Although virtual realities (VR) that are independent of light stimulations are possible (e.g., chemicals activating the thermosensors or temporal temperature ramps mimicking spatial temporal gradients), light-based optogenetic VR applications remain the most common type in systems neuroscience. In this manuscript, we have established the use of PiVR to VR applications elicited with light. Odor and taste gradients were produce through the optogenetic stimulation of the peripheral sensory of *Drosophila*. Graded virtual light gradients devoid of bilateral asymmetries and complex retinal representations were created with temporal changes in light intensity. We are now discussing the fact that VR created by PiVR are limited to temporal patterns of homogenous light stimulation.

3. It is not clear to me why a reader should prefer PiVR over other already existing alternatives and it may be useful to stress out, perhaps in a table, pros and cons of PiVR vs the "competition". FlyPI , BONSAI, FreeMO-VR are all alternative products with functionalities that overlap the ones of PiVR - the manuscript would benefit from a more straight comparison with them.

We thank the reviewer for this suggestion. To compare PiVR with other existing resources, we started by selecting the set of published tools that proposes a hardware + software solution permitting optogenetic-based virtual-reality experiments. Due to the innovative conception of FlyPi (its affordability and DIY philosophy inspired PiVR), we added this popular tool to the comparison table (S2 Table), even though FlyPI does not permit closed-loop tracking. Following the suggestion of the reviewer, we added the versatile tracking environment Bonsai, even though it does not come with a hardware.

I do not have specific comments on the manuscript, besides the fact that I found it perhaps too long and repetitive. I think the strength of this tool is that it does one thing and it does nicely but all this gets somehow lost in a manuscript so discursive and repetitive. The discussion, in particular, is not well focused.

We followed the reviewer's recommendation and refocused the manuscript on the presentation of the methodology. As advised by reviewer #1, we reduced the discussion of the biological results from three sections to a single section. In addition, we removed results that were not essential to demonstrate the capabilities of PiVR.

The figures are well presented and clear but perhaps not very focused. figures 2 and 3 read more like a demonstration that "optogenetics works". It would have been more useful to focus on PiVR versatility and show several different use cases rather than only those two very basic ones. Somehow the focus of the figures is optogenetics, not PiVR.

Regarding this point, we do not share the reviewer's opinion. We view it important to benchmark PiVR by reproducing published results. This is the main purpose of Figure 2. Testing the behavior of larvae in an odorant gradient that has the shape of a "volcano" is not a trivial application of PiVR. Figure 3 proposes a new biological application: the orientation of adult flies in a virtual checkerboard of bitter taste. The behavioral observations we are reporting are partly new. Aiming for more complex applications would seem out of the scope of this Methodology paper. Finally, Figure 4 establishes that PiVR can create virtual realities for zebrafish larvae. The application we propose is novel (creation of synthetic graded light landscapes) while enabling a comparison with published results in real light landscapes.

I also had troubles understanding part of figure 4 (F-J) and almost all of figure 5. Perhaps the concepts of slow vs fast dynamic VR can be explained in greater detail with a cartoon and examples of why and how an experimenter may want to use slow or fast dynamic VR could be provided.

We improved Figure 4I to clarify the meaning of the turn index and the conditional analysis. Concerning the interpretation of panels 4F-H, we are aware that the conclusions are not necessarily intuitive. The results of panel 4G reveal that fish turn more when they experience low-light intensity ranges. The increase in turn rate is particularly marked when fish move down-gradient while experiencing low-light intensity. The results of panel 4J show that fish do not have a turn bias toward the light gradient. While the results of Figure 4 can be related to previously published results, Figure 5 of the initial manuscript proposed a novel paradigm to investigate larval chemotaxis. Indeed, published work focused on characterizing the behavior of larvae in smooth and static gradients or conditions of white noise stimulation. The purpose of Figure 5 was to test the ability of larvae to track odor plumes without airflow. Given that the main objective of this paper is (now) to present a methodology, we did not have the space to develop a detailed analysis of larval chemotaxis in turbulent olfactory environments. Based on the reviewer's comments, we realized that the content and discussion of Figure 5 was probably insufficient to provide mechanistic insight into the ability of larvae to cope with turbulent olfactory stimulations. Such an analysis would require further data quantification. Given that both reviewers #1 and #2 encouraged us to focus the manuscript on the presentation of the methodology, we decided to remove Figure 5 from the revised manuscript.

Rev. 3:
This paper describes an open, low-cost platform to perform closed-loop behavioral experiments. The intention is to make such experiments more accessible or scalable by removing barriers of cost and development. The utility of the system is thoroughly demonstrated through example experiments in larval and adult Drosophila and zebrafish, which are analyzed to reveal new biological insights.

We thank the reviewer for appreciating the value of biological insights the virtual-reality paradigms enabled us to gain with PiVR. We believe these results complement the presentation of the methodology by showing the reader how relatively simple stimulation protocols can reveal interesting sensorimotor observations, which have the potential of representing the starting points of exciting future work. Our goal is to show that conducting such experiments does not necessitate sophisticated tools or advanced knowledge in engineering and programming.

The resources described in the paper are well designed and described and should be straightforward to apply to many current experimental questions. However, there are several existing platforms with overlapping aims and many of them are not cited or discussed in this paper. Some of these allow much more experimental flexibility than the generation of virtual environments through modulation of a one-dimensional signal that is used in the current paper. Therefore, it is not clear that the method fulfills the criteria required for a resource in PLOS Biology, in that it would enable experiments that were not possible using existing methods.

It is fortunate that our system neuroscience community has now access to various tools to conduct behavioral analysis through tracking and closed-loop stimulations. Our intention is not to present PiVR as the only tool that permits optogenetic-based virtual-reality experiments. That said, we firmly believe that PiVR has important qualities that make it distinct from other existing resources, which should address the concern about “novelty”. In the revised manuscript, we included a comparative table of the specifics of popular setups to track animals and create light-based virtual realities (S2 Table). This table highlights that PiVR is the tool-of-choice for researchers who are looking for a setup that is (i) easy to assemble, (ii) affordable and yet (iii) capable of rapid update rates compatible with applications involving fast-moving (small) animals such as adult flies and zebrafish. We agree that the use of a LCD projector can confer more flexibility to the stimulation protocols, but it also has limitations due to its higher price tag and the relatively low intensity of the projected light, which might not be sufficient to optogenetically activate neurons through opaque cuticles. For these reasons, it appears that no tool is suitable for all VR applications. The choice of the most appropriate tool should be guided by the nature of the application and the constraints of the user. The comparison S2 Table of the technical specifics of existing tools should help guide that choice.

In particular, it would be helpful to cite the following two resources and discuss them in comparison with the PIVR system:

1) Stytra
http://www.portugueslab.com/pages/resources.html
Stytra is a python-based package, which can be used for animal tracking and closed-loop presentation of stimuli. Implementations with low-cost hardware are possible. Štih V, Petrucco L*, Kist AM, Portugues R (2019)*
Stytra: An open-source, integrated system for stimulation, tracking and closed-loop behavioral experiments. PLOS Computational Biology, doi:10.1371/journal.pcbi.1006699

We have now added references to Stytra in the manuscript. This tool has also been included in the comparisons of the new S2 Table. We believe that Stytra is an excellent tool. The difference with PiVR is that it was conceived predominantly for zebrafish (e.g., the left and right eyes being features of interest being monitored). Although we have no doubt that Stytra can be applied to study other model organisms, such uses will require adaption of the source code that might not be trivial for experimentalists not versed with programming. Moreover, the estimated construction costs of Stytra are 3 to 4 times higher than PiVR, even in its cheapest configuration. For this reason, we do not think Stytra would be the most appropriate choice for labs that are not working on zebrafish or for individuals who are seeking to run behavioral screens with multiple assays. The simple design of PiVR and the documentation of its assembly should also be advantageous for pure teaching purposes.

2) Bonsai.
http://www.kampff-lab.org/bonsai
Bonsai is a software framework that is widely used for designing closed-loop behavior experiments, and can be used in conjunction with low cost hardware.
Bonsai: An event-based framework for processing and controlling data streams. Lopes G, Bonacchi N, Frazão J, Neto JP, Atallah BV, Soares S, Moreira L, Matias S, Itskov PM, Correia PA, Medina RE, Calcaterra L, Dreosti E, Paton JJ, Kampff AR. Frontiers in Neuroinformatics. 2015; 9:7.

We have added references to Bonsai to the manuscript and in the comparative S2 Table. Bonsai is a versatile tracking system. However it is very different from PiVR since it does not come with an integrated hardware. The philosophy of PiVR differ from Bonsai since the starting point of PiVR is a simple, affordable yet powerful hardware on which a versatile closed-loop tracking algorithm was built to conduct virtual-reality experiments. Thus, Bonsai appears the tool-of-choice for researchers who have already settled with a (sophisticated) assay for which a software is needed. By contrast, PiVR might be preferred by experimentalists who are looking for an integrated hardware and software package that can be built without being technically savvy.

Some minor issues:

1) In the introductory discussion on virtual reality in neuroscience, it could be worth citing the productive use of virtual reality in work in zebrafish, in the context of motor adaptation, prey capture and social

behavior.

We thank the reviewer for suggesting the additions of these new references. In the introduction, we are now referring to the application of VR to study motor adaption (Ahrens et al 2013, Frontiers), prey capture (Bianco & Engert 2015, Current Biology) and social behavior (Stowers et al. 2017, Nature Methods).

2) The closed-loop experiments based on optogenetic stimulation of the gustatory system in flies have technical similarities with experiments in the following paper, which might be appropriate to cite: Moreira, J.-M., Itskov, P. M., Goldschmidt, D., Baltazar, C., Steck, K., Tastekin, I., et al. (2019). optoPAD, a closed-loop optogenetics system to study the circuit basis of feeding behaviors. eLife, 8. http://doi.org/10.7554/eLife.43924

We have now added this reference to the manuscript. We agree that the optogenetic activation of the bitter taste neurons is common in our study and the work of Moreira et al. However, the behavioral read-out is distinct. In our work, we are studying the effect of bitter taste sensing on the organization of spatial exploration (foraging) while Moreira et al. 2019 is more focused on food ingestion.

3) The terminology used in the zebrafish swimming experiments in not aligned to common usage in the literature, and could be confusing. Individual bursts of movement of the larvae are typically called 'bouts' , and the word 'scoots' is used to refer to a particular type of bout, where the tail oscillations are mostly confined to the caudal region, and which propels the larva forward at a slow speed, without major reorientation or head yaw (also often called 'Slow swims'). Routine turns, by contrast, start with a larger tail movement that reorients the larva, which can be followed by a propulsive phase. Therefore what are called 'scoots' in this paper may encompass both 'scoots' and 'turns' in the terminology of other studies.

Thank you for spotting this inconsistent use of terminology. Throughout the manuscript, we changed our previous use of “scoots” for swim “bouts”.

4) In the discussion of phototaxis in zebrafish, there are some other papers that perhaps are relevant to cite, because they either describe virtual reality based assays for phototaxis, or discuss the choice of turns vs scoots and the direction of turns. Ahrens, M. B., Huang, K. H., Narayan, S., Mensh, B. D., & Engert, F. (2013). Frontiers in Neural Circuits, 7, 104.

Fernandes, A. M., Fero, K., Arrenberg, A. B., Bergeron, S. A., Driever, W., & Burgess, H. A. (2012). Current Biology, 22(21), 2042-2047.

Horstick, E. J., Bayleyen, Y., Sinclair, J. L., & Burgess, H. A. (2017). BMC Biology, 1-16.

We have now included the suggested references, which are strengthening the discussion of the results related to the fish phototactic experiments. Again, we are deeply grateful for the insightful suggestions of the reviewer.

Detailed discussion of the loop-time assessment featured in Sup. Fig. S1E-F

The information below complements the description of the methodology included in the revised manuscript. We added this discussion to ensure that the reviewers have access to all the details related to the implementation of the loop-time quantification proposed by reviewer #1. As we felt that this information would too detailed for reader, we did not include in the manuscript but we intend to add it to the PiVR website (www.pivr.org).

As described in Supplementary Figure S1E-F, we implemented the experiment suggested by reviewer #1. We found that the latency between an action of the animal and the instantiation of the LED update is shorter than 30 ms. This result is based on two measurements: (1) the **timestamp of the frame** and (2) the **shutter speed** of the camera. These two values are recorded based on the (sub)millisecond precision of the internal clocks of the real-time operating software of the GPU and the camera, respectively (see documentation: https://picamera.readthedocs.io/en/release-1.13/fov.html#division-of-labor).

Practically, we used a normal PiVR setup, removed the long-pass filter to detect the red stimulus light and amended the PiVR software by the following:

1) At each 50th frame of a trial (and multiples thereof), turn the red LED on.
2) Take the median of a set of 10 pixels in the search box and compare the average current intensity to the intensity of the previous frame.
3) If this difference is larger than 2, consider the LED “ON” and immediately turn the LED “OFF”.
4) If the LED has been turned “OFF” in the previous frame, turn the LED “ON” again.

The code used for this control can be found on the Gitlab repository of PiVR (https://gitlab.com/LouisLab/pivr) in branch ‘LED_flash_test’ in the file ‘fast_tracking.py’ with relevant code going from line 1457 to 1584 (https://gitlab.com/LouisLab/pivr/-/blob/LED_flash_test/PiVR/fast_tracking.py). In addition, we added the data and analysis script to the dryad repository.

Using the methodology described above, we found that the detection of a ‘LED ON’ event was either detected during the earliest possible frame or one frame later. This difference in latency was dependent on the region of the image (region of interest, ROI) used to compare the pixel intensity. The detection latency was systematically longer when the ROI was in the top as compared to the bottom of the image (see Supplementary Figure S1F). As illustrated in the figure below, this can be explained by the fact that turning the LED “ON” **while** the camera is grabbing the frame through the rolling shutter cannot lead to a successful detection of the change in light intensity. During frame #50 the LED is still “OFF” in the top part of the image

whereas it is “ON” in the lower part of the image.

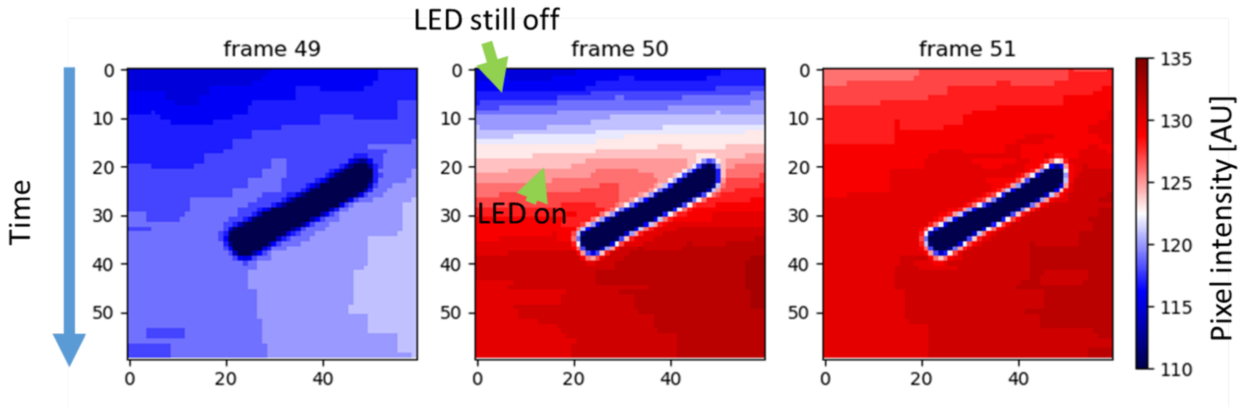

As shown in Supplementary Figure S1Fiii, the number of frames between two consecutive events corresponding to the LED being turned “ON” was 3 frames if the ROI was located in the top part of the image. The schematic diagram below outlines our interpretation of this 3-frame delay: The LED is being turned “ON” during frame (j) while the frame is being recorded (vertical arrow in frame j + 1). If the PiVR algorithm is set to monitor a ROI in the upper part of the image, this ROI is read before the LED intensity has switched to the “ON” state. Therefore, the LED “ON” event is **not** observed during frame j. Instead, it is detected during processing of frame j + 1. This delay happens again when the second LED “ON” event is detected in frame j + 4.

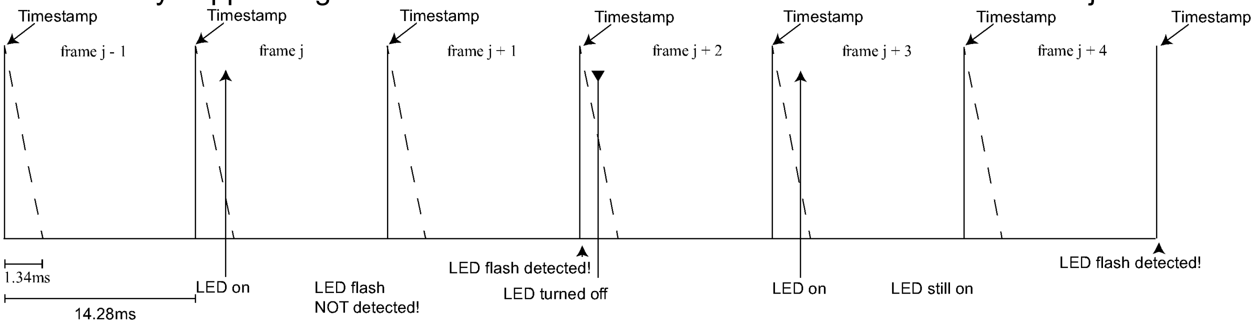

By contrast, if PiVR is set to monitor a ROI in the lower part of the image, the LED turn “ON” event can detected while processing frame j + 1 (see below).

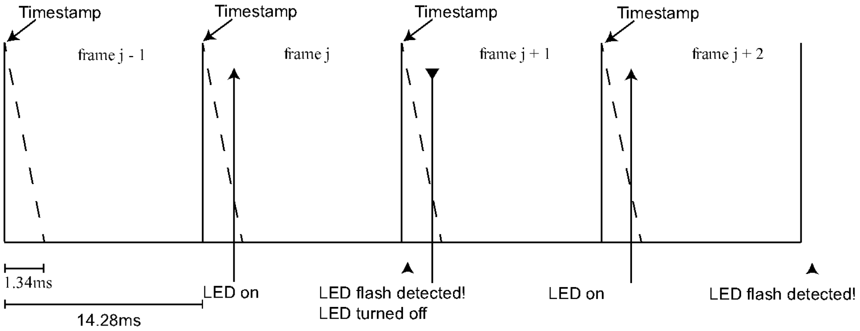

Consequently, the time that elapse between the two LED “ON” events is either 5 frames (or X ms at 70 Hz) for a ROI in the top part of the image or 2 frames for a ROI in the bottom part of the image.

Based on these observations, we conclude that the LED “ON” event must be happening **while** the image is being captured, which corresponds to a duration of 1.34ms. The longest possible latency between the action of an animal (in this example during frame j) and the actuation of the update of the LED is therefore 2 frames + 1.34ms (time window associated with frame j + 2), which is equivalent to less than 30 ms (frame duration at 70 Hz is 14.29 ms).
