# Supplementary material for: PiVR: An affordable and versatile closed-loop platform to study unrestrained sensorimotor behavior
Source: PLoS Biol. 2020 Jul 14;18(7):e3000712. doi: 10.1371/journal.pbio.3000712 (PMC7360024; doi:10.1371/journal.pbio.3000712)
Supplement: Attachment — Submitted filename: Point-by-point_answer_final.pdf [file pbio.3000712.s021.pdf]

Below is our point-by-point answer to the requests that we received on April 27, 2020. As described below, most of these requests had been addressed in the manuscript we resubmitted on April 10, 2020. We believe we have now addressed all other pending issues.

Dear Dr Louis,

Thank you for resubmitting your manuscript "PiVR: an affordable and versatile closed-loop platform to study unrestrained sensorimotor behavior" to PLOS Biology. We noticed a few remaining issues that will need to be addressed before your manuscript can be formally accepted. Your submission has been sent back to you to enable you to make these changes.

We would appreciate your prompt cooperation with our requests at your earliest convenience. If you have any questions or require clarification, please do not hesitate to get in touch.

MANUSCRIPT REQUIREMENTS

Please update your manuscript with the following changes:

1. Please remove your Data Statement section from your manuscript file as this information is automatically pulled from the Data Availability question on the submission form.

We removed the Data Statement section from the manuscript file.

2. Please remove the funding information from your manuscript file. The published version is pulled automatically from the Financial Disclosure section of the submission form.

We removed Financial Disclosure from the manuscript file.

3. Your references need to be formatted according to the ICMJE standard reference style. PLOS uses the numbered citation [citation-sequence] method and first six authors, et al. Authors should be listed as "Last name, First Initial". Please note that references should be cited in square [ ] brackets and not round ( ) brackets to ensure that they are correctly typeset. Additionally, the reference list should be formatted as “1.” and not “[1]”. Please adhere to the PLOS style for all references. For a full outline see our Author Guidelines: http://journals.plos.org/plosbiology/s/submission-guidelines#loc-references

To the best of our knowledge, the references were already formatted according to your guidelines. Citations were generated with EndNote. To avoid any problems, we downloaded the PLoS.ens style from your website: http://journals.plos.org/plosbiology/s/submission-guidelines#loc-references. We re-updated all citations with the style you provided. However, we noticed that the format of the citations is not fully consistent with the request above for a "Last name, First Initial" format. Also, the citations in bracket include a space between the numbers ([12, 13] versus [12,13]). We did not fix these potential problems since we assume that the PLoS.ens style distributed through your website is correct and up-to-date.

4. Please put all reference citations in square brackets, with no spaces, separated by either commas or dashes, e.g. [1,2,3] or [1-3].

In the previous resubmission, the references were already in square brackets and either separated by commas of dashes with no spaces.

Following the instructions of point 3, we downloaded the PLoS.ens style from your website: http://journals.plos.org/plosbiology/s/submission-guidelines#loc-references. We re-updated all citations with the style you provided. We noticed that the PLoS.ens creates a space between the numbers inside the citation brackets. As we assume that the PLoS.ens style distributed through your website is correct and up-to-date, we did not modify this PLoS.ens style to remove the extra spaces in the citations.

5. Please make sure all references are cited in ascending numerical order in the text.

We confirm that all references are cited in ascending numerical order. The citations were generated with EndNote using the PLoS.ens style distributed through your website.

6. Please ensure that each supporting information file has a legend at the end of the manuscript file, after the Reference list.

We added legend to S1 HTML file at the end of the manuscript list. No further changes appeared to be needed for the legend of the other supporting information (S1-S8 Fig, S1-S8 Movie, S1-S2 Table).

7. Please make sure that the Dryad DOI is active to give us access as we need to check the data before the manuscript is accepted for production.

We confirm that the DOI of the datadryad is https://doi.org/10.25349/D9ZK50 as indicated throughout the manuscript.

*We received a "private" link for **peer review purposes** which can be used to inspect the data before publication: https://datadryad.org/stash/share/EeCn7Fg8JzwHNSqO6k5YyLalzBE9D4_VKI9Jnwg4ilA*

8. You should also indicate in all the corresponding figure legends where the data can be found. We need the data underlying the following figures:
Fig. 3C, F; Fig. 4G, H, J; Fig. S1B, C, D; Fig. S3E, D, I, J; Fig. S4E, F; Fig. S7A, B, C and Fig. S8A, B.

We added the following statement : All data used to create these plots is available from https://doi.org/10.25349/D9ZK50" below Fig. 3, Fig 4, Fig S1, Fig S3, Fig S4, Fig S7 and Fig S8. In addition, we added the statement to Fig. 2.

9. Thank you for providing a Striking Image. Please upload a Word Doc in which you include a caption, credit, and confirmation that the image can publish under the Creative Commons Attribution License (https://creativecommons.org/licenses/by/4.0/).

As instructed, we have uploaded a word document with the necessary information.

FIGURE REQUIREMENTS

Please complete the following instructions regarding your figure files:

1. Please ensure that all main figure files follow PLOS naming conventions in the File Inventory. The file name should be in the format “Fig1.tif” and the file description should be “Fig 1”.

We confirm that files are named “Fig1.tif” and are referred to as “Fig 1” in the manuscript.

2. Please ensure that all main figure files are cited in ascending numerical order in the main text of the article.

We confirm that all main figure files are cited in ascending numerical order in the main text of the article.

3. Please ensure that all main figures are referenced in the manuscript as Fig 1, Fig 2, etc. (including capitalization), rather than Figure 1, Figure 2, etc. Please note, however, that the file names themselves must not include the space, i.e. Fig1.tif, Fig2.eps.

Please use the following examples of naming conventions for citing multiple figures in your manuscript:
- Figs 3 and 4
- Figs 3, 4, and 7
- Figs 1-5
- Fig 3A and 3B
- Figs 3A and 7B

We confirm that we followed the naming convention indicated above.

4. Place each main figure legend directly below the paragraph in which that figure is first referenced.

We confirm that the main figure legend is placed below the paragraph in which the figure is first referenced.

5. Please note that all figures and supporting information files will be published under the Creative Commons Attribution License (creativecommons.org/licenses/by/4.0/). Under this license, authors retain ownership of the copyright for their article, but allow anyone to download, reuse, reprint, modify, distribute, and/or copy articles in PLOS journals for any reason, even commercially, so long as the original authors and source are cited. Permission is not required from PLOS or authors to reuse any content as long as proper attribution is provided.

We ask that you please confirm via email to plosbiology@plos.org that you either created the following figure panels or have written permission from the copyright holder to publish them under a CC-BY license:

- larvae illustrations in Fig 4 Panels F and I

- clock photo in Supplementary figure 1A
- photographs in Supplementary figure 2 A-D
- Kelp fly, spider, firefly and pill bug photographs in Supplementary figure 6 A-D

Please note that any written confirmation you send to us must clarify that the copyright holder understands and agrees to the terms of this license. Note that an email confirmation is acceptable.

As instructed, we sent an email to plosbiology@plos.org on May 14 with the following content:

> *To whom it may concern:*
>
> *We hereby state that*
>
> *The larval illustrations in Fig 4 Panels F and I, the picture of the clock shown in Supplementary figure 1A, the photographs in supplementary figure 2A-D and the image of the Kelp fly in supplementary figure 6A were created by David Tadres, the first author of the paper. Mr. Tadres agrees to the Creative Commons Attribution License which states:*
>
> *Please note that all figures and supporting information files will be published under the Creative Commons Attribution License (creativecommons.org/licenses/by/4.0/). Under this license, authors retain ownership of the copyright for their article, but allow anyone to download, reuse, reprint, modify, distribute, and/or copy articles in PLOS journals for any reason, even commercially, so long as the original authors and source are cited. Permission is not required from PLOS or authors to reuse any content as long as proper attribution is provided.*
>
> *Regarding the photograph of the firefly and the pill bug in S6C-D. The copyright holder of this work, Igor Siwanowicz, has given us permission to publish them under the Creative Commons Attribution License. See screenshot at the end of this email.*
>
> *Regarding the photograph of the spider in S6B. The copyright holder of this work, Stella Glasauer, has given us permission to publish the photograph under the Creative Commons Attribution License. See screenshot at the end of this email.*

Additionally, we are unable to publish S8 Movie, as it currently contains an identifiable logo. Please blur out the below logo, or edit the clip to remove the shots where the logo is visible:

- Raspberry Pi logos

We confirm that the Raspberry Pi logos in Movie S8 has been blurred.

You will find your submission in the 'Submissions Sent Back to Author' folder. Once you have made these changes, please re-approve the submission following the same steps as before.

We have proceeded as instructed and hope that all pending issues have been resolved. Thank you for your assistance with the publication of this manuscript.
